# Polycyclic heteroaromatics *via* hydrazine-catalyzed ring-closing carbonyl–olefin metathesis†

**DOI:** 10.1039/d1sc06234d

**Published:** 2022-02-01

**Authors:** Eun Kee Cho, Phong K. Quach, Yunfei Zhang, Jae Hun Sim, Tristan H. Lambert

**Affiliations:** Department of Chemistry and Chemical Biology, Cornell University Ithaca New York 14853 USA tristan.lambert@cornell.edu

## Abstract

The use of hydrazine-catalyzed ring-closing carbonyl–olefin metathesis (RCCOM) to synthesize polycyclic heteroaromatic (PHA) compounds is described. In particular, substrates bearing Lewis basic functionalities such as pyridine rings and amines, which strongly inhibit acid catalyzed RCCOM reactions, are shown to be compatible with this reaction. Using 5 mol% catalyst loadings, a variety of PHA structures can be synthesized from biaryl alkenyl aldehydes, which themselves are readily prepared by cross-coupling.

## Abstract 2

Hydrazine catalysis enables the ring-closing carbonyl–olefin metathesis (RCCOM) to form polycyclic heteroaromatics, especially those with basic functionality.

## Graphical abstract

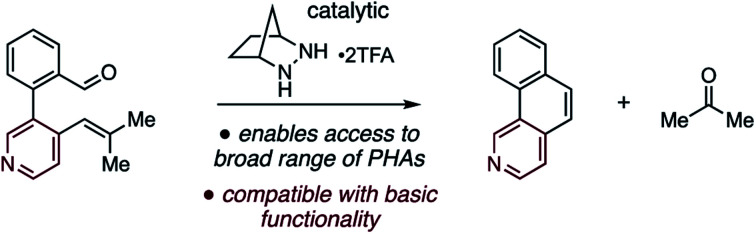

Polycyclic heteroaromatic (PHA) structures comprise the core framework of many valuable compounds with a diverse range of applications (Fig. 1A).^1^ For example, polycyclic azines (*e.g.* quinolines) are embedded in many alkaloid natural products, including diplamine^2^ and eupolauramine^3^ to name just a few. These types of structures are also of interest for their biological activity, such as with the inhibitor of the Src-SH3 protein–protein interaction shown in Fig. 1A.^4^ Many nitrogenous PHAs are also useful as ligands for transition metal catalysis, as exemplified by the widely used ligand 1,10-phenanthroline.^5^ Meanwhile, chalcogenoarenes^6^ such as dinaphthofuran^7^ and benzodithiophene^8^ have attracted high interest for both their medicinal properties^9^ and especially for their potential use as organic light-emitting diodes (OLEDs), organic photovoltaics (OPVs), and organic field-effect transistors (OFETs).^10^ These and numerous other examples have inspired the development of a wide variety of strategies to construct PHAs.^1,11–14^ Although these approaches are as varied as the structures they target, the wide range of molecular configurations within PHA chemical space and the challenges inherent in exerting control over heteroatom position and global structure make novel syntheses of these structures a topic of continuing interest.

**Fig. 1:**
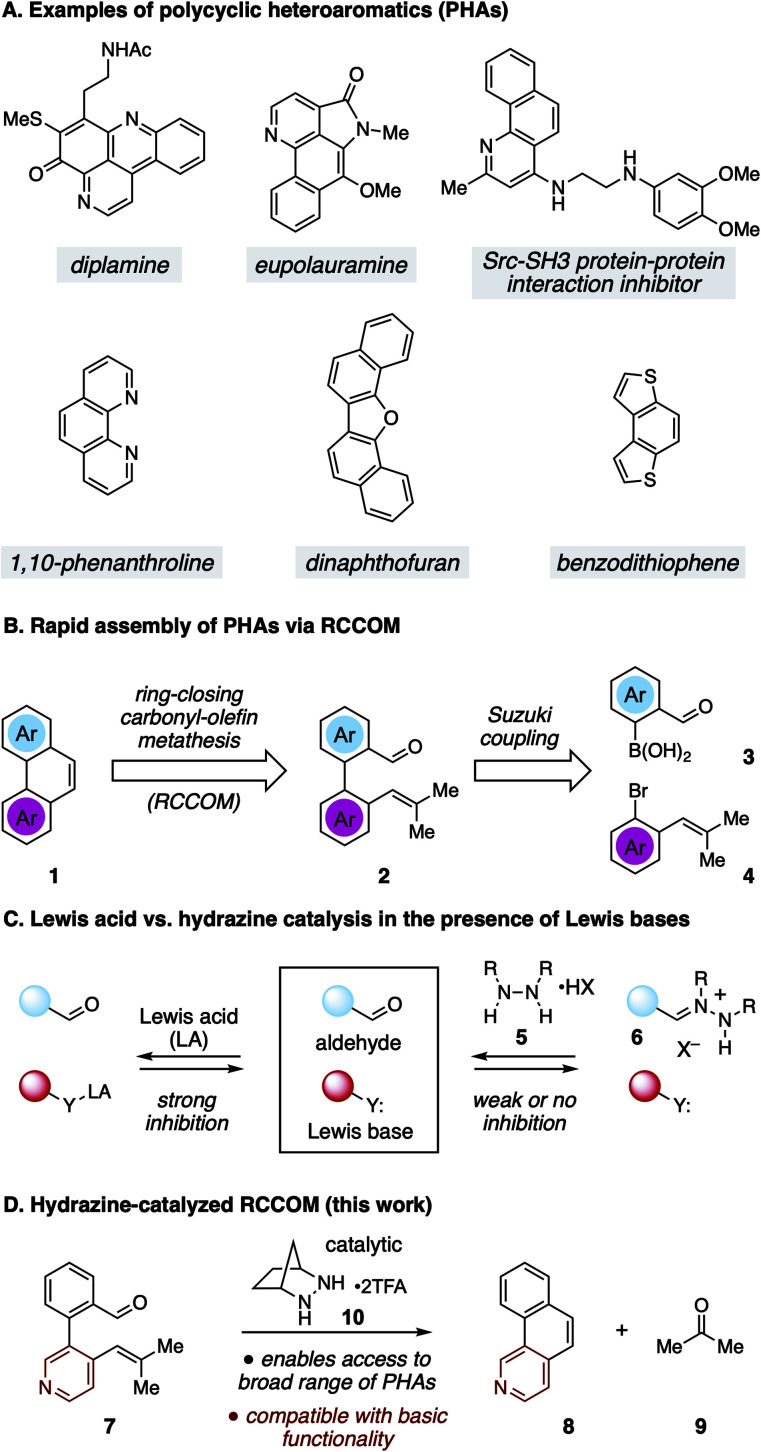
(A) Examples of PHAs. (B) RCCOM strategy for PHA synthesis. (C) Lewis base inhibition for Lewis acid *vs.* hydrazine catalyzed RCCOM. (D) Hydrazine-catalyzed RCCOM for PHA synthesis.

One potentially advantageous strategy for PHA synthesis is the use of ring-closing carbonyl–olefin metathesis^15^ (RCCOM) to forge one of the PHA rings, starting from a suitably disposed alkenyl aldehyde precursor 2 that can be easily assembled by cross-coupling (Fig. 1B). In related work, the application of RCCOM to form polycyclic aromatic hydrocarbons (PAHs) was reported by Schindler in 2017.^16^ In this case, 5 mol% FeCl_3_ catalyzed the metathesis of substrates to form phenanthrenes and related compounds in high yields at room temperature. This method was highly attractive for its efficiency, its use of an earth-abundant metal catalyst, and the production of benign acetone as the only by-product. Nevertheless, one obvious drawback to the use of Lewis acid activation is that the presence of any functionality that is significantly more Lewis basic than the carbonyl group can be expected to strongly inhibit these reactions (Fig. 1C). Such a limitation thus renders this method incompatible with a wide swath of complex molecules, especially PHAs comprised of azine rings. This logic argues for a mechanistically orthogonal RCCOM approach that allows for the synthesis of PHA products with a broader range of ring systems and functional groups.

We have developed an alternative approach to catalytic carbonyl–olefin metathesis that makes use of the condensation of 1,2-dialkylhydrazines 5 with aldehydes to form hydrazonium ions 6 as the key catalyst–substrate association step.^17–19^ This interaction has a much broader chemoorthogonality profile than Lewis acid–base interactions and should thus be much less prone to substrate inhibition than acid-catalyzed approaches. In this Communication, we demonstrate that hydrazine-catalyzed RCCOM enables the rapid assembly of PHAs bearing basic functionality (Fig. 1D).

For our optimization studies, we chose biaryl pyridine aldehyde 7 as the substrate (Table 1). Using 5 mol% of the hydrazine salt 10 furnished the RCCOM product 8 in 67% yield after 15 h at 80 °C in a sealed vial with THF as a solvent (entry 1). The [2.2.2]-bicyclic hydrazine^20^ salt 11 was also productive (entry 2), albeit somewhat less so. Notably, iron(iii) chloride generated no conversion at either ambient or elevated temperatures (entries 3 and 4). Trifluoroacetic acid (TFA) was similarly ineffective (entry 5). Meanwhile, a screen of various solvents revealed that, while the transformation could occur in a range of media (entries 6–9), THF was optimal. Finally, by raising the temperature to 90 °C (entry 10) or 100 °C (entry 11), up to 96% NMR yield (85% isolated yield) of adduct 8 could be obtained in the same time period.

**Table 1:** Optimization studies^a^

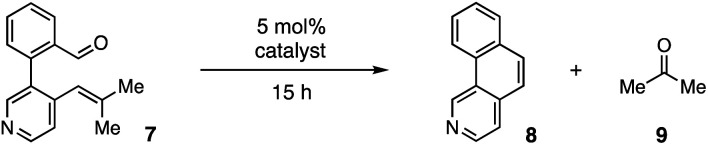				
Entry	Catalyst	Solvent	Temp. (°C)	8 yield (%)
1	10	THF	80	67
2	11	THF	80	53
3	FeCl_3_	DCE	rt	0
4	FeCl_3_	DCE	80	0
5	TFA	THF	80	0^b^
6	10	i-PrOH	80	31
7	10	CH_3_CN	80	28
8	10	EtOAc	80	26
9	10	Toluene	80	24
10	10	THF	90	87
11	10	THF	100	96^c^
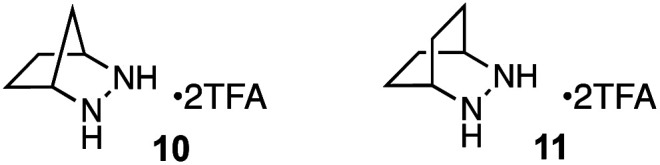				

a Conditions: substrate 8 (0.2 mmol) and 5 mol% catalyst in 0.4 mL of solvent (0.5 M) in a 5 mL sealed tube were heated to the temperature indicated for 15 h. Yields were determined by ^1^H NMR using CH_2_Br_2_ as an internal standard.

b 2 equiv. of TFA was used.

c 85% isolated yield.

Using the optimized conditions, we explored the synthesis of various PHAs (Fig. 2). In addition to benzo[*h*]isoquinoline (8), products 12 and 13 with fluorine substitution at various positions could be generated in good yields. Similarly, benzoisoquinolines 14 and 15 bearing electron-donating methoxy groups and the dioxole-fused product 16 were also accessed efficiently. Furthermore, a phenolic ether product 17 with a potentially acid-labile *N*-Boc group was generated in modest yield. We found that an even more electron-donating dimethylamino group was also compatible with this chemistry, allowing for the production of 18 in 68% yield. On the other hand, adduct 19 bearing a strongly electron-withdrawing trifluoromethyl group was isolated in only modest yield. The naphtho-fused isoquinoline 20 could be generated as well; however, 20 mol% catalyst was required to realize a 35% yield. The thiophene-fused product 21 was furnished in much better yield, also with the higher catalyst loading. Although not a heterocyclic system, we found that the reaction to form phenanthrene (22) was well-behaved, providing that compound in 83% yield. In addition, an amino-substituted phenanthrene 23 was also formed in good yield. Other thiophene-containing PAHs such as 24–26 were produced efficiently. On the other hand, adduct 27 was generated only in low yield. Naphthofuran (28), which is known to have antitumor and oestrogenic properties,^21^ was synthesized in good yield. Finally, pharmaceutically important structures such as benzocarbazole^22^29 and naphthoimidazole^23^30 could be accessed in moderate yields with increased catalyst loading.

**Fig. 2:**
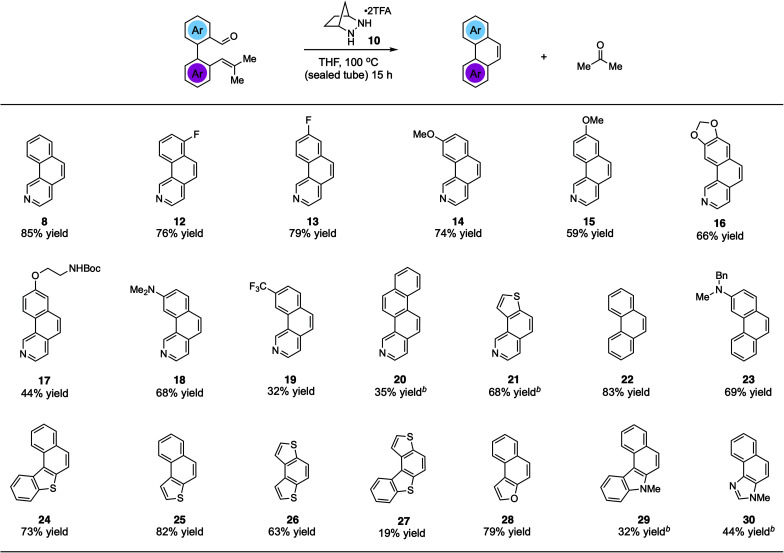
Substrate scope studies for hydrazine 1-catalyzed RCCOM synthesis of polycyclic heteroaromatics. ^*a*^ Conditions: substrate and catalyst 1·(TFA)_2_ (5 mol%) in THF (0.5 M) were heated to 100 °C in a 5 mL sealed tube for 15 h. Yields were determined on purified products. ^*b*^ 20 mol% catalyst.

We also examined the scope of the olefin substitution pattern (Table 2). For this screen, we reacted biaryl substrates with 5 mol% hydrazinium salt 10 in THF at 100 °C in a sealed vial. The trisubstituted 2-methyl-1-propenyl substrate 7 was by far superior to the others in terms of reactivity and yield (entry 1), reaching full conversion in 15 h as already discussed.

**Table 2:** Screen of the olefin substituent^a^

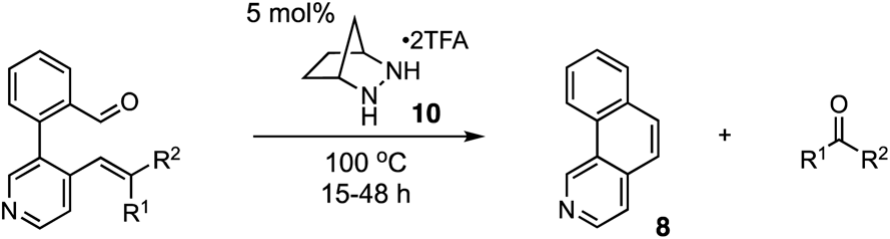			
Entry	Substrate	Time (h)	Yield (%)
1	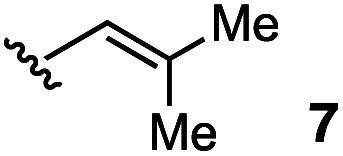	15	96
2	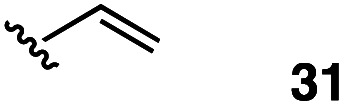	48	5
3^b^	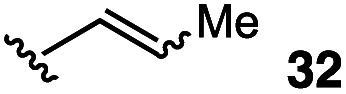	48	27
4	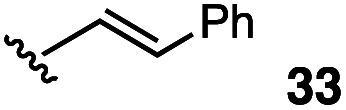	48	54
5	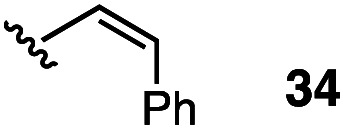	48	64

a Conditions: 5 mol% 10 in THF (0.5 M) in a 5 mL sealed tube were heated to the temperature indicated for 15–48 h. Conversions and yields were determined by ^1^H NMR using CH_2_Br_2_ as an internal standard.

b Mixture of *E*/*Z* (2 : 1) isomers.

The vinyl substrate 31 led to very little desired product (entry 2), while the propenyl substrate 32 (2 : 1 mixture of *E* and *Z* isomers) was somewhat improved but still low-yielding (entry 3). Finally, styrenyl substrates 33 and 34 (entries 4 and 5) led to improved yields relative to 31 and 32, with the *cis* isomer 34 being slightly more efficient (entry 5).

In order to better understand the facile nature of this RCCOM reaction, we conducted DFT calculations for each step of the proposed reaction pathway (Fig. 3A). Condensation of the substrate 7 with [2.2.1]-hydrazinium 10 to afford the hydrazonium *Z*-35 was found to be exergonic by −13 kcal mol^−1^. Isomerization of *Z*-35 to *E*-35 comes at a cost of ∼3 kcal mol^−1^, but the total activation energy for cycloaddition (*cf.*36), taking into account this isomerization, was still relatively modest at only +21.0 kcal mol^−1^ with an overall exergonicity of −11.1 kcal mol^−1^. The energetic change for proton transfer in the conversion of cycloadduct 37a to the cycloreversion precursor 37b was negligible (+1.2 kcal mol^−1^). Interestingly, including the proton migration step, the cumulative energy barrier for cycloreversion 38 was found to be only +21.7 kcal mol^−1^, nearly the same as for the cycloaddition. Undoubtedly, the formation of an aromatic ring greatly facilitates this step relative to other types of substrates. Unsurprisingly, the cycloreversion to produce benzoisoquinoline 8 along with hydrazonium 39 was calculated to be strongly exergonic. Finally, the hydrolysis of 39 to regenerate hydrazinium catalyst 10 (and acetone) required an energy input approximately equal to that gained from the condensation with the substrate to form 35.

**Fig. 3:**
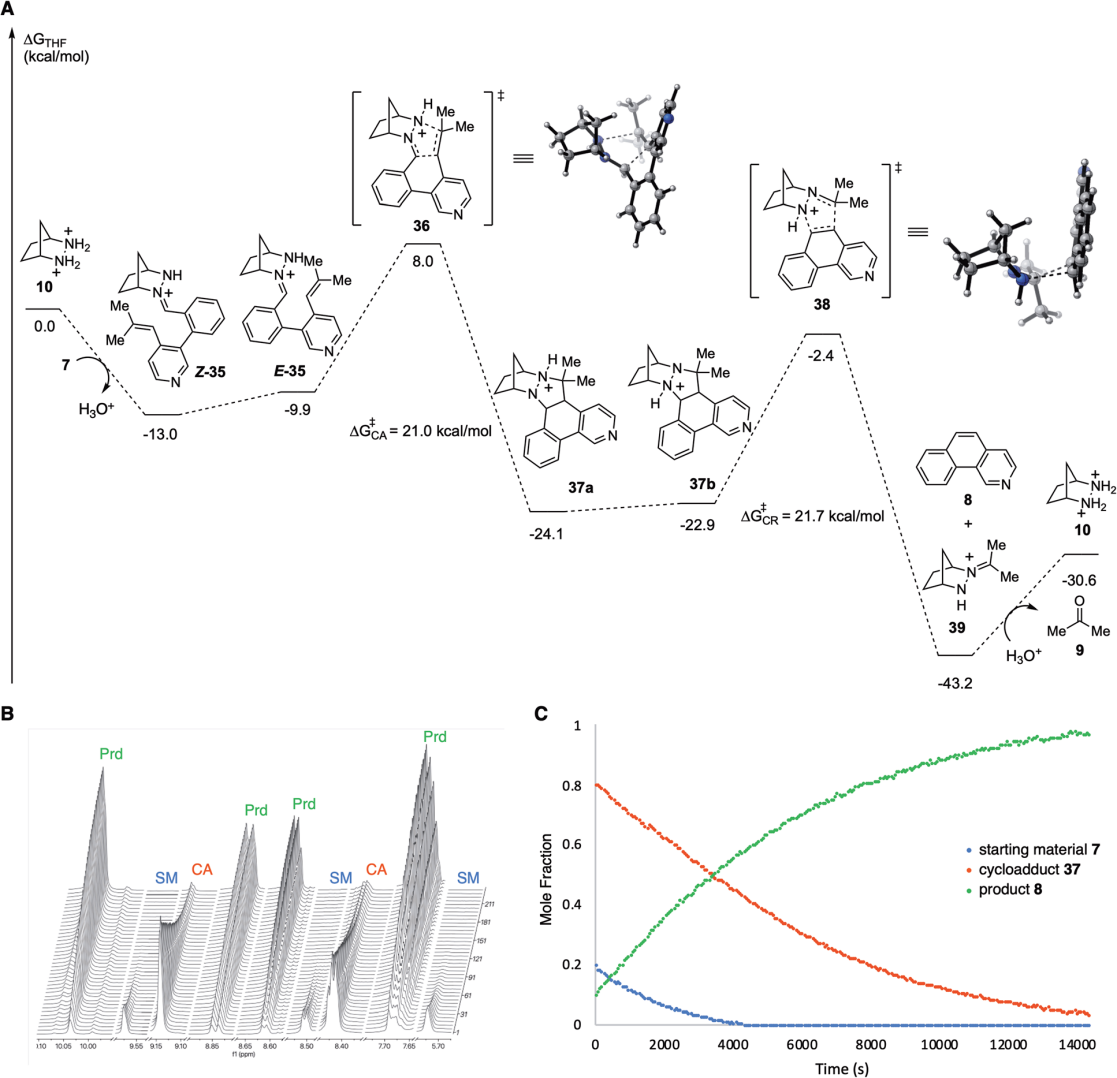
(A) Computational study of hydrazine 10-catalyzed RCCOM of biaryl aldehyde 7. Calculations were performed at the PCM(THF)-M06-2X/6-311+G(d,p)//6-31G(d) level of theory.^24,25^ All energies are given in units of kcal mol^−1^. (B) ^1^H NMR spectroscopy of the RCCOM reaction of 7 catalyzed by 10 at 60 °C in THF-*d*8 with mesitylene as internal standard for 5 hours. (C) Plot of the data showing conversion *vs.* time. SM = starting material 7; CA = cycloadduct 37; Prd = product 8.

Given the low activation energy barriers of both the cycloaddition and cycloreversion steps, we reasoned it should be possible for the reaction to proceed at a relatively low temperature. In fact, we observed 82% conversion of biaryl aldehyde 7 to cycloadduct 37 (72%) and benzoisoquinoline 8 (10%) at 40 °C over 6 hours. Attempts to isolate the cycloadduct 37 resulted in complete conversion to 8 during column chromatography. Meanwhile, at 60 °C over approximately 4 hours, 95% of the starting material 7, *via* the intermediate cycloadduct 37, was converted to benzoisoquinoline product 8 (Fig. 3B and C). The rate of consumption of the cycloadduct was consistent with first-order behavior, and upon fitting, revealed the rate constant for cycloreversion as *k*_CR_ = 2.14 × 10^−4^ s^−1^, with a half-life of 54 minutes. These observations corroborate the computational results, in particular showing that the cycloreversion step is quite facile with these types of substrates compared to other hydrazine-catalyzed COM reactions we have investigated^17^ and that cycloaddition and cycloreversion have energetically similar activation energies.

In conclusion, the development of catalytic carbonyl–olefin metathesis reactions has opened new possibilities for the rapid construction of complex molecules. The current work demonstrates this strategy as a means to rapidly access polycyclic heteroaromatics, which often require lengthy sequences that can be complicated by the presence of basic functionality. The ability of the hydrazine catalysis platform to accommodate such functional groups provides a novel approach to polycyclic heteroaromatic synthesis and greatly expands the landscape of structures accessible by RCCOM.

## Data availability

Data for this work, including experimental procedures, characterization data for all new compounds, and DFT computational details are provided in the ESI.†

## Author contributions

T. H. L. conceived of and directed the project. E. K. C., P. K. Q., Y. Z., and J. H. S. conducted the experiments and collected and analyzed the data. P. K. Q. performed all DFT calculations. T. H. L., E. K. C., and P. K. Q. wrote the manuscript.

## Conflicts of interest

There are no conflicts to declare.

## Supplementary Material

SC-013-D1SC06234D-s001
