# Supplementary material for: Polycyclic heteroaromatics via hydrazine-catalyzed ring-closing carbonyl–olefin metathesis
Source: Chem Sci. 2022 Feb 1;13(8):2418–22. doi: 10.1039/d1sc06234d (PMC8864716; doi:10.1039/d1sc06234d)
Supplement: SC-013-D1SC06234D-s001 [file SC-013-D1SC06234D-s001.pdf]

Electronic Supplementary Material (ESI) for Chemical Science.
This journal is © The Royal Society of Chemistry 2022

Supporting Information

Synthesis of Polycyclic Heteroaromatics via Hydrazine-Catalyzed
Ring-Closing Carbonyl-Olefin Metathesis

Eun Kee Cho, Phong K. Quach, Yunfei Zhang, Jae Hun Sim and Tristan H. Lambert

Table of Contents

General Information...*S3*

Experimental Details...*S4*

Synthesis of olefins S4

Synthesis of metathesis substrates S8

RCCOM reactions...S19

Screen of the olefin moiety...S30

Substrate precursor synthesis S34

Kinetic Study...*S41*

Computational Data *S43*

References..*S53*

NMR Spectra..*S54*

General Information

Commercial reagents were purchased from Fisher Chemicals, Sigma-Aldrich, Oakwood Chemical Company, TCI and Acros Organics and used without purification. All reactions were performed in the fume hood under atmospheric pressure, unless otherwise noted. Reaction products were stored in scintillation vials at ambient temperature.

Reactions were monitored by thin-layer chromatography (TLC) on EMD Silica Gel 60 F254 plates under UV light (254 nm) or visualized with I_2. Flash chromatography was performed using silica gel 60 (230-400 mesh) from SilicaFlash on a Biotage Isolera One system. Organic solutions were concentrated under reduced pressure on a Büchi rotary evaporator R-200. Proton nuclear magnetic resonance (^{1}H NMR) spectra and carbon nuclear magnetic resonance (^{13}C NMR) spectra were recorded on Bruker Magnet System 500 MHz, Varian Magnet System 400 MHz and 300 MHz. All chemical shifts are reported in parts per million (ppm) downfield from tetramethylsilane. Proton resonances are referenced to residual protium in the NMR solvent (7.26 ppm for $CHCl_3$). Carbon resonances are referenced to the carbon resonances of the NMR solvent (77.16 ppm for $CDCl_3$). Data are represented as follows: chemical shift, multiplicity (s = singlet, d = doublet, t = triplet, q = quartet, m = multiplet), coupling constants in hertz (Hz), integration. For the ^{1}H NMR yield determinations shown in Figure 1, a relaxation delay (D1) parameter of 1 s was used. Mass spectral (MS) data were obtained on Advion Mass Spectrometer equipped with an APCI (Atmospheric Pressure Chemical Ionization) module and HRMS data with direct analysis in real-time mass spectrometry (DART-MS).

Synthesis of Starting Materials

a. Synthesis of olefins

General olefination procedure for substrate precursors:

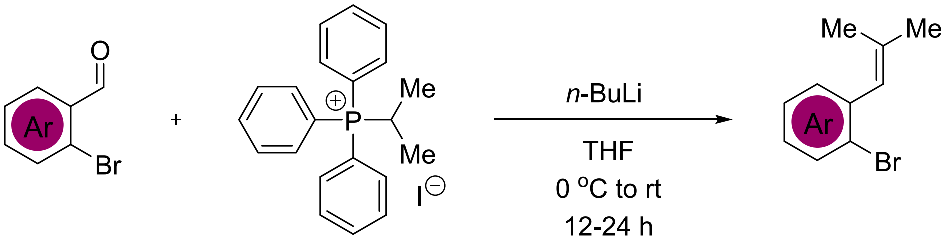

A modified version of a reported procedure was followed.[1] A flame-dried round bottom flask equipped with a magnetic stir bar was charged with isopropyltriphenylphosphonium iodide (1.1 equiv) and anhydrous THF (0.3 M). The solution was cooled to 0 °C with an ice bath before a solution of n-BuLi (1.2 equiv, 2.5 M in hexanes) was added. After stirring for 1 h at 0 °C, the aldehyde substrate (1.0 equiv) was slowly added. The reaction was allowed to warm to room temperature and stirred for 15 h (overnight) before being quenched with water. The biphasic solution was extracted with ethyl acetate three times. The combined organic phases were washed with brine, dried over $MgSO_4$, and concentrated *in vacuo*. The crude residue was purified *via* column chromatography eluting with a mixture of hexanes and ethyl acetate to furnish the desired olefin.

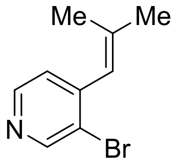

(*3-bromo-4-(2-methylprop-1-en-1-yl)pyridine*) (40) : The general olefination procedure was followed with 3-bromoisonicotinaldehyde (3.00 g, 16.1 mmol) as the substrate. The crude residue was purified by column chromatography with 10% ethyl acetate:hexanes gradient to furnish the title compound as a yellow oil (3.10 g, 14.6 mmol, 90% yield). ^{1}H NMR (500 MHz, $CDCl_3$) δ 8.65 (s, 1H), 8.39 (d, J = 4.9 Hz, 1H), 7.13 (d, J = 4.9 Hz, 1H), 6.17 (s, 1H), 1.93 (s, 3H), 1.77 (s, 3H). ^{13}C NMR (126 MHz, $CDCl_3$) δ 152.03, 147.75, 146.15, 140.98, 125.24, 122.66, 122.46, 26.66, 19.73. DART-MS m/z calcd for $C_9H_{10}BrN$ $(M + H)^+$ = 211.9997, found 212.0071.

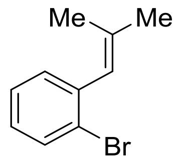

(***1-bromo-2-(2-methylprop-1-en-1-yl)benzene***) **(41):** The general olefination procedure was followed using 2-bromobenzaldehyde (3.70 g, 20.0 mmol) as the substrate. The crude residue was purified by column chromatography with 1% ethyl acetate:hexanes gradient to furnish the title compound as a colorless oil (3.88 g, 18.4 mmol, 92% yield). ^{1}H NMR (500 MHz, $CDCl_3$) δ 7.58 (dd, J = 7.9, 1.1 Hz, 1H), 7.29 – 7.24 (m, 2H), 7.08 (ddd, J = 8.6, 6.6, 2.4 Hz, 1H), 6.29 – 6.24 (m, 1H), 1.96 (d, J = 1.4 Hz, 3H), 1.77 (d, J = 1.3 Hz, 3H). The ^{1}H NMR spectrum matched with the reported values.[1] DART-MS m/z calcd for $C_{10}H_{11}Br$ $(M + H)^+$ = 211.0044, found 211.0120.

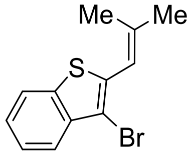

(***3-bromo-2-(2-methylprop-1-en-1-yl)benzo[b]thiophene***) **(42):** The general olefination procedure was followed using 3-bromobenzo[b]thiophene-2-carboxaldehyde (1.00 g, 4.15 mmol) as the substrate. The crude residue was purified by column chromatography with 1-5% ethyl acetate:hexanes gradient to furnish the title compound as a yellow liquid (750 mg, 2.81 mmol, 67% yield). ^{1}H NMR (400 MHz, $CDCl_3$) δ 7.77 (dd, J = 13.8, 8.0 Hz, 2H), 7.42 (t, J = 7.6 Hz, 1H), 7.34 (t, J = 7.5 Hz, 1H), 6.65 – 6.48 (m, 1H), 2.07 (s, 3H), 2.02 (s, 3H). ^{13}C NMR (126 MHz, $CDCl_3$) δ 140.34, 137.80, 137.43, 135.55, 125.06, 125.00, 122.84, 121.99, 117.67, 106.90, 27.64, 20.47. DART-MS m/z calcd for $C_{12}H_{11}BrS$ $(M + H)^+$ = 266.9765, found 266.9745.

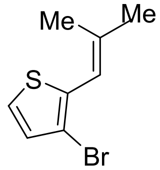

(***3-bromo-2-(2-methylprop-1-en-1-yl)thiophene***) **(43):** The general olefination procedure was followed using 3-bromothiophene-2-carboxaldehyde (3.00 g, 15.7 mmol) as the substrate. The crude residue was purified by column chromatography with 1% ethyl acetate:hexanes gradient to furnish the title compound as a brown liquid (2.99 g, 13.8 mmol, 88% yield). ^{1}H NMR (500 MHz, $CDCl_3$) δ 7.19 (d, J = 5.4 Hz, 1H), 6.97 (d, J = 5.3 Hz, 1H), 6.39 (t, J = 1.7 Hz, 1H), 2.00 – 1.95 (m, 6H). ^{13}C NMR (126 MHz, $CDCl_3$) δ 137.57, 135.31, 129.68, 123.71, 117.03, 109.64, 27.37, 20.18. DART-MS m/z calcd for C_8H_9BrS $(M + H)^+$ = 216.9608, found 216.9321.

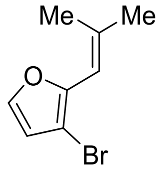

(***3-bromo-2-(2-methylprop-1-en-1-yl)furan***) **(44):** The general olefination procedure was followed using 3-bromofuran-2-carboxaldehyde (500 mg, 2.86 mmol). The crude residue was purified by column chromatography with 1-5% ethyl acetate:hexanes gradient to furnish the title compound as a brown liquid (173 mg, 0.860 mmol, 30% yield). ^{1}H NMR (400 MHz, $CDCl_3$) δ 7.32 (d, J = 2.0 Hz, 1H), 6.41 (d, J = 2.0 Hz, 1H), 6.04 (q, J = 1.5 Hz, 1H), 2.05 (s, 3H), 1.92 (s, 3H). ^{13}C NMR (126 MHz, $CDCl_3$) δ 150.51, 140.93, 137.80, 114.03, 111.00, 97.08, 27.25, 20.06. DART-MS m/z calcd for C_8H_9BrO $(M + H)^+$ = 200.9837, found 200.9736.

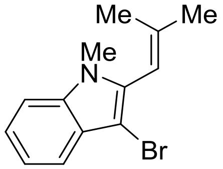

(***3-bromo-1-methyl-2-(2-methylprop-1-en-1-yl)-1H-indole***) **(45):** The general olefination procedure was followed using 3-bromo-1-methyl-1H-indole-2-carboxaldehyde (1.00 g, 4.20 mmol). The crude residue was purified by column chromatography with 25% ethyl acetate:hexanes gradient to furnish the title compound as a light-yellow liquid (1.06 g, 4.01 mmol, 96% yield). ^{1}H NMR (500 MHz, $CDCl_3$) δ 7.60 – 7.55 (m, 1H), 7.25 (qd, J = 8.0, 1.5 Hz, 2H), 7.19 (ddd, J = 8.0, 6.3, 1.8 Hz, 1H), 6.04 (p, J = 1.5 Hz, 1H), 3.61 (s, 3H), 2.02 (d, J = 1.5 Hz, 3H), 1.77 (d, J = 1.4 Hz, 3H). ^{13}C NMR (126 MHz, $CDCl_3$) δ 144.24, 136.42, 135.94, 127.23, 122.23, 120.08, 118.87, 113.32, 109.38, 90.15, 30.89, 25.82, 20.97. DART-MS m/z calcd for $C_{13}H_{14}BrN$ $(M + H)^+$ = 264.0310, found 264.0387.

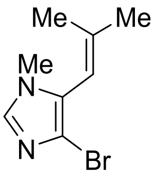

(***4-bromo-1-methyl-5-(2-methylprop-1-en-1-yl)-1H-imidazole***) **(46):** The general olefination procedure was followed using 4-bromo-1-methyl-1H-imidazole-5-carboxaldehyde (250 mg, 1.32 mmol). The crude residue was purified by column chromatography with 50% ethyl acetate:hexanes gradient to furnish the title compound as a brown liquid (177 mg, 0.823 mmol, 62% yield). ^{1}H NMR (500 MHz, $CDCl_3$) δ 7.40 (s, 1H), 5.70 (p, J = 1.5 Hz, 1H), 3.48 (s, 3H), 1.91 (d, J = 1.5 Hz, 3H), 1.68 (d, J = 1.4 Hz, 3H). ^{13}C NMR (126 MHz, $CDCl_3$) δ 144.67, 136.51, 128.04, 114.33, 109.89, 32.62, 25.65, 20.79. DART-MS m/z calcd for $C_8H_{11}BrN_2$ $(M + H)^+$ = 215.0106, found 215.0175.

b. Synthesis of metathesis substrates

General Suzuki cross-coupling procedure for metathesis substrates:

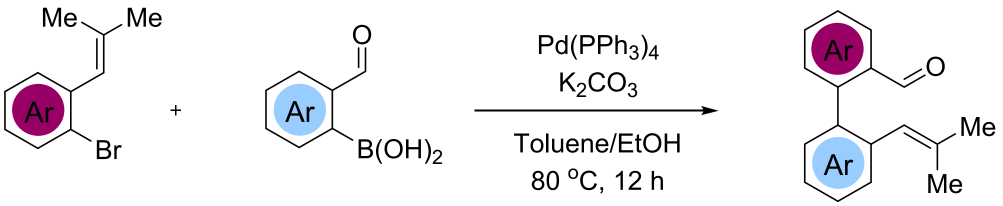

A modified version of the reported procedure was followed.[1] To a round bottom flask equipped with a magnetic stir bar were added aryl bromide (1.0 equiv), $Pd(PPh_3)_4$ (5 mol%), aryl boronic acid (1.2 equiv), and K_2CO_3 (3.2 equiv). A 1:1 mixture of toluene / ethanol solution (0.6 M) was added, and the mixture was heated to reflux for 15 h. After cooling to room temperature, the reaction mixture was diluted with ethyl acetate and washed with water. The biphasic solution was extracted with ethyl acetate three times. The combined organic phases were washed with brine, dried over $MgSO_4$, and concentrated *in vacuo*. The crude residue was purified *via* column chromatography eluting with a mixture of hexanes and ethyl acetate to furnish the desired cross-coupled product.

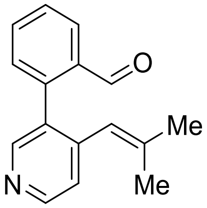

(*2-(4-(2-methylprop-1-en-1-yl)pyridin-3-yl)benzaldehyde*) (7): The general cross-coupling procedure was followed using 3-bromo-4-(2-methylprop-1-en-1-yl)pyridine (1.06 g, 5.00 mmol). The crude residue was purified by column chromatography with 50% ethyl acetate:hexanes gradient to furnish the title compound as a yellow liquid (900 mg, 3.80 mmol, 76% yield). ^{1}H NMR (500 MHz, $CDCl_3$) δ 9.59 (s, 1H), 8.59 (d, J = 4.8 Hz, 1H), 8.52 (s, 1H), 7.99 (d, J = 8.6 Hz, 1H), 7.65 (t, J = 6.9 Hz, 1H), 7.52 (t, J = 7.6 Hz, 1H), 7.30 (d, J = 7.3 Hz, 1H), 7.23 (d, J = 5.0 Hz, 1H), 5.70 (s, 1H), 1.78 (s, 3H), 1.71 (s, 3H). ^{13}C NMR (126 MHz, $CDCl_3$) δ 191.10, 150.45, 149.26, 145.86, 141.37, 140.99, 134.49, 133.88, 132.85, 131.19, 128.67, 127.60, 123.87, 122.02, 26.54, 19.55. DART-MS m/z calcd for $C_{16}H_{15}NO$ $(M + H)^+$ = 238.1154, found 238.1227.

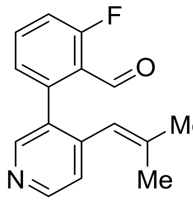

(***2-fluoro-6-(4-(2-methylprop-1-en-1-yl)pyridin-3-yl)benzaldehyde***) **(47):** The general cross-coupling procedure was followed using 3-bromo-4-(2-methylprop-1-en-1-yl)pyridine (100 mg, 0.471 mmol). The crude residue was purified by column chromatography with 50% ethyl acetate:hexanes gradient to furnish the title compound as a yellow liquid (91.3 mg, 0.358 mmol, 76% yield). ^{1}H NMR (500 MHz, $CDCl_3$) δ 9.83 (s, 1H), 8.57 (d, *J* = 5.1 Hz, 1H), 8.43 (s, 1H), 7.59 (td, *J* = 8.0, 5.4 Hz, 1H), 7.23 – 7.17 (m, 2H), 7.05 (d, *J* = 7.5 Hz, 1H), 5.69 (s, 1H), 1.74 (d, *J* = 1.4 Hz, 3H), 1.73 (d, *J* = 1.5 Hz, 3H). ^{13}C NMR (126 MHz, $CDCl_3$) δ 187.77, 187.74, 164.04, 162.99 (d, *J* = 262.2 Hz), 161.95, 149.62, 149.38 (d, *J* = 60.4 Hz), 149.14, 145.38, 141.72, 141.71, 141.22, 134.88, 134.84 (d, *J* = 10.3 Hz), 134.79, 132.95, 132.94 (d, *J* = 2.4 Hz), 132.94, 127.16, 127.13, 123.80, 123.07, 123.05 (d, *J* = 7.0 Hz), 123.02, 121.57, 121.49, 116.73, 116.64 (d, *J* = 21.5 Hz), 116.56, 26.51, 19.46. ^{19}F NMR (470 MHz, $CDCl_3$) δ -116.94 (dd, *J* = 10.6, 5.4 Hz). DART-MS m/z calcd for $C_{16}H_{14}FNO$ $(M + H)^+$ = 256.1059, found 256.1132.

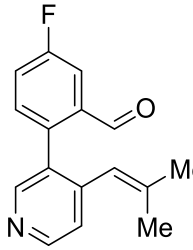

(***5-fluoro-2-(4-(2-methylprop-1-en-1-yl)pyridin-3-yl)benzaldehyde***) **(48):** The general cross-coupling procedure was followed using 3-bromo-4-(2-methylprop-1-en-1-yl)pyridine (50.0 mg, 0.236 mmol). The crude residue was purified by column chromatography with 50% ethyl acetate:hexanes gradient to furnish the title compound as a yellow liquid (28.5 mg, 0.112 mmol, 47% yield). ^{1}H NMR (400 MHz, $CDCl_3$) δ 9.52 (d, *J* = 3.1 Hz, 1H), 8.62 (d, *J* = 5.2 Hz, 1H), 8.52 (s, 1H), 7.68 (dd, *J* = 8.9, 2.8 Hz, 1H), 7.38 (td, *J* = 8.1, 2.6 Hz, 1H), 7.32 (dd, *J* = 8.5, 5.3 Hz, 1H), 7.29 – 7.23 (m, 1H), 5.69 (s, 1H), 1.79 (s, 3H), 1.74 (s, 3H). ^{13}C NMR (101 MHz, $CDCl_3$) δ 189.71, 163.91, 162.66 (d, *J* = 250.3 Hz), 161.42, 150.42, 149.39, 145.94, 141.70, 136.84, 136.82(d, *J* = 2.7 Hz), 136.81, 136.12 (d, *J* = 6.2 Hz), 136.15, 136.08, 133.07, 133.04 (d, *J* = 7.4 Hz), 133.00, 131.76, 123.88, 121.74, 121.11, 121.00 (d, *J* = 21.9 Hz), 120.89, 113.94, 113.83 (d, *J* = 22.5 Hz), 113.72, 26.45, 19.45. ^{19}F NMR (376 MHz, $CDCl_3$) δ -111.77 (dq, *J* = 8.1, 4.1 Hz). DART-MS m/z calcd for $C_{16}H_{14}FNO$ $(M + H)^+$ = 256.1059, found 256.1131.

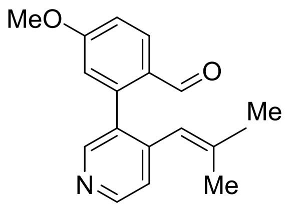

(***4-methoxy-2-(4-(2-methylprop-1-en-1-yl)pyridin-3-yl)benzaldehyde***) **(49)**: The general cross-coupling procedure was followed using 3-bromo-4-(2-methylprop-1-en-1-yl)pyridine (100 mg, 0.471 mmol). The crude residue was purified by column chromatography with 35% ethyl acetate:hexanes gradient to furnish the title compound as a yellow liquid (62.0 mg, 0.232 mmol, 49% yield). ^{1}H NMR (500 MHz, $CDCl_3$) δ 9.45 (s, 1H), 8.60 (d, *J* = 5.1 Hz, 1H), 8.52 (s, 1H), 7.99 (d, *J* = 8.7 Hz, 1H), 7.24 (d, *J* = 5.1 Hz, 1H), 7.03 (dd, *J* = 8.6, 2.6 Hz, 1H), 6.75 (d, *J* = 2.6 Hz, 1H), 5.76 – 5.71 (m, 1H), 3.90 (s, 3H), 1.81 (d, *J* = 1.5 Hz, 3H), 1.74 (d, *J* = 1.5 Hz, 3H). ^{13}C NMR (126 MHz, $CDCl_3$) δ 189.85, 163.75, 150.15, 149.17, 145.65, 143.34, 141.16, 132.78, 129.94, 127.99, 123.71, 121.85, 115.88, 114.28, 55.69, 26.56, 19.52. DART-MS m/z calcd for $C_{17}H_{17}NO_2$ $(M + H)^+$ = 268.1259, found 268.1176.

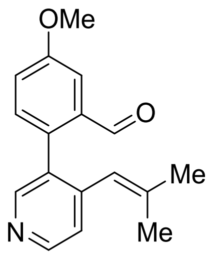

(***5-methoxy-2-(4-(2-methylprop-1-en-1-yl)pyridin-3-yl)benzaldehyde***) **(50):** The general cross-coupling procedure was followed using 3-bromo-4-(2-methylprop-1-en-1-yl)pyridine (50.0 mg, 0.236 mmol). The crude residue was purified by column chromatography with 40% ethyl acetate:hexanes gradient to furnish the title compound as a yellow liquid (38.5 mg, 0.144 mmol, 61% yield). ^{1}H NMR (400 MHz, $CDCl_3$) δ 9.57 (s, 1H), 8.62 (d, *J* = 5.1 Hz, 1H), 8.55 (s, 1H), 7.53 (d, *J* = 2.3 Hz, 1H), 7.29 (d, *J* = 13.8 Hz, 1H), 7.25 (d, *J* = 5.8 Hz, 1H), 5.75 (s, 1H), 3.95 (s, 3H), 1.83 (d, *J* = 1.4 Hz, 3H), 1.77 (d, *J* = 1.5 Hz, 3H). ^{13}C NMR (101 MHz, $CDCl_3$) δ 190.94, 159.71, 150.68, 148.92, 145.98, 141.06, 135.33, 133.65, 132.47, 132.38, 123.78, 122.13, 121.23, 110.19, 55.61, 26.48, 19.67. DART-MS m/z calcd for $C_{17}H_{17}NO_2$ $(M + H)^+$ = 268.1259, found 268.1334.

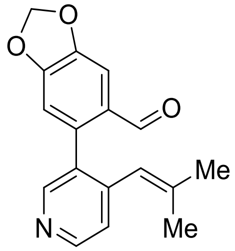

(***6-(4-(2-methylprop-1-en-1-yl)pyridin-3-yl)benzo[d][1,3]dioxole-5-carboxaldehyde***) **(51):** The general cross-coupling procedure was followed using 3-bromo-4-(2-methylprop-1-en-1-yl)pyridine (100 mg, 0.471 mmol). The crude residue was purified by column chromatography with 40% ethyl acetate:hexanes gradient to furnish the title compound as a yellow liquid (122 mg, 0.435 mmol, 92% yield). ^{1}H NMR (500 MHz, $CDCl_3$) δ 9.35 (s, 1H), 8.56 (d, J = 5.2 Hz, 1H), 8.46 (s, 1H), 7.42 (s, 1H), 7.24 (d, J = 5.2 Hz, 1H), 6.68 (s, 1H), 6.16 – 6.04 (m, 2H), 5.74 (t, J = 1.7 Hz, 1H), 1.80 (d, J = 1.4 Hz, 3H), 1.74 (d, J = 1.5 Hz, 3H). ^{13}C NMR (126 MHz, $CDCl_3$) δ 189.34, 152.32, 150.09, 148.72, 148.36, 146.26, 141.45, 137.89, 132.61, 129.52, 123.87, 121.77, 110.58, 106.43, 102.32, 26.62, 19.54. DART-MS m/z calcd for $C_{17}H_{15}NO_3$ $(M + H)^+$ = 282.1052, found 282.1489.

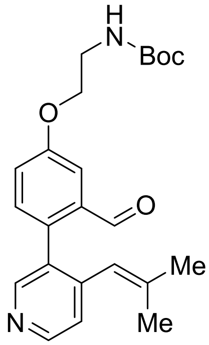

(***tert-butyl (2-(3-formyl-4-(4-(2-methylprop-1-en-1-yl)pyridin-3-yl)phenoxy)ethyl)carbamate***) **(52):** The general cross-coupling procedure was followed using 3-bromo-4-(2-methylprop-1-en-1-yl)pyridine (50.0 mg, 0.236 mmol). The crude residue was purified by column chromatography with 50% ethyl acetate:hexanes gradient to furnish the title compound as a yellow liquid (58.3 mg, 0.147 mmol, 62% yield). ^{1}H NMR (400 MHz, $CDCl_3$) δ 9.50 (s, 1H), 8.56 (d, J = 5.1 Hz, 1H), 8.48 (s, 1H), 7.46 (d, J = 2.5 Hz, 1H), 7.24 – 7.18 (m, 3H), 5.69 (s, 1H), 5.18 – 4.99 (m, 1H), 4.13 – 4.09 (m, 2H), 3.56 (q, J = 5.5 Hz, 2H), 1.79 – 1.76 (m, 3H), 1.73 – 1.69 (m, 3H), 1.44 (s, 9H). ^{13}C NMR (101 MHz, $CDCl_3$) δ 190.71, 158.71, 155.87, 150.56, 148.90, 146.00, 141.14, 135.37, 133.85, 132.45, 132.39, 123.79, 122.06, 121.14, 111.32, 77.27, 67.58, 60.37, 28.40, 26.46, 19.43. DART-MS m/z calcd for $C_{23}H_{28}N_2O_4$ $(M + H)^+$ = 397.2049, found 397.2129.

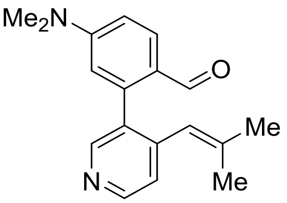

(***4-(dimethylamino)-2-(4-(2-methylprop-1-en-1-yl)pyridin-3-yl)benzaldehyde***) **(53):** The general cross-coupling procedure was followed using 3-bromo-4-(2-methylprop-1-en-1-yl)pyridine (50.0 mg, 0.236 mmol). The crude residue was purified by column chromatography with 50% ethyl acetate:hexanes gradient to furnish the title compound as a yellow liquid (34.7 mg, 0.124 mmol, 53% yield). ^{1}H NMR (400 MHz, $CDCl_3$) δ 9.31 (s, 1H), 8.56 (d, *J* = 5.1 Hz, 1H), 8.50 (s, 1H), 7.90 (d, *J* = 8.9 Hz, 1H), 7.22 (d, *J* = 5.1 Hz, 1H), 6.73 (dd, *J* = 9.0, 2.6 Hz, 1H), 6.38 (d, *J* = 2.6 Hz, 1H), 5.79 (s, 1H), 3.08 (s, 6H), 1.82 (d, 3H), 1.73 (d, *J* = 1.5 Hz, 3H). ^{13}C NMR (101 MHz, $CDCl_3$) δ 189.52, 153.50, 150.25, 148.79, 145.56, 143.24, 140.44, 133.94, 129.63, 123.51, 123.37, 122.12, 112.51, 111.10, 40.09, 26.63, 19.57. DART-MS m/z calcd for $C_{18}H_{20}N_2O(M + H)^+$ = 281.1576, found 281.1652.

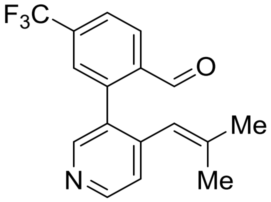

(***2-(4-(2-methylprop-1-en-1-yl)pyridin-3-yl)-4-(trifluoromethyl)benzaldehyde***) **(54):** A 15 mL round bottom flask equipped with a stir bar was charged with (2-formyl-5-(trifluoromethyl)phenyl)boronic acid (123.3 mg, 0.566 mmol) anhydrous potassium carbonate (217 mg, 1.57 mmol), 3-bromo-4-(2-methylprop-1-en-1-yl)pyridine (100 mg, 0.471 mmol), $Pd(PPh_3)_4$ (27.2 mg, 24.0 µmol), and THF (1.5 mL). The mixture was stirred vigorously at room temperature for 20 minutes and then at 60 °C for 24 hours. The crude residue was purified by column chromatography with 25% ethyl acetate:hexanes gradient to furnish the title compound as a light-yellow oil (6.60 mg, 22.0 µmol, 4.6 % yield). While the NMR of the product was satisfactory, there was a coeluting impurity that could not be separated. The product was used as is in the next step. ^{1}H NMR (500 MHz, $CDCl_3$) δ 9.64 (s, 1H), 8.69 (d, *J* = 5.0 Hz, 1H), 8.60 (s, 1H), 8.14 (d, *J* = 8.1 Hz, 1H), 7.82 (dd, *J* = 8.1, 1.7 Hz, 1H), 7.63 (d, *J* = 1.8 Hz, 1H), 7.30 (d, *J* = 5.1 Hz, 1H), 5.70 (s, 1H), 1.82 (d, *J* = 1.4 Hz, 3H), 1.76 (d, *J* = 1.4 Hz, 3H). DART-MS m/z calcd for $C_{17}H_{14}F_3NO$ $(M + H)^+$ = 306.1027, found 306.1105.

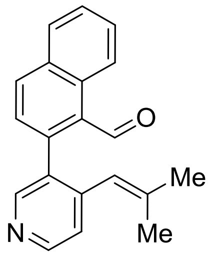

(***2-(4-(2-methylprop-1-en-1-yl)pyridin-3-yl)-1-naphthaldehyde***) **(55):** The general cross-coupling procedure was followed using 3-bromo-4-(2-methylprop-1-en-1-yl)pyridine (59.0 mg, 0.278 mmol). The crude residue was purified by column chromatography with 10% methanol:dichloromethane gradient to furnish the title compound as a light-yellow oil (31.6 mg, 0.110 mmol, 39.6 % yield). While the NMR of the product was satisfactory, there was a coeluting impurity that could not be separated. The product was used as is in the next step. ^{1}H NMR (500 MHz, $CDCl_3$) δ 9.73 (d, *J* = 0.9 Hz, 1H), 8.72 (d, *J* = 5.2 Hz, 1H), 8.52 (s, 1H), 8.07 (d, *J* = 8.6 Hz, 1H), 8.00 – 7.93 (m, 2H), 7.64 (ddd, *J* = 8.1, 4.7, 3.2 Hz, 1H), 7.49 – 7.45 (m, 2H), 7.40 (d, *J* = 5.2 Hz, 1H), 5.56 (q, *J* = 1.6 Hz, 1H), 1.84 (d, *J* = 1.4 Hz, 3H), 1.60 (d, *J* = 1.5 Hz, 3H). DART-MS m/z calcd for $C_{20}H_{17}NO$ $(M + H)^+$ = 288.1310, found 288.1389.

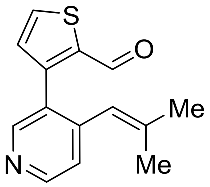

(***3-(4-(2-methylprop-1-en-1-yl)pyridin-3-yl)thiophene-2-carboxaldehyde***) **(56):** The general cross-coupling procedure was followed using 3-bromo-4-(2-methylprop-1-en-1-yl)pyridine (382 mg, 1.80 mmol). The crude residue was purified by column chromatography with 20% ethyl acetate:hexanes gradient to furnish the title compound as a yellow liquid (280 mg, 1.15 mmol, 64% yield). ^{1}H NMR (400 MHz, $CDCl_3$) δ 9.54 (s, 1H), 8.59 (d, *J* = 5.1 Hz, 1H), 8.54 (s, 1H), 7.77 (d, *J* = 4.8 Hz, 1H), 7.29 (d, *J* = 5.2 Hz, 1H), 7.10 (d, *J* = 5.0 Hz, 1H), 5.92 (s, 1H), 1.81 (d, *J* = 4.2 Hz, 6H). ^{13}C NMR (101 MHz, $CDCl_3$) δ 183.39, 150.36, 149.29, 146.26, 146.11, 141.58, 140.03, 134.08, 131.36, 129.19, 124.57, 121.88, 26.75, 19.78. DART-MS m/z calcd for $C_{14}H_{13}NOS$ $(M + H)^+$ = 244.0718, found 244.0788.

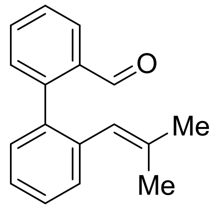

(***2'-(2-methylprop-1-en-1-yl)-[1,1'-biphenyl]-2-carboxaldehyde***) **(57):** The general cross-coupling procedure was followed using 1-bromo-2-(2-methylprop-1-en-1-yl)benzene (1.06 g, 5.00 mmol). The crude residue was purified by column chromatography with 5% ethyl acetate:hexanes gradient to furnish the title compound as a yellow liquid (900 mg, 3.79 mmol, 76% yield). ^{1}H NMR (500 MHz, $CDCl_3$) δ 9.54 (s, 1H), 7.92 (d, J = 7.7 Hz, 1H), 7.58 – 7.54 (m, 1H), 7.41 (t, J = 7.5 Hz, 1H), 7.36 – 7.32 (m, 1H), 7.25 (d, J = 7.0 Hz, 4H), 5.70 (s, 1H), 1.69 (s, 3H), 1.62 (s, 3H). ^{13}C NMR (126 MHz, $CDCl_3$) δ 192.13, 145.24, 138.29, 137.28, 137.18, 134.26, 133.54, 131.08, 130.35, 129.88, 128.03, 127.80, 126.77, 126.61, 124.44, 26.19, 19.29. DART-MS m/z calcd for $C_{17}H_{16}O$ $(M + H)^+$ = 237.1201, found 237.1276.

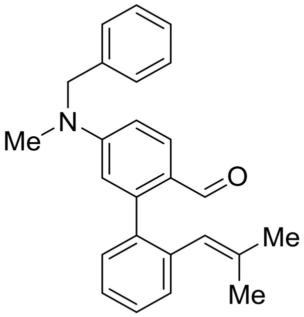

(***5-(benzyl(methyl)amino)-2'-(2-methylprop-1-en-1-yl)-[1,1'-biphenyl]-2-carboxaldehyde***) **(58):** The general cross-coupling procedure was followed using 1-bromo-2-(2-methylprop-1-en-1-yl)benzene (50.0 mg, 0.237 mmol). The crude residue was purified by column chromatography with 25% ethyl acetate:hexanes gradient to furnish the title compound as a yellow liquid (31.6 mg, 89.0 µmol, 38% yield). ^{1}H NMR (500 MHz, $CDCl_3$) δ 9.36 (s, 1H), 7.90 (d, J = 8.9 Hz, 1H), 7.36 – 7.32 (m, 3H), 7.30 – 7.26 (m, 4H), 7.23 – 7.17 (m, 2H), 6.78 (dd, J = 9.0, 2.7 Hz, 1H), 6.50 (d, J = 2.6 Hz, 1H), 5.84 (d, J = 2.5 Hz, 1H), 4.65 (dd, 2H), 3.13 (s, 3H), 1.75 (d, J = 1.4 Hz, 3H), 1.71 (d, J = 1.5 Hz, 3H). 13C NMR (126 MHz, CDCl3) δ 190.49, 153.02, 147.60, 138.13, 137.99, 137.43, 136.21, 130.16, 129.61, 129.04, 128.83, 127.54, 127.33, 126.49, 126.13, 124.44, 123.76, 112.75, 110.85, 55.86, 38.65, 26.22, 19.27. DART-MS m/z calcd for $C_{25}H_{25}NO$ $(M + H)^+$ = 356.1936, found 356.2013.

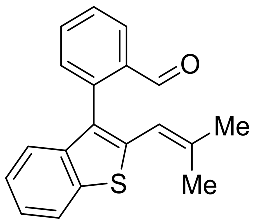

(***2-(2-(2-methylprop-1-en-1-yl)benzo[b]thiophen-3-yl)benzaldehyde***) **(59):** The general cross-coupling procedure was followed using 3-bromo-2-(2-methylprop-1-en-1-yl)benzo[b]thiophene (300 mg, 1.12 mmol). The crude residue was purified by column chromatography with 5% ethyl acetate:hexanes gradient to furnish the title compound as a pale-yellow oil (317 mg, 1.08 mmol, 96% yield). ^{1}H NMR (400 MHz, $CDCl_3$) δ 9.71 (d, J = 0.9 Hz, 1H), 8.12 (dd, J = 7.8, 1.5 Hz, 1H), 7.86 (dt, J = 7.6, 1.2 Hz, 1H), 7.72 (td, J = 7.5, 1.5 Hz, 1H), 7.58 (tt, J = 7.5, 1.1 Hz, 1H), 7.43 (dd, J = 7.6, 1.3 Hz, 1H), 7.37 – 7.29 (m, 3H), 6.10 (p, J = 1.4 Hz, 1H), 2.03 (d, J = 1.3 Hz, 3H), 1.83 (d, J = 1.5 Hz, 3H). ^{13}C NMR (101 MHz, $CDCl_3$) δ 192.19, 140.09, 139.89, 139.47, 139.40, 138.61, 135.20, 134.05, 132.00, 129.45, 128.39, 127.46, 124.80, 124.60, 122.45, 122.02, 117.32, 27.58, 20.41. DART-MS m/z calcd for $C_{19}H_{16}OS$ $(M + H)^+$ = 293.0922, found 293.0954.

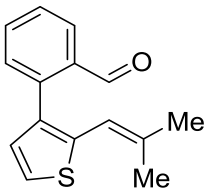

(***2-(2-(2-methylprop-1-en-1-yl)thiophen-3-yl)benzaldehyde***) **(60):** The general cross-coupling procedure was followed using 3-bromo-2-(2-methylprop-1-en-1-yl)thiophene (100 mg, 0.461 mmol). The crude residue was purified by column chromatography with 1-5% ethyl acetate:hexanes gradient to furnish the title compound as a yellow liquid (70.0 mg, 0.289 mmol, 63% yield). ^{1}H NMR (400 MHz, $CDCl_3$) δ 9.81 (s, 1H), 8.01 (dd, J = 7.9, 1.5 Hz, 1H), 7.63 (td, J = 7.5, 1.5 Hz, 1H), 7.48 (t, J = 7.5 Hz, 1H), 7.37 (d, J = 7.7 Hz, 1H), 7.30 (d, J = 5.1 Hz, 1H), 7.03 (d, J = 5.2 Hz, 1H), 6.03 – 5.95 (m, 1H), 1.91 (d, 3H), 1.78 (d, J = 1.5 Hz, 3H). ^{13}C NMR (126 MHz, $CDCl_3$) δ 192.46, 140.72, 138.72, 137.77, 134.70, 134.39, 133.62, 131.34, 129.57, 127.81, 127.21, 123.46, 116.70, 27.16, 20.17. DART-MS m/z calcd for $C_{15}H_{14}OS$ $(M + H)^+$ = 243.0765, found 243.0477.

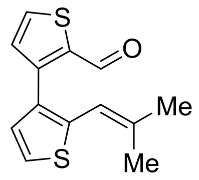

(***2'-(2-methylprop-1-en-1-yl)-[3,3'-bithiophene]-2-carboxaldehyde***) **(61):** The general cross-coupling procedure was followed using 3-bromo-2-(2-methylprop-1-en-1-yl)thiophene (100 mg, 0.461 mmol). The crude residue was purified by column chromatography with 10% ethyl acetate:hexanes gradient to furnish the title compound as a yellow liquid (39.0 mg, 0.161 mmol, 35% yield). ^{1}H NMR (400 MHz, $CDCl_3$) δ 9.72 (d, J = 1.3 Hz, 1H), 7.73 (dd, J = 4.9, 1.3 Hz, 1H), 7.30 (d, J = 5.2 Hz, 1H), 7.13 (d, J = 4.9 Hz, 1H), 7.05 (d, J = 5.2 Hz, 1H), 6.19 (h, J = 1.5 Hz, 1H), 1.93 (d, J = 1.2 Hz, 3H), 1.85 (d, J = 1.5 Hz, 3H). ^{13}C NMR (101 MHz, $CDCl_3$) δ 184.40, 145.83, 139.25, 138.91, 138.09, 133.75, 131.13, 130.94, 129.19, 123.76, 116.82, 27.18, 20.16. DART-MS m/z calcd for $C_{13}H_{12}OS_2$ $(M + H)^+$ = 249.0330, found 249.0204.

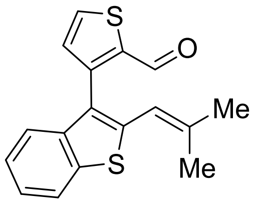

(***3-(2-(2-methylprop-1-en-1-yl)benzo[b]thiophen-3-yl)thiophene-2-carboxaldehyde***) **(62):** The general cross-coupling procedure was followed using 3-bromo-2-(2-methylprop-1-en-1-yl)benzo[b]thiophene (300 mg, 1.12 mmol). The crude residue was purified by column chromatography with 1-5% ethyl acetate:hexanes gradient to furnish the title compound as a yellow oil (96.0 mg, 0.328 mmol, 30% yield). ^{1}H NMR (400 MHz, $CDCl_3$) δ 9.58 (d, J = 1.3 Hz, 1H), 7.87 – 7.82 (m, 2H), 7.51 – 7.45 (m, 1H), 7.38 – 7.31 (m, 2H), 7.21 (d, J = 5.0 Hz, 1H), 6.23 (h, J = 1.4 Hz, 1H), 2.02 (d, J = 1.3 Hz, 3H), 1.88 (d, J = 1.4 Hz, 3H). ^{13}C NMR (126 MHz, $CDCl_3$) δ 184.25, 144.37, 140.41, 140.28, 140.24, 139.33, 138.59, 134.20, 131.24, 125.73, 124.88, 124.71, 122.31, 122.04, 117.26, 27.55, 20.36. DART-MS m/z calcd for $C_{17}H_{14}OS_2$ $(M + H)^+$ = 299.0486, found 299.0622.

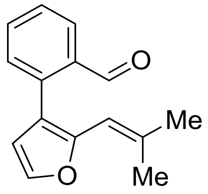

(***2-(2-(2-methylprop-1-en-1-yl)furan-3-yl)benzaldehyde***) **(63):** The general cross-coupling procedure was followed using 3-bromo-2-(2-methylprop-1-en-1-yl)furan (500 mg, 2.86 mmol). The crude residue was purified by column chromatography with 20% ethyl acetate:hexanes gradient to furnish the title compound as a tan liquid (173 mg, 0.860 mmol, 30% yield). ^{1}H NMR (500 MHz, $CDCl_3$) δ 9.90 (d, *J* = 0.9 Hz, 1H), 7.89 (dd, *J* = 7.8, 1.5 Hz, 1H), 7.49 (td, *J* = 7.5, 1.5 Hz, 1H), 7.36 (d, *J* = 1.9 Hz, 1H), 7.33 (tt, *J* = 7.6, 1.2 Hz, 1H), 7.27 (dd, *J* = 7.8, 1.3 Hz, 1H), 6.37 (d, *J* = 1.9 Hz, 1H), 5.69 (p, *J* = 1.4 Hz, 1H), 1.89 (d, *J* = 1.3 Hz, 3H), 1.70 (d, *J* = 1.4 Hz, 3H). ^{13}C NMR (126 MHz, $CDCl_3$) δ 192.46, 151.35, 140.93, 138.44, 137.96, 134.17, 133.73, 131.37, 127.65, 127.49, 118.10, 113.55, 111.56, 27.20, 20.29. DART-MS m/z calcd for $C_{15}H_{14}O_2$ $(M + H)^+$ = 227.0994, found 227.1069.

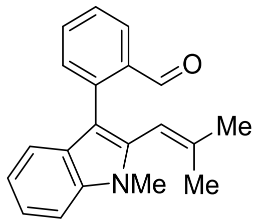

(***2-(1-methyl-2-(2-methylprop-1-en-1-yl)-1H-indol-3-yl)benzaldehyde***) **(64)**: The general cross-coupling procedure was followed using 3-bromo-1-methyl-2-(2-methylprop-1-en-1-yl)-1H-indole (400 mg, 1.51 mmol). The crude residue was purified by column chromatography with 10% ethyl acetate:hexanes gradient to furnish the title compound as a yellow liquid (109 mg, 0.376 mmol, 25% yield). ^{1}H NMR (500 MHz, $CDCl_3$) δ 9.83 (d, *J* = 0.9 Hz, 1H), 8.00 (dd, *J* = 7.9, 1.5 Hz, 1H), 7.64 (td, *J* = 7.5, 1.5 Hz, 1H), 7.58 (dq, *J* = 7.7, 1.2 Hz, 2H), 7.44 – 7.37 (m, 2H), 7.29 (ddd, *J* = 8.2, 7.0, 1.2 Hz, 1H), 7.17 (ddd, *J* = 8.0, 7.0, 1.1 Hz, 1H), 6.04 (p, *J* = 1.5 Hz, 1H), 3.73 (s, 3H), 1.79 (d, *J* = 1.5 Hz, 3H), 1.24 (d, *J* = 1.4 Hz, 3H). ^{13}C NMR (126 MHz, $CDCl_3$) δ 192.90, 143.90, 139.91, 137.62, 136.98, 133.86, 133.68, 131.72, 127.60, 127.42, 126.42, 122.12, 120.39, 118.80, 113.26, 110.39, 109.38, 30.43, 25.60, 20.14. APCI-MS m/z calcd for $C_{20}H_{19}NO$ $(M + H)^+$ = 290.1, found 290.1.

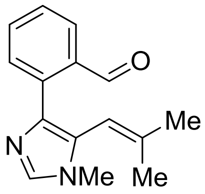

(***2-(1-methyl-5-(2-methylprop-1-en-1-yl)-1H-imidazol-4-yl)benzaldehyde***) **(65):** A modified version of a reported procedure was followed.[2] To a round bottom flask equipped with a magnetic stir bar were added 4-bromo-1-methyl-5-(2-methylprop-1-en-1-yl)-1H-imidazole (70.0 mg, 0.325 mmol, 1.0 equiv), $Pd(PPh_3)_4$ (6 mol%), 2-formyl phenylboronic acid (61.0 mg, 0.407 mmol, 1.2 equiv) and $K_3PO_4{\cdot}H_2O$ (150 mg, 0.651 mmol, 2.0 equiv). A mixture of 1,4-dioxane (1.3 mL) and H_2O (0.32 mL) was added. The reaction mixture was heated to reflux for 15 h in a 100 °C bath. After the reaction was allowed to cool to room temperature, it was diluted with ethyl acetate and washed with water. The biphasic solution was extracted with ethyl acetate three times. The combined organic phases were washed with brine, dried over $MgSO_4$, and concentrated *in vacuo*. The crude residue was purified by column chromatography with 75% ethyl acetate:hexanes gradient to furnish the title compound as a faint yellow liquid (30.3 mg, 0.126 mmol, 39% yield). ^{1}H NMR (500 MHz, $CDCl_3$) δ 10.05 (s, 1H), 7.92 – 7.89 (m, 1H), 7.60 – 7.54 (m, 3H), 7.38 – 7.34 (m, 1H), 5.90 – 5.87 (m, 1H), 3.57 (s, 3H), 1.79 (d, J = 1.4 Hz, 3H), 1.19 (s, 3H). ^{13}C NMR (126 MHz, $CDCl_3$) δ 192.73, 137.71, 135.48, 133.42, 132.14, 132.07, 130.13, 128.56, 128.46, 127.18, 127.09, 110.86, 31.92, 25.54, 19.92. DART-MS m/z calcd for $C_{15}H_{16}N_2O$ $(M + H)^+$ = 241.1263, found 241.1340.

c. RCCOM reactions

General ring-closing carbonyl-olefin metathesis procedure for polycyclic heteroaromatics.

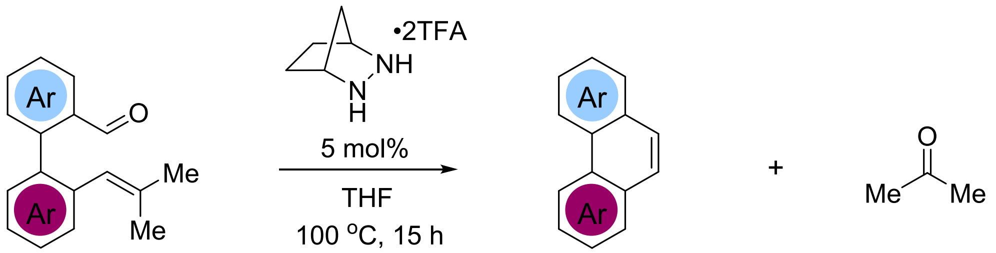

A sealed vial equipped with a stir bar was charged with the metathesis substrate (1.0 equiv), catalyst (5 mol%), and THF (0.5 M). The solution was stirred for 15 h at 100 °C. After completion, the solvent was removed *in vacuo* and the crude residue was purified via column chromatography eluting with a mixture of hexanes and ethyl acetate to furnish the desired compound.

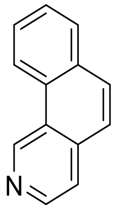

(*benzo[h]isoquinoline*) (8): The general metathesis procedure was followed using 2-(4-(2-methylprop-1-en-1-yl)pyridin-3-yl)benzaldehyde (47.5 mg, 0.200 mmol). The crude residue was purified by column chromatography with 50% ethyl acetate:hexanes gradient to furnish the title compound as a brown liquid (30.9 mg, 0.172 mmol, 86% yield). ^{1}H NMR (500 MHz, $CDCl_3$) δ 10.06 (s, 1H), 8.80 (d, J = 8.2 Hz, 1H), 8.71 (d, J = 5.3 Hz, 1H), 7.95 (dd, J = 8.7, 6.3 Hz, 2H), 7.77 – 7.66 (m, 4H). ^{13}C NMR (126 MHz, $CDCl_3$) δ 146.61, 144.80, 136.03, 132.25, 131.91, 129.36, 128.98, 127.98, 127.56, 125.13, 124.83, 122.03, 121.31, 13.76. DART-MS m/z calcd for $C_{13}H_9N$ $(M + H)^+$ = 180.0735, found 180.0808.

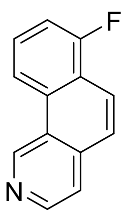

(***7-fluorobenzo[h]isoquinoline***) **(12):** The general metathesis procedure was followed using 2-fluoro-6-(4-(2-methylprop-1-en-1-yl)pyridin-3-yl)benzaldehyde (51.3 mg, 0.201 mmol). The product was purified by column chromatography with 35% ethyl acetate:hexanes gradient to furnish the title compound as a brown solid (30.0 mg, 0.152 mmol, 76% yield). ^{1}H NMR (500 MHz, $CDCl_3$) δ 9.99 (s, 1H), 8.73 (d, J = 5.4 Hz, 1H), 8.52 (d, J = 8.4 Hz, 1H), 8.19 (d, J = 9.0 Hz, 1H), 7.75 – 7.68 (m, 2H), 7.65 (td, J = 8.1, 5.7 Hz, 1H), 7.33 (dd, J = 10.0, 7.9 Hz, 1H). ^{13}C NMR (126 MHz, $CDCl_3$) δ 160.28, 159.28 (d, J = 251.9 Hz), 158.28, 147.09, 145.55, 135.95, 131.08 (d, J = 3.9 Hz), 131.09, 131.06, 128.14, 128.10 (d, J = 8.7 Hz), 128.07, 125.33, 125.32 (d, J = 2.2 Hz), 125.31, 124.42 (d, J = 2.6 Hz), 124.43, 124.41, 123.47, 123.44 (d, J = 6.8 Hz), 123.42, 121.83, 121.77 (d, J = 15.3 Hz), 121.71, 121.32, 117.80, 117.78 (d, J = 4.0 Hz), 117.76, 112.12, 112.04 (d, J = 20.3 Hz), 111.96. ^{19}F NMR (470 MHz, $CDCl_3$) δ -121.32 (dd, J = 10.0, 5.7 Hz). DART-MS m/z calcd for $C_{13}H_8FN$ $(M + H)^+$ = 198.0641, found 198.0714.

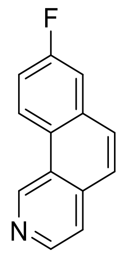

(***8-fluorobenzo[h]isoquinoline***) **(13):** The general metathesis procedure was followed using 5-fluoro-2-(4-(2-methylprop-1-en-1-yl)pyridin-3-yl)benzaldehyde (28.5 mg, 0.112 mmol). The crude residue was purified by column chromatography with 25% ethyl acetate:hexanes gradient to furnish the title compound as a pale yellow solid (17.3 mg, 88.0 μmmol, 79% yield). ^{1}H NMR (500 MHz, $CDCl_3$) δ 9.98 (s, 1H), 8.83 – 8.59 (m, 2H), 7.87 (d, J = 8.9 Hz, 1H), 7.74 – 7.68 (m, 2H), 7.56 (dd, J = 9.2, 2.7 Hz, 1H), 7.48 (td, J = 8.6, 2.7 Hz, 1H). ^{13}C NMR (101 MHz, $CDCl_3$) δ 162.92, 161.69 (d, J = 248.1 Hz), 160.46, 146.48, 144.90, 135.33, 133.74, 133.70 (d, J = 8.7 Hz), 133.66, 130.92, 130.90 (d, J = 3.7 Hz), 130.88, 126.20, 125.91, 124.42, 124.33, 121.40, 117.04, 116.93 (d, J = 23.8 Hz), 116.81, 113.28, 113.18 (d, J = 20.7 Hz), 113.08. ^{19}F NMR (376 MHz, $CDCl_3$) δ -113.11. DART-MS m/z calcd for $C_{13}H_8FN$ $(M + H)^+$ = 198.0641, found 198.0715.

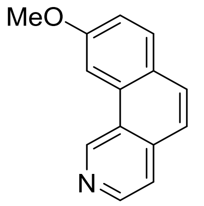

(***9-methoxybenzo[h]isoquinoline***) **(14):** The general metathesis procedure was followed using 4-methoxy-2-(4-(2-methylprop-1-en-1-yl)pyridin-3-yl)benzaldehyde (62.0 mg, 0.232 mmol). The crude residue was purified by column chromatography with 30% ethyl acetate:hexanes gradient to furnish the title compound as a dark yellow solid (36.0 mg, 0.172 mmol, 74% yield). ^{1}H NMR (500 MHz, $CDCl_3$) δ 9.98 (s, 1H), 8.69 (d, *J* = 5.4 Hz, 1H), 8.16 (d, *J* = 2.5 Hz, 1H), 7.89 (d, *J* = 8.8 Hz, 1H), 7.86 (d, *J* = 8.8 Hz, 1H), 7.70 (d, *J* = 5.4 Hz, 1H), 7.56 (d, *J* = 8.7 Hz, 1H), 7.31 (dd, *J* = 8.8, 2.5 Hz, 1H), 4.05 (s, 3H). ^{13}C NMR (126 MHz, $CDCl_3$) δ 159.44, 146.67, 144.77, 136.41, 131.53, 130.89, 130.41, 126.97, 124.65, 122.37, 121.30, 117.77, 103.14, 55.61. DART-MS m/z calcd for $C_{14}H_{11}NO$ $(M + H)^+$ = 210.0841, found 210.0917.

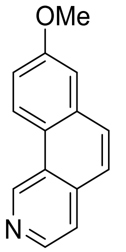

(***8-methoxybenzo[h]isoquinoline***) **(15):** The general metathesis procedure was followed using 2-(4-(2-methylprop-1-en-1-yl)pyridin-3-yl)benzaldehyde (38.5 mg, 0.144 mmol). The crude residue was purified by column chromatography with 20% ethyl acetate:hexanes gradient to furnish the title compound as a light brown solid (17.8 mg, 85.0 µmol, 59% yield). ^{1}H NMR (400 MHz, $CDCl_3$) δ 9.96 (s, 1H), 8.70 (d, *J* = 9.1 Hz, 1H), 8.65 (d, *J* = 5.4 Hz, 1H), 7.87 (d, *J* = 8.9 Hz, 1H), 7.71 – 7.65 (m, 2H), 7.38 (dd, *J* = 9.0, 2.7 Hz, 1H), 7.30 (d, *J* = 2.7 Hz, 1H), 3.98 (s, 3H). ^{13}C NMR (126 MHz, $CDCl_3$) δ 158.93, 146.24, 143.96, 134.80, 133.79, 131.30, 125.45, 125.29, 123.59, 123.53, 121.25, 118.45, 109.07, 55.50. APCI-MS m/z calcd for $C_{14}H_{11}NO$ $(M + H)^+$ = 210.0841, found 210.0917.

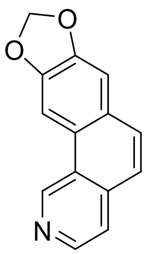

(***[1,3]dioxolo[4',5':5,6]benzo[1,2-h]isoquinoline***) **(16):** The general metathesis procedure was followed using 6-(4-(2-methylprop-1-en-1-yl)pyridin-3-yl)benzo[d][1,3]dioxole-5-carboxaldehyde (52.4 mg, 0.186 mmol). The crude residue was purified by column chromatography with 40% ethyl acetate:hexanes gradient to furnish the title compound as a brown solid (27.5 mg, 0.123 mmol, 66% yield). ^{1}H NMR (500 MHz, $CDCl_3$) δ 9.83 (s, 1H), 8.62 (d, J = 5.4 Hz, 1H), 8.09 (s, 1H), 7.78 (d, J = 8.7 Hz, 1H), 7.65 (d, J = 5.4 Hz, 1H), 7.57 (d, J = 8.8 Hz, 1H), 7.23 (s, 1H), 6.13 (s, 2H). ^{13}C NMR (126 MHz, $CDCl_3$) δ 149.10, 148.23, 146.69, 143.87, 135.00, 131.07, 128.84, 125.75, 124.96, 123.18, 121.08, 106.09, 101.71, 100.10. DART-MS m/z calcd for $C_{14}H_9NO_2$ $(M + H)^+$ = 224.0633, found 224.0823.

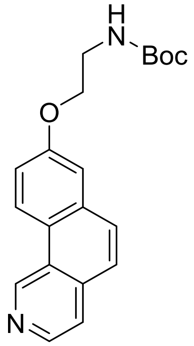

(***tert-butyl (2-(benzo[h]isoquinolin-8-yloxy)ethyl)carbamate***) **(17):** The general metathesis procedure was followed using tert-butyl (2-(3-formyl-4-(4-(2-methylprop-1-en-1-yl)pyridin-3-yl)phenoxy)ethyl)carbamate (58.3 mg, 0.147 mmol). The crude residue was purified by column chromatography with 50% ethyl acetate:hexanes gradient to furnish the title compound as a pale-yellow solid (22.0 mg, 65.0 µmol, 44% yield). ^{1}H NMR (500 MHz, $CDCl_3$) δ 9.96 (s, 1H), 8.68 (d, J = 9.1 Hz, 2H), 7.85 (d, J = 8.8 Hz, 1H), 7.68 (dd, J = 11.5, 7.0 Hz, 2H), 7.35 (dd, J = 9.0, 2.6 Hz, 1H), 7.28 (d, J = 2.6 Hz, 1H), 5.11 (s, 1H), 4.19 (t, J = 5.1 Hz, 2H), 3.63 (q, J = 5.4 Hz, 2H), 1.46 (s, 9H). ^{13}C NMR (126 MHz, $CDCl_3$) δ 157.91, 155.96, 146.10, 143.84, 134.92, 133.74, 131.34, 125.53, 123.73, 123.68, 121.35, 118.55, 109.87, 79.69, 67.45, 40.14, 29.71, 28.42. DART-MS m/z calcd for $C_{20}H_{22}N_2O_3$ $(M + H)^+$ = 339.1630, found 339.1711.

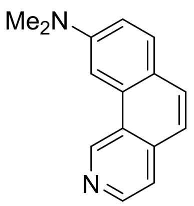

(***N,N-dimethylbenzo[h]isoquinolin-9-amine***) **(18):** The general metathesis procedure was followed using 4-(dimethylamino)-2-(4-(2-methylprop-1-en-1-yl)pyridin-3-yl)benzaldehyde (34.7 mg, 0.124 mmol). The crude residue was purified by column chromatography with 30% ethyl acetate:hexanes gradient to furnish the title compound as an orange solid (18.6 mg, 84.0 µmol, 68% yield). ^{1}H NMR (500 MHz, $CDCl_3$) δ 9.96 (s, 1H), 8.63 (d, J = 5.3 Hz, 1H), 7.87 (d, J = 2.6 Hz, 1H), 7.79 (dd, J = 14.3, 8.8 Hz, 2H), 7.64 (d, J = 5.4 Hz, 1H), 7.40 (d, J = 8.7 Hz, 1H), 7.20 (dd, J = 8.9, 2.5 Hz, 1H), 3.18 (s, 6H).^{13}C NMR (126 MHz, $CDCl_3$) δ 150.01, 146.64, 144.48, 136.78, 131.70, 130.96, 129.82, 124.54, 124.01, 121.21, 120.23, 115.15, 101.79, 40.72, 40.69. DART-MS m/z calcd for $C_{15}H_{14}N_2$ $(M + H)^+$ = 223.1157, found 223.1231.

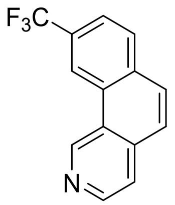

(***9-(trifluoromethyl)benzo[h]isoquinoline***) **(19):** The general metathesis procedure was followed using 2-(4-(2-methylprop-1-en-1-yl)pyridin-3-yl)-4-(trifluoromethyl)benzaldehyde (6.60 mg, 22.0 µmol). The crude residue was purified by column chromatography with 20% ethyl acetate:hexanes gradient to furnish the title compound as a white solid (1.70 mg, 7.00 µmol, 32% yield). ^{1}H NMR (500 MHz, $CDCl_3$) δ 10.09 (s, 1H), 9.07 (s, 1H), 8.80 (d, J = 5.4 Hz, 1H), 8.07 (d, J = 8.3 Hz, 1H), 8.01 (d, J = 8.9 Hz, 1H), 7.89 (dd, J = 8.3, 1.7 Hz, 1H), 7.84 (d, J = 8.9 Hz, 1H), 7.79 (d, J = 5.4 Hz, 1H). ^{13}C NMR (126 MHz, $CDCl_3$) δ 146.51, 145.64, 136.30, 133.99, 131.12, 129.88, 129.81, 129.77 (d, J = 32.7 Hz), 129.62, 128.92, 127.24, 125.32, 124.84, 123.62, 123.60 (d, J = 3.4 Hz), 123.59, 123.16, 121.47, 119.60, 119.59 (d, J = 4.3 Hz), 119.57. ^{19}F NMR (470 MHz, $CDCl_3$) δ -62.02. DART-MS m/z calcd for $C_{14}H_8F_3N$ $(M + H)^+$ = 248.0609, found 248.0680.

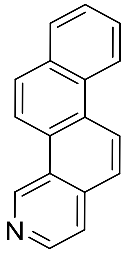

(***naphtho[1,2-h]isoquinoline***) **(20):** The general metathesis procedure was followed using 2-(4-(2-methylprop-1-en-1-yl)pyridin-3-yl)-1-naphthaldehyde (31.6 mg, 0.110 mmol) and catalyst (20 mol%). The crude residue was purified by column chromatography with 25% ethyl acetate:hexanes gradient to furnish the title compound as a brown solid (8.60 mg, 38.0 µmol, 35% yield). ^{1}H NMR (500 MHz, $CDCl_3$) δ 10.41 (s, 1H), 9.09 (d, J = 8.4 Hz, 1H), 8.70 (d, J = 5.4 Hz, 1H), 8.05 (dd, J = 9.6, 8.1 Hz, 2H), 7.98 (d, J = 8.5 Hz, 1H), 7.88 – 7.82 (m, 3H), 7.76 (ddd, J = 8.4, 6.8, 1.6 Hz, 1H), 7.70 (t, J = 7.5 Hz, 1H). ^{13}C NMR (126 MHz, $CDCl_3$) δ 150.74, 143.40, 136.68, 133.79, 131.60, 131.42, 129.36, 128.75, 128.47, 128.17, 127.01, 126.85, 126.79, 126.60, 125.58, 125.48, 120.95. DART-MS m/z calcd for $C_{17}H_{11}N$ $(M + H)^+$ = 230.0891, found 230.0961.

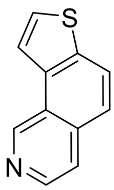

(***thieno[2,3-h]isoquinoline***) **(21):** The general metathesis procedure was followed using 2-(4-(2-methylprop-1-en-1-yl)pyridin-3-yl)benzaldehyde (80.0 mg, 0.329 mmol) and catalyst (20 mol%). The crude residue was purified by column chromatography with 50% ethyl acetate:hexanes gradient to furnish the title compound as a white solid (41.3 mg, 0.223 mmol, 68% yield). ^{1}H NMR (400 MHz, $CDCl_3$) δ 9.75 (s, 1H), 8.63 (d, J = 5.6 Hz, 1H), 8.10 (dd, J = 10.4, 7.1 Hz, 2H), 7.81 – 7.64 (m, 3H). ^{13}C NMR (126 MHz, $CDCl_3$) δ 147.97, 143.29, 138.47, 135.20, 134.11, 128.01, 125.33, 124.31, 122.99, 121.09, 121.05. DART-MS m/z calcd for $C_{11}H_7NS$ $(M + H)^+$ = 186.0299, found 186.0372.

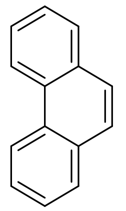

(***phenanthrene***) **(22):** The general metathesis procedure was followed using 2'-(2-methylprop-1-en-1-yl)-[1,1'-biphenyl]-2-carboxaldehyde (47.3 mg, 0.200 mmol). The crude residue was purified by column chromatography with 5% ethyl acetate:hexanes gradient to furnish the title compound as a white solid (29.9 mg, 0.168 mmol, 84% yield). ^{1}H NMR (500 MHz, $CDCl_3$) δ 8.75 (d, *J* = 8.1 Hz, 2H), 7.96 (d, *J* = 7.7 Hz, 2H), 7.81 (s, 2H), 7.72 (t, *J* = 7.0 Hz, 2H), 7.67 (t, *J* = 7.3 Hz, 2H). DART-MS m/z calcd for $C_{14}H_{10}$ $(M + H)^+$ = 179.0783, found 179.0859.

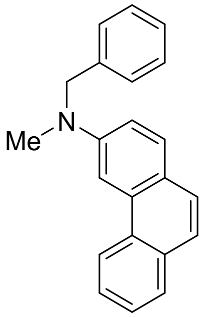

(***N-benzyl-N-methylphenanthren-3-amine***) **(23):** The general metathesis procedure was followed using 2'-(2-methylprop-1-en-1-yl)-[1,1'-biphenyl]-2-carboxaldehyde (31.6 mg, 89.0 µmol). The crude residue was purified by column chromatography with 5% ethyl acetate:hexanes gradient to furnish the title compound as a pale-yellow solid (18.1 mg, 61.0 µmol, 69% yield). ^{1}H NMR (500 MHz, $CDCl_3$) δ 8.54 (dd, *J* = 7.4, 2.0 Hz, 1H), 7.92 – 7.79 (m, 2H), 7.74 (d, *J* = 8.8 Hz, 1H), 7.62 (d, *J* = 8.7 Hz, 1H), 7.60 – 7.52 (m, 2H), 7.50 (d, *J* = 8.7 Hz, 1H), 7.37 – 7.31 (m, 4H), 7.30 – 7.26 (m, 1H), 7.19 (dd, *J* = 8.8, 2.5 Hz, 1H), 4.74 (s, 2H), 3.23 (s, 3H).^{13}C NMR (126 MHz, $CDCl_3$) δ 148.53, 138.82, 132.76, 131.68, 129.67, 129.58, 128.70, 128.52, 127.07, 126.86, 126.70, 126.30, 125.68, 124.11, 122.83, 122.64, 114.77, 103.15, 56.96, 38.93. DART-MS m/z calcd for $C_{22}H_{19}N$ $(M + H)^+$ = 298.1517, found 298.1594.

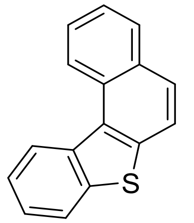

(***benzo[b]naphtho[1,2-d]thiophene***) **(24):** The general metathesis procedure was followed using 3-(2-(2-methylprop-1-en-1-yl)benzo[b]thiophen-3-yl)thiophene-2-carboxaldehyde (100 mg, 0.342 mmol). The crude residue was purified by column chromatography with 1% ethyl acetate:hexanes gradient to furnish the title compound as a white solid (58.9 mg, 0.251 mmol, 73% yield). ^{1}H NMR (400 MHz, $CDCl_3$) δ 9.02 (dt, *J* = 8.5, 0.9 Hz, 1H), 8.87 (dt, *J* = 8.4, 1.0 Hz, 1H), 8.06 – 7.99 (m, 2H), 7.95 – 7.88 (m, 2H), 7.75 (ddd, *J* = 8.5, 6.9, 1.5 Hz, 1H), 7.60 (tdd, *J* = 7.9, 7.0, 1.2 Hz, 2H), 7.51 (ddd, *J* = 8.1, 7.2, 1.1 Hz, 1H). ^{13}C NMR (126 MHz, $CDCl_3$) δ 139.76, 138.65, 136.74, 131.96, 130.68, 129.48, 129.07, 127.89, 127.17, 125.26, 124.93, 124.84, 124.75, 123.25, 123.22, 121.11. DART-MS m/z calcd for $C_{16}H_{10}S$ $(M + H)^+$ = 235.0503, found 235.0534.

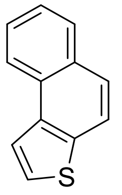

(***naphtho[2,1-b]thiophene***) **(25):** The general metathesis procedure was followed using2-(2-(2-methylprop-1-en-1-yl)thiophen-3-yl)benzaldehyde (60.0 mg, 0.248 mmol). The crude residue was purified by column chromatography with 1% ethyl acetate:hexanes gradient to furnish the title compound as a white solid (38.0 mg, 0.206 mmol, 83% yield). ^{1}H NMR (500 MHz, $CDCl_3$) δ 8.39 – 8.29 (m, 1H), 8.01 (d, *J* = 5.4 Hz, 1H), 7.96 (dd, *J* = 8.0, 1.3 Hz, 1H), 7.91 (d, *J* = 8.8 Hz, 1H), 7.76 (d, *J* = 8.8 Hz, 1H), 7.67 – 7.57 (m, 2H), 7.55 (ddd, *J* = 8.1, 6.9, 1.3 Hz, 1H). ^{13}C NMR (126 MHz, $CDCl_3$) δ 137.40, 135.94, 130.99, 129.37, 128.56, 126.48, 125.83, 125.30, 125.06, 123.62, 122.03, 120.69. DART-MS m/z calcd for $C_{12}H_8S$ $(M + H)^+$ = 185.0347, found 185.0379.

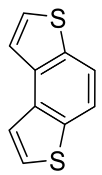

(***benzo[1,2-b:4,3-b']dithiophene***) **(26):** The general metathesis procedure was followed using 2'-(2-methylprop-1-en-1-yl)-[3,3'-bithiophene]-2-carboxaldehyde (40.0 mg, 0.161 mmol). The crude residue was purified by column chromatography with 1% ethyl acetate:hexanes gradient to furnish the title compound as a white solid (19.5 mg, 0.102 mmol, 63% yield). ^{1}H NMR (500 MHz, $CDCl_3$) δ 7.83 (s, 2H), 7.72 (d, *J* = 5.3 Hz, 2H), 7.57 (d, *J* = 5.4 Hz, 2H). ^{13}C NMR (126 MHz, $CDCl_3$) δ 136.44, 134.66, 126.50, 121.93, 118.77. DART-MS m/z calcd for $C_{10}H_6S_2$ $(M + H)^+$ = 190.9911, found 190.9908.

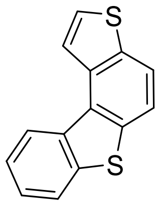

(***[1]Benzothieno[5,4-b][1]benzothiophen***) **(27):** The general metathesis procedure was followed using 3-(2-(2-methylprop-1-en-1-yl)benzo[b]thiophen-3-yl)thiophene-2-carboxaldehyde (96.0 mg, 0.322 mmol). The crude residue was purified by column chromatography with 1% dichloromethane:hexanes gradient to furnish the title compound as a white solid (16.8 mg, 70.0 µmol, 22% yield). ^{1}H NMR (500 MHz, $CDCl_3$) δ 8.55 (d, *J* = 8.0 Hz, 1H), 8.29 – 8.21 (m, 1H), 8.03 – 7.93 (m, 2H), 7.84 (d, *J* = 8.5 Hz, 1H), 7.74 (d, *J* = 5.5 Hz, 1H), 7.57 (ddd, *J* = 8.1, 7.1, 1.2 Hz, 1H), 7.51 (td, *J* = 7.6, 1.2 Hz, 1H). ^{13}C NMR (126 MHz, $CDCl_3$) δ 139.56, 137.61, 136.46, 135.73, 134.64, 129.53, 127.76, 125.82, 124.60, 123.71, 123.02, 121.36, 121.04, 119.04. DART-MS m/z calcd for $C_{14}H_8S_2$ $(M + H)^+$ = 241.0067, found 241.0099.

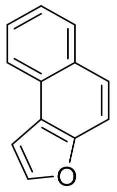

(***naphtho[2,1-b]furan***) **(28):** The general metathesis procedure was followed using 2-(2-(2-methylprop-1-en-1-yl)furan-3-yl)benzaldehyde (51.0 mg, 0.225 mmol). The crude residue was purified by column chromatography with 1% ethyl acetate:hexanes gradient to furnish the title compound as a pale-yellow solid (29.9 mg, 0.178 mmol, 79% yield). ^{1}H NMR (500 MHz, $CDCl_3$) δ 8.16 (dd, *J* = 8.3, 1.2 Hz, 1H), 8.00 – 7.95 (m, 1H), 7.79 (d, *J* = 2.0 Hz, 1H), 7.75 (d, *J* = 9.0 Hz, 1H), 7.70 (dd, *J* = 8.9, 0.9 Hz, 1H), 7.61 (ddd, *J* = 8.2, 6.9, 1.3 Hz, 1H), 7.51 (ddd, *J* = 8.1, 6.9, 1.2 Hz, 1H), 7.29 (dd, *J* = 2.1, 0.9 Hz, 1H). ^{13}C NMR (126 MHz, $CDCl_3$) δ 152.55, 144.23, 130.36, 128.76, 127.85, 126.32, 125.21, 124.52, 123.45, 122.67, 112.55, 105.62. DART-MS m/z calcd for $C_{12}H_8O$ $(M + H)^+$ = 169.0575, found 169.0606.

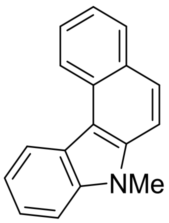

(***7-methyl-7H-benzo[c]carbazole***) **(29):** The general metathesis procedure was followed using 2-(1-methyl-2-(2-methylprop-1-en-1-yl)-1H-indol-3-yl)benzaldehyde (86.2 mg, 0.298 mmol) and catalyst (20 mol%). The crude residue was purified by column chromatography with 5% dichloromethane:hexanes gradient to furnish the title compound as a white solid (21.7 mg, 94.0 μmol, 32% yield). ^{1}H NMR (500 MHz, $CDCl_3$) δ 8.82 (d, *J* = 8.3 Hz, 1H), 8.61 (d, *J* = 8.0 Hz, 1H), 8.03 (dd, *J* = 8.1, 1.3 Hz, 1H), 7.92 (d, *J* = 8.8 Hz, 1H), 7.73 (ddd, *J* = 8.3, 6.9, 1.3 Hz, 1H), 7.65 (d, *J* = 8.8 Hz, 1H), 7.60 – 7.46 (m, 3H), 7.41 (ddd, *J* = 8.1, 6.6, 1.6 Hz, 1H), 3.97 (s, 3H). ^{13}C NMR (126 MHz, $CDCl_3$) δ 139.93, 138.51, 129.97, 129.23, 128.90, 127.22, 126.90, 124.10, 123.43, 123.19, 122.78, 122.06, 119.76, 114.84, 110.56, 109.11, 29.28. DART-MS m/z calcd for $C_{17}H_{13}N$ $(M + H)^+$ = 232.1048, found 232.1080.

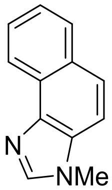

(***3-methyl-3H-naphtho[1,2-d]imidazole***) **(30):** The general metathesis procedure was followed using 2-(1-methyl-5-(2-methylprop-1-en-1-yl)-1H-imidazol-4-yl)benzaldehyde (11.5 mg, 48.0 µmol) and catalyst (20 mol%). The crude residue was purified by column chromatography with 10% methanol:dichloromethane gradient to furnish the title compound as a light brown solid (3.8 mg, 0.021 mmol, 44% yield). ^{1}H NMR (500 MHz, $CDCl_3$) δ 8.71 – 8.66 (m, 1H), 8.09 (s, 1H), 7.95 (d, J = 8.1 Hz, 1H), 7.76 (d, J = 8.8 Hz, 1H), 7.66 (ddd, J = 8.2, 6.9, 1.2 Hz, 1H), 7.56 – 7.49 (m, 2H), 3.99 (s, 3H). ^{13}C NMR (126 MHz, $CDCl_3$) δ 140.68, 130.48, 130.34, 128.42, 126.99, 126.47, 125.02, 124.80, 122.01, 109.84, 31.68. DART-MS m/z calcd for $C_{12}H_{10}N_2$ $(M + H)^+$ = 183.0844, found 183.0915.

d. Screen of the olefin moiety

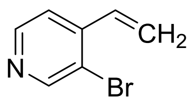

(***3-bromo-4-vinylpyridine***) **(66) :** The title compound was prepared according to a published procedure and the characterization data matched that of the reported compound.[3] A mixture of 3,4-dibromopyridine (300 mg, 1.27 mmol, 1.0 equiv), 4,4,5,5-tetramethyl-2-vinyl-1,3,2-dioxaborolane (195 mg, 1.27 mmol, 1.0 equiv), aq. K_3PO_4 (3.80 mL, 1 M, 3.80 mmol, 3.0 equiv), and Pd(dppf)Cl_2 (92.7 mg, 0.127 mmol, 10 mol%) in DMF (12.7 mL, 0.1M) was stirred at 60 °C under N_2 for 3 h. The reaction mixture was diluted with H_2O and extracted with ethyl acetate. The organic layer was washed with H_2O and then brine and then dried over Na_2SO_4. After concentration, the crude residue was purified by column chromatography with 10% ethyl acetate:hexanes gradient to furnish the title compound as a light-yellow liquid (170 mg, 0.924 mmol, 73% yield). ^{1}H NMR (400 MHz, $CDCl_3$) δ 8.70 (s, 1H), 8.45 (d, J = 5.1 Hz, 1H), 7.41 (d, J = 5.1 Hz, 1H), 6.99 (dd, J = 17.5, 11.0 Hz, 1H), 5.94 (d, J = 17.5 Hz, 1H), 5.59 (d, J = 11.0 Hz, 1H). ^{13}C NMR (101 MHz, $CDCl_3$) δ 152.49, 148.27, 144.48, 133.66, 121.41, 120.85, 120.73. DART-MS m/z calcd for C_7H_6BrN $(M + H)^+$ = 183.9684, found 183.9775.

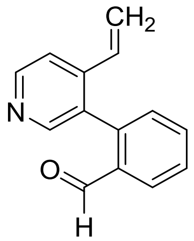

(***2-(4-vinylpyridin-3-yl)benzaldehyde***) **(31) :** The general cross-coupling procedure was followed using 3-bromo-4-vinylpyridine (35.0 mg, 0.190 mmol). The crude residue was purified by column chromatography with 25% ethyl acetate:hexanes gradient to furnish the title compound as a yellow liquid (31.0 mg, 0.148 mmol, 78% yield). ^{1}H NMR (500 MHz, $CDCl_3$) δ 9.73 (d, J = 0.8 Hz, 1H), 8.65 (d, J = 5.3 Hz, 1H), 8.51 (d, J = 0.8 Hz, 1H), 8.06 (dd, J = 7.8, 1.5 Hz, 1H), 7.69 (td, J = 7.5, 1.5 Hz, 1H), 7.59 (tt, J = 7.5, 1.1 Hz, 1H), 7.53 (d, J = 5.3 Hz, 1H), 7.34 (dd, J = 7.7, 1.3 Hz, 1H), 6.38 (dd, J = 17.5, 11.0 Hz, 1H), 5.90 (dd, J = 17.5, 0.8 Hz, 1H), 5.41 (dd, J = 11.0, 0.7 Hz, 1H). ^{13}C NMR (126 MHz, $CDCl_3$) δ 191.13, 150.87, 149.72, 144.07, 140.18, 134.51, 133.93, 132.56, 131.92, 131.50, 128.92, 128.14, 120.65, 119.07. DART-MS m/z calcd for $C_{14}H_{11}NO$ $(M + H)^+$ = 210.0841, found 210.0917.

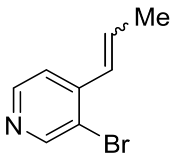

(E/Z)-3-bromo-4-(prop-1-en-1-yl)pyridine **(67) :** The general olefination procedure was followed using 3-bromoisonicotinaldehyde (500 mg, 2.69 mmol) and ethyltriphenylphosphonium iodide (1.24 g, 2.96 mmol). The crude residue was purified by column chromatography with 10% ethyl acetate:hexanes gradient to furnish the title compound (*E/Z* mixture, 4:1 ratio) as a yellow oil (200 g, 1.01 mmol, 38% yield). ^{1}H NMR (400 MHz, $CDCl_3$) δ 8.71 (s, 1H), 8.65 (s, 0H), 8.45 (d, *J* = 4.9 Hz, 1H), 8.38 (d, *J* = 5.0 Hz, 0H), 7.34 (d, *J* = 5.1 Hz, 0H), 7.22 (d, *J* = 4.9 Hz, 1H), 6.71 – 6.61 (m, 0H), 6.51 – 6.44 (m, 0H), 6.41 (dq, *J* = 11.6, 1.8 Hz, 1H), 6.05 (dq, *J* = 11.6, 7.2 Hz, 1H), 1.96 (dd, *J* = 6.6, 1.7 Hz, 1H), 1.82 (dd, *J* = 7.2, 1.8 Hz, 3H). ^{13}C NMR (101 MHz, $CDCl_3$) δ 152.39, 152.09, 148.00, 147.69, 144.97, 144.66, 133.64, 131.63, 127.83, 127.05, 124.92, 18.90, 14.63. DART-MS m/z calcd for C_8H_8BrN $(M + H)^+$ = 197.9840, found 197.9913.

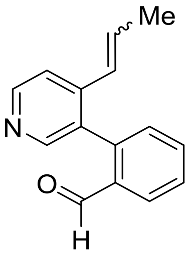

(E/Z)-2-(4-(prop-1-en-1-yl)pyridin-3-yl)benzaldehyde **(32) :** The general cross-coupling procedure was followed using (*E/Z*)-3-bromo-4-(prop-1-en-1-yl)pyridine (100 mg, 0.505 mmol). The crude residue was purified by column chromatography with 30% ethyl acetate:hexanes gradient to furnish the title compound (*E/Z* mixture, 2:1 ratio) as a yellow liquid (60.0 mg, 0.269 mmol, 53 % yield). ^{1}H NMR (500 MHz, $CDCl_3$) δ 9.71 (d, *J* = 0.8 Hz, 1H), 9.67 (d, *J* = 0.8 Hz, 1H), 8.64 (d, *J* = 5.1 Hz, 1H), 8.58 (d, *J* = 5.3 Hz, 1H), 8.55 (s, 1H), 8.45 (s, 1H), 8.06 (dd, *J* = 7.7, 1.5 Hz, 1H), 8.02 (dd, *J* = 7.8, 1.5 Hz, 1H), 7.74 – 7.63 (m, 2H), 7.61 – 7.52 (m, 2H), 7.46 (d, *J* = 5.3 Hz, 1H), 7.35 – 7.30 (m, 2H), 6.43 (dq, *J* = 15.7, 6.7 Hz, 1H), 6.06 – 5.99 (m, 1H), 5.96 (dt, *J* = 11.7, 1.8 Hz, 1H), 5.85 (dq, *J* = 11.8, 7.1 Hz, 1H), 1.82 (dd, *J* = 7.1, 1.7 Hz, 3H), 1.78 (dd, *J* = 6.8, 1.7 Hz, 3H). ^{13}C NMR (126 MHz, $CDCl_3$) δ 191.31, 191.19, 150.84, 150.54, 149.52, 149.21, 144.53, 144.37, 140.69, 134.46, 134.37, 133.95, 133.85, 133.39, 131.92, 131.47, 131.24, 131.16, 128.76, 128.70, 127.84, 127.77, 126.63, 126.36, 123.47, 119.03, 18.88, 14.57. DART-MS m/z calcd for $C_{15}H_{13}NO$ $(M + H)^+$ = 224.0997, found 224.1073.

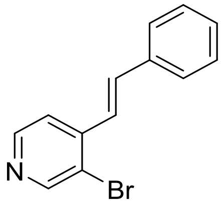

(*(E)-3-bromo-4-styrylpyridine*) (68): The general olefination procedure was followed using 3-bromoisonicotinaldehyde (500 mg, 2.69 mmol) and benzyltriphenylphosphonium bromide (1.28 g, 2.96 mmol). The crude residue was purified by column chromatography with 15% ethyl acetate:hexanes gradient to furnish the title compound as a yellow oil (*E*:*Z*=1:1.9, 655 mg, 2.52 mmol, 94 % yield, for *E*-isomer: 214 mg, 0.822 mmol). ^{1}H NMR (400 MHz, $CDCl_3$) δ 8.74 (s, 1H), 8.49 (d, *J* = 5.2 Hz, 1H), 7.64 – 7.58 (m, 2H), 7.56 (d, *J* = 5.2 Hz, 1H), 7.47 – 7.34 (m, 4H), 7.29 (d, *J* = 9.5 Hz, 1H). ^{13}C NMR (101 MHz, $CDCl_3$) δ 152.61, 148.18, 144.26, 135.95, 135.37, 129.21, 128.95, 127.35, 124.70, 120.39. DART-MS m/z calcd for $C_{13}H_{10}BrN$ $(M + H)^+$ = 259.9997, found 260.0052.

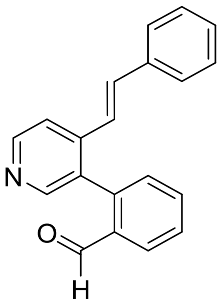

(*(E)-2-(4-styrylpyridin-3-yl)benzaldehyde*) (33) : The general cross-coupling procedure was followed using 3-bromo-4-vinylpyridine (100 mg, 0.384 mmol). The crude residue was purified by column chromatography with 45% ethyl acetate:hexanes gradient to furnish the title compound as a yellow liquid (103 mg, 0.362 mmol, 94 % yield). ^{1}H NMR (500 MHz, $CDCl_3$) δ 9.77 (s, 1H), 8.63 (d, *J* = 5.3 Hz, 1H), 8.49 (s, 1H), 8.07 (dd, *J* = 7.8, 1.4 Hz, 1H), 7.69 (td, *J* = 7.5, 1.5 Hz, 1H), 7.64 – 7.56 (m, 2H), 7.35 (dd, *J* = 7.6, 1.3 Hz, 1H), 7.26 (dd, *J* = 12.8, 2.6 Hz, 5H), 7.21 (d, *J* = 16.3 Hz, 1H), 6.69 (d, *J* = 16.2 Hz, 1H). ^{13}C NMR (126 MHz, $CDCl_3$) δ 191.07, 151.06, 149.59, 143.88, 140.31, 135.95, 135.06, 134.62, 134.00, 132.02, 131.72, 128.99, 128.98, 128.81, 128.25, 127.11, 123.44, 118.95. DART-MS m/z calcd for $C_{20}H_{15}NO$ $(M + H)^+$ = 286.1154, found 286.1231.

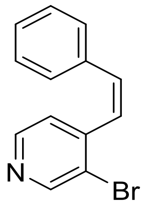

(***(Z)*-3-bromo-4-styrylpyridine**) **(69):** The general olefination procedure was followed using 3-bromoisonicotinaldehyde (500 mg, 2.69 mmol) and benzyltriphenylphosphonium bromide (1.28 g, 2.96 mmol). The crude residue was purified by column chromatography with 10% ethyl acetate:hexanes gradient to furnish the title compound as a yellow solid (*E*:*Z*=1:1.9, 655 mg, 2.52 mmol, 94 % yield, for *Z*-isomer: 441 mg, 1.70 mmol). ^{1}H NMR (500 MHz, $CDCl_3$) δ 8.78 (s, 1H), 8.29 (d, J = 5.0 Hz, 1H), 7.26 (dd, J = 4.9, 1.9 Hz, 3H), 7.20 – 7.14 (m, 2H), 7.09 (d, J = 5.0 Hz, 1H), 6.90 (d, J = 12.2 Hz, 1H), 6.56 (d, J = 12.2 Hz, 1H). ^{13}C NMR (126 MHz, $CDCl_3$) δ 152.38, 147.71, 145.73, 135.42, 134.51, 128.90, 128.48, 128.11, 126.69, 125.02, 122.21. DART-MS m/z calcd for $C_{13}H_{10}BrN$ $(M + H)^+$ = 259.9997, found 260.0050.

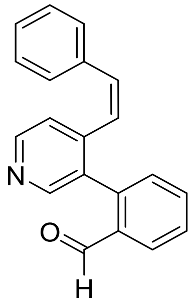

(***(Z)*-2-(4-styrylpyridin-3-yl)benzaldehyde**) **(34) :** The general cross-coupling procedure was followed using 3-bromo-4-vinylpyridine (100 mg, 0.384 mmol). The crude residue was purified by column chromatography with 35% ethyl acetate:hexanes gradient. The fractions were combined and concentrated in vacuo, providing the title compound (108 mg, 0.378 mmol, 98 % yield) as a yellow liquid. ^{1}H NMR (500 MHz, $CDCl_3$) δ 9.88 (s, 1H), 8.65 – 8.46 (m, 2H), 8.04 (dd, J = 7.7, 1.5 Hz, 1H), 7.62 (td, J = 7.5, 1.5 Hz, 1H), 7.56 (tt, J = 7.6, 0.9 Hz, 1H), 7.34 (d, J = 5.2 Hz, 1H), 7.28 – 7.22 (m, 4H), 7.13 – 7.05 (m, 2H), 6.66 (d, J = 12.2 Hz, 1H), 6.27 (d, J = 12.2 Hz, 1H). ^{13}C NMR (126 MHz, $CDCl_3$) δ 190.98, 151.08, 149.07, 144.84, 140.34, 135.68, 134.82, 134.12, 133.63, 132.94, 131.47, 128.62, 128.58, 128.47, 128.21, 128.05, 125.65, 123.58. DART-MS m/z calcd for $C_{20}H_{15}NO$ $(M + H)^+$ = 286.1154, found 286.1231.

e. Substrate Precursor Synthesis

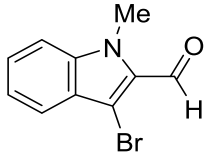

(***3-bromo-1-methyl-1H-indole-2-carboxaldehyde***) **(70):** The compound was prepared according to a published procedure and the characterization data matched that of the reported compound.[4] To a solution of 1-methyl-1H-indole-2-carboxaldehyde (1.00 g, 6.28 mmol, 1.0 equiv) in chloroform (0.025 M, 251 mL) was added *N*-bromosucciinmide (NBS) (2.25 g, 13.8 mmol, 2.2 equiv). The resultant mixture was stirred at room temperature and monitored by TLC. After removing the solvent in vacuo, the crude residue was purified by column chromatography with 10% ethyl acetate:hexanes gradient to furnish the title compound as a light yellow solid (1.20 g, 5.04 mmol, 80% yield). ^{1}H NMR (500 MHz, $CDCl_3$) δ 10.10 (s, 1H), 7.67 (dt, *J* = 8.2, 1.0 Hz, 1H), 7.46 (ddd, *J* = 8.2, 6.9, 1.2 Hz, 1H), 7.35 (dt, *J* = 8.6, 0.9 Hz, 1H), 7.25 (ddd, *J* = 7.9, 6.9, 0.9 Hz, 1H), 4.04 (s, 3H). The ^{1}H NMR spectrum matched with the reported values.[4]

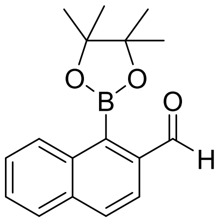

(***1-(4,4,5,5-tetramethyl-1,3,2-dioxaborolan-2-yl)-2-naphthaldehyde***) **(71) :** The compound was prepared according to a published procedure and the characterization data matched that of the reported compound.[5] 1-bromo-2-naphthaldehyde (100 mg, 0.425 mmol, 1.0 equiv), KOAc (125 mg, 1.28 mmol, 3.0 equiv), $PdCl_2$(dppf) (9.30 mg, 13.0 μmol, 0.03 equiv), and bispinacolatodiboron (118 mg, 0.468 mmol, 1.1 equiv) were dissolved in anhydrous degassed toluene (0.2 M, 2.0 mL) and the reaction mixture was heated to reflux overnight. After completion, the reaction mixture was cooled to room temperature and filtered through a pad of Celite and concentrated in vacuo. The crude residue was purified by column chromatography with 20% ethyl acetate:hexanes gradient to furnish the title compound as a white solid (94.2 mg, 0.334 mmol, 78 % yield). ^{1}H NMR (500 MHz, $CDCl_3$) δ 10.20 (s, 1H), 8.12 – 8.09 (m, 1H), 7.89 (d, *J* = 8.5 Hz, 1H), 7.84 (d, *J* = 8.5 Hz, 1H), 7.80 (dd, *J* = 7.6, 1.7 Hz, 1H), 7.55 – 7.46 (m, 2H), 1.47 (s, 12H). The ^{1}H NMR spectrum matched with the reported values.[5]

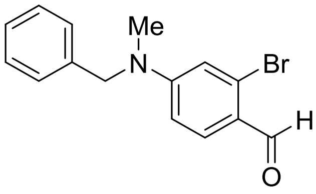

(*4-(benzyl(methyl)amino)-2-bromobenzaldehyde*) **(72) :** The compound was prepared according to a published procedure and the characterization data matched that of the reported compound.[6] A solution of 2-bromo-4-fluorobenzaldehyde (1.00 g, 4.93 mmol, 1.0 equiv), N-methylbenzylamine (0.597 g, 4.93 mmol, 1.0 equiv), and K_2CO_3 (1.36 g, 9.85 mmol, 2.0 equiv) in DMF (0.3 M, 16.4mL) was heated to 100 °C overnight. The solution was cooled to room temperature and diluted with dichloromethane and water. The biphasic solution was extracted with dichloromethane three times. The combined organic phases were washed with brine and concentrated *in vacuo*. The crude residue was purified by column chromatography with 25% ethyl acetate:hexanes gradient to furnish the title compound (1.30 g, 4.27 mmol, 87% yield) as a light yellow oil. ^{1}H NMR (400 MHz, $CDCl_3$) δ 10.09 (d, *J* = 0.8 Hz, 1H), 7.78 (d, *J* = 8.9 Hz, 1H), 7.38 – 7.32 (m, 2H), 7.31 – 7.27 (m, 1H), 7.18 – 7.14 (m, 2H), 6.88 (d, *J* = 2.5 Hz, 1H), 6.69 (ddd, *J* = 9.0, 2.6, 0.9 Hz, 1H), 4.63 (s, 2H), 3.14 (s, 3H). The ^{1}H NMR spectrum matched with the reported values.[6]

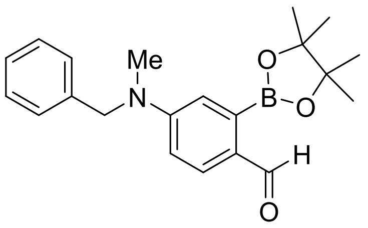

(*4-(benzyl(methyl)amino)-2-(4,4,5,5-tetramethyl-1,3,2-dioxaborolan-2-yl)benzaldehyde*) **(73) :** The compound was prepared according to a published procedure and the characterization data matched that of the reported compound.[6] To a solution of 4-(benzyl(methyl)amino)-2-bromobenzaldehyde (500 mg, 1.64 mmol. 1.0 equiv) in 1,4-dioxane (0.12M, 13.7 mL) was added bis-pinacoldiboron (459 mg, 1.81 mmol, 1.1 equiv), KOAc (484 mg, 4.93 mmol, 3.0 equiv), and Pd(dppf)Cl_2 (36.0 mg, 49.0 µmol, 3 mol%). The mixture was degassed with N_2 and heated at 90 °C overnight. The solution was cooled to room temperature and filtered through a short pack of Celite. The filtrate was concentrated, and the crude residue was purified by column chromatography with 20% ethyl acetate:hexanes gradient to furnish the title compound as a red solid (535 mg, 1.52 mmol, 93% yield). ^{1}H NMR (500 MHz, $CDCl_3$) δ 9.54 (d, *J* = 0.8 Hz, 1H), 7.91 (d, *J* = 8.9 Hz, 1H), 7.36 – 7.30 (m, 2H), 7.29 – 7.26 (m, 1H), 7.19 – 7.14 (m, 2H), 6.80 (ddd, *J* = 9.0, 2.7, 0.8 Hz, 1H), 6.54 (d, *J* = 2.6 Hz, 1H), 4.65 (s, 2H), 3.12 (s, 3H), 1.24 (s, 12H). The ^{1}H NMR spectrum matched with the reported values.[6]

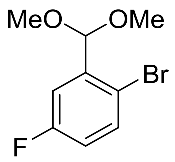

(***1-bromo-2-(dimethoxymethyl)-4-fluorobenzene***) **(74):** The compound was prepared according to a published procedure and the characterization data matched that of the reported compound.[7] To a 100 mL round bottom flask equipped with a stir bar and a reflux condenser was added 2-bromo-5-fluorobenzaldehyde (5.00 g, 24.6 mmol) dissolved in 10 ml of methanol. Concentrated sulfuric acid (0.125 mL, 2.34 mmol) was added slowly into the stirring mixture. Subsequently, trimethyl orthoformate (3.50 mL, 25.0 mmol) was added dropwise into the reaction mixture at room temperature. The solution was then heated to reflux for 1 h. After cooling to room temperature, the solution was basified to pH 11 using a concentrated NaOMe solution in methanol. The solvent was removed *in vacuo*. Subsequently, DCM (30 mL) was added to the remaining reaction slurry, and the mixture was filtered through Celite. The filtrate was collected and concentrated in vacuo to furnish the title compound as a colorless liquid (1.48 g, 24% yield). ^{1}H NMR (300 MHz, $CDCl_3$) δ 7.51 (dd, J = 8.8, 5.1 Hz, 1H), 7.35 (dd, J = 9.4, 3.1 Hz, 1H), 7.00 – 6.87 (m, 1H), 5.50 (d, J = 1.3 Hz, 1H), 3.38 (d, J = 0.7 Hz, 6H). The ^{1}H NMR spectrum matched with the reported values.[7]

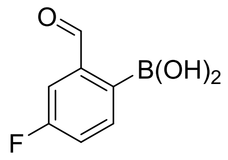

(***(4-fluoro-2-formylphenyl)boronic acid***) **(75):** The compound was prepared according to a published procedure and the characterization data matched that of the reported compound.[7] To a 100 mL flame dried round bottom flask equipped with a stir bar was added a combination of anhydrous diethyl ether (42.0 mL) and THF (8.40 mL). After being cooled to –78 °C in a dry ice/acetone bath, 1.6 M n-butyllithium in hexanes (14.4 mL, 23.1 mmol) was added, followed by a dropwise addition of 1-bromo-2-(dimethoxymethyl)-4-fluorobenzene (5.00 g, 20.1 mmol). After stirring for 1 h at –78 °C, triethyl borate (3.60 mL, 23.1 mmol) was added slowly in to the flask. The mixture was stirred for 1 hour in the dry ice/acetone bath. Then, the cooling bath was removed and the solution was acidified to pH 3 using 3 M aqueous HCl. The reaction mixture was extracted with Et_2O (3 x 50 mL) and the organic layer was combined and concentrated *in vacuo* until most of the diethyl ether had evaporated. Water (10 mL) was added into the mixture and the reaction

was concentrated *in vacuo* at 50 °C to remove water. Upon filtration and concentration *in vacuo*, the title compound was obtained as an off white solid (1.48 g, 44 % yield). ^{1}H NMR spectrum of this compound was mostly benzoxaborole resulted from intramolecular condensation. ^{1}H NMR (300 MHz, CD_3OD) δ 7.66 – 7.55 (m, 1H), 7.20 – 7.09 (m, 2H), 5.98 (s, 1H). The ^{1}H NMR spectrum matched with the reported values.[7]

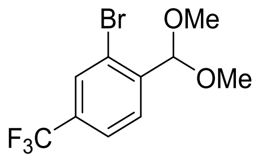

(***2-bromo-1-(dimethoxymethyl)-4-(trifluoromethyl)benzene***) **(76):** The compound was prepared according to a published procedure and the characterization data matched that of the reported compound.[8] To a 100 mL round bottom flask equipped with a stir bar and a reflux condenser was added 2-bromo-4-(trifluoromethyl)benzaldehyde (20.0 g, 79.1 mmol) dissolved in 32 ml of methanol. Concentrated sulfuric acid (0.400 mL, 7.53 mmol) was added slowly into the stirred mixture. Subsequently, trimethyl orthoformate (11.2 mL, 103 mmol) was added dropwise to the reaction mixture at room temperature. The solution was then heated to reflux for 1 h. After cooling to room temperature, the solution was basified to pH 11 using a concentrated NaOMe solution in methanol. The crude residue was concentrated *in vacuo*. Subsequently, DCM (30 mL) was added to the reaction slurry and the mixture was filtered through Celite. The filtrate was collected and concentrated in vacuo to furnish the title compound as a colorless liquid (19.6 g, 83% yield). ^{1}H NMR (300 MHz, $CDCl_3$) δ 7.83 (s, 1H), 7.74 (d, J = 8.1 Hz, 1H), 7.59 (d, J = 8.3 Hz, 1H), 5.57 (s, 1H), 3.39 (s, 6H). The ^{1}H NMR spectrum matched with the reported values.[8]

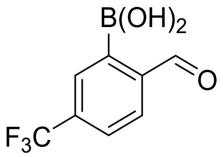

(***(2-formyl-5-(trifluoromethyl)phenyl)boronic acid***) **(77):** The compound was prepared according to a published procedure and the characterization data matched that of the reported compound.[8] To a 100 mL flame dried round bottom flask equipped with a stir bar was added a combination of anhydrous diethyl ether (34.5 mL) and THF (6.90 mL). After being cooled to –78 °C in a dry ice/acetone bath, 2.5 M n-butyllithium in hexanes (7.60 mL, 19.0 mmol) was added, followed by dropwise addition of 2-bromo-1-(dimethoxymethyl)-4-(trifluoromethyl)benzene (4.91 g, 16.4 mmol). After stirring for 1 h at –78 °C, triethyl borate (3.20 mL, 19.0 mmol) was added slowly. The mixture was stirred for 1 h in the dry ice/acetone bath. Then, the cooling bath was removed

and the solution was acidified to pH 3 using 3 M aqueous HCl. The reaction was extracted with Et_2O (3 x 50 mL), and the organic layers were combined and concentrated *in vacuo* to yield a thick oil. The oil was triturated with water (25.0 mL) and dissolved with a small amount of acetone. The acetone solution was layered with hexanes (50.0 mL) to precipitate a white solid. Upon filtration and drying *in vacuo*, the title compound was obtained as an off white solid (0.565 g, 15.8% yield). ^{1}H NMR (400 MHz, $CDCl_3$) δ 10.05 (s, 1H), 8.56 – 8.51 (m, 1H), 8.06 (d, *J* = 7.9 Hz, 1H), 7.96 (dd, *J* = 8.0, 2.0 Hz, 1H), 7.41 (s, 2H). The ^{1}H NMR spectrum matched with the reported values.[8]

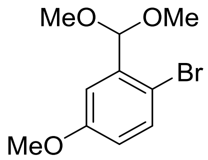

(***1-bromo-2-(dimethoxymethyl)-4-methoxybenzene***) **(78):** The compound was prepared according to a published procedure and the characterization data matched that of the reported compound.[9] To a 100 mL round bottom flask equipped with a stir bar and a reflux condenser was added 2-bromo-5-methoxybenzaldehyde (5.00 g, 23.2 mmol) dissolved in 32 mL of methanol. Concentrated sulfuric acid (0.120 mL, 2.20 mmol) was added slowly into the stirring mixture. Subsequently, trimethyl orthoformate (3.30 mL, 30.2 mmol) was added dropwise into the reaction mixture at room temperature. The solution was then heated to reflux for 1 h. After cooling to room temperature, the solution was basified to pH 11 using a concentrated NaOMe solution in methanol. The crude residue was concentrated *in vacuo*. Subsequently, DCM (30 mL) was added to the reaction slurry and the mixture was filtered through Celite. The filtrate was collected and concentrated in vacuo to furnish the title compound as a colorless liquid (5.49 g, 91% yield).). ^{1}H NMR (300 MHz, $CDCl_3$) δ 7.43 (d, *J* = 8.7 Hz, 1H), 7.16 (d, *J* = 3.1 Hz, 1H), 6.77 (dd, *J* = 8.8, 3.1 Hz, 1H), 5.51 (s, 1H), 3.81 (s, 3H), 3.40 (s, 6H). The ^{1}H NMR spectrum matched with the reported values.[9]

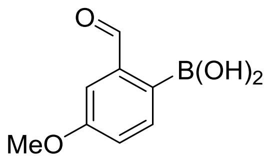

(***(2-formyl-4-methoxyphenyl)boronic acid***) **(79):** The compound was prepared according to a published procedure and the characterization data matched that of the reported compound.[10] To a 100 mL flame dried round bottom flask equipped with a stir bar was added anhydrous diethyl ether (40.1 mL) and THF (8.0 mL). After being cooled to –78 °C in a dry ice/acetone bath, 1.6 M

n-butyllithium in hexanes (13.8 mL, 22.0 mmol) was added, followed by dropwise addition of 1-bromo-2-(dimethoxymethyl)-4-methoxybenzene (5.00 g, 19.1 mmol). After stirring for 1 h at –78 °C, triethyl borate (3.50 mL, 22.0 mmol) was added slowly to the flask. The mixture was stirred for 1 h in the dry ice/acetone bath, after which the cooling bath was removed and the solution was acidified to pH 3 using 3 M aqueous HCl. The reaction mixture was extracted with Et_2O (3 x 50 mL) and the organic layer was combined and concentrated *in vacuo* to yield a thick oil. This oil layer was triturated with water (25.0 mL) and dissolved in a small amount of acetone. A white precipitate was produced with the addition of hexanes (50.0 mL). Upon filtration and drying *in vacuo*, the title compound was obtained as an off white solid (0.844 g, 24.5 % yield). ^1H NMR spectrum of this compound was a mixture of free boronic acid and benzoxaborole resulted from intramolecular condensation in 18:82 ratio respectively. Benzoxaborole ^1H NMR (300 MHz, CD_3OD) δ 7.51 (d, *J* = 8.8 Hz, 1H), 6.99 – 6.95 (m, 2H), 5.96 (s, 1H), 3.83 (s, 3H). Free boronic acid ^1H NMR (300 MHz, CD_3OD) δ 9.92 (s, 1H), 7.52 (s, 1H), 7.19 (d, *J* = 8.1 Hz, 1H), 6.89 (dd, *J* = 8.1, 2.2 Hz, 1H), 3.88 (s, 3H). The ^1H NMR spectrum matched with the reported values.[10]

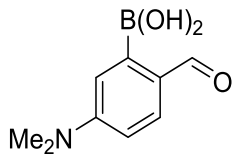

(*(5-(dimethylamino)-2-formylphenyl)boronic acid*) (80): The compound was prepared according to a published procedure and the characterization data matched that of the reported compound.[11] A flamed dried 1 L round bottom flask was charged with trimethylenediamine (4.77 mL, 36.1 mmol) in THF (76.0 mL) and then cooled to –20 °C in a NaCl-ice bath. A 2.5 M solution of n-BuLi in hexanes (14.1 mL, 35.5 mmol) was added and the mixture was stirred for 15 minutes. A solution of 4-(dimethylamino)benzaldehyde (5.00 g, 33.5 mmol) in THF (7.6 mL) was added and the solution was stirred for another 15 minutes. Then, another 2.5 M solution of n-BuLi in hexanes (40.1 mL, 100 mmol) was added slowly, after which the mixture was allowed to warm to room temperature for 15 h. The mixture was then cooled to –78 °C with a dry ice/acetone bath, and triisopropylborate (24.7 mL, 107 mmol) was added slowly. The mixture was slowly warmed to room temperature and stirred for 1 h. A 3 M aqueous solution of HCl (101 mL) was added, the flask was equipped with a reflux condenser, and the mixture was then heated to reflux for 30 min. After cooling to room temperature, the reaction mixture was extracted with diethyl ether (3 x 50 mL). The combined organic layers were treated with 1M aqueous NaOH (760 mL). The aqueous phase was collected, acidified with 3 M aqueous HCl (253 mL), and extracted with diethyl ether (3 x 50 mL). The combined organic layers were concentrated *in vacuo* until a solid white solid was observed. The solid was then triturated with diethyl ether and further dried *in vacuo* to afford the title compound as an off-white solid (0.215 g, 3.3% yield). ^1H NMR (300 MHz, CD_3OD) δ 9.59 (s, 1H), 7.70 (d, *J* = 8.7 Hz, 1H), 6.80 – 6.74 (m, 1H), 6.73 – 6.69 (m, 1H), 3.11 (s, 6H). The ^1H NMR spectrum matched with the reported values.[11]

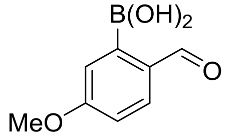

(*(2-formyl-5-methoxyphenyl)boronic acid*) **(81):** The compound was prepared according to a published procedure and the characterization data matched that of the reported compound.[11] A flamed dried 1 L round bottom flask was charged with trimethylenediamine (2.30 mL, 15.8 mmol) in THF (33.3 mL) and then cooled to –20 °C in a NaCl-ice bath. A 2.5 M solution of n-BuLi in hexanes (6.2 mL, 15.5 mmol) was added and the mixture was stirred for 15 minutes. A solution of 4-methoxybenzaldehyde (2.00 g, 14.7 mmol) in THF (3.3 mL) was added and the solution was stirred for another 15 minutes. Then, another 2.5 M solution of n-BuLi in hexanes (17.6 mL, 44.0 mmol) was added slowly, after which the mixture was allowed to warm to room temperature for 15 h. The mixture was then cooled to –78 °C with a dry ice/acetone bath, and triisopropylborate (10.8 mL, 47.0 mmol) was added slowly, and the mixture was slowly warmed to room temperature and stirred for 1 h. A 3 M aqueous HCl solution (44.0 mL) was added, the flask was equipped with a reflux condenser, and the mixture was heated to reflux for 30 min. After cooling to room temperature, the reaction mixture was extracted with diethyl ether (3 x 50 mL). The combined organic layers were treated with 1 M aqueous NaOH (333 mL). The aqueous phase was collected, acidified with 3 M aqueous HCl (111 mL), and extracted with diethyl ether (3 x 50 mL). The combined organic layers were concentrated *in vacuo* until a solid white solid was observed. The solid was then triturated with diethyl ether and dried *in vacuo* to afford the title compound as an off-white solid (0.520 g, 20% yield). ^{1}H NMR (300 MHz, CD_3OD) δ 9.81 (s, 1H), 7.88 (d, J = 8.5 Hz, 1H), 7.09 (dd, J = 8.6, 2.4 Hz, 1H), 6.97 (d, J = 2.5 Hz, 1H), 3.90 (s, 3H). The ^{1}H NMR spectrum matched with the reported values.[11]

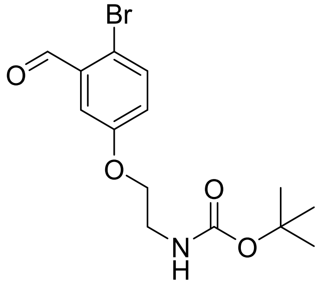

(***tert-butyl (2-(4-bromo-3-formylphenoxy)ethyl)carbamate***) **(82):** The compound was prepared according to a published procedure and the characterization data matched that of the reported compound.[12] To a 25 mL round bottom flask equipped with a stir bar was added 2-bromo-5-hydroxybenzaldehyde (1.00 g, 5.00 mmol) , N-Boc-bromoethylamine (1.30 g, 5.50 mmol), potassium carbonate (2.10 g, 15.0 mmol), and DMF (5 mL). The reaction mixture was heated to

80 °C for 15 h. The reaction mixture was diluted with water (50.0 mL) and extracted with ethyl acetate (3 x 50 mL). The combined organic layers were washed with brine, dried over Na_2SO_4, and concentrated *in vacuo*. The crude residue was purified by silica gel chromatography using 0–25% gradient to afford the title compound as a light yellow oil (461 mg, 27 % yield). ^{1}H NMR (300 MHz, $CDCl_3$) δ 10.31 (s, 1H), 7.54 (d, *J* = 8.7 Hz, 1H), 7.40 (d, *J* = 3.1 Hz, 1H), 7.03 (dd, *J* = 8.9, 3.1 Hz, 1H), 4.05 (t, *J* = 5.1 Hz, 2H), 3.55 (d, *J* = 5.7 Hz, 2H), 1.45 (s, 9H). The ^{1}H NMR spectrum matched with the reported values.[12]

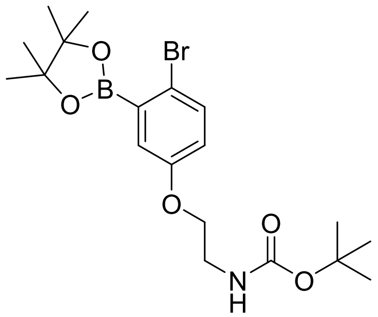

***tert*-butyl(2-(3-formyl-4-(4,4,5,5-tetramethyl-1,3,2-dioxaborolan-2-yl)phenoxy)ethyl)carbamate (83):** The compound was prepared according to a published procedure and the characterization data matched that of the reported compound.[12] To a 25 mL round bottom flask equipped with a stir bar was added compound **83** (461 mg, 1.34 mmol) , B_2pin_2 (0.850 g, 3.30 mmol), Pd(dppf)Cl_2 (54.4 mg, 74.3 μmol), potassium acetate (411 mg, 4.19 mmol) and dioxane (8.40 mL). The reaction mixture was purged with nitrogen gas for 15 min and heated to 85 °C for 1 h. The reaction mixture was diluted with water (50.0 mL) and extracted with ethyl acetate (3 x 50 mL). The combined organic layers were washed with brine, dried over Na_2SO_4, and concentrated *in vacuo*. The crude residue was purified by silica gel chromatography using 0–25% gradient to afford the title compound as a light yellow oil (326 mg, 62 % yield). ^{1}H NMR (500 MHz, $CDCl_3$) δ 10.66 (s, 1H), 7.87 (d, *J* = 8.3 Hz, 1H), 7.48 (d, *J* = 2.7 Hz, 1H), 7.11 (dd, *J* = 8.3, 2.6 Hz, 1H), 4.96 (s, 1H), 4.09 (q, *J* = 5.2 Hz, 2H), 3.56 (q, *J* = 5.5 Hz, 2H), 2.10 (s, 1H), 1.45 (s, 9H), 1.37 (s, 10H). The ^{1}H NMR spectrum matched with the reported values.[12]

Kinetic Study

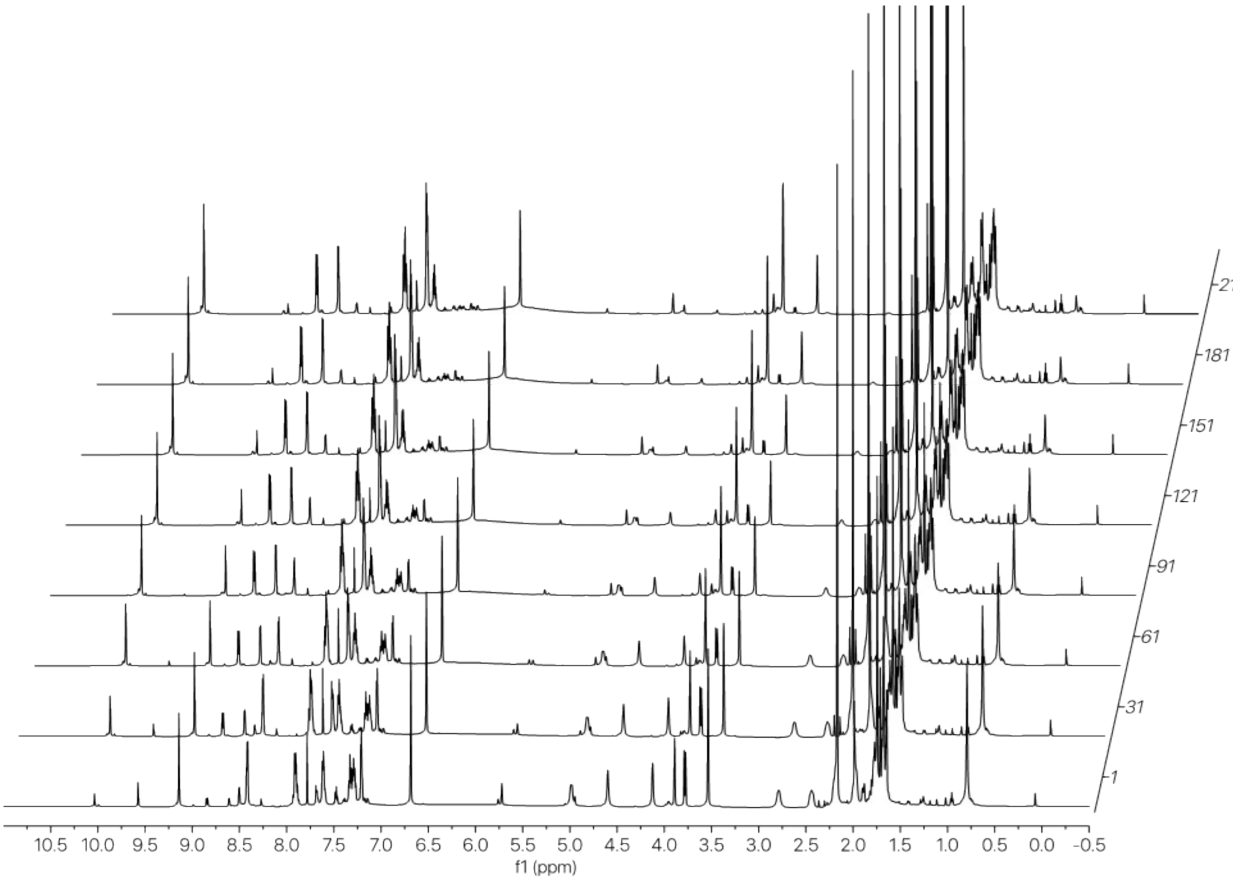

Figure S1. Stacked ^{1}H NMR spectra of the cycloreversion reaction of cycloadduct intermediate **37** at 60 °C in THF-*d8* with mesitylene as an internal standard. Note that the mixture (containing stoichiometric amount of [2.2.1]-hydrazinium **10** and starting material **7**) was heated at 40 °C for 5 hours prior to give 82% conversion of starting material **7** into either cycloadduct **37** or benzoisoquinoline **8**

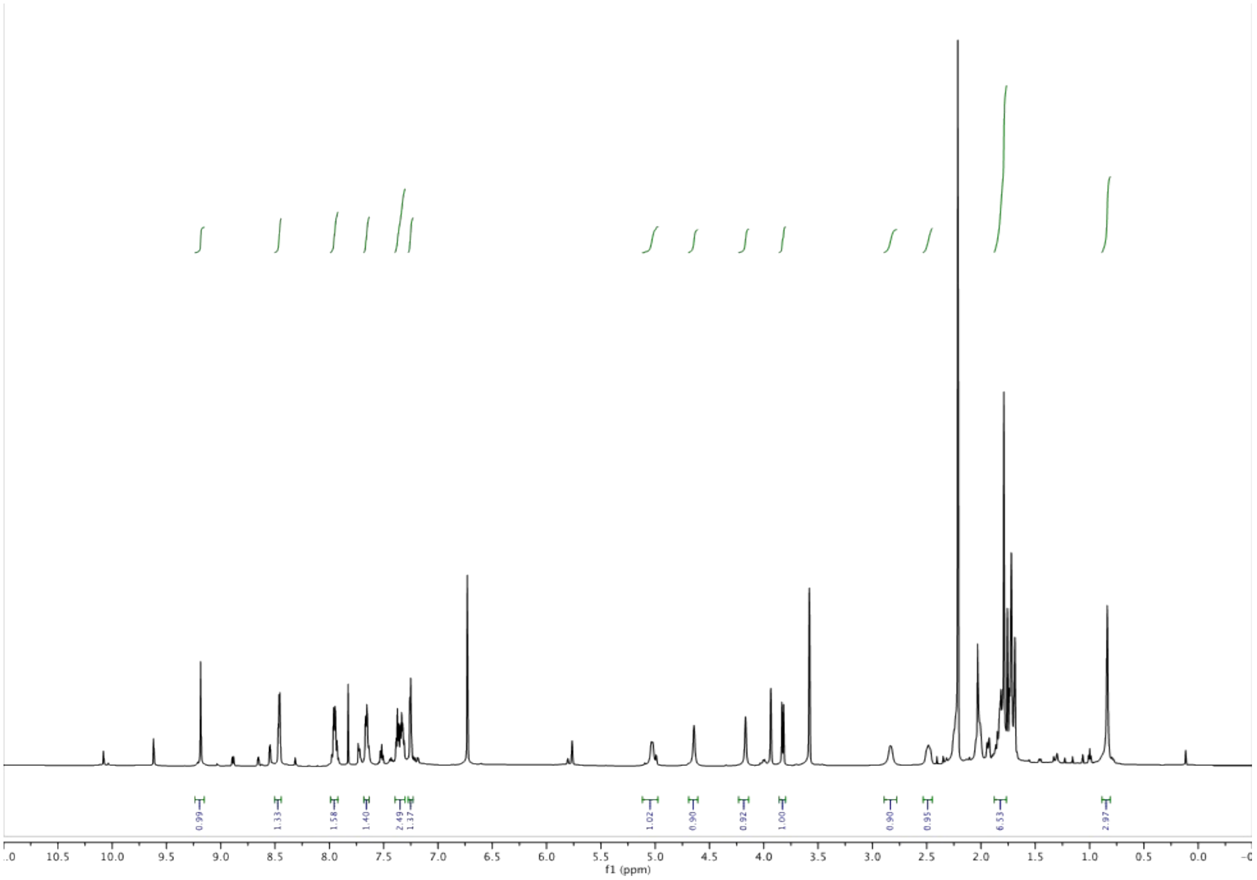

Figure S2. ^{1}H NMR spectra of the cycloreversion reaction of cycloadduct intermediate **37** at 60 °C in THF-*d8* with mesitylene as an internal standard at t = 0. Note that the mixture (containing stoichiometric amount of [2.2.1]-hydrazinium **10** and starting material **7**) was heated at 40 °C for 5 hours prior to give 82% conversion of starting material **7** into either cycloadduct **37** or benzoisoquinoline **8**. Integrated peaks are speculated to belong to cycloadduct intermediate **37**, note the two singlets at 4.64 and 4.16 ppm belonging to the bridgehead C-H protons, and the doublet at 3.83ppm (J = 9.6 Hz) belonging to pyrazolidium C-H proton adjacent to the hydrazine moiety.

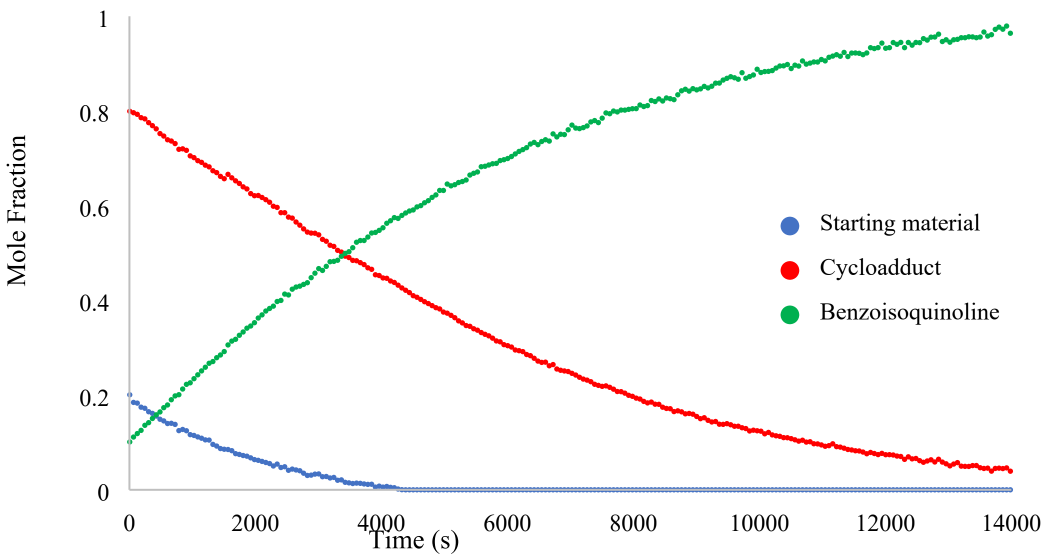

Figure S3. Plot of mole fraction of starting material **7**, cycloadduct intermediate **37**, and benzoisoquinoline product **8** versus time at 60 °C in THF-*d8* as determined by ^{1}H NMR spectroscopy versus mesitylene as an internal standard. The mixture (containing stoichiometric amount of [2.2.1]-hydrazinium **10** and starting material **7**) was heated at 40 °C for 5 hours prior to give 82% conversion of starting material **7** into either cycloadduct **37** or benzoisoquinoline **8**. The conversion of starting material **7** and cycloadduct **37** was determined based on the diminishment of representative singlets at δ 9.58 ppm and δ 9.14 ppm, respectively. The formation of benzoisoquinoline **8** was determined based on the appearance of a representative singlet at δ 10.04 ppm.

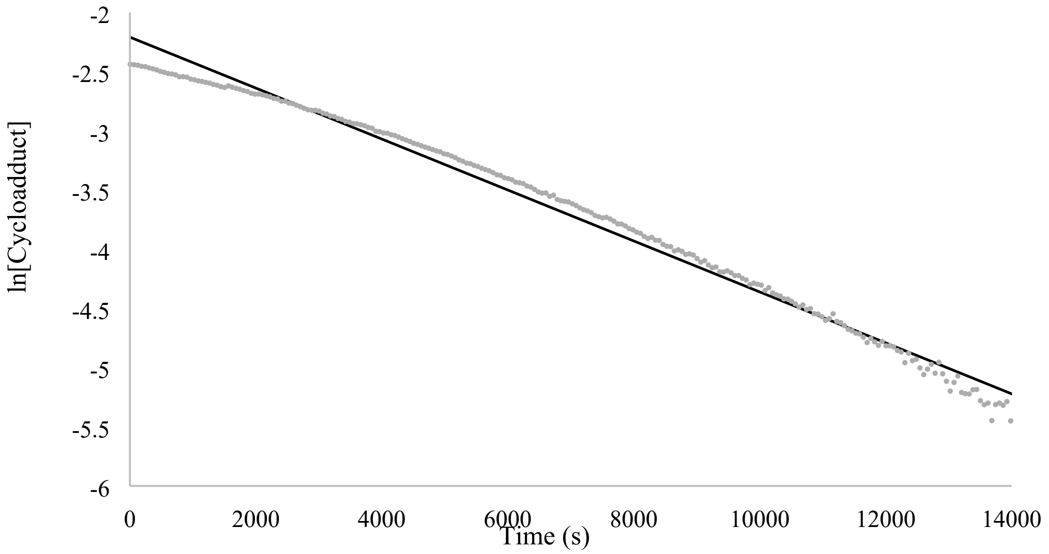

Figure S4. Plot of the natural log of the concentration of cycloadduct **37** versus time at 60 °C in THF-d_8 as determined by ^{1}H NMR spectroscopy versus mesitylene as an internal standard. The rate constant for cycloreversion $k_{CR} = 2.14 \times 10^{-4}\ s^{-1}$ was obtained through a first-order fit to $f(x) = (a-1)e^{-bx}$.

Computational Data

All DFT calculations were performed with the Gaussian 09 program package[13]. The geometry optimization of all the minima and transition states involved was carried out at the M06-2X level of theory[14,15] with the 6-31G(d) basis set[16]. The vibrational frequencies were computed at the same level to check whether each optimized structure was an energy minimum or a transition state and to evaluate its zero-point vibrational energy (ZPVE) and thermal corrections at 298 K. Solvent effects in toluene were computed at the M06-2X/6-311+G(d,p) level using the gas-phase optimized structures. Solvation energies were evaluated by a self-consistent reaction field (SCRF) using the PCM model.

Table 1. Geometric coordinates and thermally corrected M06-2X energies for **10**.

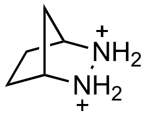

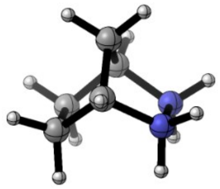

G_{THF} = -306.615451985 Hartree

Atom	X	Y	Z	Atom	X	Y	Z
N	1.1968860	0.7386940	-0.4543960	H	-2.1764150	-1.1873040	-0.0564560
N	1.1968880	-0.7386940	-0.4543960	H	0.0516170	2.1589860	0.7026150
C	-1.2716960	0.7800090	-0.5159960	H	0.8353630	-0.0000010	2.0791120
C	-1.2716960	-0.7800080	-0.5159960	H	-0.9392850	-0.0000020	2.0288580
C	-0.0823890	1.1243410	0.3817210	H	0.0516170	-2.1589870	0.7026130
C	-0.0823900	-1.1243410	0.3817200	H	2.0678710	-1.0872490	-0.0189560
C	-0.0341380	-0.0000010	1.4137650	H	1.1636310	-1.0986440	-1.4234540
H	-2.1764150	1.1873040	-0.0564550	H	2.0678710	1.0872510	-0.0189580
H	-1.2060250	1.2276020	-1.5126020	H	1.1636280	1.0986440	-1.4234540
H	-1.2060250	-1.2276000	-1.5126030				

Table 2. Geometric coordinates and thermally corrected M06-2X energies for 7.

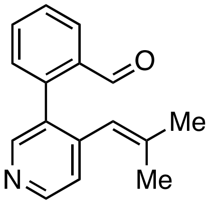

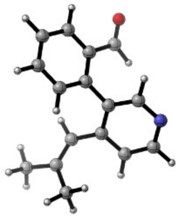

G_{THF} = -748.374320935 Hartree

Atom	X	Y	Z	Atom	X	Y	Z
O	3.7800530	1.1793320	1.5644830	C	-1.7002290	2.9761880	-0.3353730
N	-0.4660540	3.1857180	-0.7976230	C	0.3629780	2.1450530	-0.7757240
C	-2.8616490	-1.2837780	0.5040740	H	-2.3206780	-2.9108890	1.8260960
C	2.6683850	1.0259020	1.1119660	H	-3.3062000	-3.3883110	0.4264510
C	-1.6901330	-0.6593400	0.6966220	H	-4.0561930	-2.5414700	1.7739200
C	-3.1404720	-2.6026530	1.1733110	H	-4.8072080	-0.4025500	0.2057780
C	-3.9680080	-0.7858070	-0.3883290	H	-4.3619730	-1.6152980	-0.9862790
C	2.3041890	-0.1148770	0.2250990	H	-3.6406250	0.0036490	-1.0671310
C	3.2513780	-1.1323100	0.0652580	H	-0.9385130	-1.1623660	1.3034970
C	1.0403010	-0.2228370	-0.3815170	H	4.2138690	-1.0092880	0.5520190
C	0.0303580	0.8668330	-0.3127380	H	1.4500680	-3.2672960	-1.8525230
C	-1.2810960	0.6500810	0.1526050	H	3.6921510	-3.0489080	-0.7996790
C	2.9538620	-2.2625730	-0.6796380	H	-0.2239120	-1.4652550	-1.5922380
C	1.6941810	-2.3851000	-1.2685970	H	-2.3663430	3.8359790	-0.3409990
C	0.7502090	-1.3767470	-1.1203770	H	-3.1491610	1.6559060	0.5447800
C	-2.1449190	1.7503900	0.1451130	H	1.3659750	2.3218440	-1.1638000

H	1.8546750	1.7290090	1.3670760

Table 3. Geometric coordinates and thermally corrected M06-2X energies for $\mathbf{H_3O^+}$.

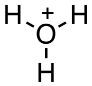

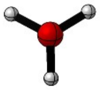

G_{THF} = -76.7850672652 Hartree

Atom	X	Y	Z	Atom	X	Y	Z
O	0.0000000	-0.0000010	0.0000110	H	0.2382740	0.9525620	-0.0000290
H	-0.9440930	-0.2699310	-0.0000290	H	0.7058200	-0.6826250	-0.0000290

Table 4. Geometric coordinates and thermally corrected M06-2X energies for ***Z*-35**.

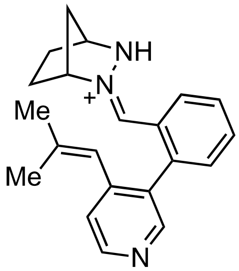

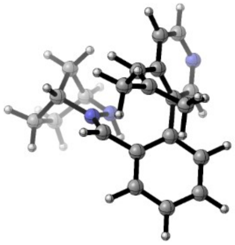

G_{THF} = -978.22542247 Hartree

Atom	X	Y	Z	Atom	X	Y	Z
N	-1.8165140	-0.3169980	-0.8205130	C	-0.1067020	2.9131560	0.3268470
N	-1.4206470	-0.8350860	0.4083600	C	-0.5078260	3.4561250	-0.8882250
N	-0.2932180	2.8619450	-2.0649410	C	0.3251810	1.6881660	-2.0361910
C	-4.0313950	-1.3712950	-0.4875790	H	-5.1101700	-1.2018000	-0.5149200
C	-3.5546810	-1.8364530	0.9211780	H	-3.7899280	-2.0902910	-1.2745190
C	-3.2654470	-0.0470640	-0.6688990	H	-3.0682950	-2.8145200	0.9166640
C	-2.5830090	-0.7134480	1.3338720	H	-4.3765570	-1.8734860	1.6400470
C	-3.2444050	0.5391400	0.7471250	H	-3.5912940	0.6087720	-1.4744830
C	2.1605220	0.6589970	2.0411490	H	-2.6341350	1.4412800	0.8276470
C	-0.2555760	-1.3048170	0.6934850	H	-4.2413870	0.6998760	1.1601440
C	0.9588150	1.1381500	1.6809100	H	-2.2362350	-0.7005030	2.3661390
C	2.3927370	0.1510950	3.4378340	H	1.4867250	0.1869110	4.0480340
C	3.3584810	0.6014660	1.1356080	H	3.1676560	0.7423540	3.9378600
C	0.8626000	-1.4209260	-0.2453210	H	2.7587630	-0.8827940	3.4118420
C	1.4989670	-2.6669300	-0.3209970	H	3.2408480	1.2198810	0.2439090
C	1.3186750	-0.3296610	-1.0154150	H	3.5553290	-0.4314080	0.8180280
C	0.7551910	1.0343590	-0.8724090	H	4.2463280	0.9423050	1.6782270
C	0.5564810	1.6812660	0.3628070	H	-0.1590300	-1.6959630	1.7039010
C	2.5365490	-2.8697340	-1.2214020	H	0.1931960	1.2187220	2.4552010
C	2.9555750	-1.8155440	-2.0309420	H	1.1587620	-3.4839270	0.3096980
C	2.3621140	-0.5620680	-1.9161690	H	3.7675580	-1.9619280	-2.7356390

H	3.0150580	-3.8404220	-1.2888640	H	-0.2818440	3.4583250	1.2499040
H	2.7362950	0.2708140	-2.5046120	H	0.4693140	1.2003920	-3.0001300
H	-1.0144300	4.4174550	-0.9212680	H	-1.5369500	-0.9429600	-1.5767350

Table 5. Geometric coordinates and thermally corrected M06-2X energies for ***E*-35**.

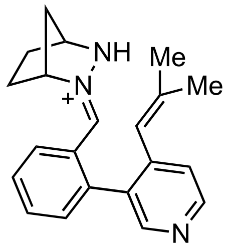

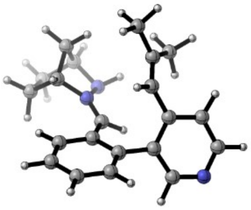

G_{THF} = -978.220419615 Hartree

Atom	X	Y	Z	Atom	X	Y	Z
N	2.1679600	-0.9790890	-1.3985680	H	5.3863630	0.0570700	-1.3264600
N	1.6506510	0.0671310	-0.5832540	H	4.1028170	0.3190650	-2.5189180
N	-4.1144460	-0.5917960	-1.7381760	H	3.2417040	2.0863490	-1.1360400
C	4.3078130	0.1626660	-1.4589770	H	4.4934250	1.7568230	0.0709850
C	3.7283010	1.2966390	-0.5588920	H	3.9872270	-2.0438750	-1.2500730
C	3.5775840	-1.0847090	-0.9367860	H	2.8449710	-1.5375080	1.1032080
C	2.7323540	0.5274280	0.3334100	H	4.4098730	-0.6634250	1.0714760
C	3.4525840	-0.8048830	0.5660940	H	2.3011110	1.0432380	1.1893870
C	-0.2665900	-2.2159520	1.4540890	H	0.3808060	-1.5103170	3.3977850
C	0.4393920	0.4773900	-0.7288150	H	1.5772860	-2.5261330	2.5565620
C	-1.1266250	-1.1959850	1.2981520	H	0.1577210	-3.2612510	3.2717210
C	0.5074000	-2.3702080	2.7362340	H	-0.4271540	-3.0459950	-0.5565640
C	-0.0292400	-3.2973700	0.4310940	H	-0.4905760	-4.2427630	0.7415320
C	-0.2171520	1.5760220	-0.0404760	H	1.0455970	-3.5085270	0.3479780
C	0.4640760	2.7426320	0.3447010	H	-0.1390640	-0.0861890	-1.4601510
C	-1.6222080	1.4910000	0.1074600	H	-1.1705240	-0.4437480	2.0857230
C	-2.3726280	0.2833080	-0.3147330	H	1.5297730	2.8404770	0.1677420
C	-2.0988410	-1.0005510	0.2003450	H	-2.1652570	4.5639950	1.4638600
C	-0.2334750	3.8119730	0.8851800	H	0.2958420	4.7152620	1.1674450
C	-1.6168860	3.7280410	1.0420960	H	-3.3775510	2.5151160	0.7837300
C	-2.3018060	2.5806930	0.6533960	H	-4.4618560	-2.6183020	-1.6337920
C	-2.8732280	-2.0550950	-0.2916160	H	-2.7489030	-3.0566180	0.1063250
C	-3.8505490	-1.8052600	-1.2507160	H	-3.6108580	1.4028040	-1.6769610
C	-3.3902050	0.4182760	-1.2669150	H	1.6476540	-1.8255990	-1.1524740

Table 6. Geometric coordinates and thermally corrected M06-2X energies for **36**.

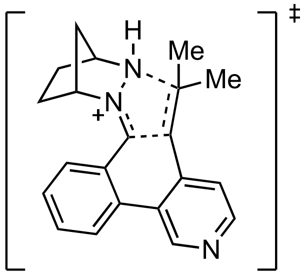

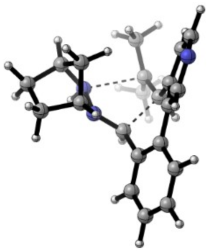

G_{THF} = -978.191924052 Hartree

Atom	X	Y	Z
N	2.1771940	-1.0591300	0.3157540
N	0.8307710	-1.2950640	0.0140950
N	-1.3085110	3.1496620	-1.7953400
C	2.7927950	-2.3206470	-1.7090050
C	1.2747730	-2.4854820	-2.0251790
C	2.8374030	-0.9434260	-1.0156400
C	0.6747820	-1.1847690	-1.4540090
C	1.7655330	-0.1580100	-1.7823410
C	1.6368060	0.8520320	1.8705990
C	-0.0389710	-0.9795150	0.9987430
C	0.2819870	0.8718020	1.5024800
C	2.7572010	1.6108520	1.2220080
C	1.9939300	0.2395660	3.1927730
C	-1.4967920	-1.0569820	0.7116340
C	-2.1989560	-2.1958830	1.1053070
C	-2.1461650	0.0055310	0.0629130
C	-1.4111080	1.2307000	-0.3250550
C	-0.2826290	1.6893300	0.3841640
C	-3.5597560	-2.3024500	0.8427000
C	-4.2187240	-1.2521380	0.2080440
C	-3.5225590	-0.1086550	-0.1673280
C	0.2377550	2.9331670	0.0253330
C	-0.3057180	3.6217720	-1.0564010
C	-1.8435090	1.9926300	-1.4190560
H	3.3996580	-2.3083700	-2.6174830
H	3.1827780	-3.1128010	-1.0637630
H	0.8303670	-3.3678320	-1.5617300
H	1.0896660	-2.5345010	-3.1008080
H	3.8204100	-0.4864670	-0.8983000
H	1.5582920	0.8401350	-1.3935530
H	1.9770370	-0.1076850	-2.8520010
H	-0.3552400	-0.9614390	-1.7226220
H	3.7057150	1.0896690	1.3738860
H	2.8495580	2.5890060	1.7146260
H	2.6097450	1.7882180	0.1570770
H	2.2647130	1.0420560	3.8915920
H	2.8745480	-0.4049520	3.1060340
H	1.1717050	-0.3212160	3.6442520
H	0.2581990	-1.3991720	1.9607320
H	-0.4049270	0.7413810	2.3434600
H	-1.6749400	-3.0012000	1.6129170
H	-5.2854240	-1.3145680	0.0203740
H	-4.1038890	-3.1904360	1.1452390
H	-4.0609490	0.7194760	-0.6160990
H	0.0914760	4.5915310	-1.3445240
H	1.0450110	3.3856380	0.5878210
H	-2.6635670	1.6302340	-2.0353430
H	2.5554110	-1.8315310	0.8705480

Table 7. Geometric coordinates and thermally corrected M06-2X energies for **37a**.

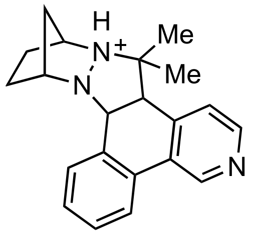

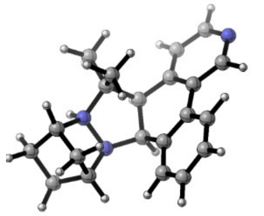

G_{THF} = -978.243109483 Hartree

Atom	X	Y	Z	Atom	X	Y	Z
N	1.8471850	-1.1977010	0.0462860	H	5.0840420	-0.7405110	0.9259610
N	1.7429150	-0.2142800	-1.0484860	H	4.3376790	-2.0622890	0.0364830
N	-4.5801000	-0.9232520	-0.0416320	H	4.1290260	-0.6331480	-1.9166060
C	4.2447740	-0.9980970	0.2763640	H	5.0171220	0.6066560	-1.0223500
C	4.1673480	-0.0775750	-0.9777730	H	2.7851230	-1.0800530	1.9892640
C	2.9302440	-0.6658550	0.9928120	H	1.8939650	1.2989750	1.1555690
C	2.8586630	0.7095480	-0.7502150	H	3.6774640	1.3594920	1.1962380
C	2.8093080	0.8439860	0.7788590	H	2.7821330	1.6035220	-1.3600370
C	0.4445540	-1.5558860	0.5366640	H	0.6707510	-3.6317440	-0.1075700
C	0.3021340	0.1620590	-1.2053480	H	-0.6087040	-3.3432950	1.0794660
C	-0.3566650	-1.1450700	-0.7300440	H	1.0761270	-3.3432450	1.6067690
C	0.4062280	-3.0592840	0.7886500	H	0.1611040	0.2731980	1.7337260
C	0.0741720	-0.8095670	1.8143830	H	0.6745760	-1.1612210	2.6575790
C	-0.2822250	1.4103750	-0.5365200	H	-0.9700760	-1.0384840	2.0421660
C	0.3779800	2.6410990	-0.5672810	H	0.1630160	0.2620300	-2.2873850
C	-1.5810670	1.3614300	0.0188860	H	-0.1546920	-1.9096700	-1.4902530
C	-2.4120370	0.1429940	-0.1102220	H	1.3225060	2.7440170	-1.0846590
C	-1.8360830	-1.0686370	-0.5026920	H	-1.8068250	4.5621680	1.1763530
C	-0.1559930	3.7744020	0.0376120	H	0.3890740	4.7116020	0.0024840
C	-1.3863610	3.6947860	0.6789310	H	-3.0608680	2.4546990	1.1384270
C	-2.0928240	2.5006640	0.6526990	H	-4.6657780	-2.9314890	-0.4940900
C	-2.6434150	-2.1913740	-0.6515700	H	-2.2254250	-3.1401860	-0.9771040
C	-4.0063940	-2.0734110	-0.3958090	H	-4.3222420	1.0697500	0.3233470
C	-3.8019800	0.1475870	0.0800550	H	2.2187370	-2.0491800	-0.3875980

Table 8. Geometric coordinates and thermally corrected M06-2X energies for **37b**.

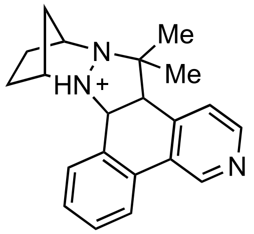

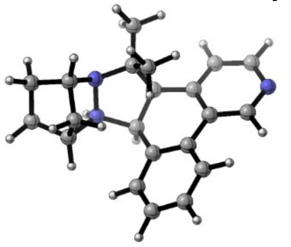

G_{THF} = -978.24118955 Hartree

Atom	X	Y	Z	Atom	X	Y	Z
N	1.8231700	-1.2968190	-0.0209050	H	4.9918600	-0.8882700	1.0742720
N	1.7714600	-0.1813500	-0.9865630	H	4.3017360	-2.0549730	-0.0686200
N	-4.5423600	-0.8305070	0.1273260	H	4.2658650	-0.3330770	-1.7662330
C	4.1958000	-1.0511750	0.3441740	H	5.0053860	0.7715800	-0.6173010
C	4.1886150	0.0578410	-0.7462430	H	2.6589060	-1.3996670	1.9207650
C	2.8161850	-0.8583930	0.9896290	H	1.7105790	1.0466080	1.3684340
C	2.8554700	0.7844920	-0.4958060	H	3.4838840	1.1668590	1.5468240
C	2.6703030	0.6717890	1.0120820	H	2.7499930	1.7496120	-0.9757200
C	0.4358600	-1.6527940	0.3877780	H	0.7053600	-3.6333290	-0.4674080
C	0.2903520	0.1903920	-1.2554820	H	-0.6489090	-3.5036040	0.6883020
C	-0.3765610	-1.1320430	-0.8367330	H	1.0170830	-3.5414070	1.2753170
C	0.3722480	-3.1786060	0.4688210	H	-0.0566430	0.0181000	1.7567470
C	-0.0243730	-1.0718970	1.7314500	H	0.6198450	-1.4236500	2.5412900
C	-0.2512820	1.4340680	-0.5808360	H	-1.0317870	-1.4366390	1.9462400
C	0.3794290	2.6720610	-0.7374820	H	0.2506070	0.3263160	-2.3413380
C	-1.4945620	1.3819050	0.0849500	H	-0.2259270	-1.8576700	-1.6448150
C	-2.3544530	0.1816710	-0.0279030	H	1.2329920	2.7704640	-1.3986610
C	-1.8434170	-1.0113960	-0.5496750	H	-1.6089350	4.5973040	1.2156670
C	-0.0953260	3.8118480	-0.1007790	H	0.4185710	4.7583190	-0.2279550
C	-1.2386280	3.7249950	0.6878280	H	-2.8590020	2.4926360	1.3279330
C	-1.9406580	2.5295440	0.7535290	H	-4.7243780	-2.7907510	-0.4783910
C	-2.6955060	-2.0952560	-0.7258730	H	-2.3301390	-3.0286480	-1.1445080
C	-4.0327300	-1.9608080	-0.3619630	H	-4.1955950	1.1187280	0.6265110
C	-3.7237740	0.2070900	0.2708960	H	2.1048010	-0.5760720	-1.8705270

Table 9. Geometric coordinates and thermally corrected M06-2X energies for **38**.

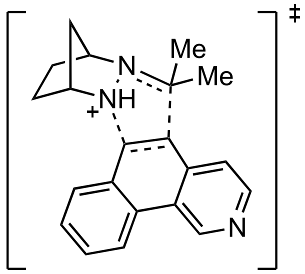

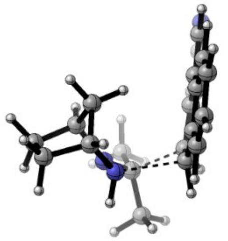

G_{THF} = -978.208568758 Hartree

Atom	X	Y	Z	Atom	X	Y	Z
N	2.1003060	0.1759380	0.0544690	H	3.3960710	-0.6441470	-2.9376150
N	1.7161760	-1.0818490	0.5122460	H	4.2055310	-0.2536930	-1.4132620
N	-2.7858610	2.7075710	-1.3977160	H	3.6913970	-2.4718640	-0.6048710
C	3.2955700	-0.6685590	-1.8499670	H	2.9210000	-2.8291720	-2.1501750
C	2.9654480	-2.1033060	-1.3348960	H	1.9355510	1.1225620	-1.8539410
C	2.0433570	0.1298430	-1.4249390	H	-0.0589200	-0.5465320	-1.3496340
C	1.5660180	-1.9134630	-0.7125140	H	0.8985460	-1.2462090	-2.6878180
C	0.9258780	-0.8908230	-1.6559900	H	0.9966380	-2.8122890	-0.4725940
C	1.8508970	1.2203330	0.8990860	H	2.2701340	0.1304730	2.7620420
C	-0.1697150	-0.3820080	1.6941630	H	2.2193170	1.8909990	2.9239900
C	-0.0509910	1.0071900	1.4304960	H	3.5968890	1.1194410	2.1260090
C	2.5113300	1.0717840	2.2645160	H	1.4042930	2.7739110	-0.5621420
C	2.0518100	2.5901910	0.2963780	H	3.0970660	2.6965590	-0.0143710
C	-1.1532980	-1.2099560	1.0504130	H	1.8530690	3.3551890	1.0493200
C	-1.2600480	-2.5700850	1.4055510	H	0.2899390	-0.7881160	2.5907020
C	-2.0173670	-0.6571280	0.0759010	H	0.1314890	1.6315480	2.3047320
C	-1.9179140	0.7740540	-0.2299000	H	-0.5955900	-2.9677000	2.1692780
C	-1.0028160	1.5893860	0.4661170	H	-3.7889190	-3.4728960	-0.6512760
C	-2.1955720	-3.3834210	0.8009390	H	-2.2795180	-4.4281130	1.0799220
C	-3.0464450	-2.8417890	-0.1739590	H	-3.6494470	-1.1216770	-1.2705630
C	-2.9618790	-1.5060740	-0.5268430	H	-2.0369970	4.5496650	-0.8448330
C	-1.0730810	2.9734490	0.2576690	H	-0.4457280	3.6506140	0.8284190
C	-1.9669060	3.4796280	-0.6697200	H	-3.4614160	0.8212290	-1.7554930
C	-2.7619260	1.4063680	-1.1650360	H	2.4011550	-1.4650850	1.1663840

Table 10. Geometric coordinates and thermally corrected M06-2X energies for **39**.

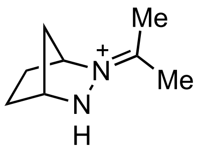

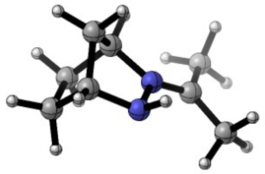

G_{THF} = -422.921477266 Hartree

Atom	X	Y	Z
N	0.4434510	-0.1062370	0.1966510
N	-0.2435470	-1.3371650	0.0505660
C	-1.2939370	1.1872050	-0.8654320
C	-2.0544720	-0.1610640	-1.0518290
C	-0.5678260	0.9662110	0.4755740
C	-1.6691430	-0.9414910	0.2157640
C	-1.5849400	0.1573430	1.2844250
C	1.7087770	0.0147150	-0.0219780
C	2.5016440	-1.2056100	-0.3518560
C	2.3574590	1.3595030	0.1028090
H	-1.9748040	2.0358310	-0.7671180
H	-0.5970090	1.4055770	-1.6779960
H	-1.7488160	-0.6995180	-1.9499960
H	-3.1348750	-0.0098540	-1.0915580
H	-0.0958960	1.8257490	0.9460410
H	-1.2099930	-0.1867580	2.2518510
H	-2.5181530	0.7074140	1.4194730
H	-2.2647360	-1.8267680	0.4326500
H	0.0565440	-1.9542020	0.8093970
H	2.7767740	-1.7303080	0.5727400
H	3.4237840	-0.9345800	-0.8653760
H	1.9094940	-1.8909030	-0.9644300
H	1.7561810	2.1449120	-0.3615820
H	3.3391790	1.3425760	-0.3695980
H	2.4976270	1.6137740	1.1601260

Table 11. Geometric coordinates and thermally corrected M06-2X energies for **8**.

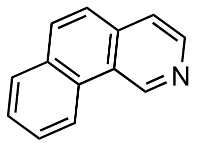

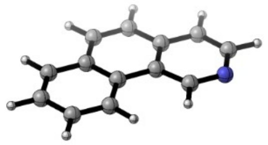

G_{THF} = -555.352106284 Hartree

Atom	X	Y	Z
N	-2.8391440	-1.5706920	-0.0000020
C	0.6550890	2.0932840	-0.0000010
C	-0.6983710	2.0979580	0.0000010
C	1.4024090	0.8640640	-0.0000010
C	2.8128980	0.8849430	-0.0000030
C	0.7179830	-0.3766030	0.0000010
C	-0.7357650	-0.3687610	0.0000000
C	-1.4304510	0.8614450	0.0000010
C	3.5365140	-0.2862000	0.0000000
C	2.8615270	-1.5190720	0.0000020
C	1.4833730	-1.5624200	0.0000030
C	-2.8383950	0.8288530	0.0000020
C	-3.4844520	-0.3859070	0.0000000
C	-1.5204620	-1.5442460	-0.0000040
H	1.2108490	3.0274210	-0.0000020
H	-1.2555050	3.0305000	0.0000030
H	3.3191910	1.8467280	-0.0000040
H	3.4279740	-2.4449450	0.0000050
H	4.6214600	-0.2601990	-0.0000010
H	0.9900570	-2.5278020	0.0000060
H	-4.5700720	-0.4387160	0.0000030
H	-3.4022800	1.7570050	0.0000040
H	-1.0390490	-2.5191820	-0.0000070

Table 12. Geometric coordinates and thermally corrected M06-2X energies for **9**.

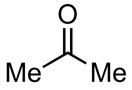

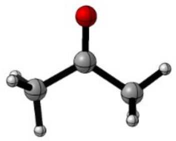

G_{THF} = -193.070934767 Hartree

Atom	X	Y	Z
O	-0.0217140	1.3876680	0.0000440
C	0.0055140	0.1788740	-0.0001680
C	-1.2822700	-0.6293650	0.0000210
C	1.3049740	-0.5959450	0.0000230
H	-1.8710110	-0.3577720	-0.8805910
H	-1.8707680	-0.3577960	0.8808060
H	-1.1078740	-1.7072780	-0.0000110
H	1.3522040	-1.2466040	0.8803540
H	2.1494200	0.0933230	0.0001320
H	1.3524270	-1.2466000	-0.8802960

References

[1] C. C. McAtee, P. S. Riehl, C. S. Schindler, *J. Am. Chem. Soc.* **2017**, *139*, 2960–2963.
[2] M. A. Düfert, K. L. Billingsley, S. L. Buchwald, *J. Am. Chem. Soc.* **2013**, *135*, 12877–12885.
[3] S. He, P. Li, X. Dai, H. Liu, Z. Lai, D. Xiao, C. C. McComas, C. Du, Y. Liu, J. Yin, Q. Dang, N. Zorn, X. Peng, R. P. Nargund, A. Palani, *Tetrahedron Lett.* **2017**, *58*, 1373–1375.
[4] X. Jiang, J. Yang, F. Zhang, P. Yu, P. Yi, Y. Sun, Y. Wang, *Adv. Synth. Catal.* **2016**, *358*, 2678–2683.
[5] S. Staniland, R. W. Adams, J. J. W. McDouall, I. Maffucci, A. Contini, D. M. Grainger, N. J. Turner, J. Clayden, *Angew. Chem.* **2016**, *128*, 10913–10917.
[6] D. Ding, Y. Zhao, Q. Meng, D. Xie, B. Nare, D. Chen, C. J. Bacchi, N. Yarlett, Y.-K. Zhang, V. Hernandez, Y. Xia, Y. Freund, M. Abdulla, K.-H. Ang, J. Ratnam, J. H. McKerrow, R. T. Jacobs, H. Zhou, J. J. Plattner, *ACS Med. Chem. Lett.* **2010**, *1*, 165–169.
[7] K. Kowalska, A. Adamczyk-Woźniak, P. Gajowiec, B. Gierczyk, E. Kaczorowska, Ł. Popenda, G. Schroeder, A. Sikorski, A. Sporzyński, *J. Fluor. Chem.* **2016**, *187*, 1–8.
[8] A. Adamczyk-Woźniak, J. T. Gozdalik, D. Wieczorek, I. D. Madura, E. Kaczorowska, E. Brzezińska, A. Sporzyński, J. Lipok, *Molecules* **2020**, *25*, 799.
[9] Y. Hoshimoto, C. Nishimura, Y. Sasaoka, R. Kumar, S. Ogoshi, *Bull. Chem. Soc. Jpn.* **2020**, *93*, 182–186.
[10] M. Koepf, F. Melin, J. Jaillard, J. Weiss, *Tetrahedron Lett.* **2005**, *46*, 139–142.
[11] Z. el abidine Chamas, E. Marchi, A. Modelli, Y. Fort, P. Ceroni, V. Mamane, *Eur. J. Org. Chem.* **2013**, *2013*, 2316–2324.
[12] S. Cambray, A. Bandyopadhyay, J. Gao, *Chem. Commun.* **2017**, *53*, 12532–12535.
[13] M. J. Frisch, G. W. Trucks, H. B. Schlegel, G. E. Scuseria, M. A. Robb, J. R. Cheeseman, G. Scalmani, V. Barone, G. A. Petersson, H. Nakatsuji, X. Li, M. Caricato, A. Marenich, J. Bloino, B. G. Janesko, R. Gomperts, B. Mennucci, H. P. Hratchian, J. V. Ortiz, A. F. Izmaylov, J. L. Sonnenberg, D. Williams-Young, F. Ding, F. Lipparini, F. Egidi, J. Goings, B. Peng, A. Petrone, T. Henderson, D. Ranasinghe, V. G. Zakrzewski, J. Gao, N. Rega, G. Zheng, W. Liang, M. Hada, M. Ehara, K. Toyota, R. Fukuda, J. Hasegawa, M. Ishida, T. Nakajima, Y. Honda, O. Kitao, H. Nakai, T. Vreven, K. Throssell, J. A. Montgomery, Jr., J. E. Peralta, F. Ogliaro, M. Bearpark, J. J. Heyd, E. Brothers, K. N. Kudin, V. N. Staroverov, T. Keith, R. Kobayashi, J. Normand, K. Raghavachari, A. Rendell, J. C. Burant, S. S. Iyengar, J. Tomasi, M. Cossi, J. M. Millam, M. Klene, C. Adamo, R. Cammi, J. W. Ochterski, R. L. Martin, K. Morokuma, O. Farkas, J. B. Foresman, and D. J. Fox **2009**.
[14] Y. Zhao, D. G. Truhlar, *Theor. Chem. Acc.* **2008**, *120*, 215–241.
[15] Y. Zhao, D. G. Truhlar, *Acc. Chem. Res.* **2008**, *41*, 157–167.
[16] W. J. Hehre, L. Radom, P. von R. Schleyer, J. Pople, *Ab Initio Molecular Orbital Theory*, Wiley: New York, **1986**.

NMR Spectra

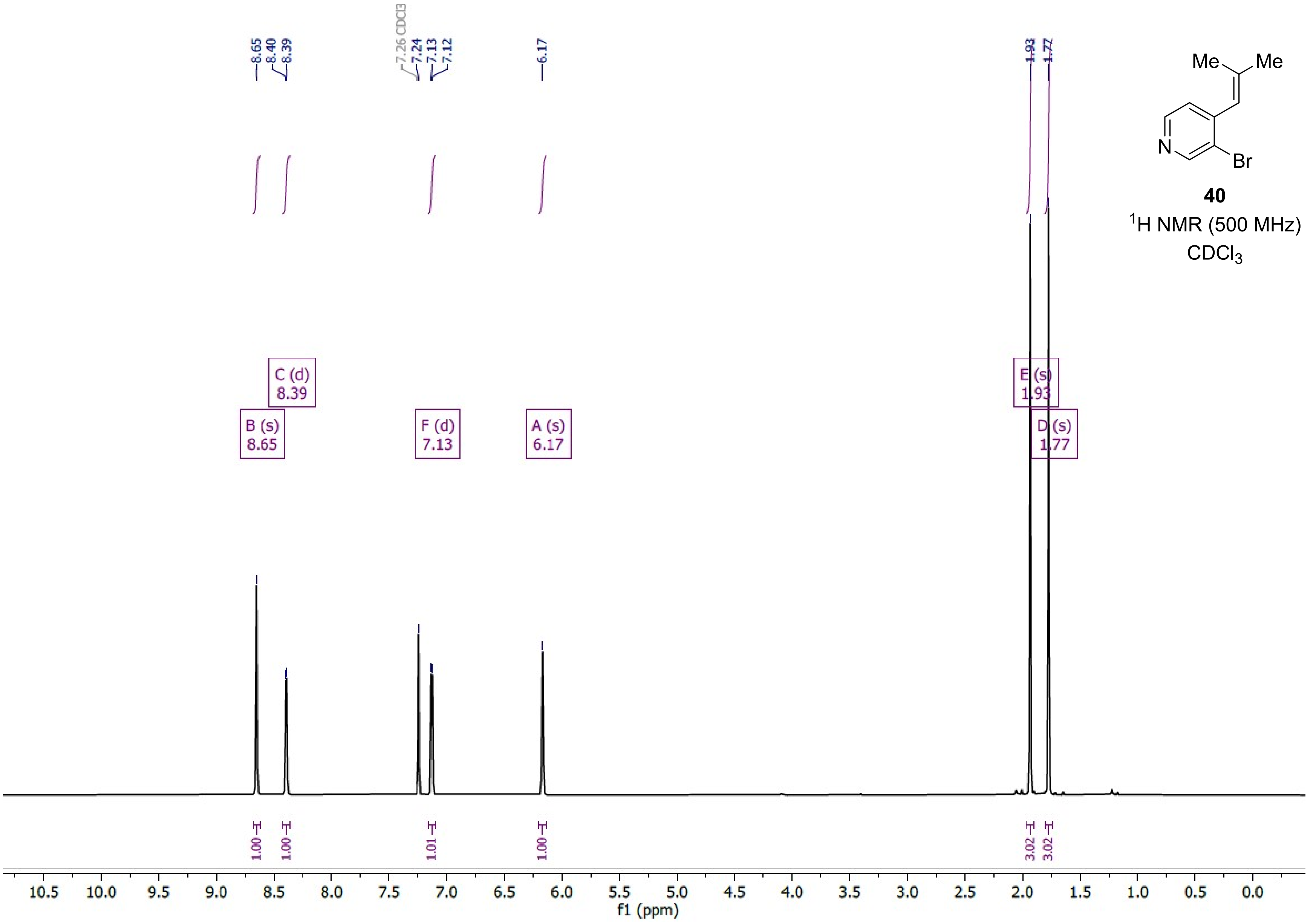

Me
Me
N
Br
40
¹H NMR (500 MHz)
CDCl3
8.65
8.40
8.39
7.26 CDCl3
7.24
7.13
7.12
6.17
1.93
1.77
C (d) 8.39
B (s) 8.65
F (d) 7.13
A (s) 6.17
E (s) 1.93
D (s) 1.77
1.00
1.00
1.01
1.00
3.02
3.02
f1 (ppm)

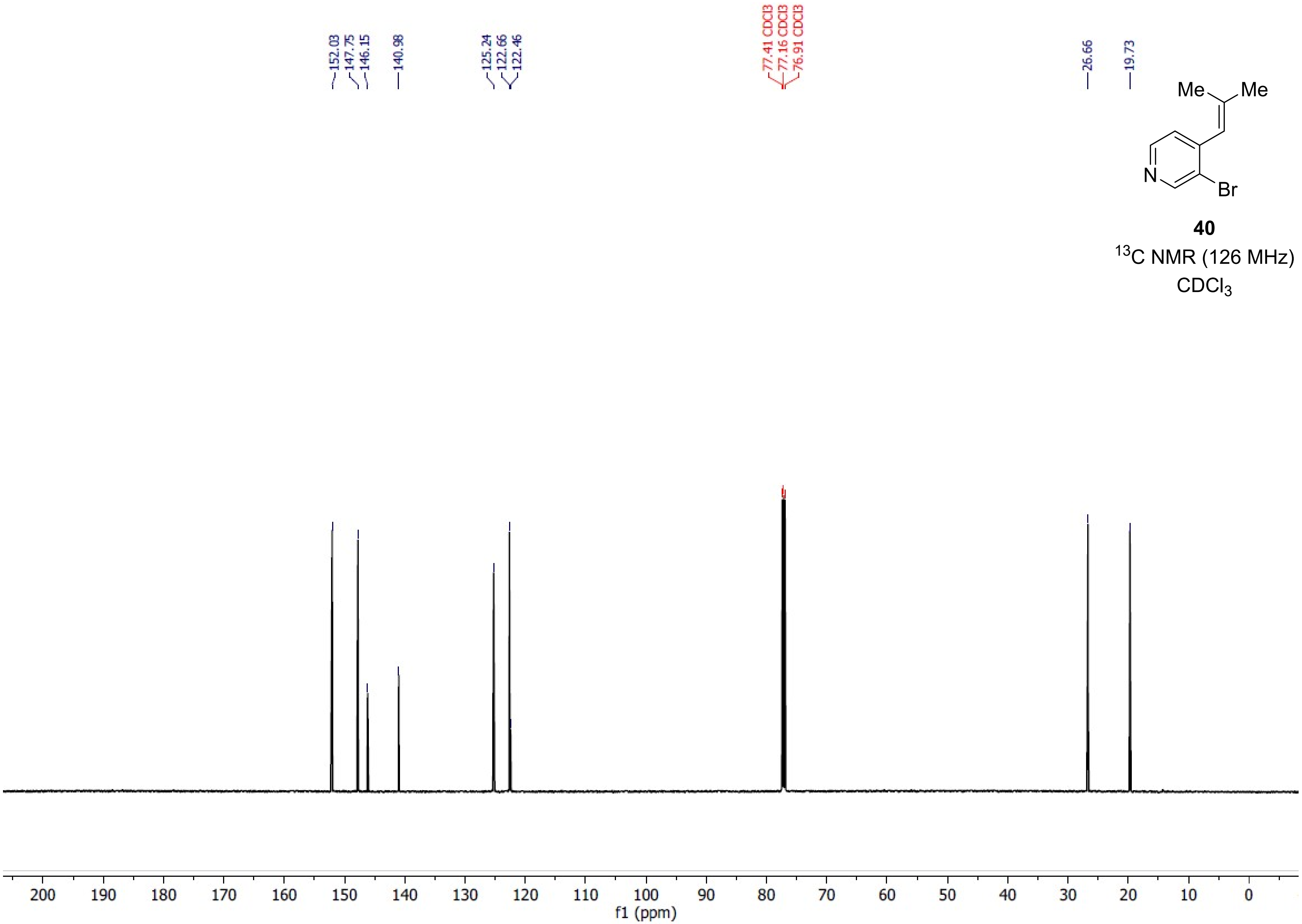
152.03
147.75
146.15
140.98
125.24
122.66
122.46
77.41 CDCl3
77.16 CDCl3
76.91 CDCl3
26.66
19.73
Me
Me
N
Br
40
^{13}C NMR (126 MHz)
$CDCl_3$
200 190 180 170 160 150 140 130 120 110 100 90 80 70 60 50 40 30 20 10 0
f1 (ppm)

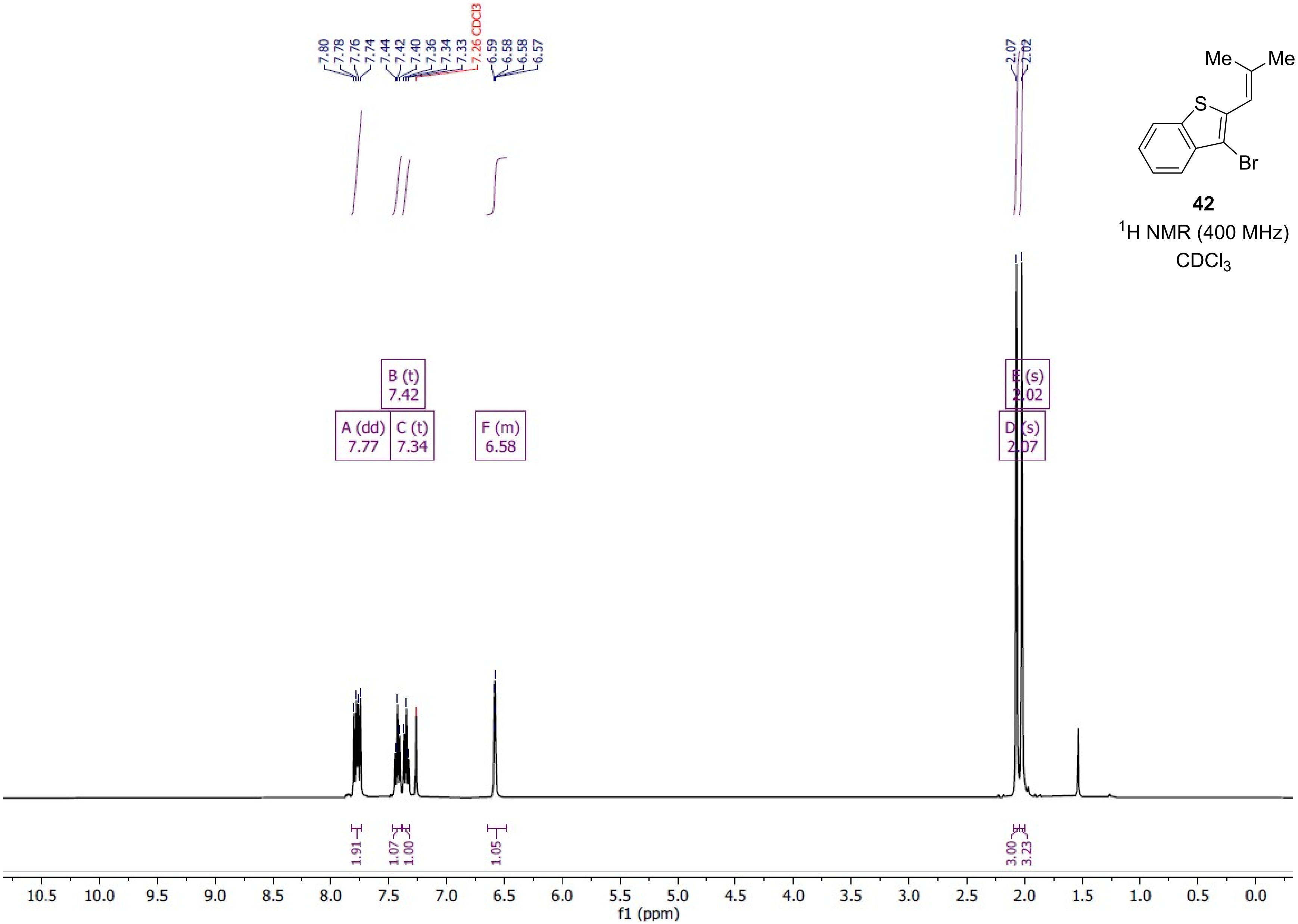

42

^1H NMR (400 MHz)

$CDCl_3$

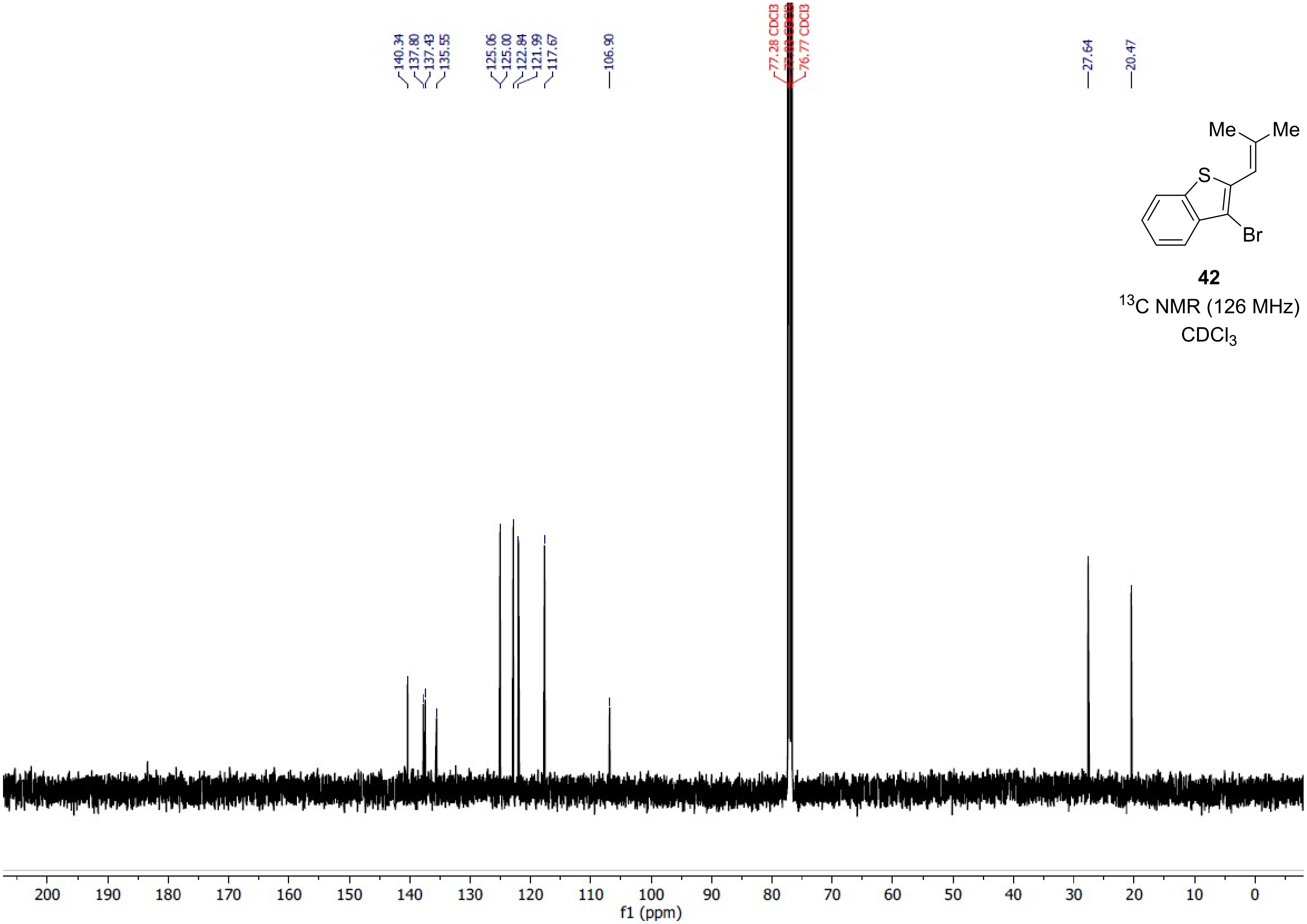

42

^{13}C NMR (126 MHz)

$CDCl_3$

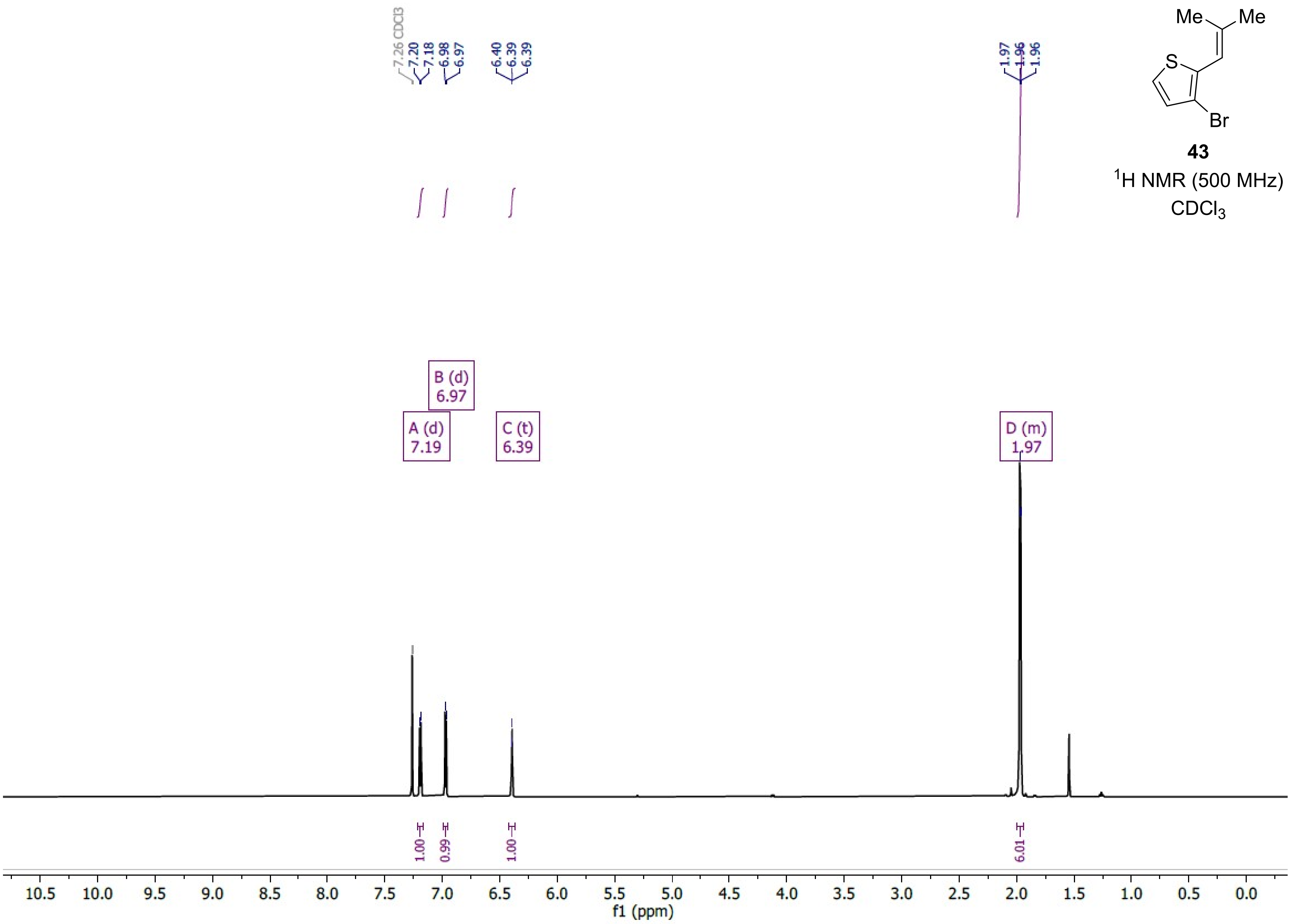

43

^{1}H NMR (500 MHz)

$CDCl_3$

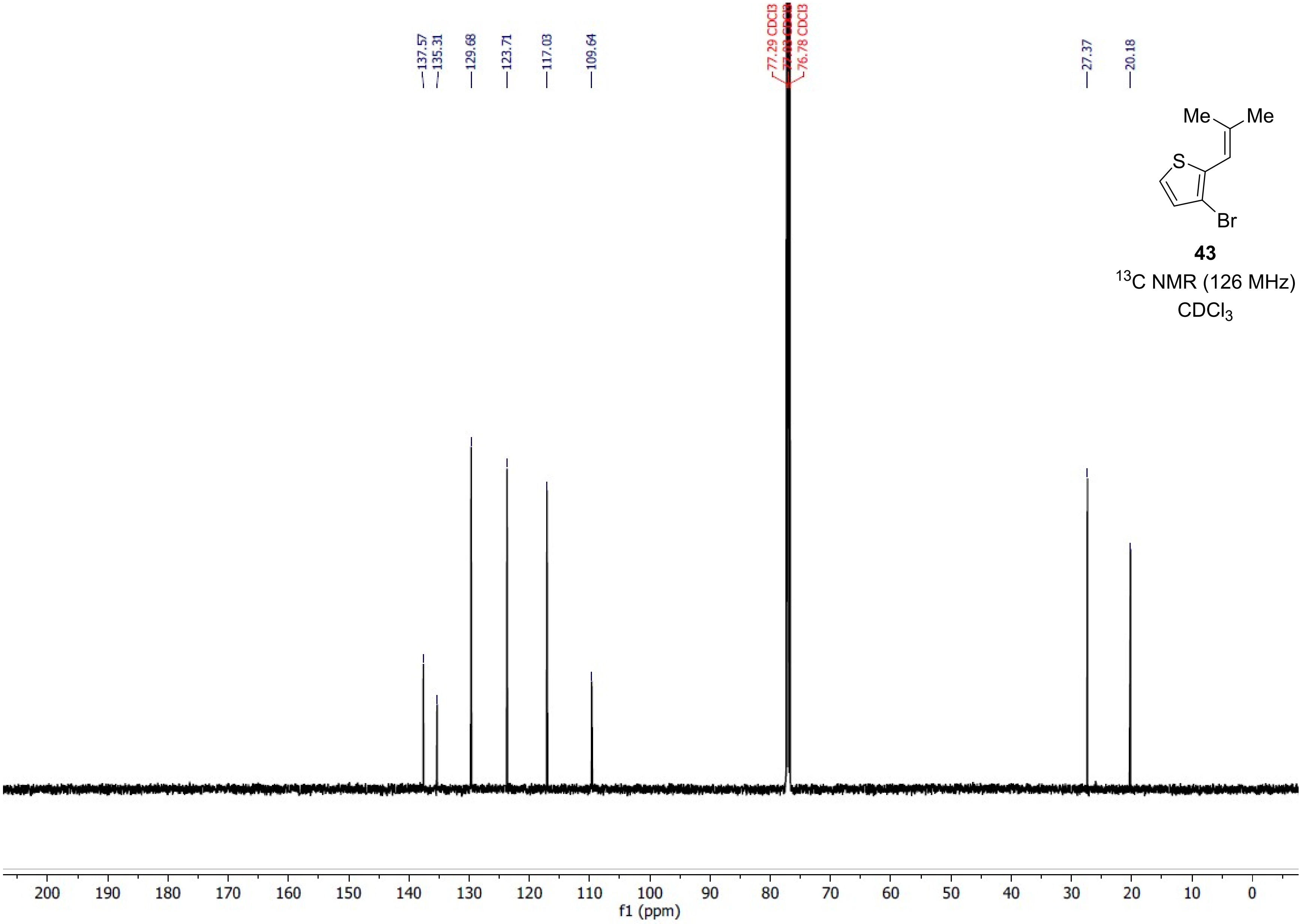

43

^{13}C NMR (126 MHz)

$CDCl_3$

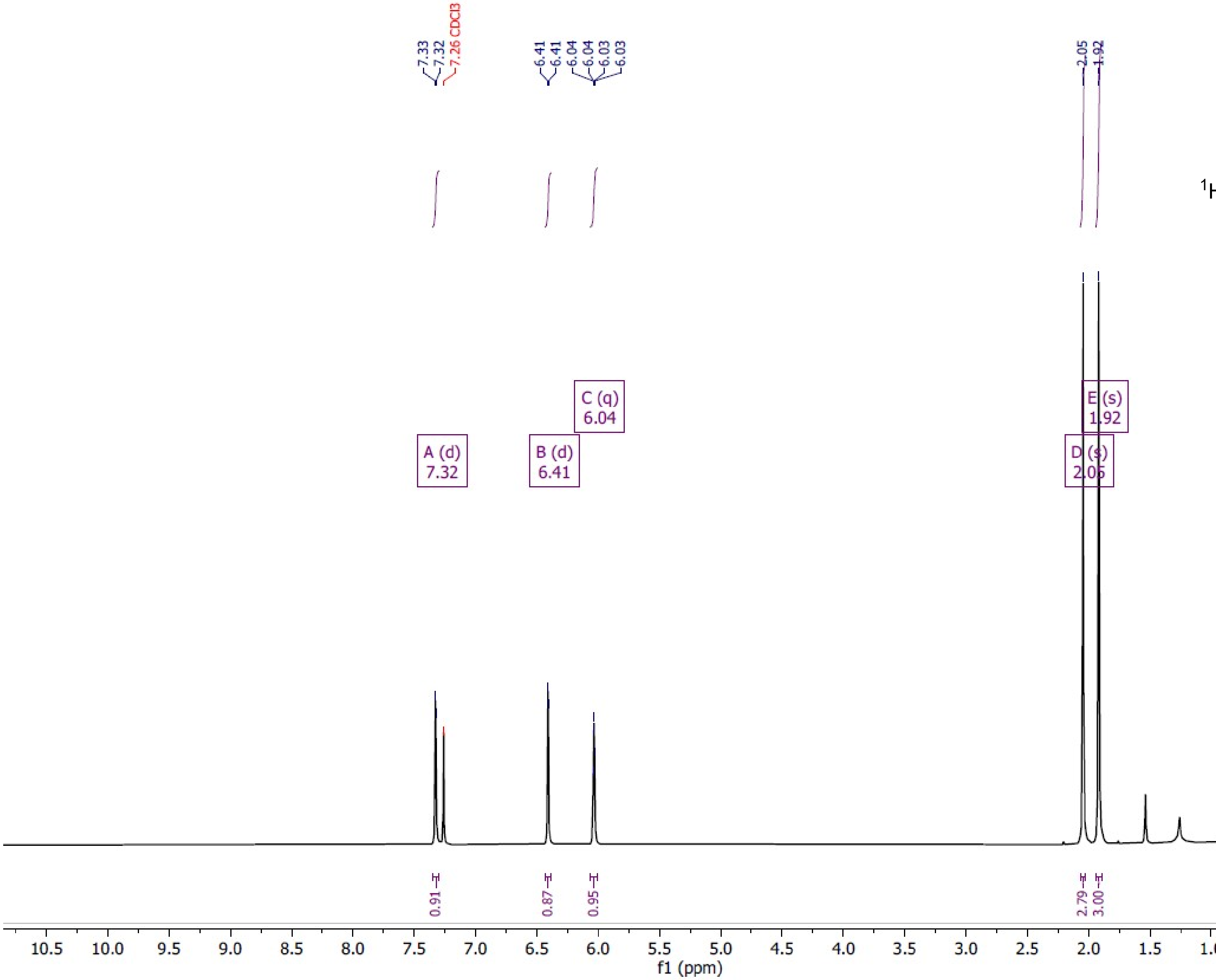

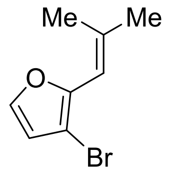

44

^{1}H NMR (400 MHz)

$CDCl_3$

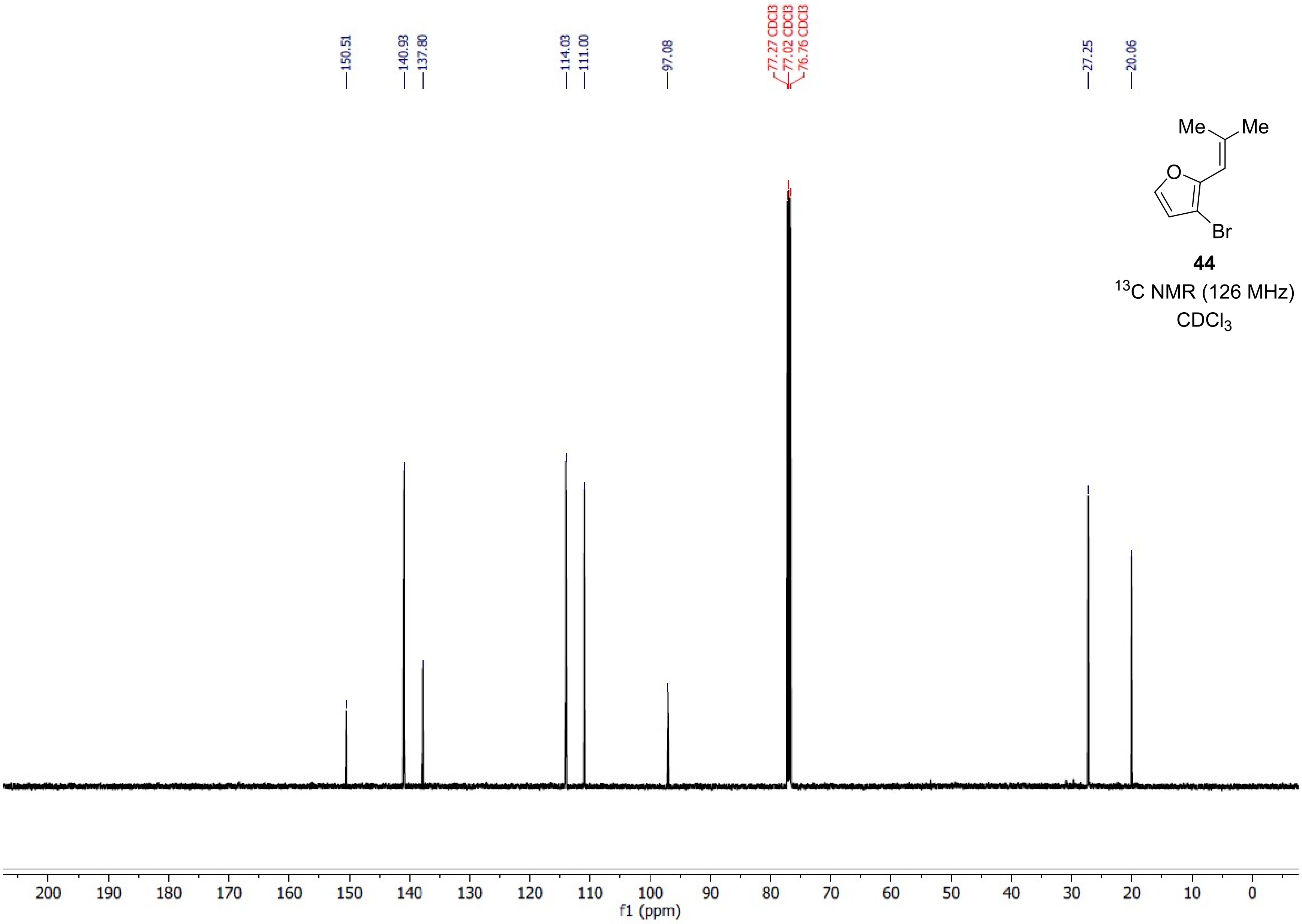
150.51
140.93
137.80
114.03
111.00
97.08
77.27 CDCl3
77.02 CDCl3
76.76 CDCl3
27.25
20.06
Me
Me
O
Br
44
^{13}C NMR (126 MHz)
$CDCl_3$
200 190 180 170 160 150 140 130 120 110 100 90 80 70 60 50 40 30 20 10 0
f1 (ppm)

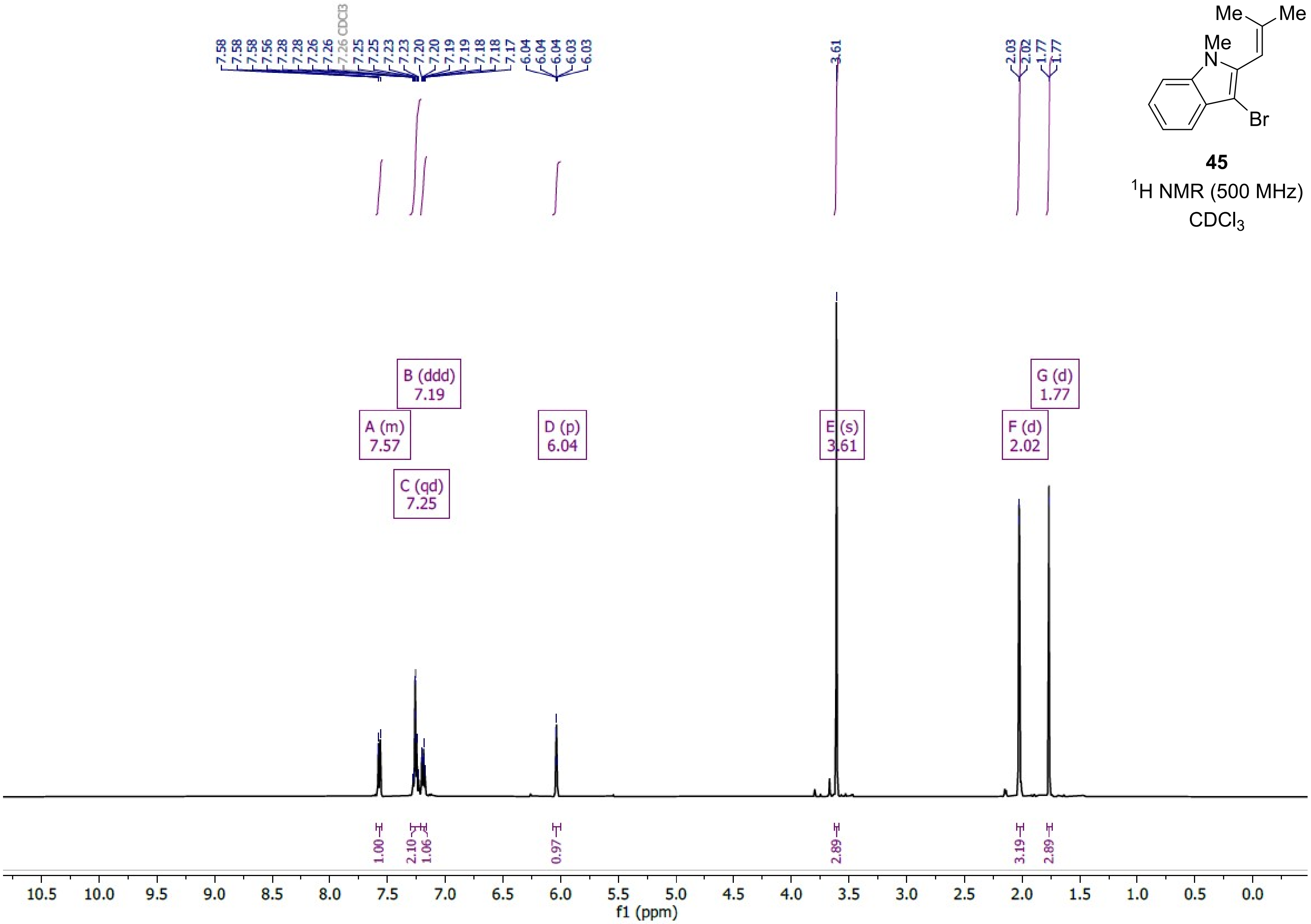

45

^{1}H NMR (500 MHz)

$CDCl_3$

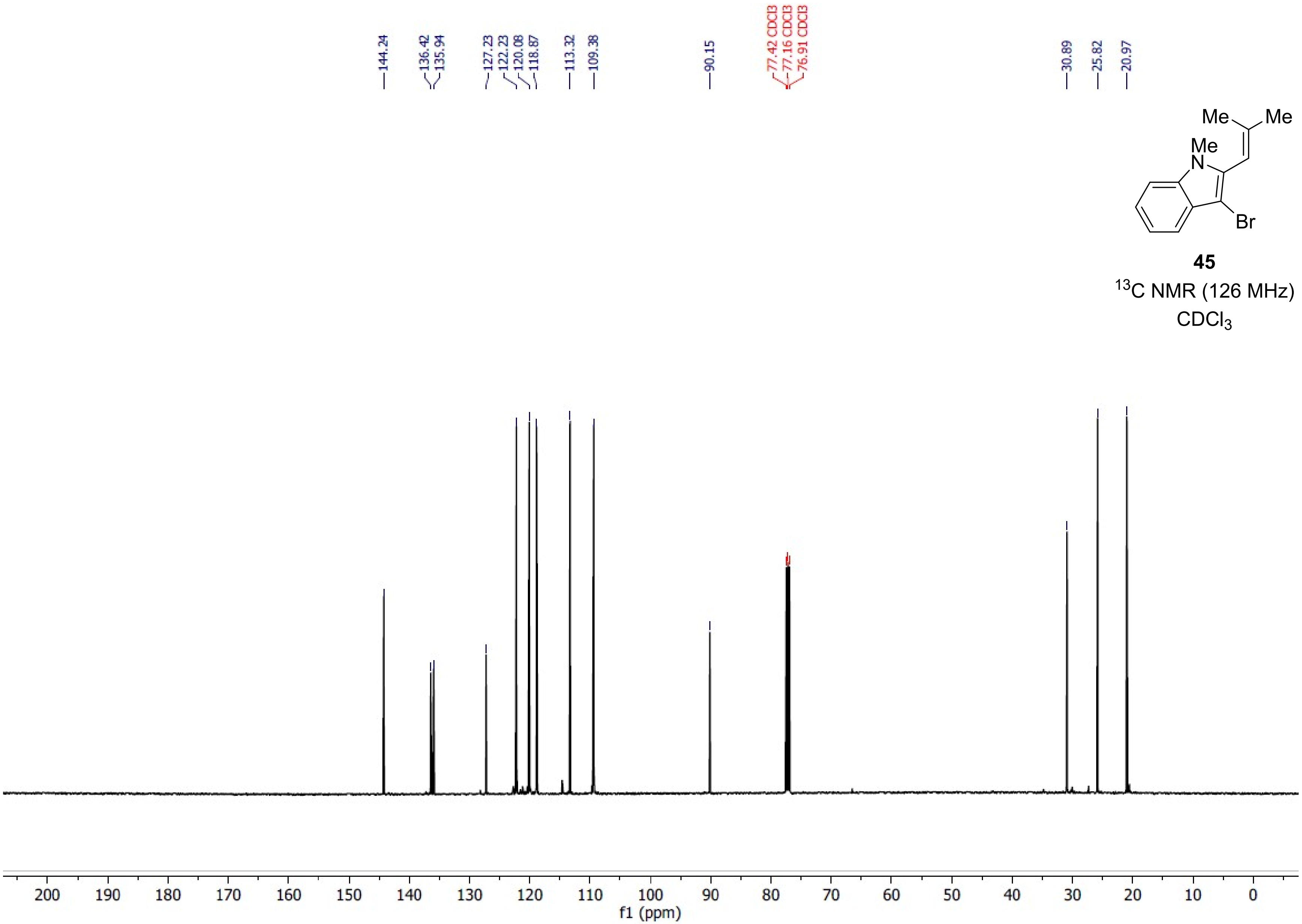

45

^{13}C NMR (126 MHz)

$CDCl_3$

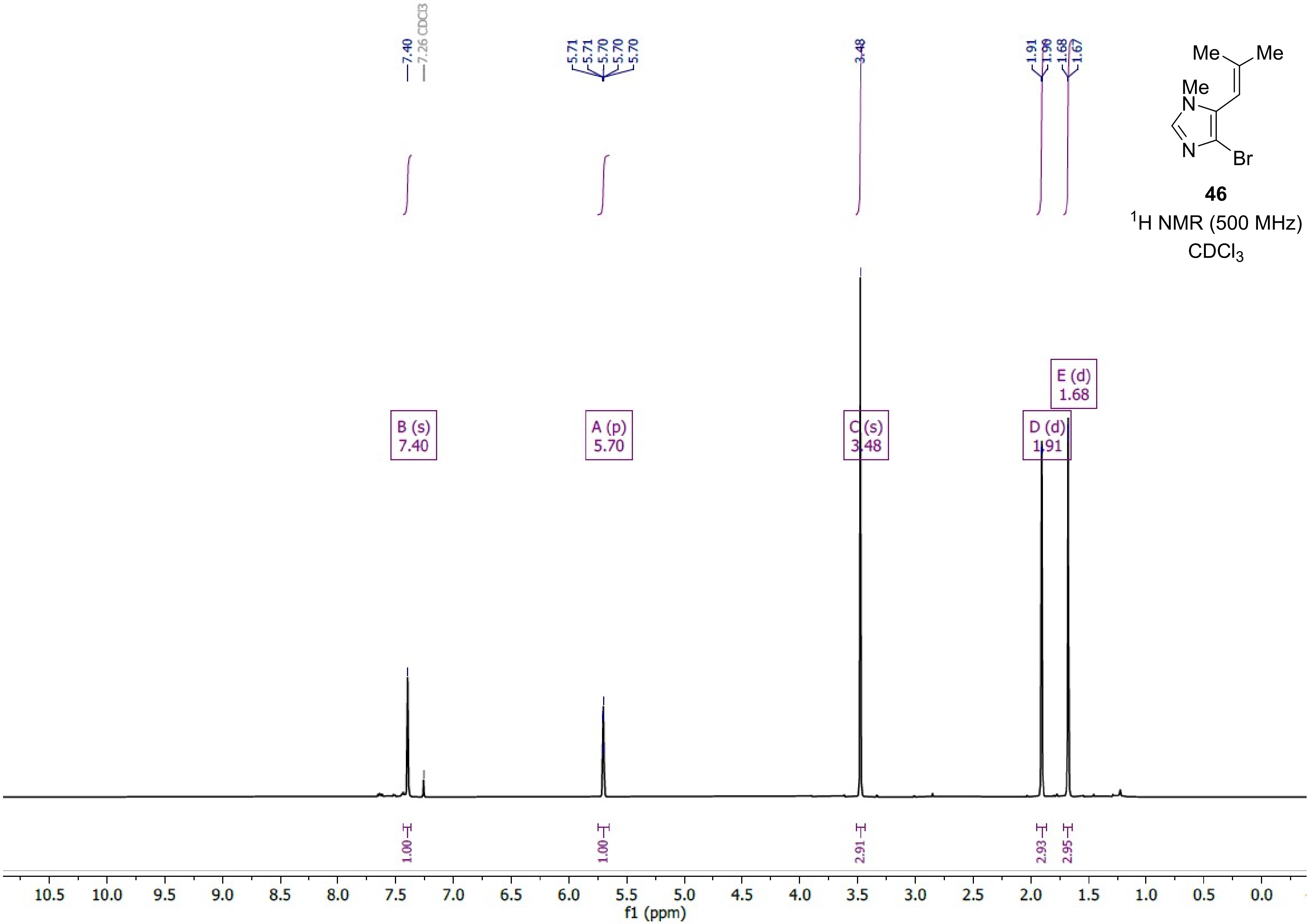

46

^{1}H NMR (500 MHz)

$CDCl_3$

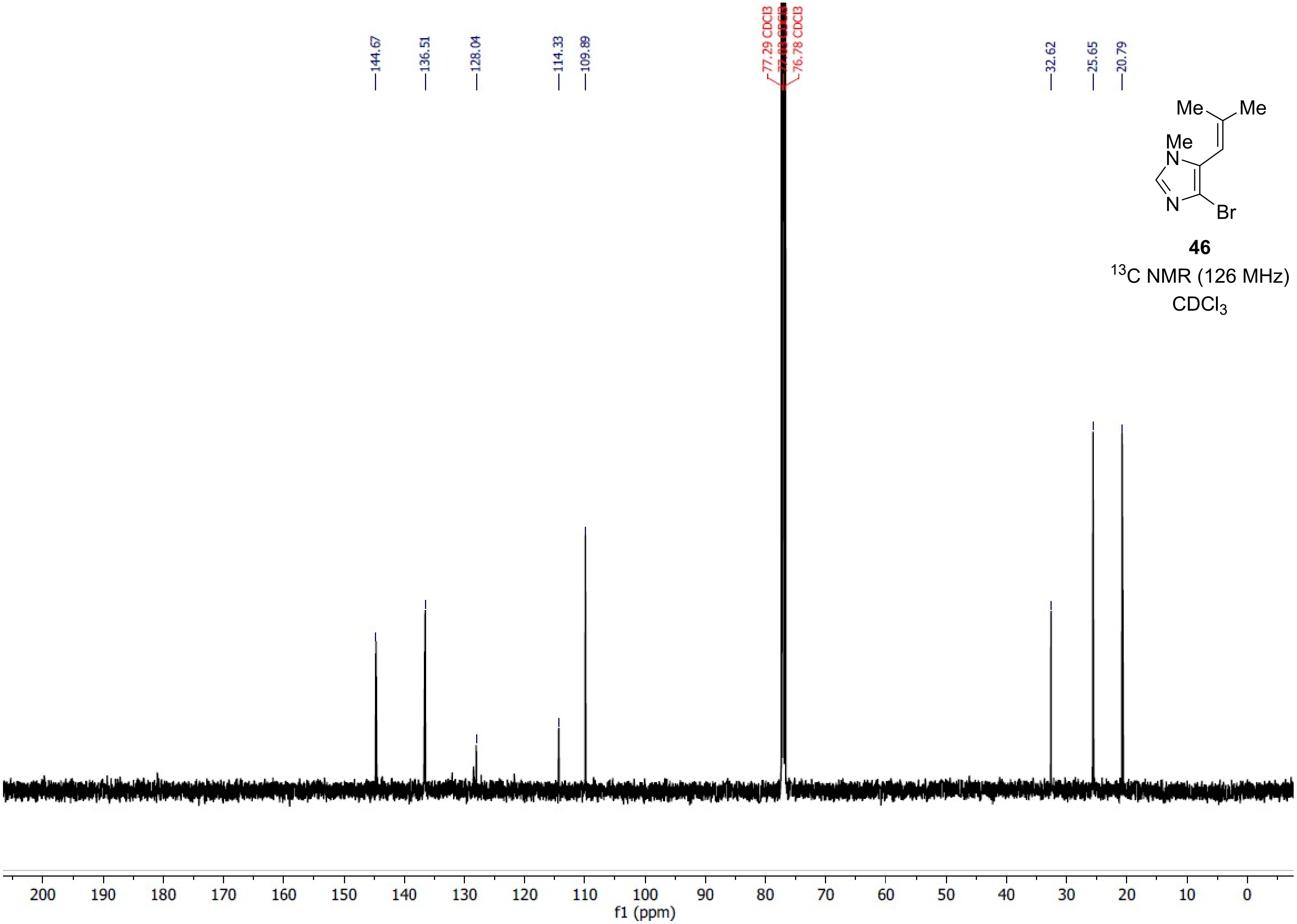

46
^{13}C NMR (126 MHz)
$CDCl_3$

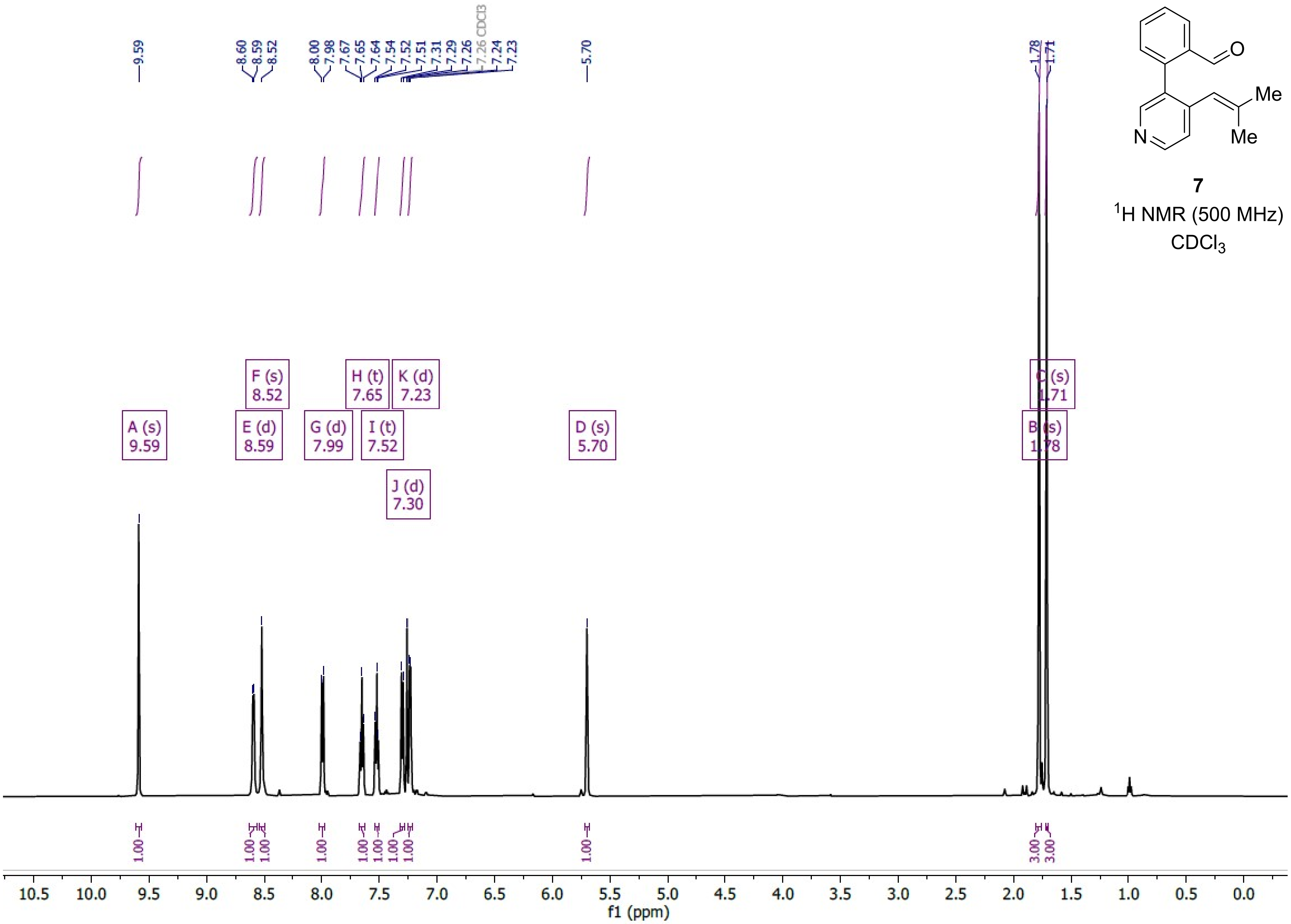

7

^{1}H NMR (500 MHz)

$CDCl_3$

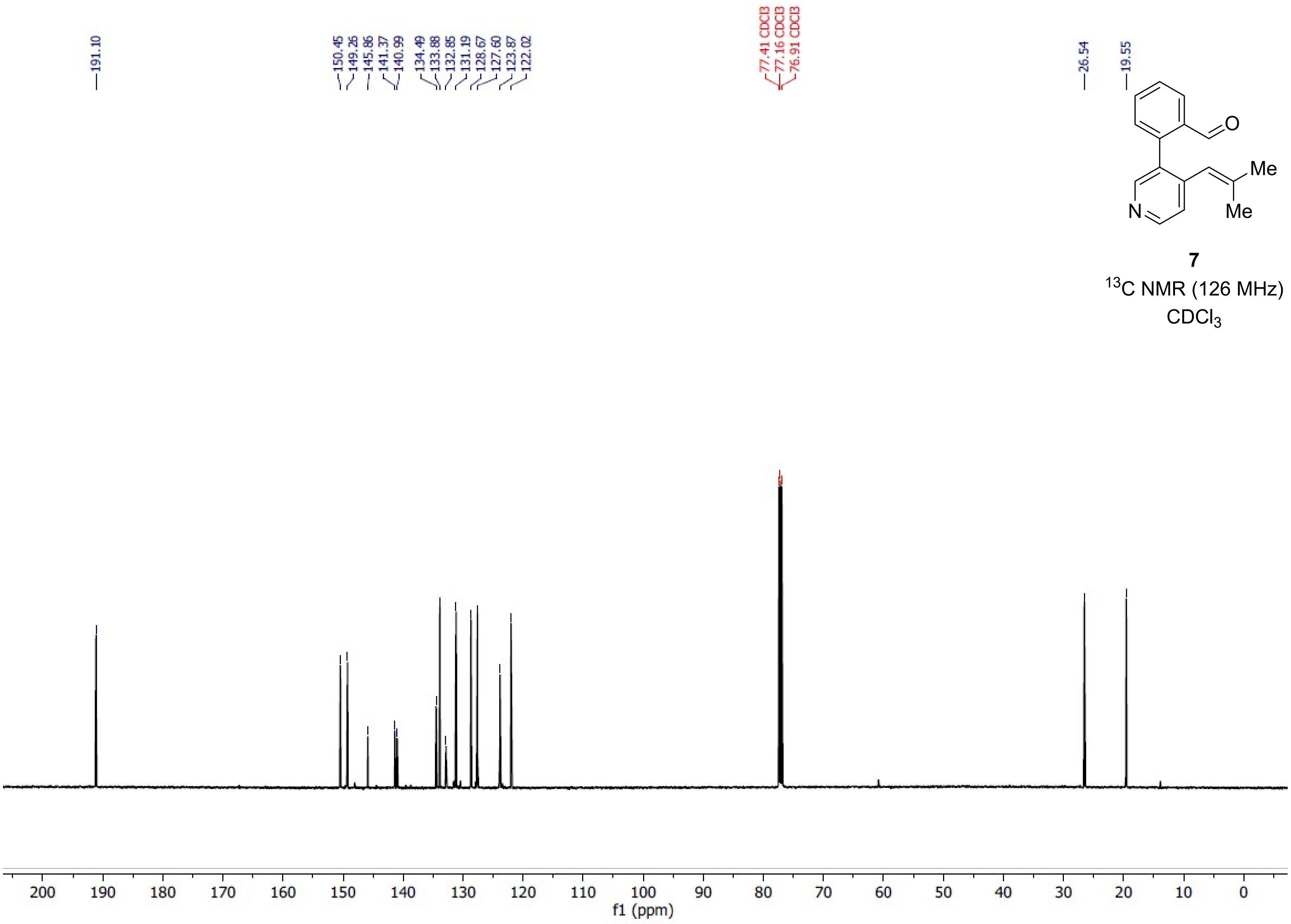
191.10
150.45
149.26
145.86
141.37
140.99
134.49
133.88
132.85
131.19
128.67
127.60
123.87
122.02
77.41 CDCl3
77.16 CDCl3
76.91 CDCl3
26.54
19.55
O
Me
Me
N
7
13C NMR (126 MHz)
CDCl3
200 190 180 170 160 150 140 130 120 110 100 90 80 70 60 50 40 30 20 10 0
f1 (ppm)

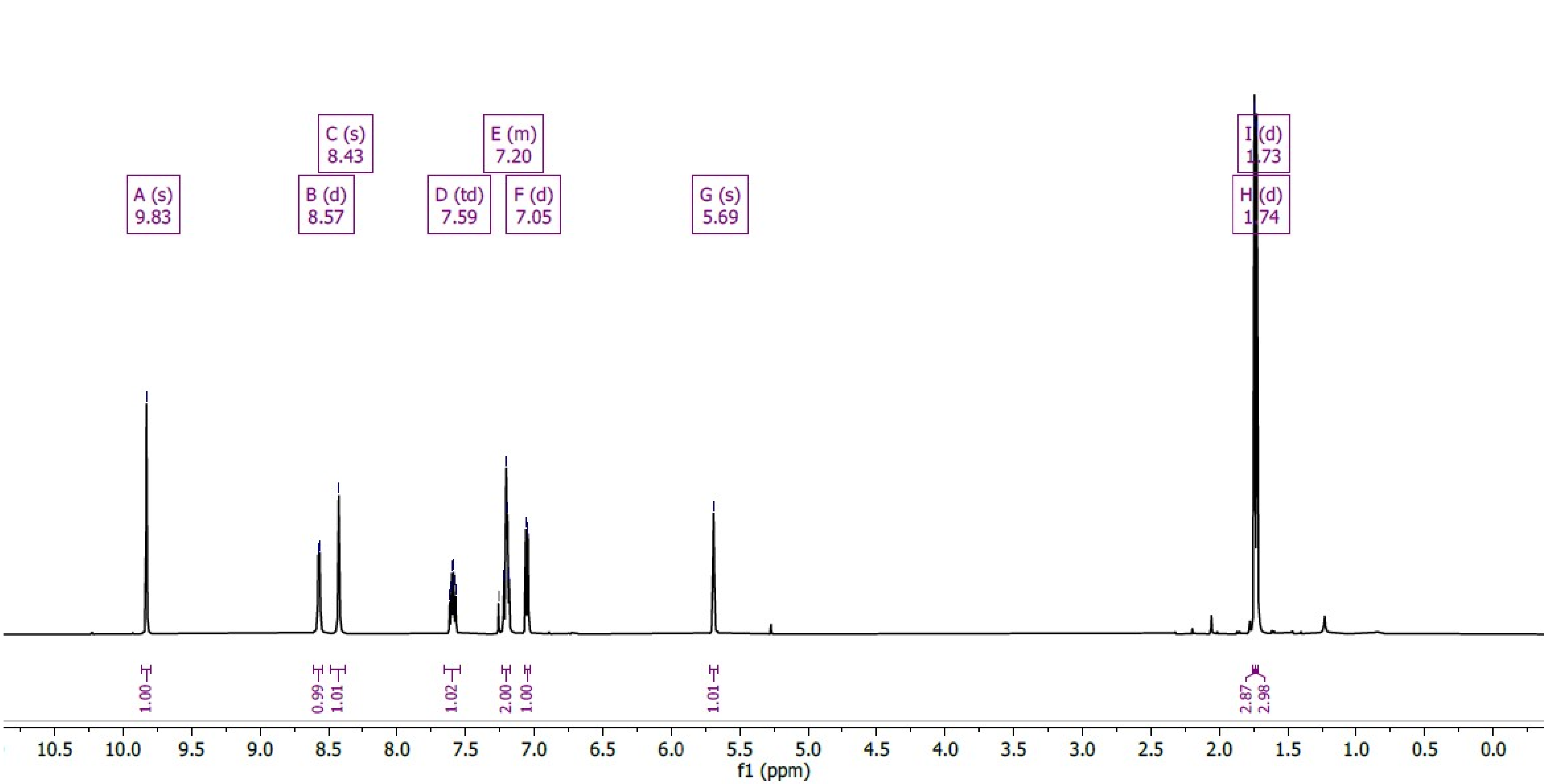

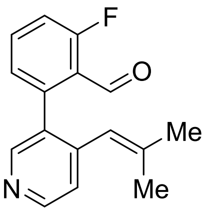

47

^{1}H NMR (500 MHz)

$CDCl_3$

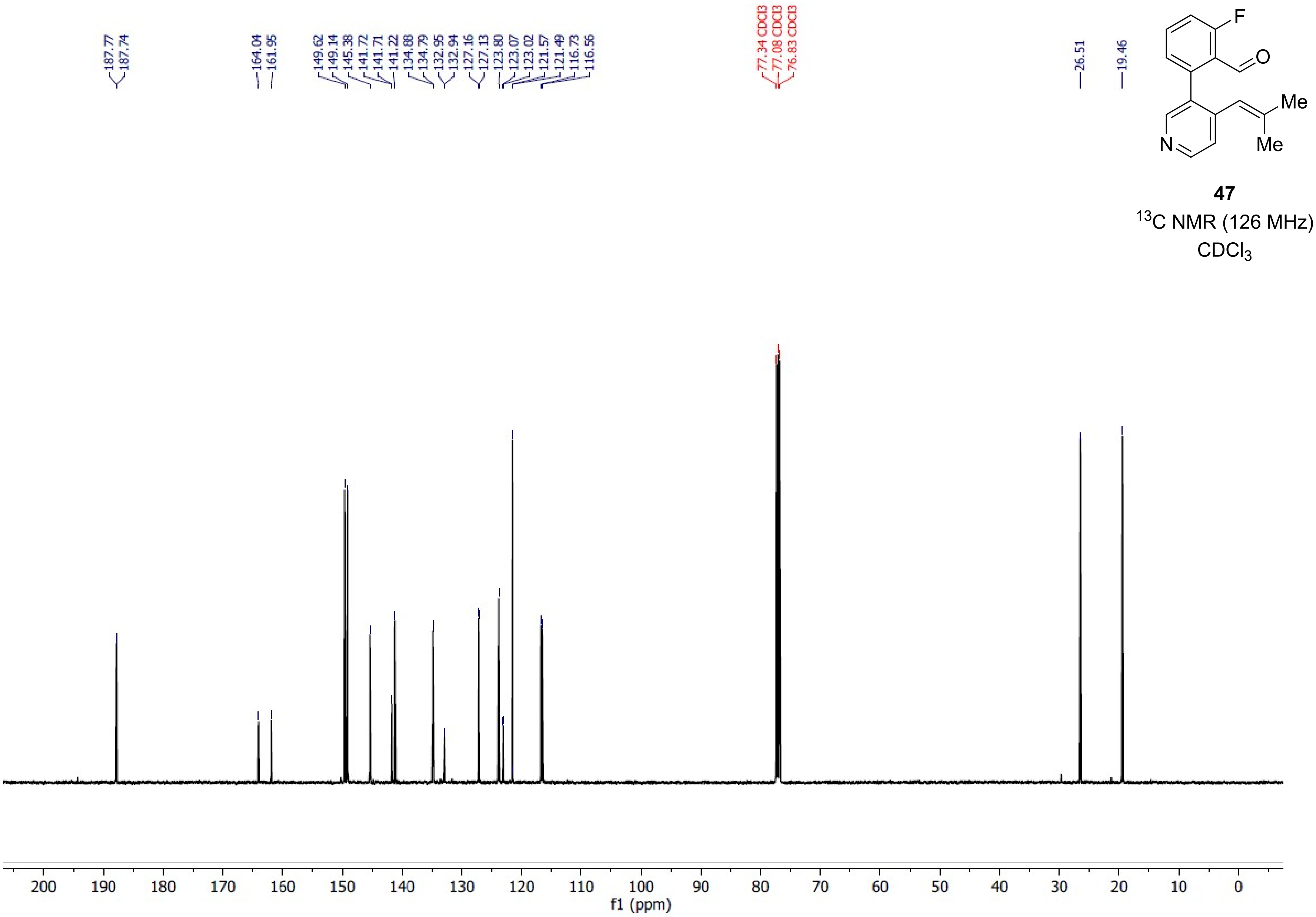

47

^{13}C NMR (126 MHz)

$CDCl_3$

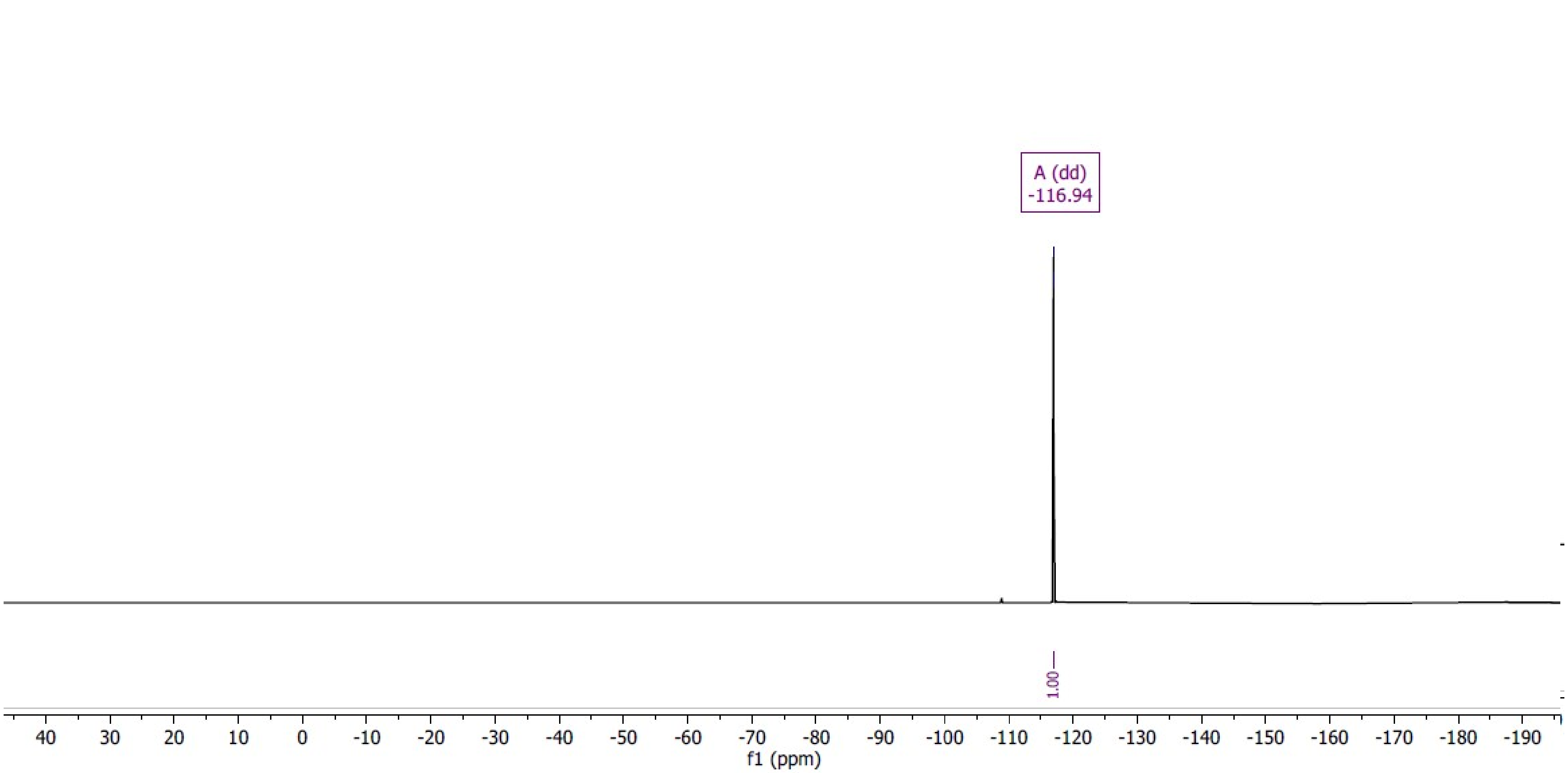

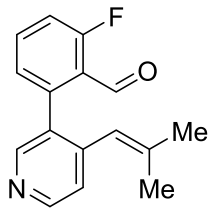

47

^{19}F NMR (470 MHz)

$CDCl_3$

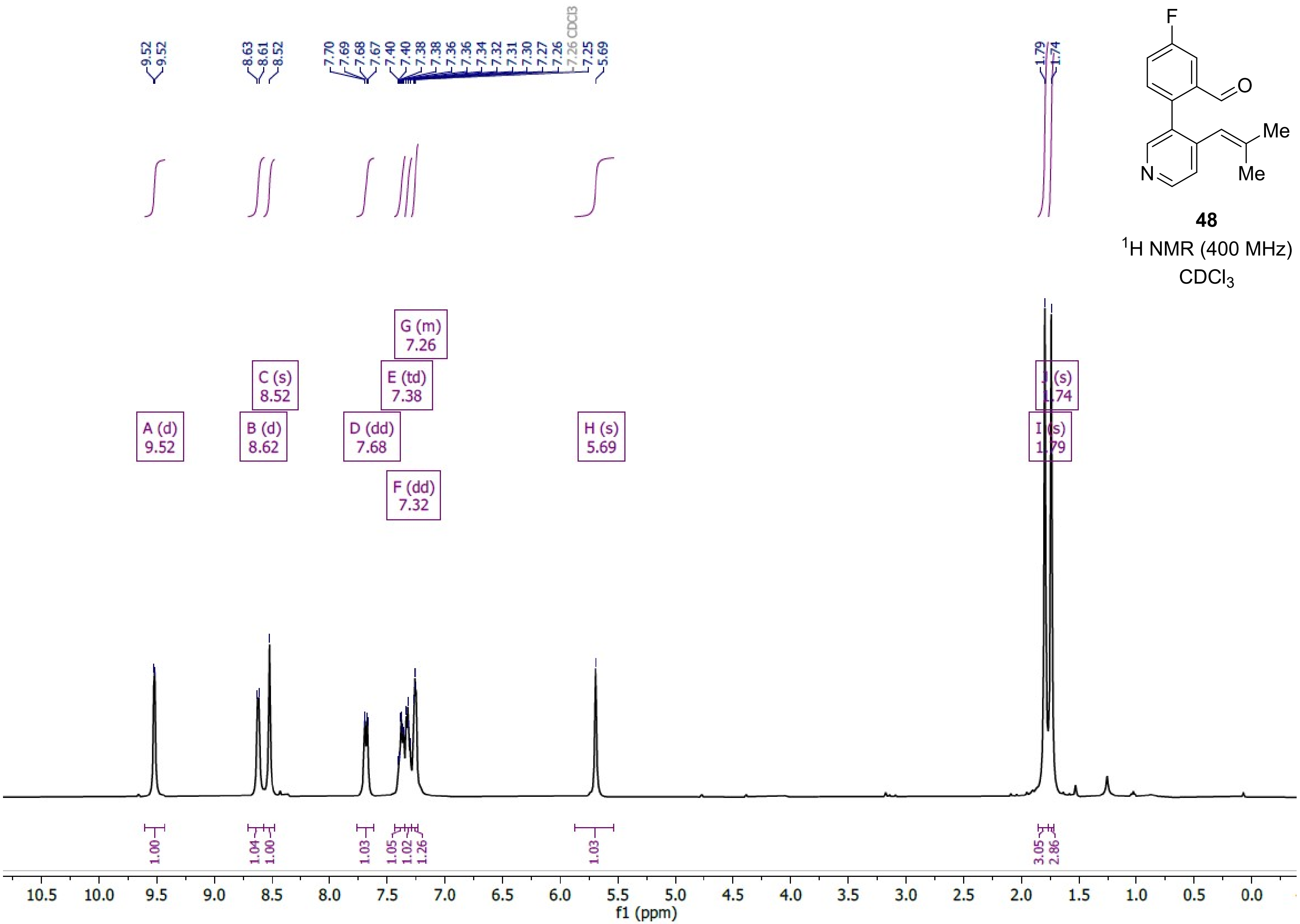

48

^{1}H NMR (400 MHz)

$CDCl_3$

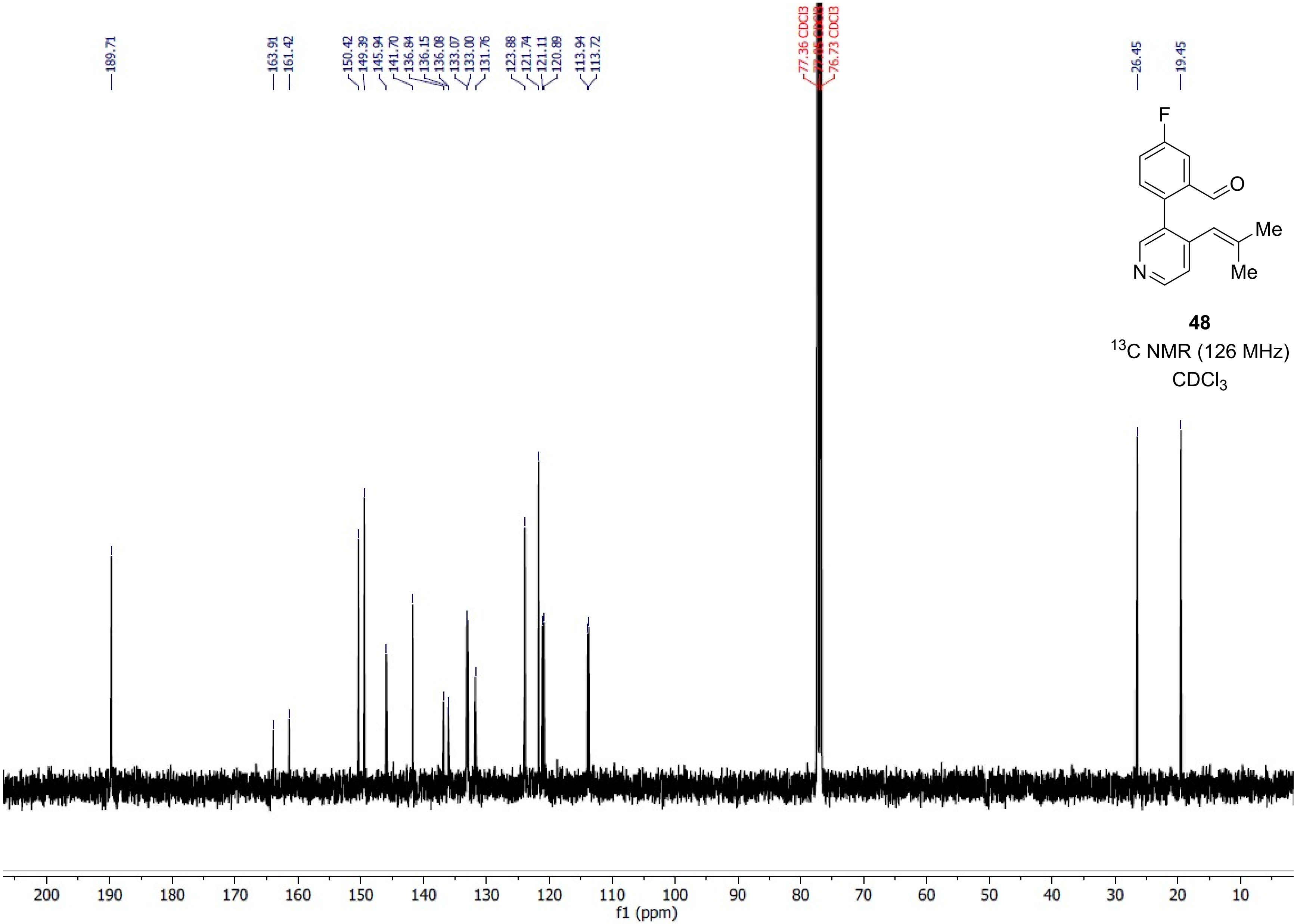

48

^{13}C NMR (126 MHz)

$CDCl_3$

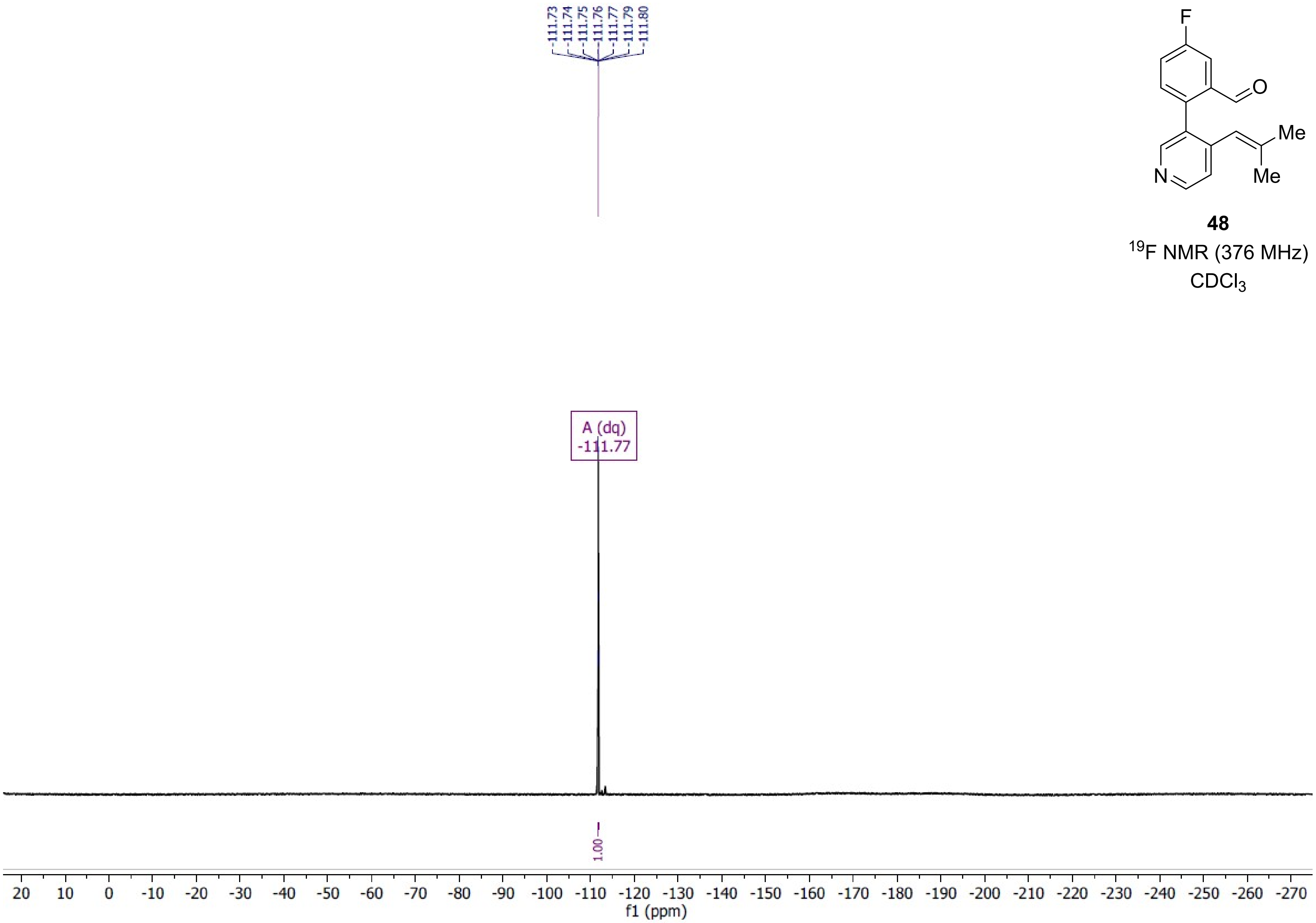

48

^{19}F NMR (376 MHz)

$CDCl_3$

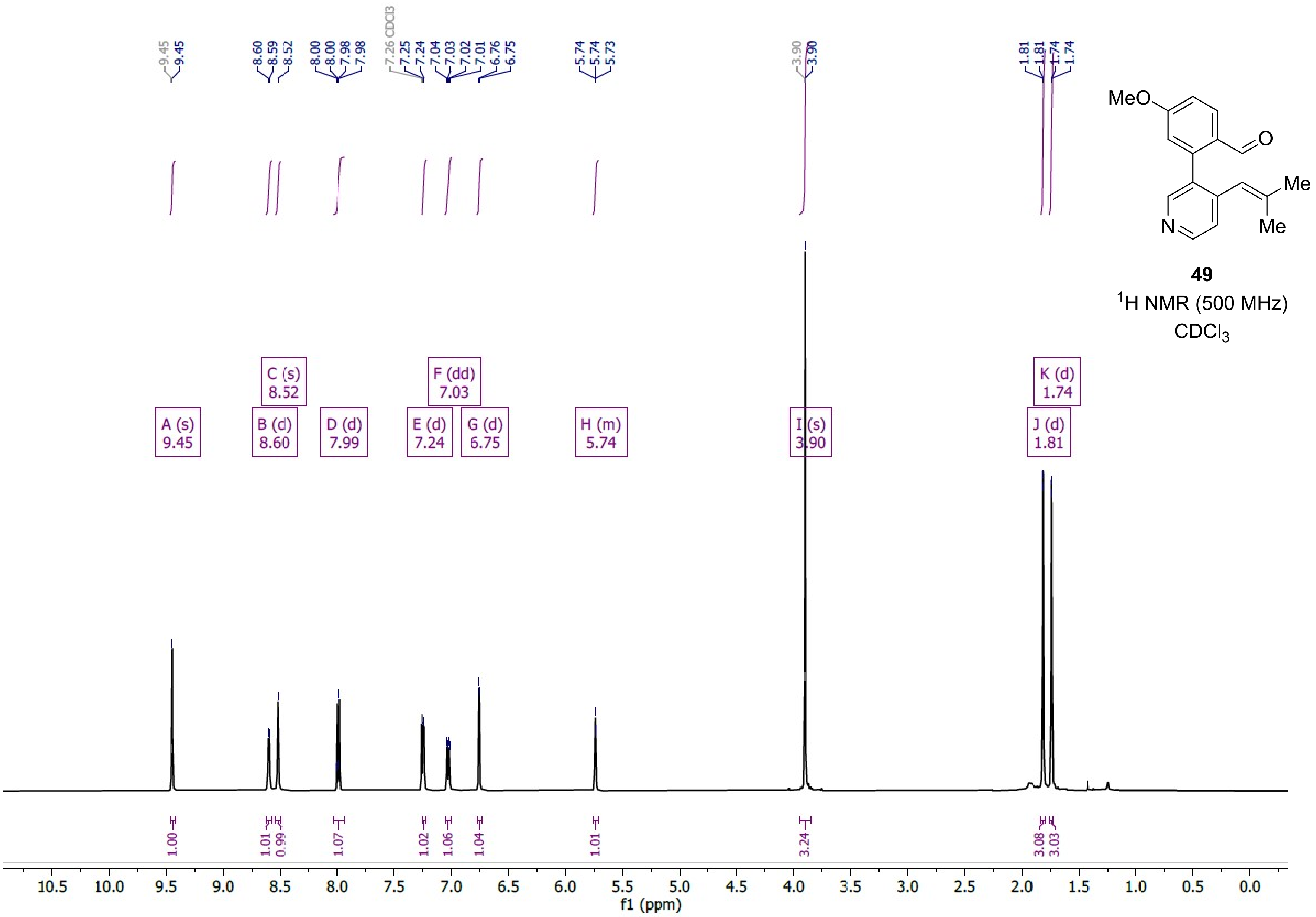

49

^{1}H NMR (500 MHz)

$CDCl_3$

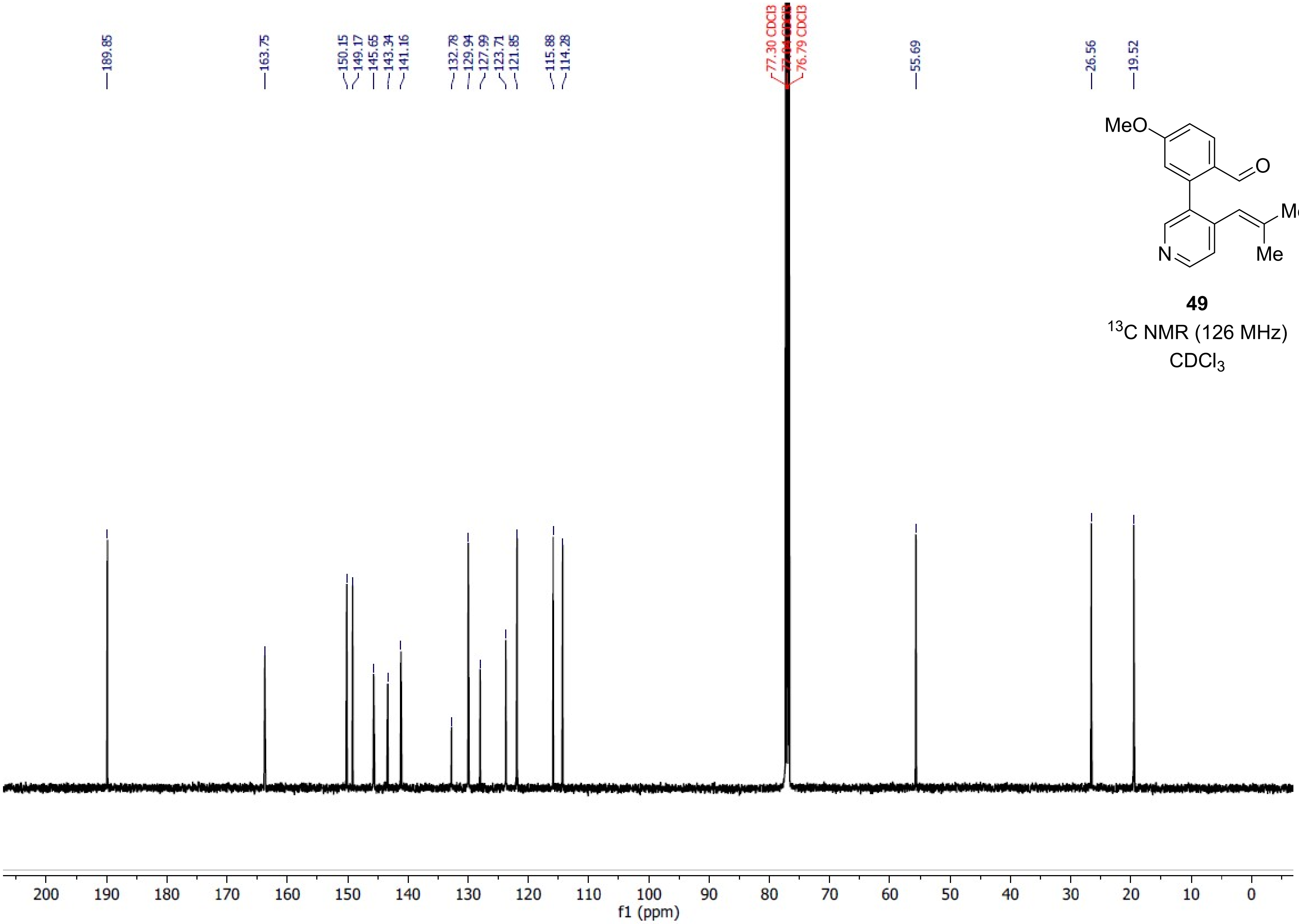

49

^{13}C NMR (126 MHz)

$CDCl_3$

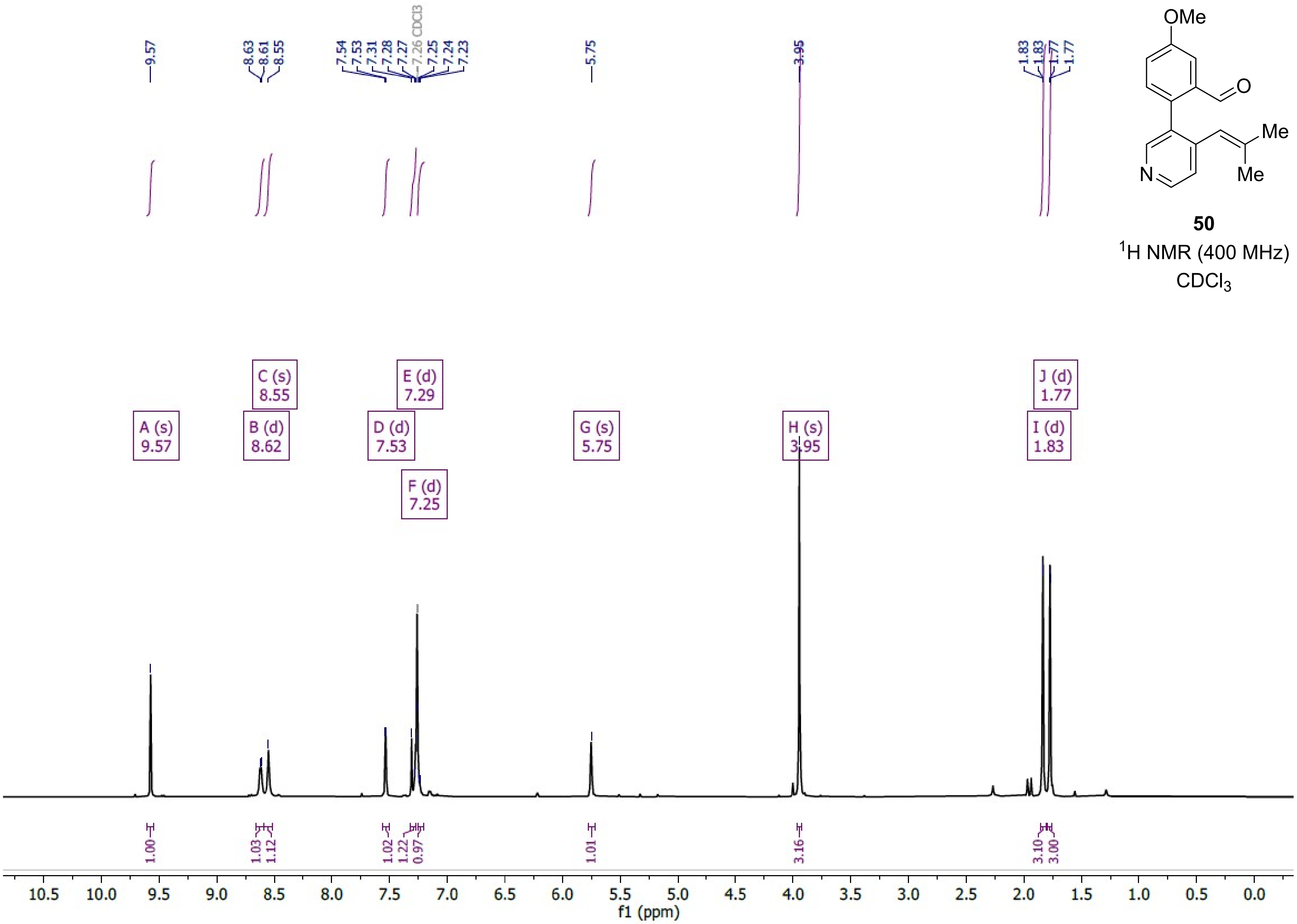

50

^{1}H NMR (400 MHz)

$CDCl_3$

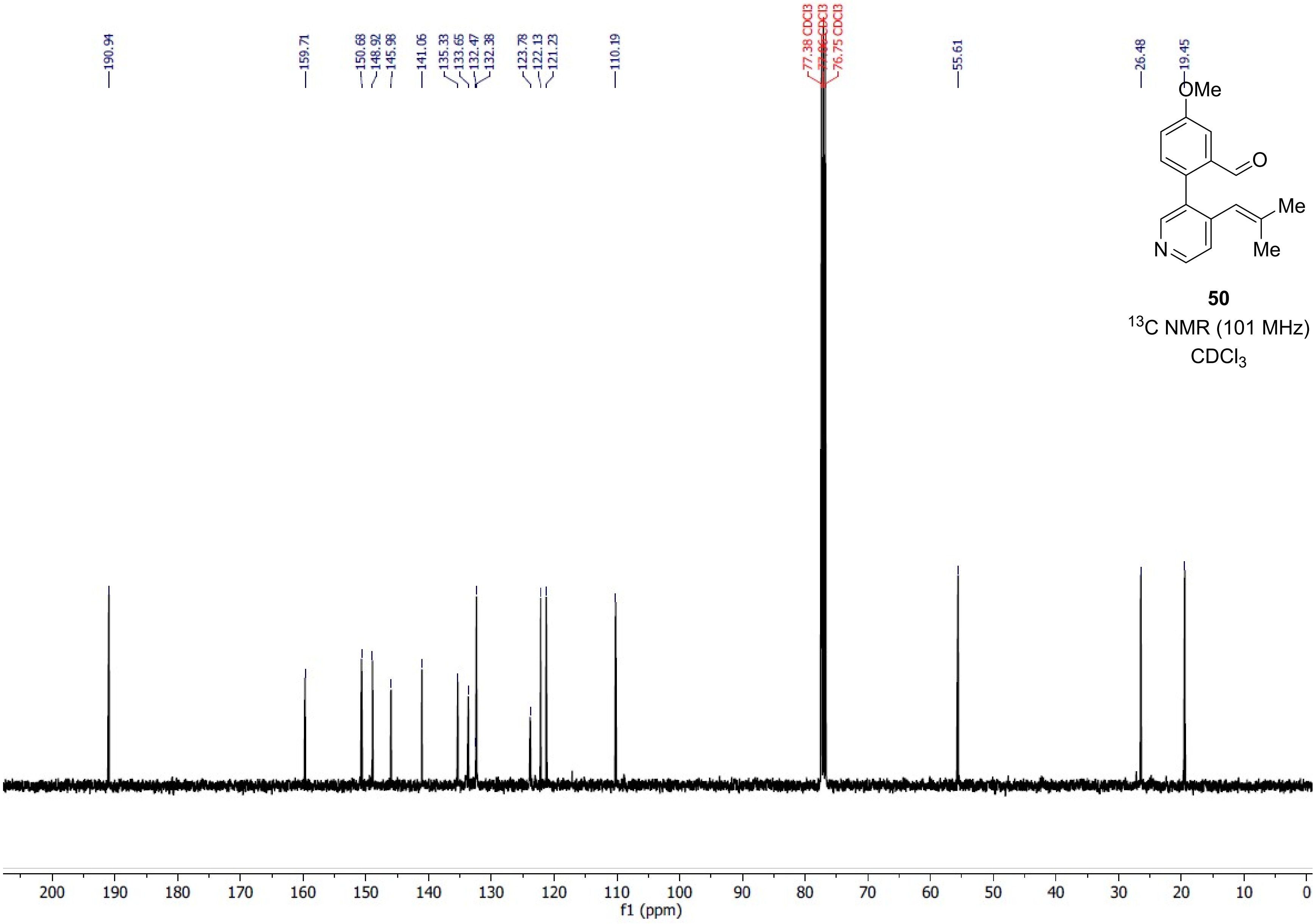

190.94
159.71
150.68
148.92
145.98
141.06
135.33
133.65
132.47
132.38
123.78
122.13
121.23
110.19
77.38 CDCl3
77.06 CDCl3
76.75 CDCl3
55.61
26.48
19.45
OMe
O
Me
Me
N
50
^{13}C NMR (101 MHz)
$CDCl_3$
200
190
180
170
160
150
140
130
120
110
100
90
80
70
60
50
40
30
20
10
0
f1 (ppm)

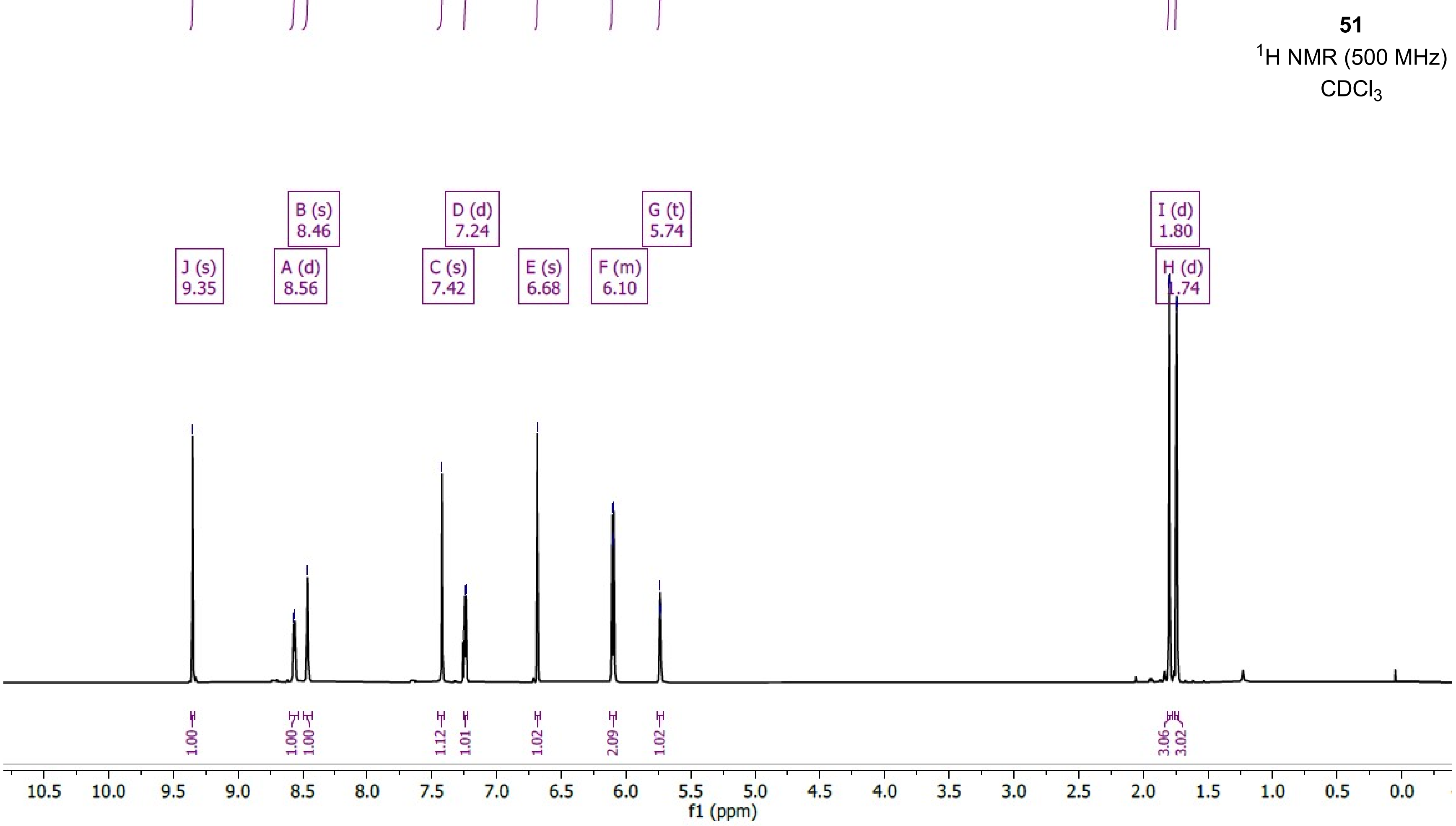

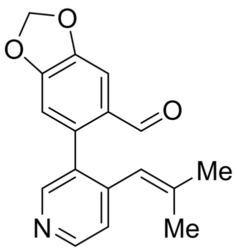

51

^{1}H NMR (500 MHz)

$CDCl_3$

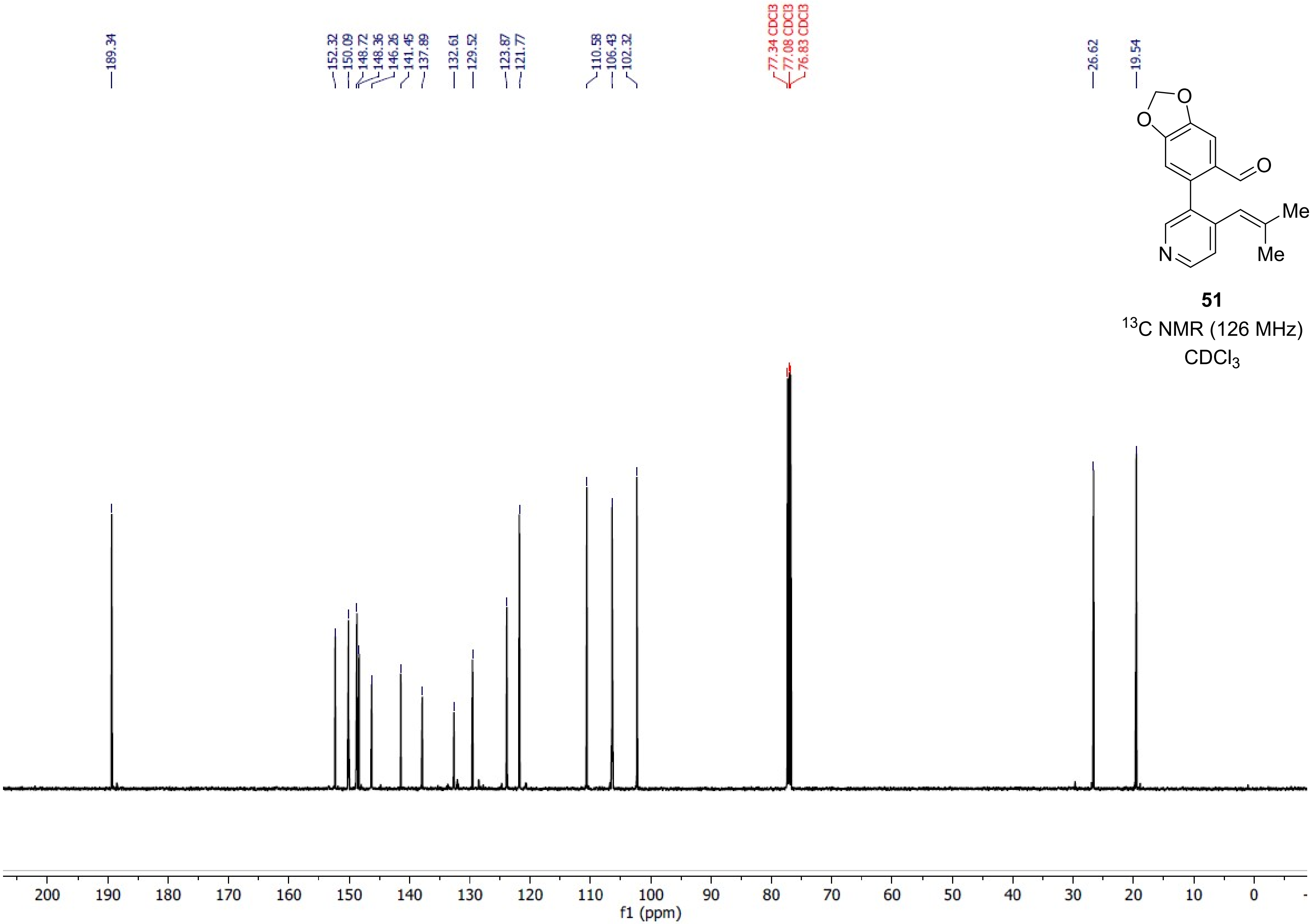

51

^{13}C NMR (126 MHz)

$CDCl_3$

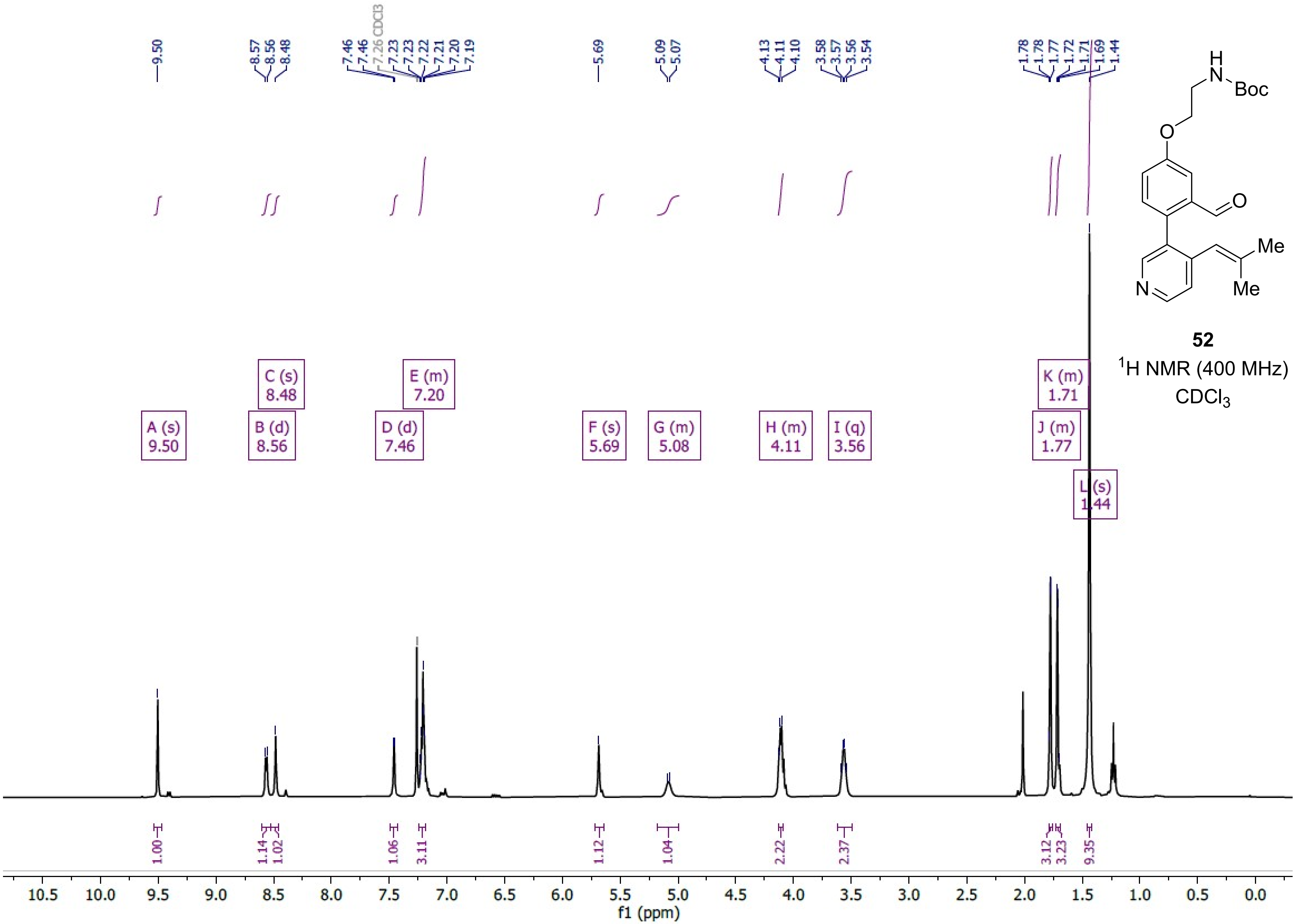

52

^{1}H NMR (400 MHz)

$CDCl_3$

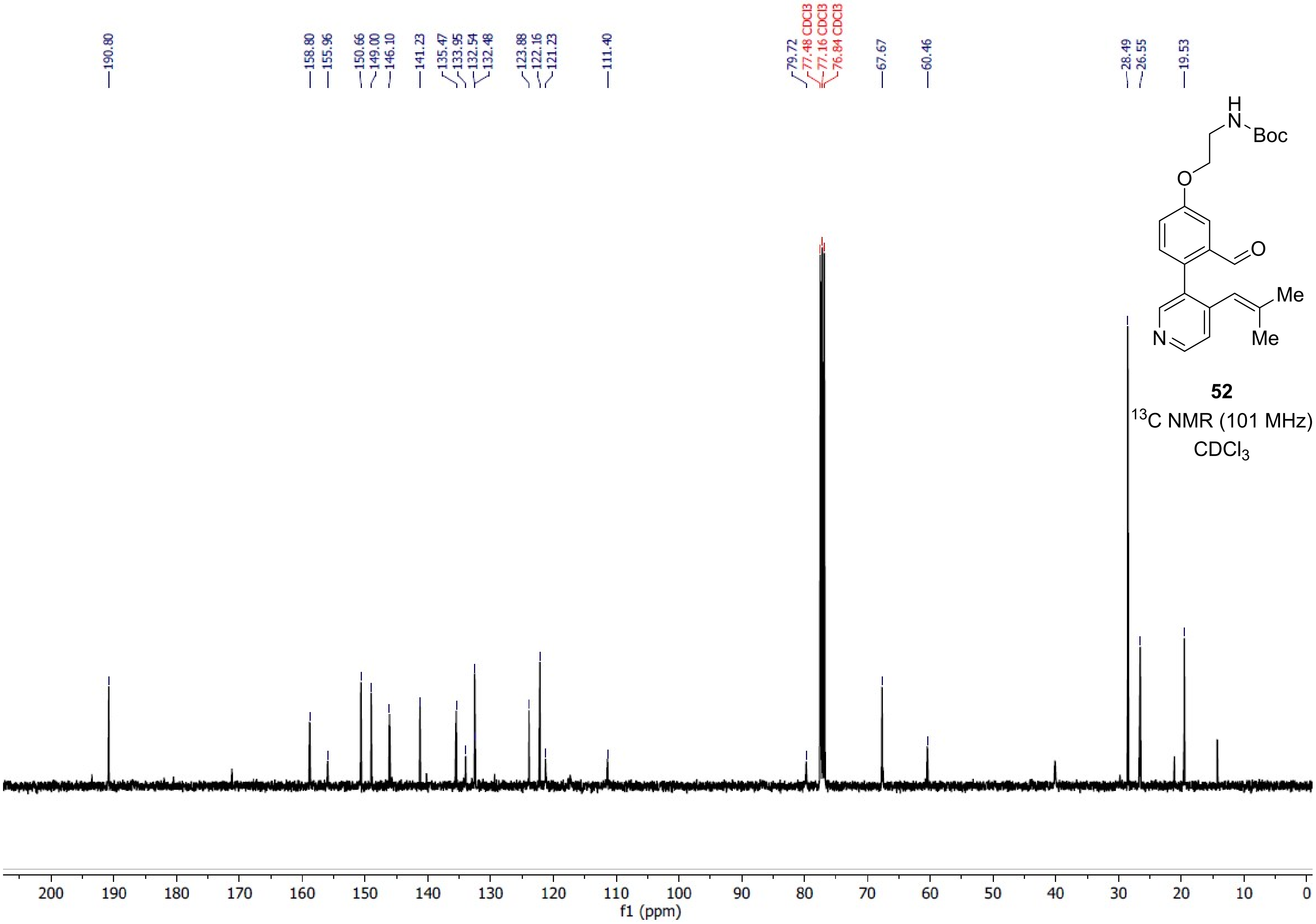

52

^{13}C NMR (101 MHz)

$CDCl_3$

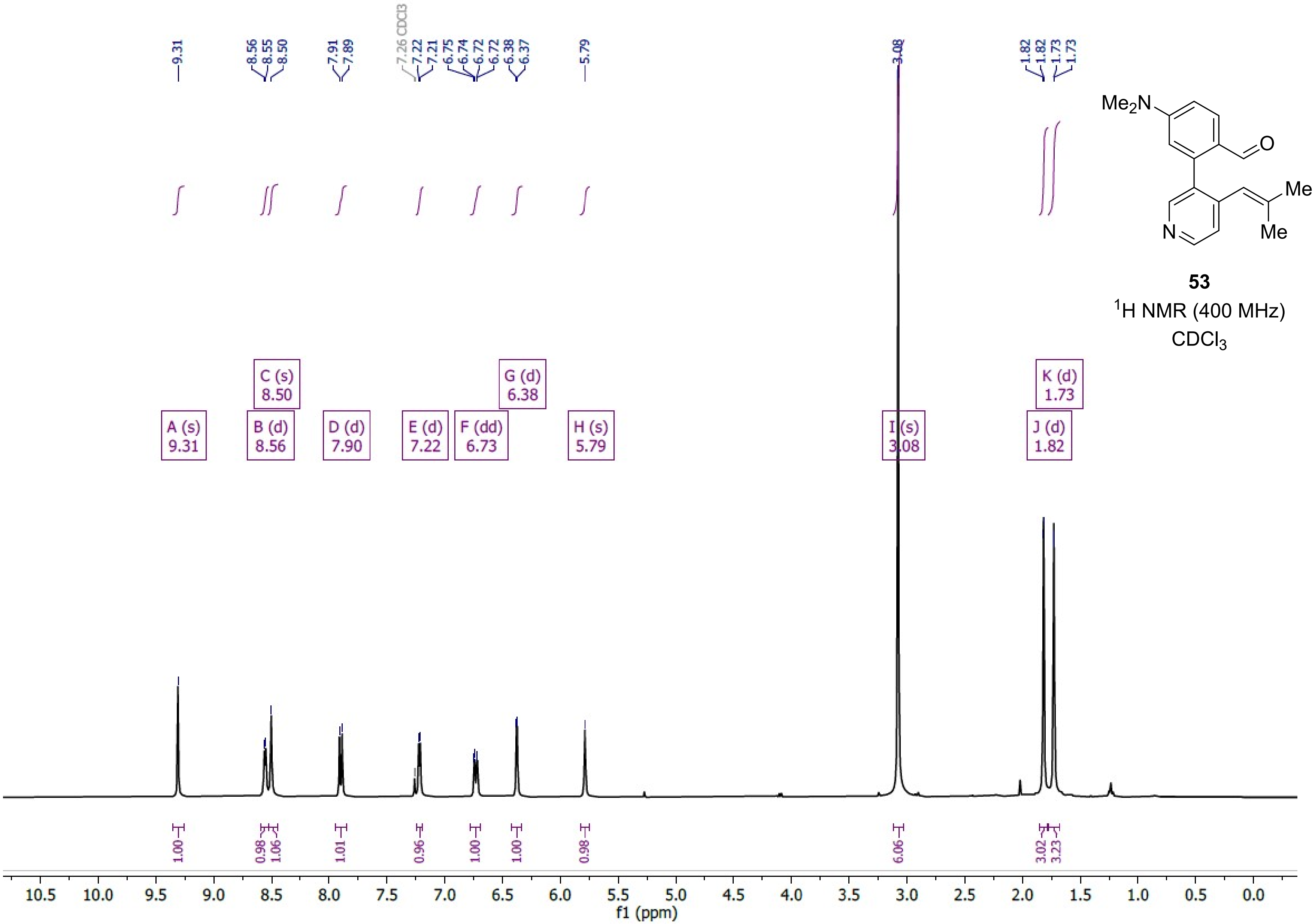

53

^{1}H NMR (400 MHz)

$CDCl_3$

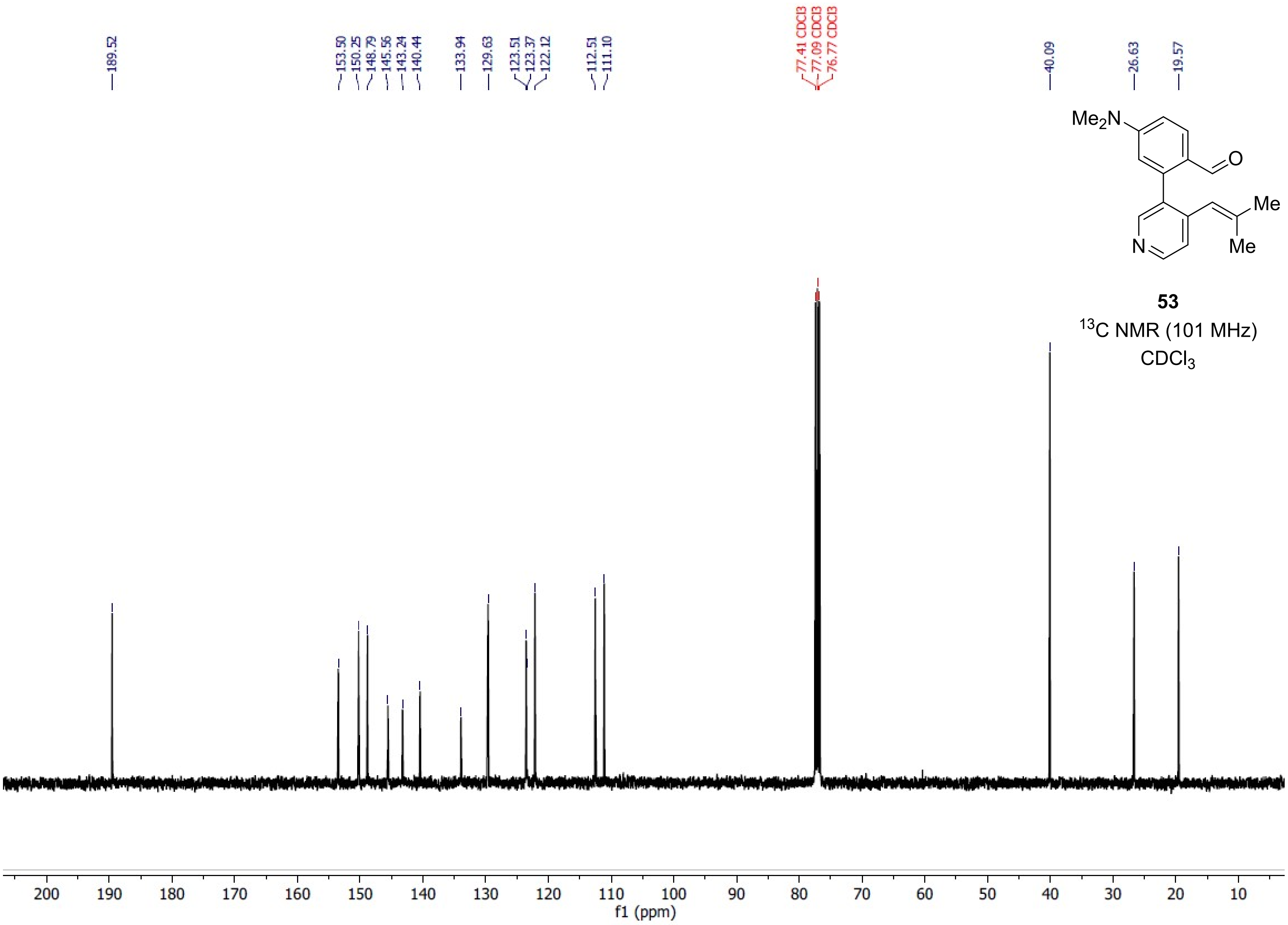

53

^{13}C NMR (101 MHz)

$CDCl_3$

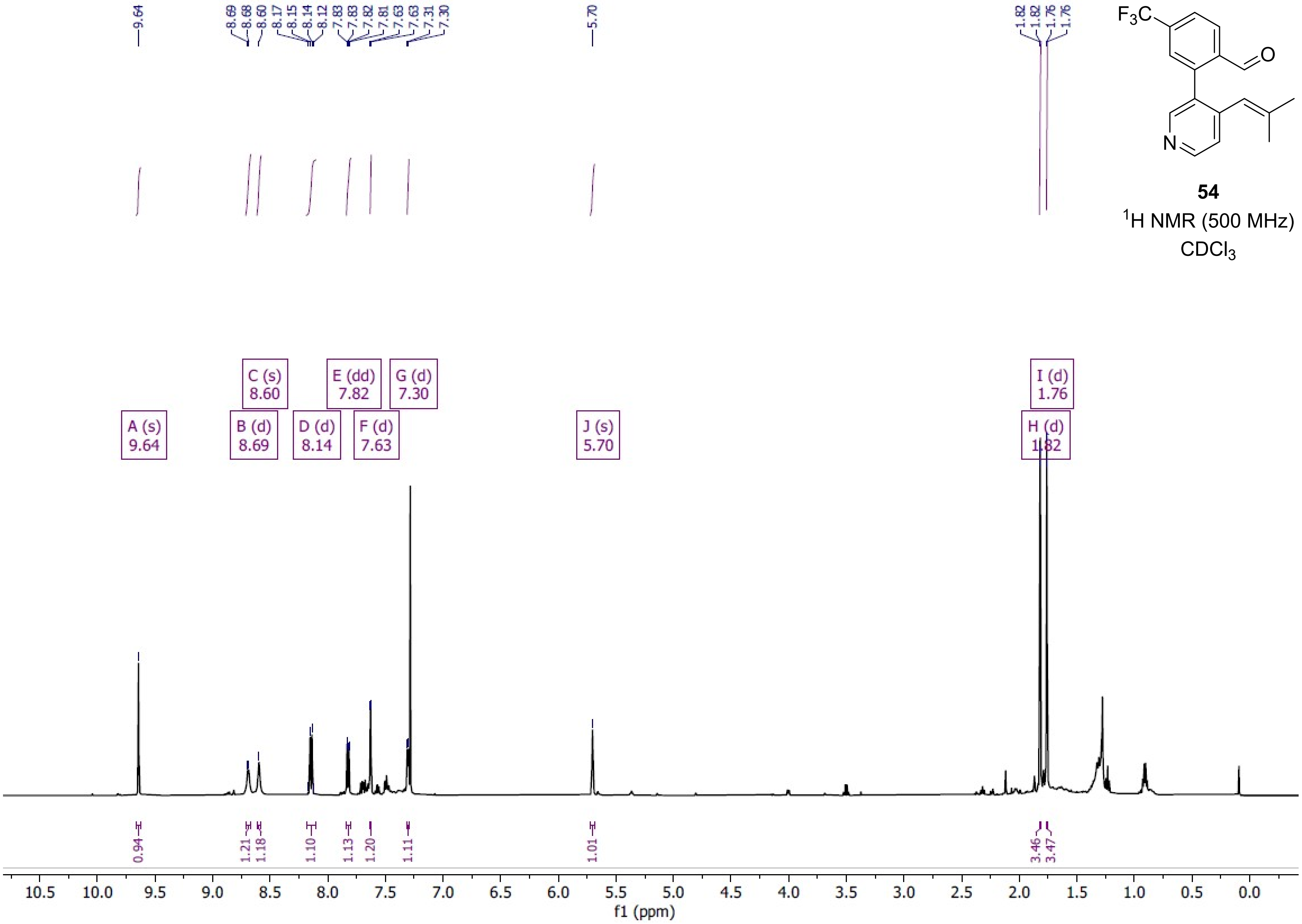

54

^{1}H NMR (500 MHz)
$CDCl_3$

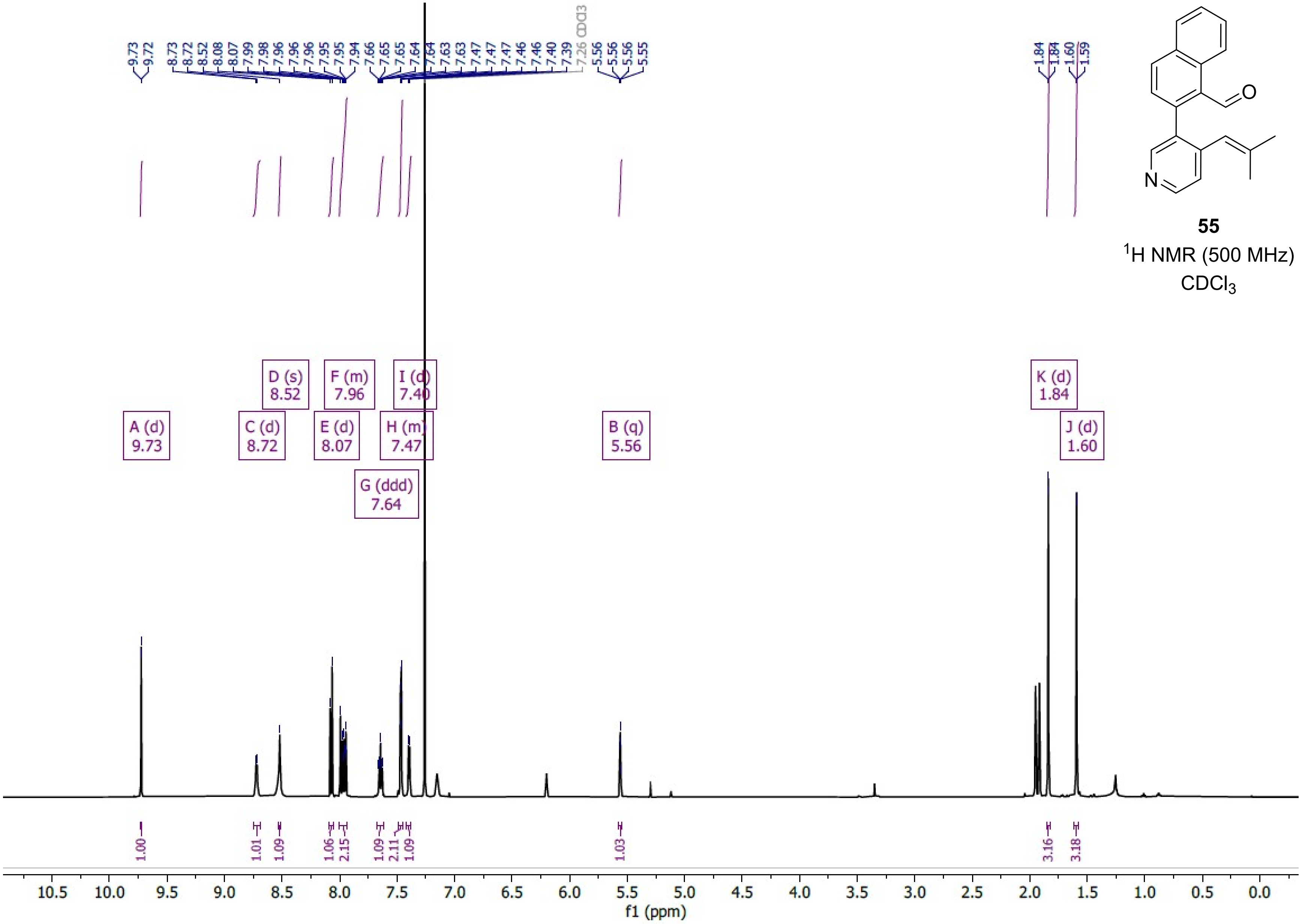

55
^{1}H NMR (500 MHz)
$CDCl_3$
A (d) 9.73
B (q) 5.56
C (d) 8.72
D (s) 8.52
E (d) 8.07
F (m) 7.96
G (ddd) 7.64
H (m) 7.47
I (d) 7.40
J (d) 1.60
K (d) 1.84
f1 (ppm)

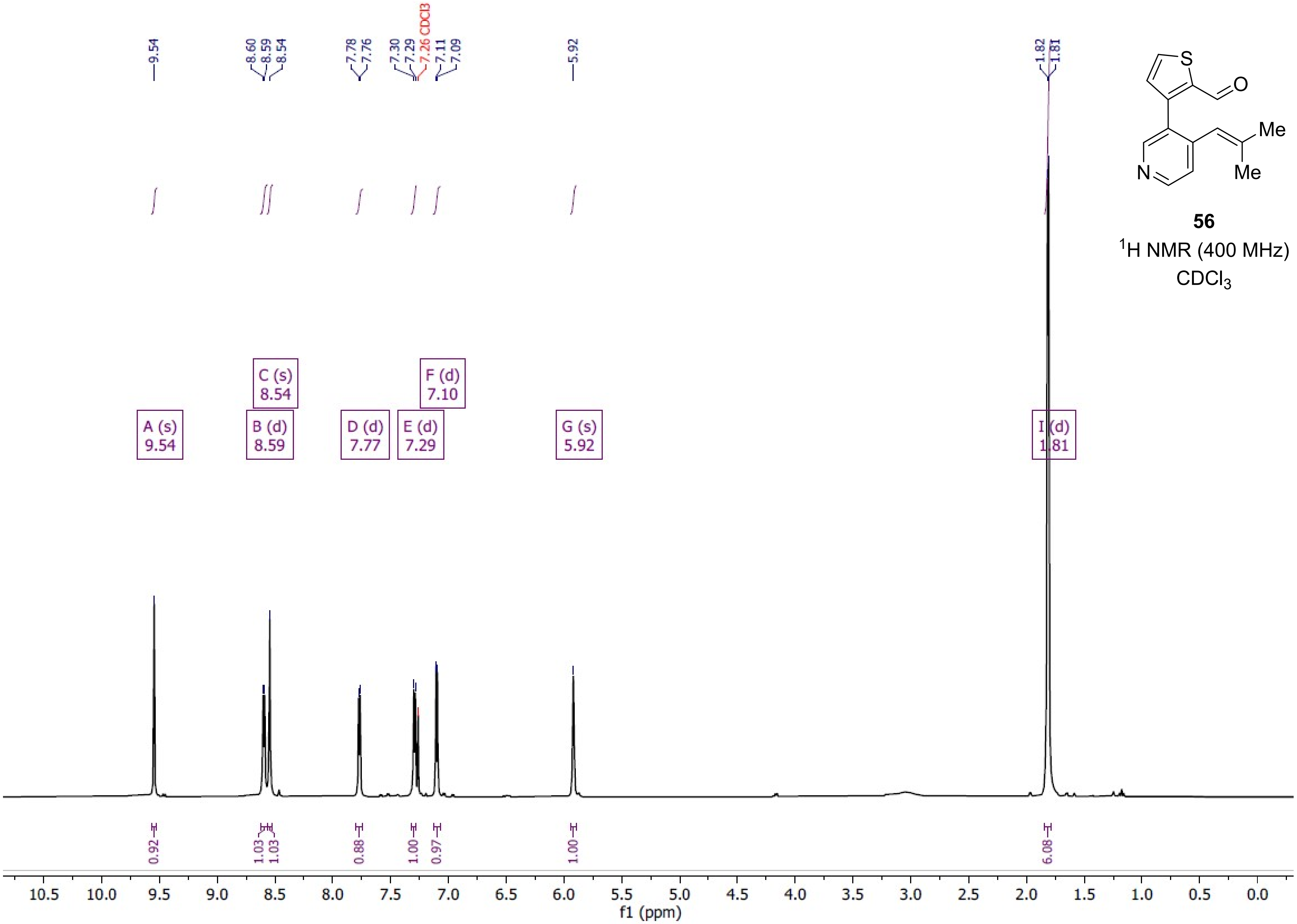

56

^{1}H NMR (400 MHz)

$CDCl_3$

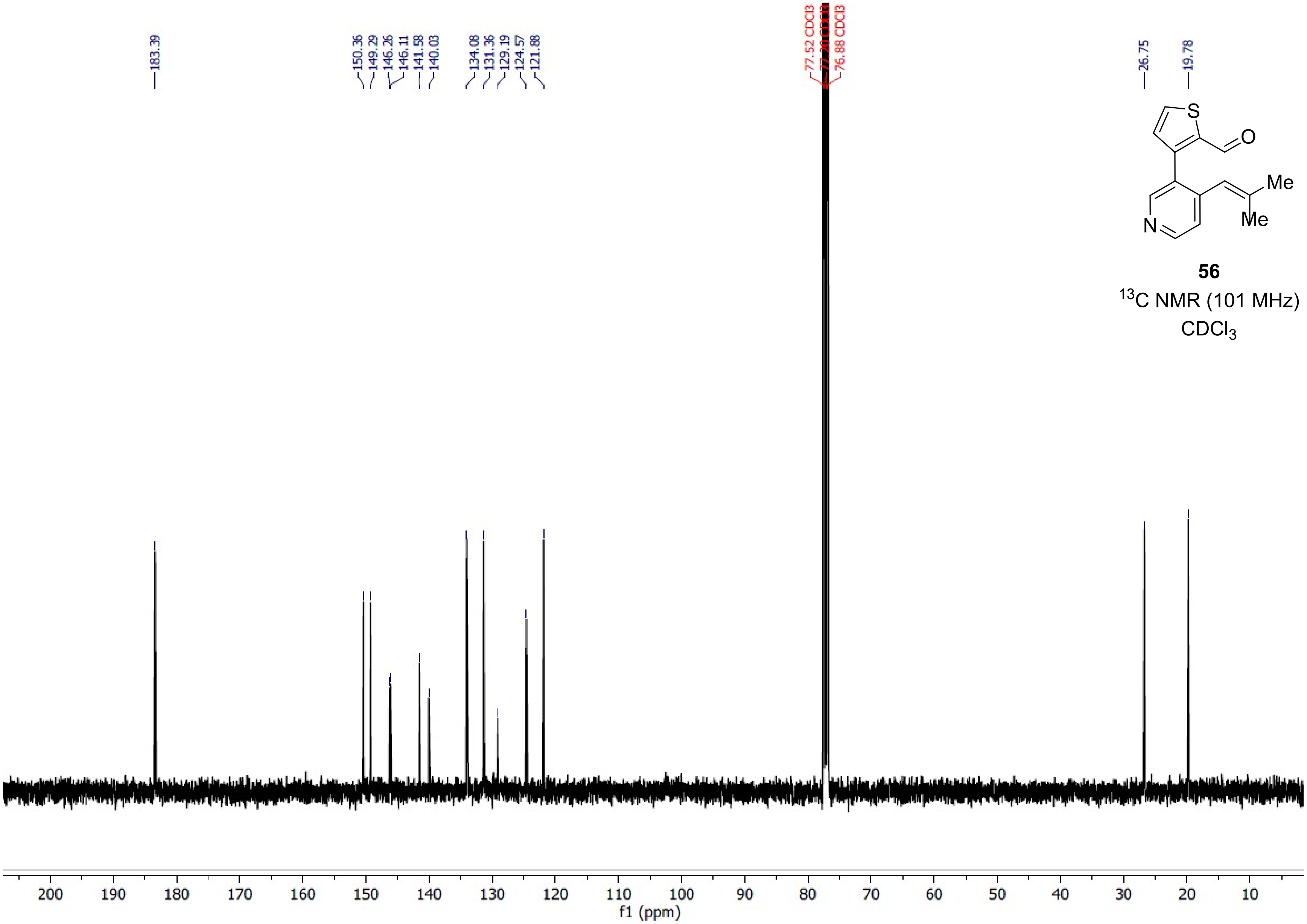

56

^{13}C NMR (101 MHz)

$CDCl_3$

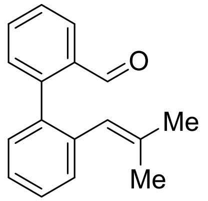

57

^{1}H NMR (500 MHz)

$CDCl_3$

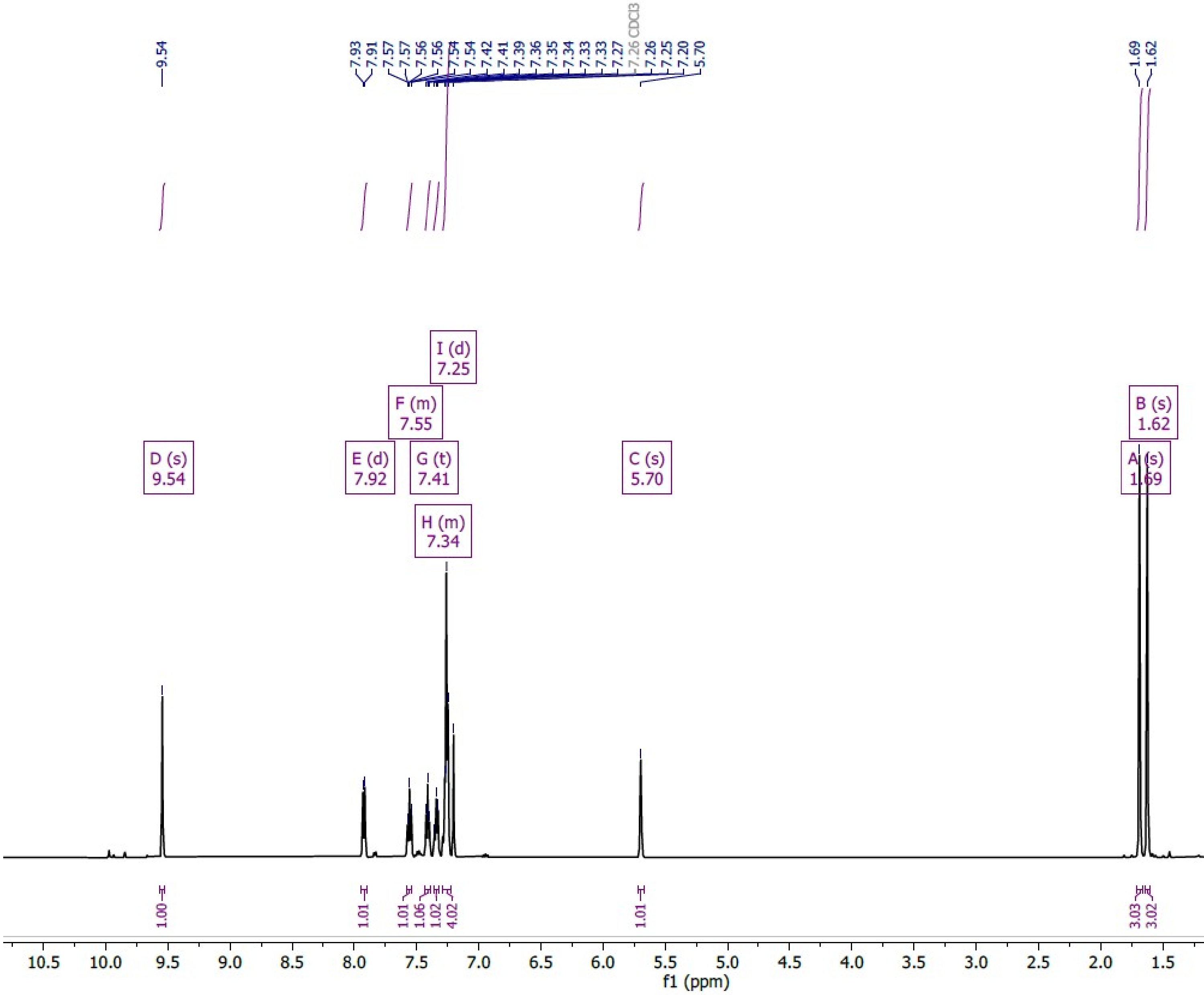

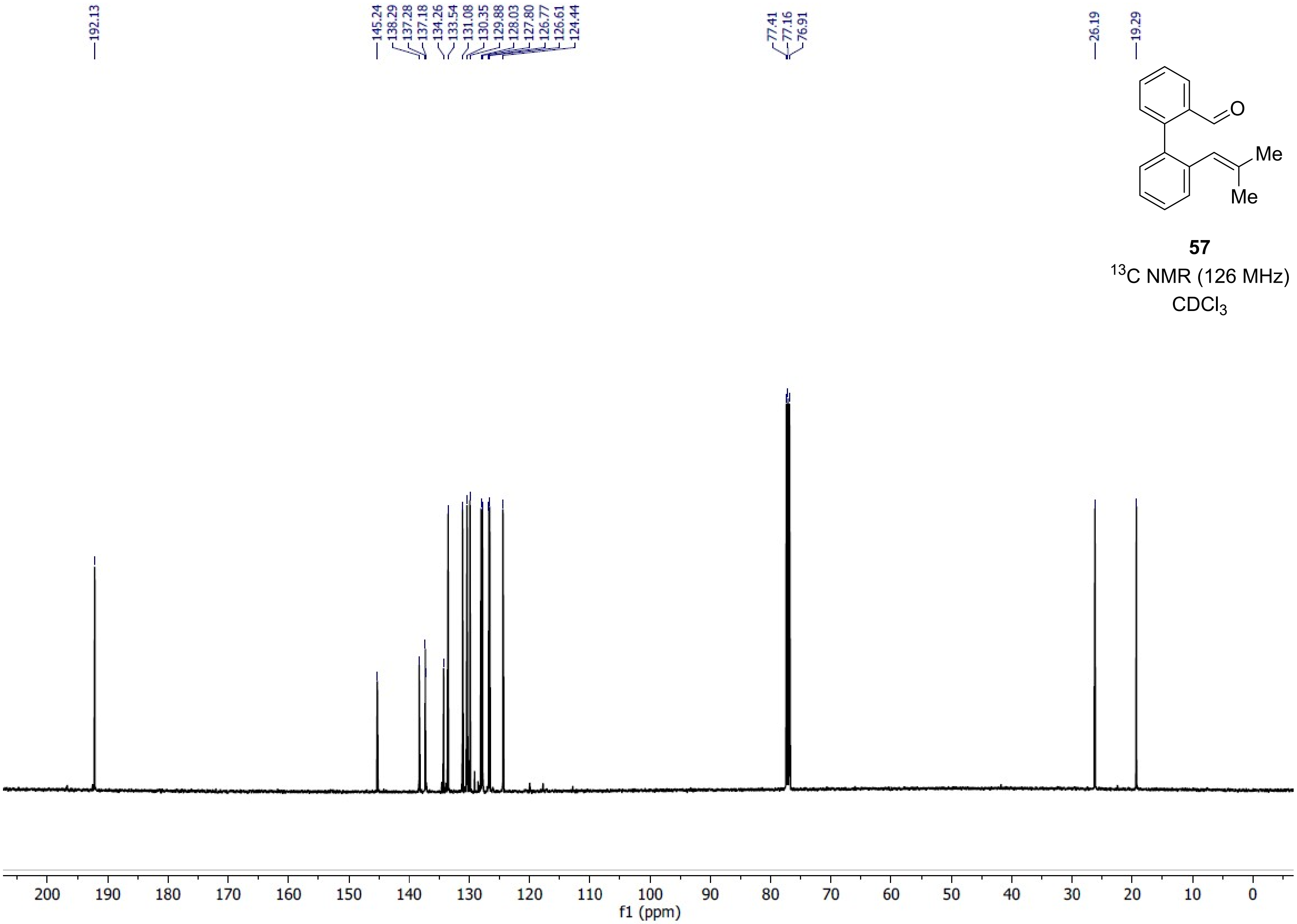

57

^{13}C NMR (126 MHz)

$CDCl_3$

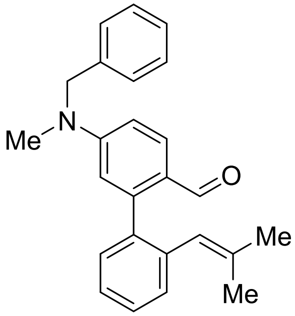

58

^{1}H NMR (500 MHz)

$CDCl_3$

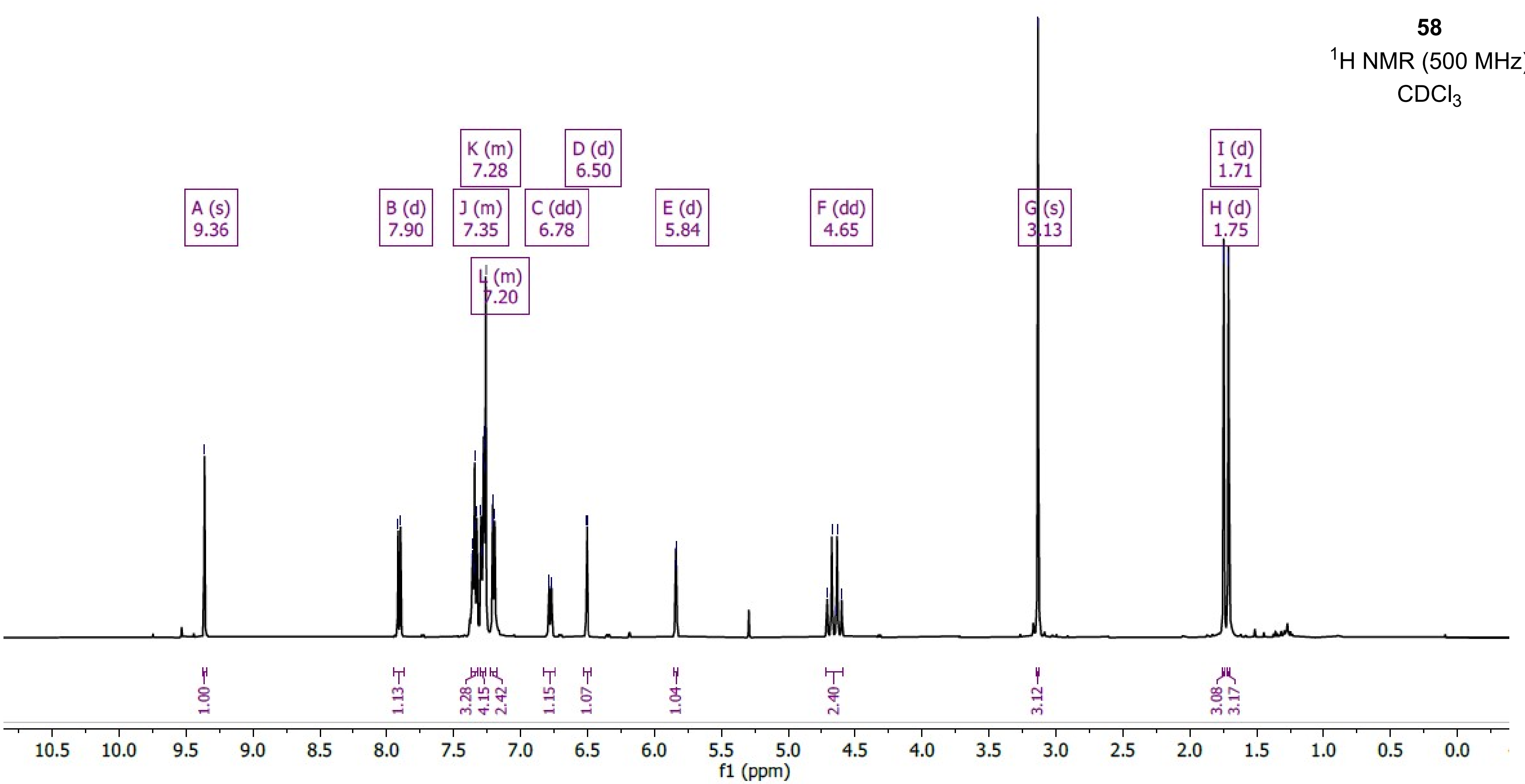

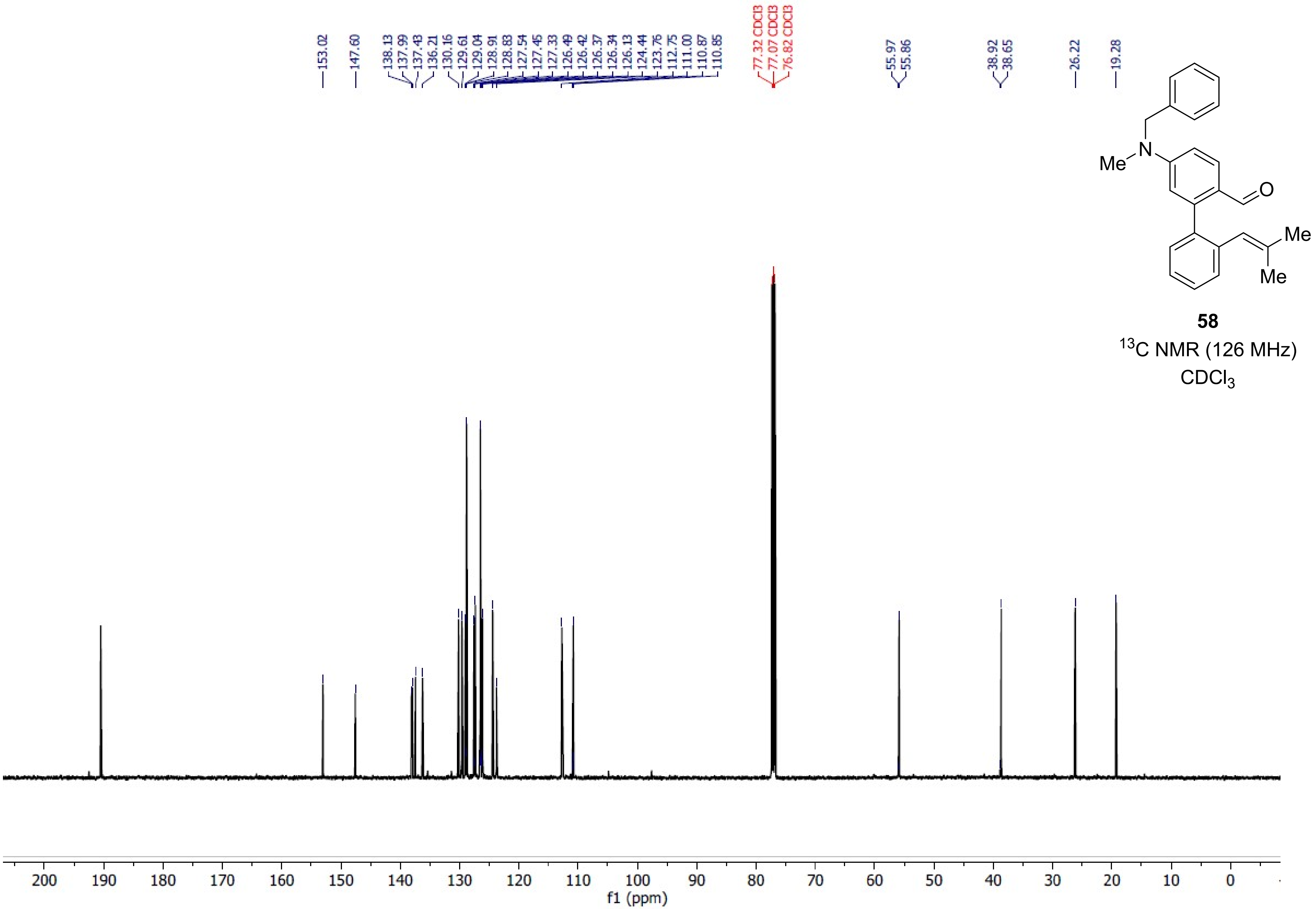

58

^{13}C NMR (126 MHz)

$CDCl_3$

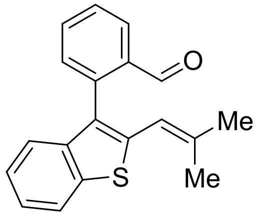

59

^{1}H NMR (400 MHz)

$CDCl_3$

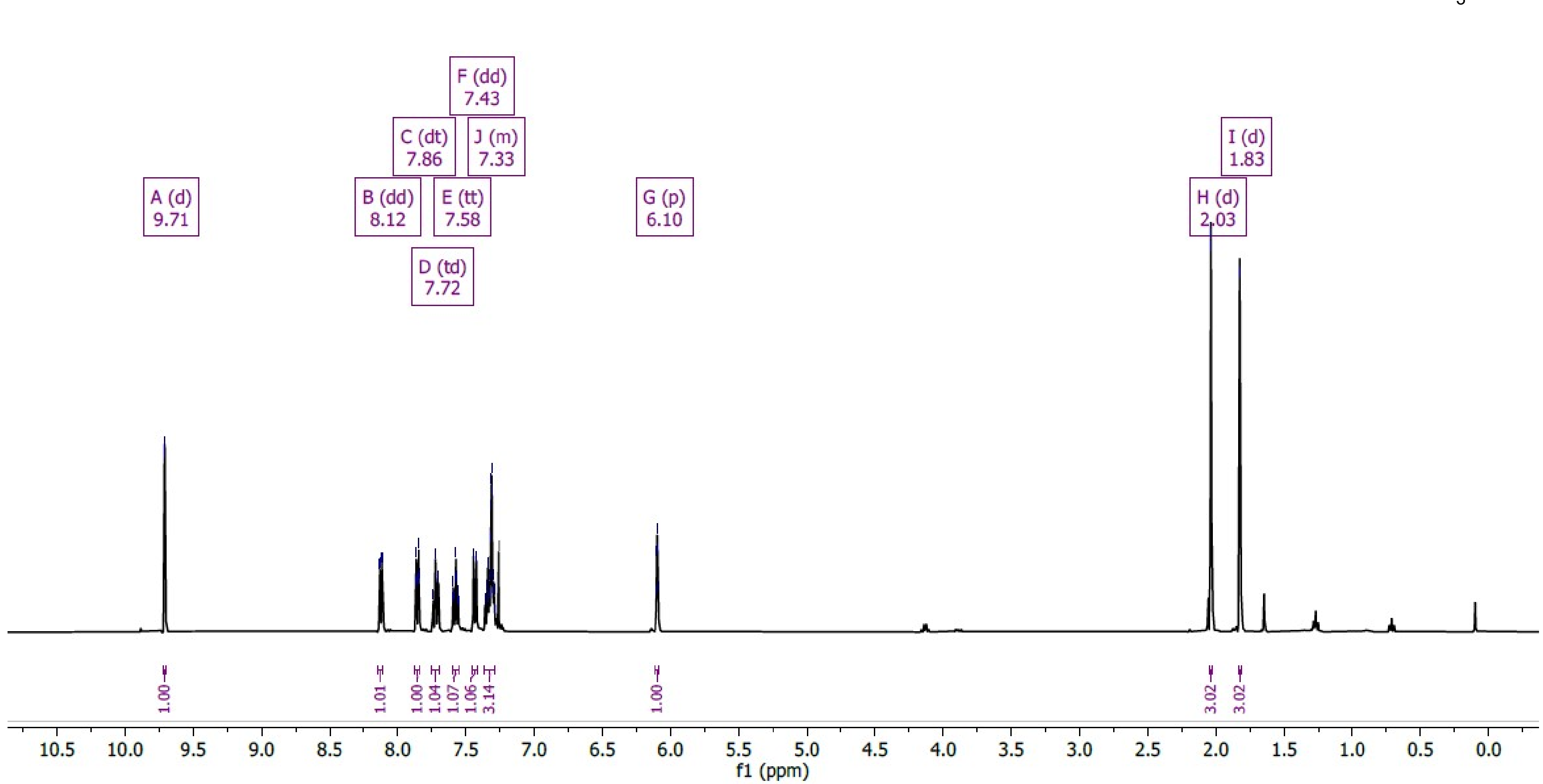

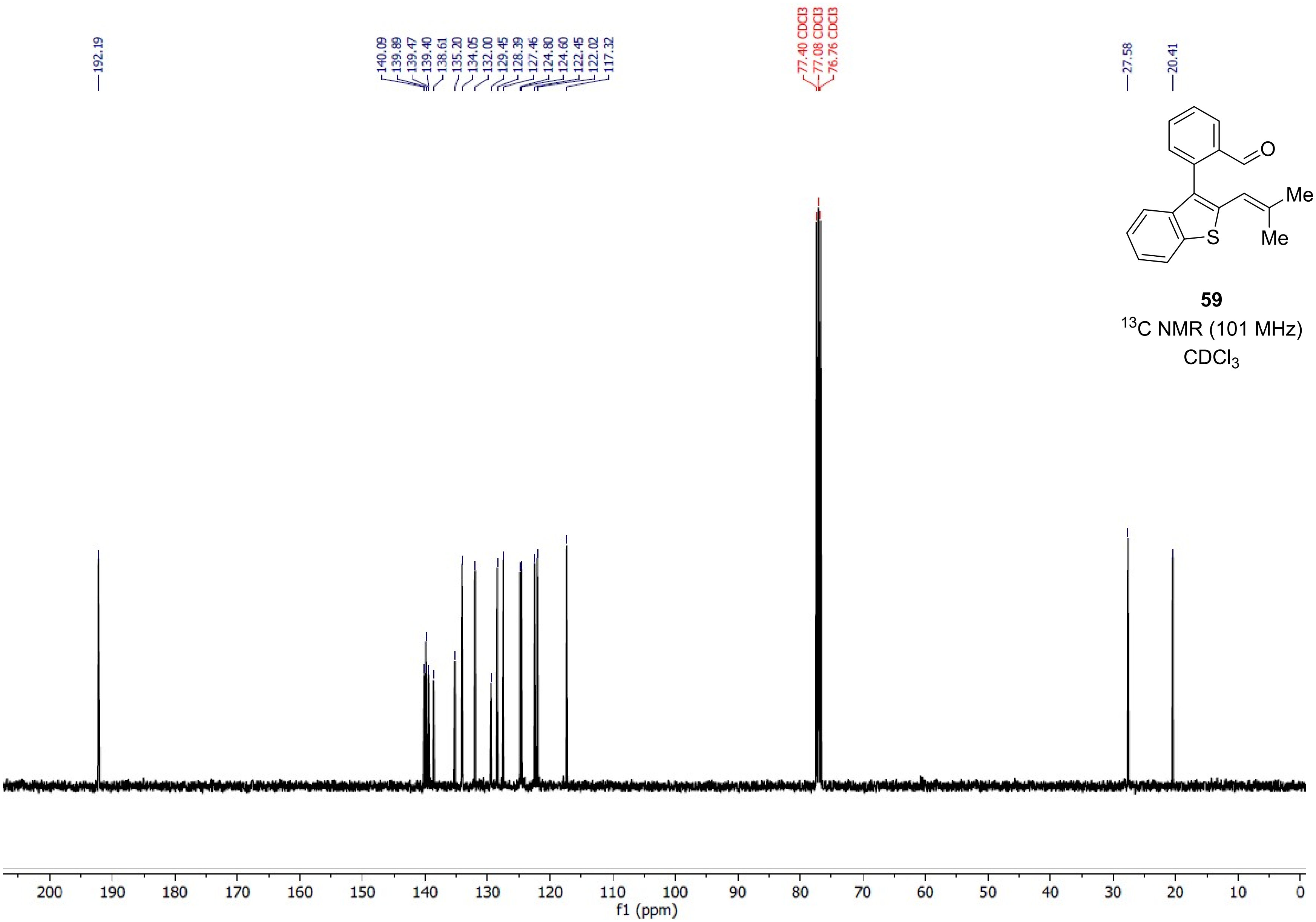

59

^{13}C NMR (101 MHz)

$CDCl_3$

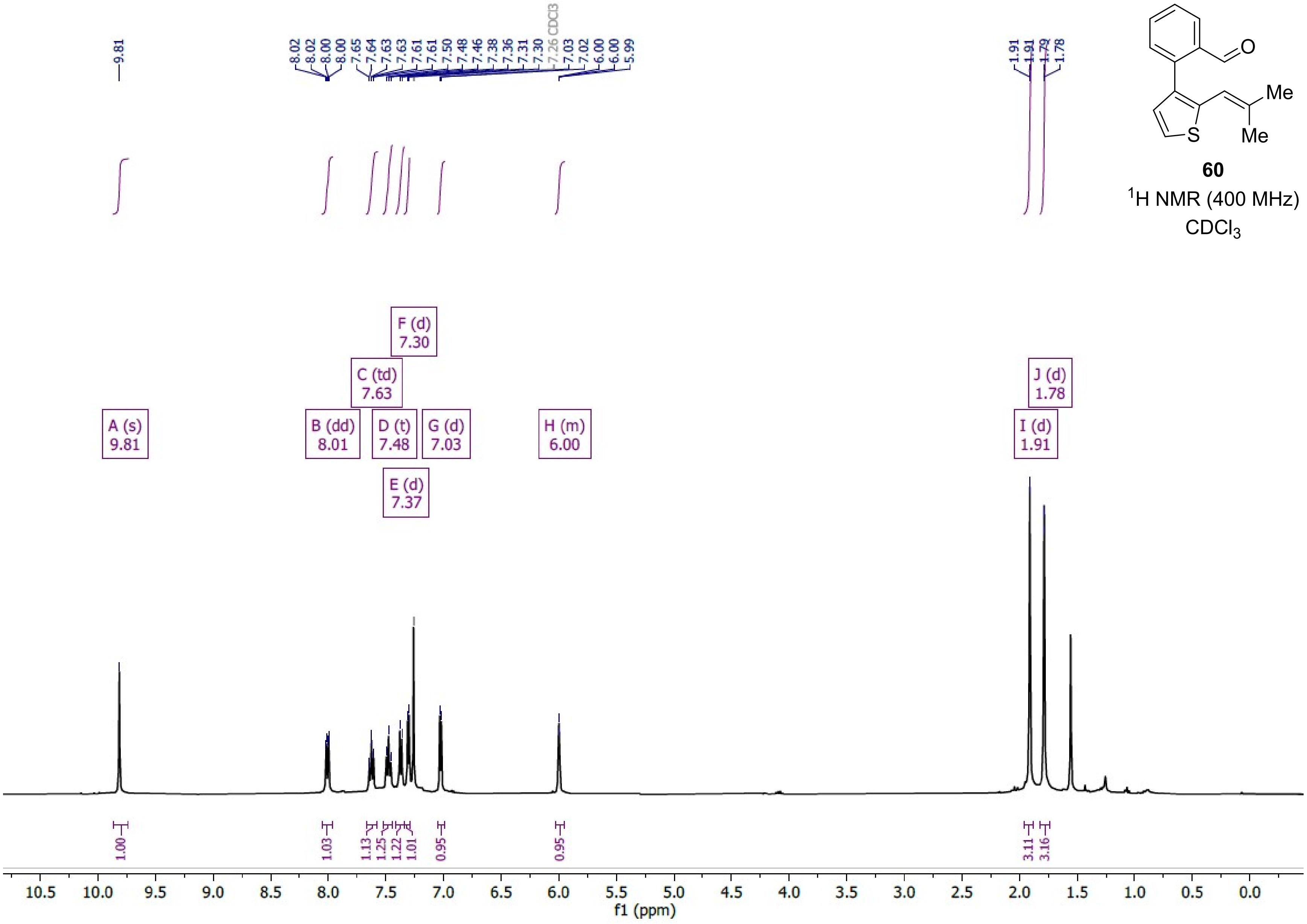

60
^{1}H NMR (400 MHz)
$CDCl_3$
A (s) 9.81
B (dd) 8.01
C (td) 7.63
D (t) 7.48
E (d) 7.37
F (d) 7.30
G (d) 7.03
H (m) 6.00
I (d) 1.91
J (d) 1.78
f1 (ppm)

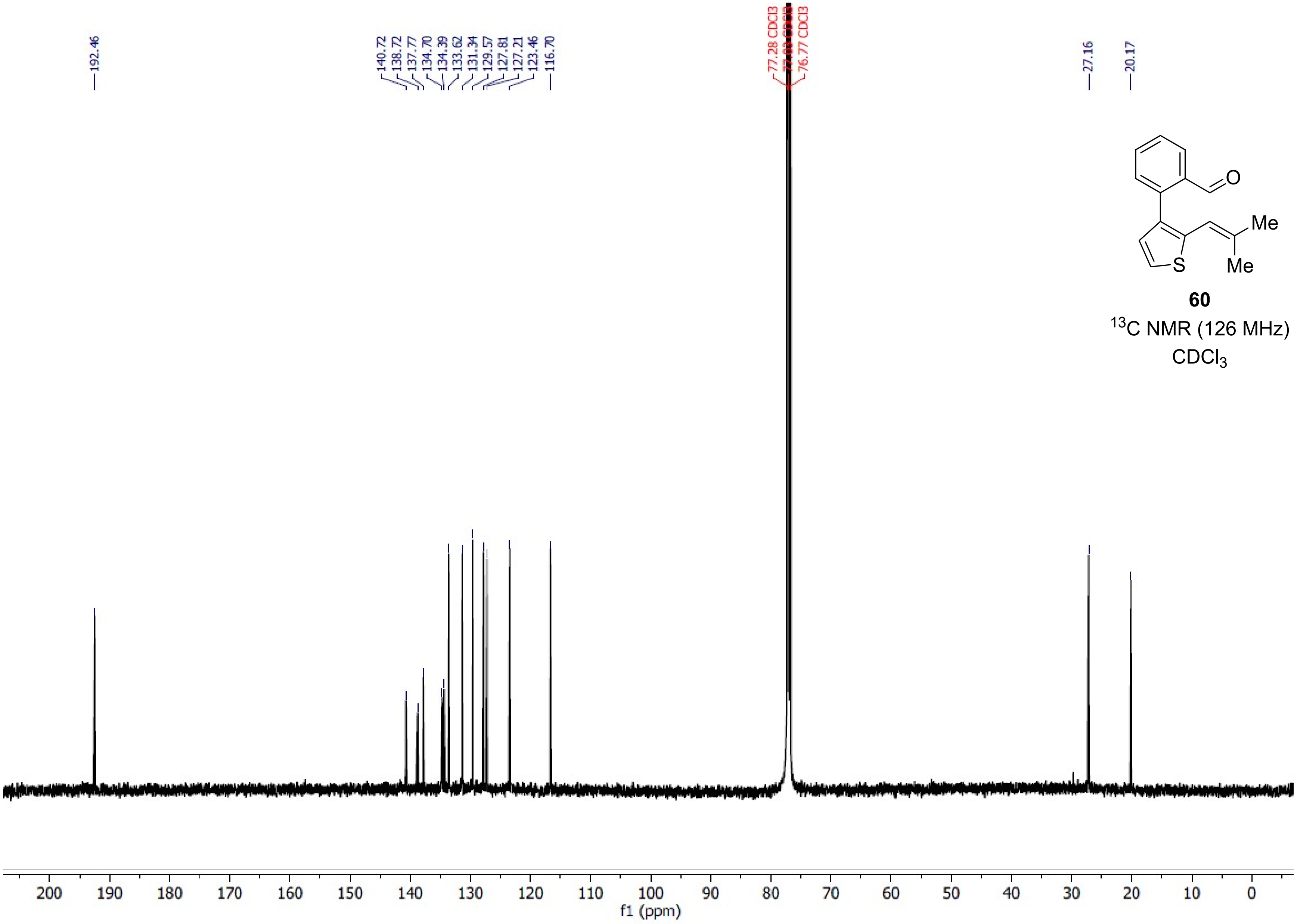
192.46
140.72
138.72
137.77
134.70
134.39
133.62
131.34
129.57
127.81
127.21
123.46
116.70
77.28 CDCl3
76.77 CDCl3
27.16
20.17
O
Me
Me
S
60
^{13}C NMR (126 MHz)
$CDCl_3$
200
190
180
170
160
150
140
130
120
110
100
90
80
70
60
50
40
30
20
10
0
f1 (ppm)

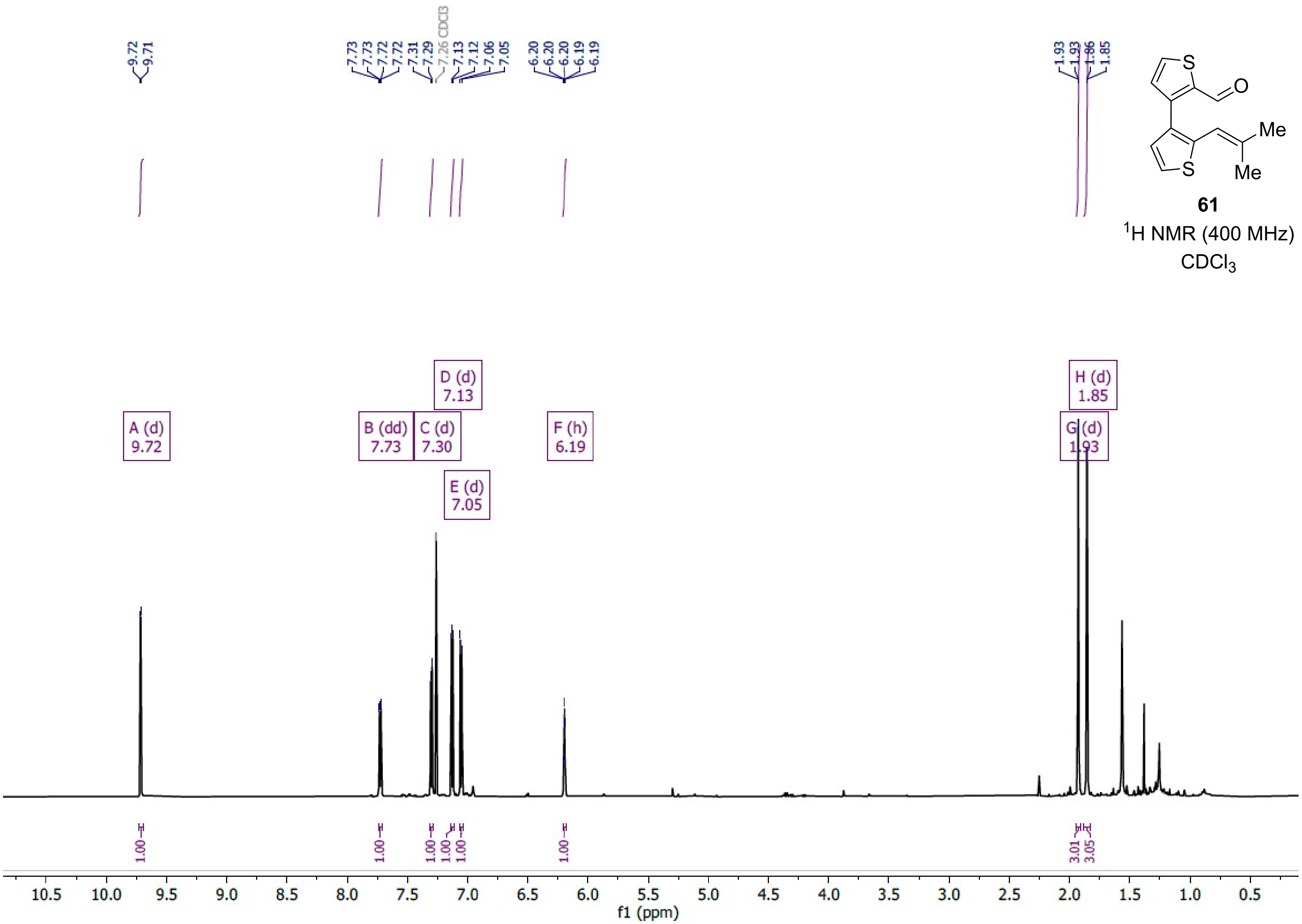

61

^{1}H NMR (400 MHz)

$CDCl_3$

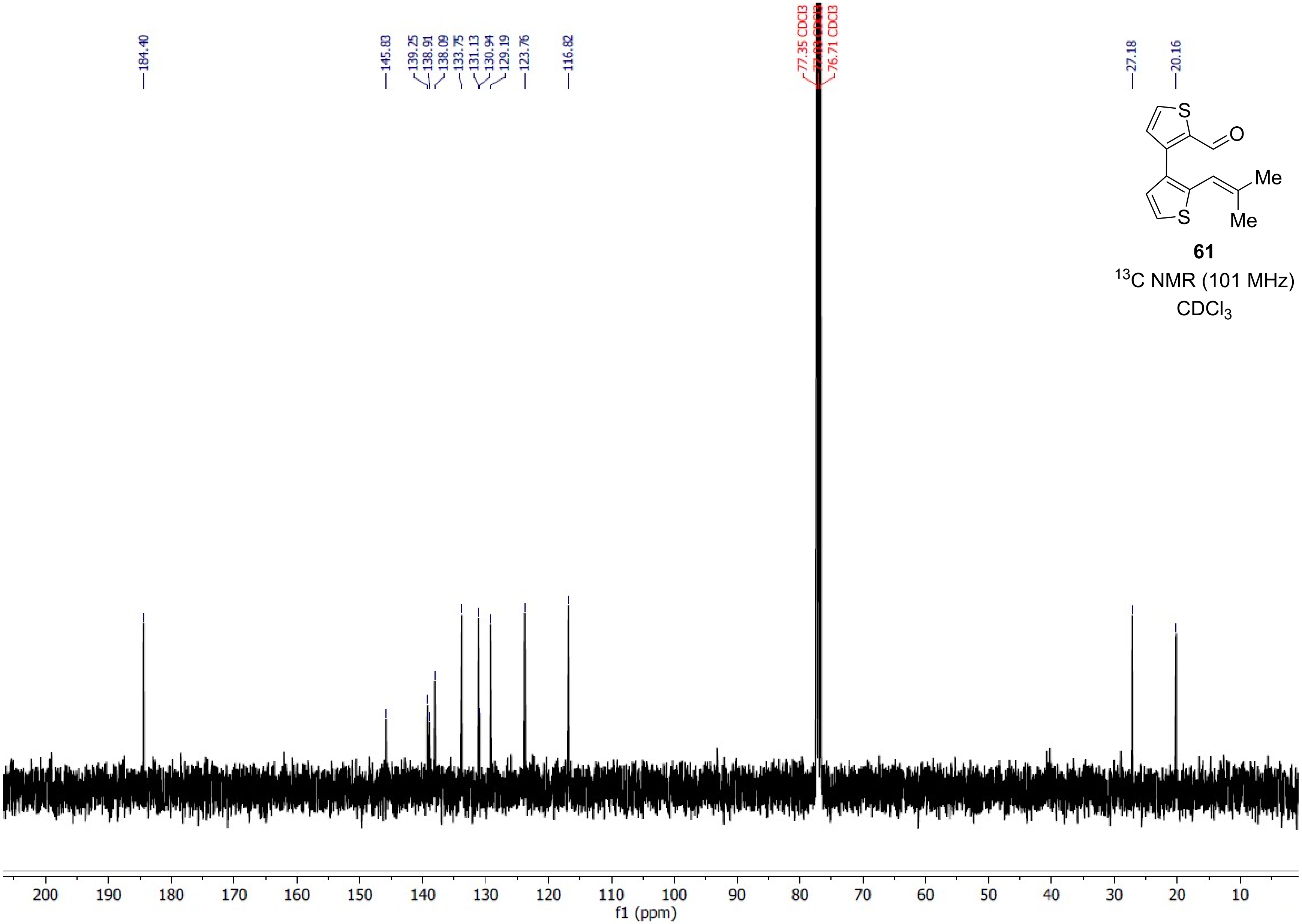
184.40
145.83
139.25
138.91
138.09
133.75
131.13
130.94
129.19
123.76
116.82
77.35 CDCl3
76.71 CDCl3
27.18
20.16
S
O
Me
Me
61
^{13}C NMR (101 MHz)
$CDCl_3$
200 190 180 170 160 150 140 130 120 110 100 90 80 70 60 50 40 30 20 10
f1 (ppm)

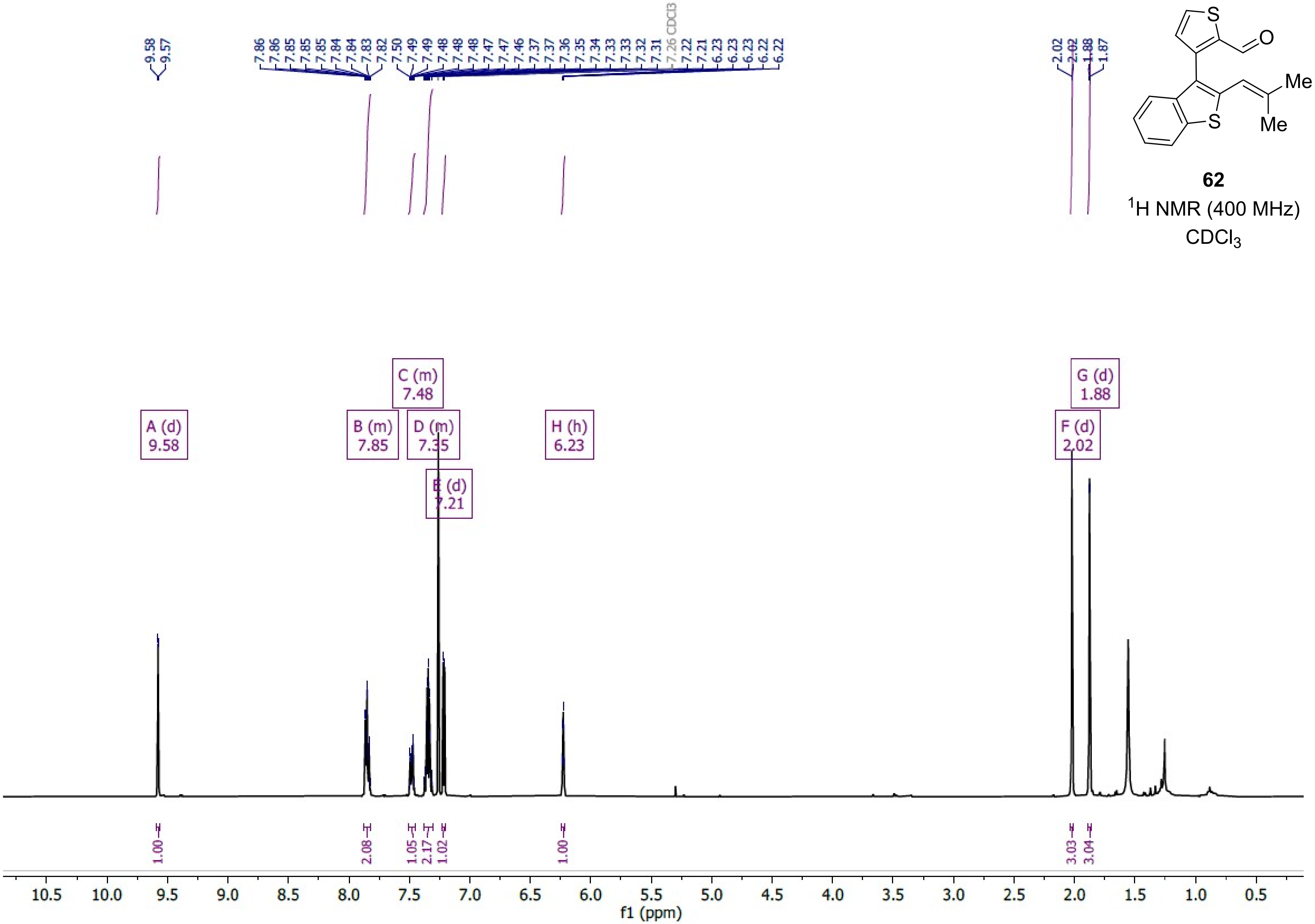

62

^{1}H NMR (400 MHz)

$CDCl_3$

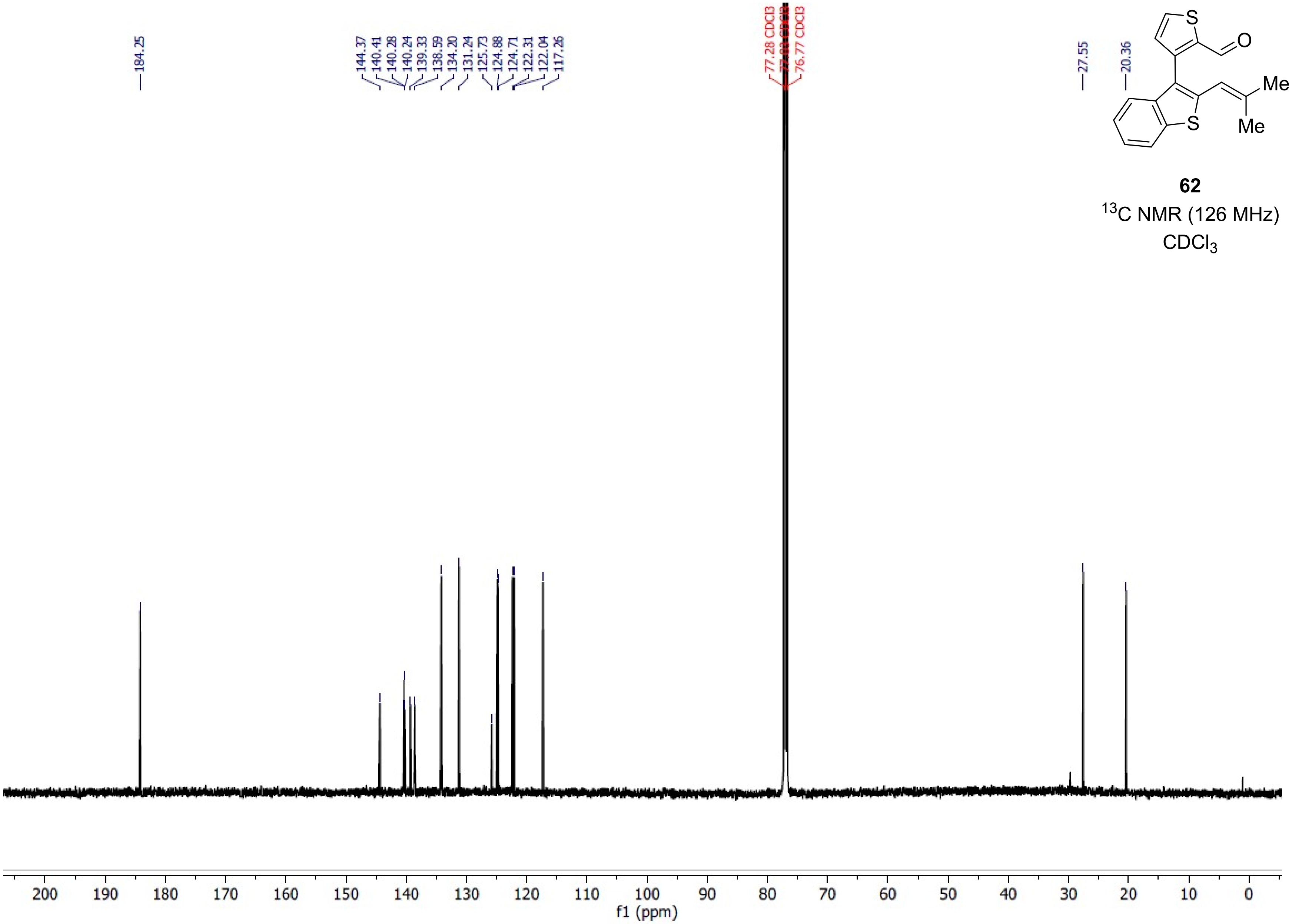

62

^{13}C NMR (126 MHz)

$CDCl_3$

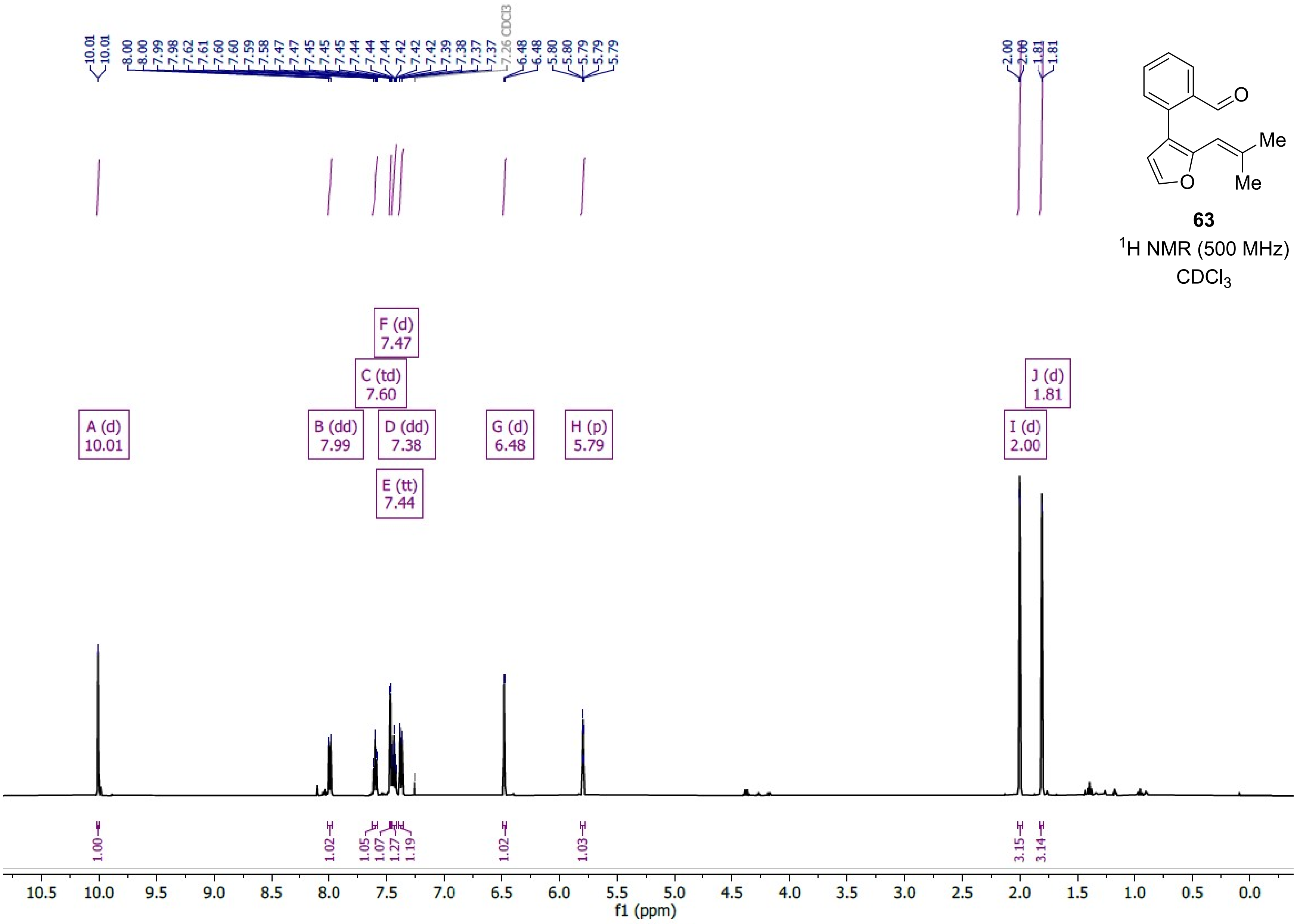

63

^{1}H NMR (500 MHz)

$CDCl_3$

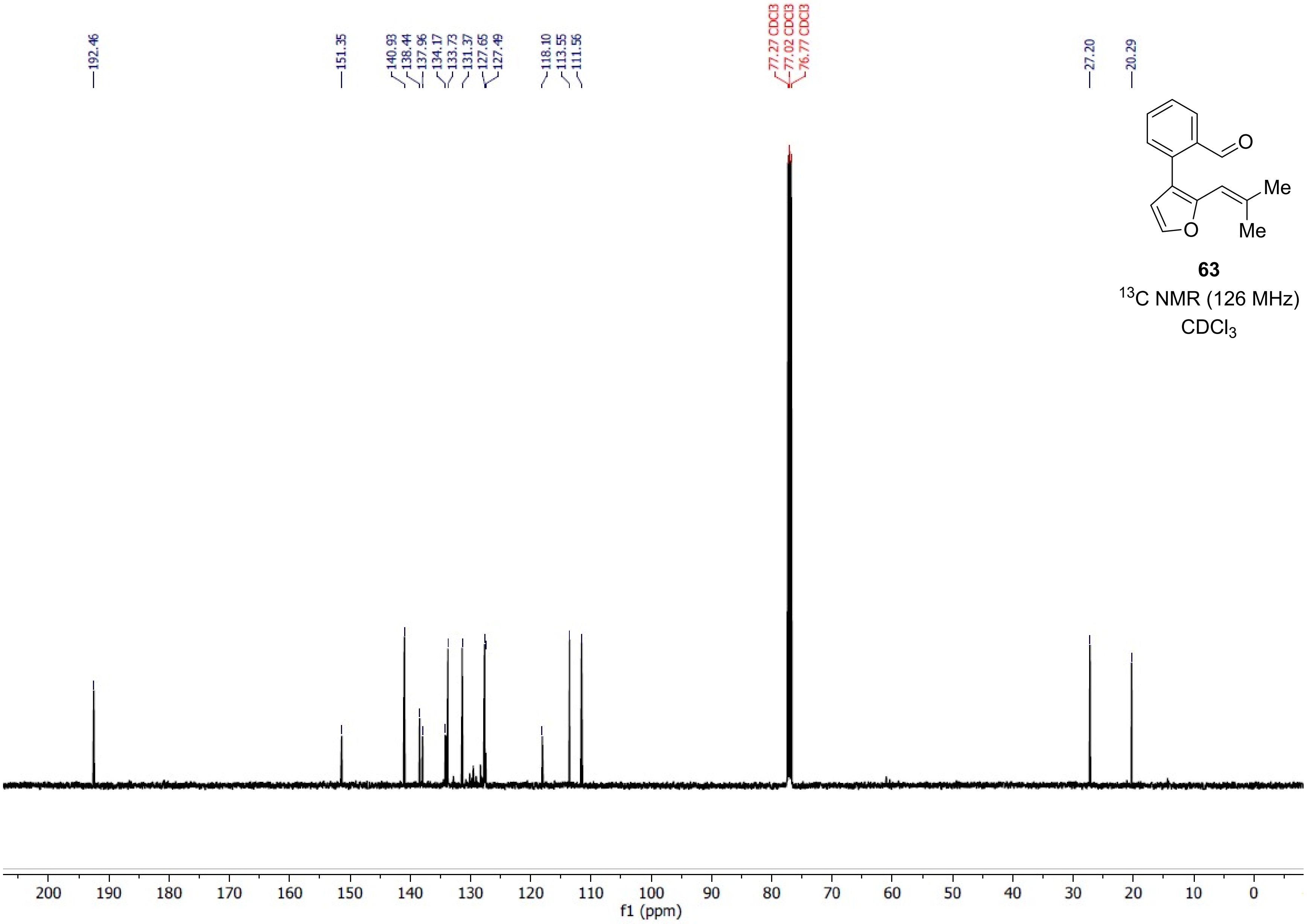

63

^{13}C NMR (126 MHz)

$CDCl_3$

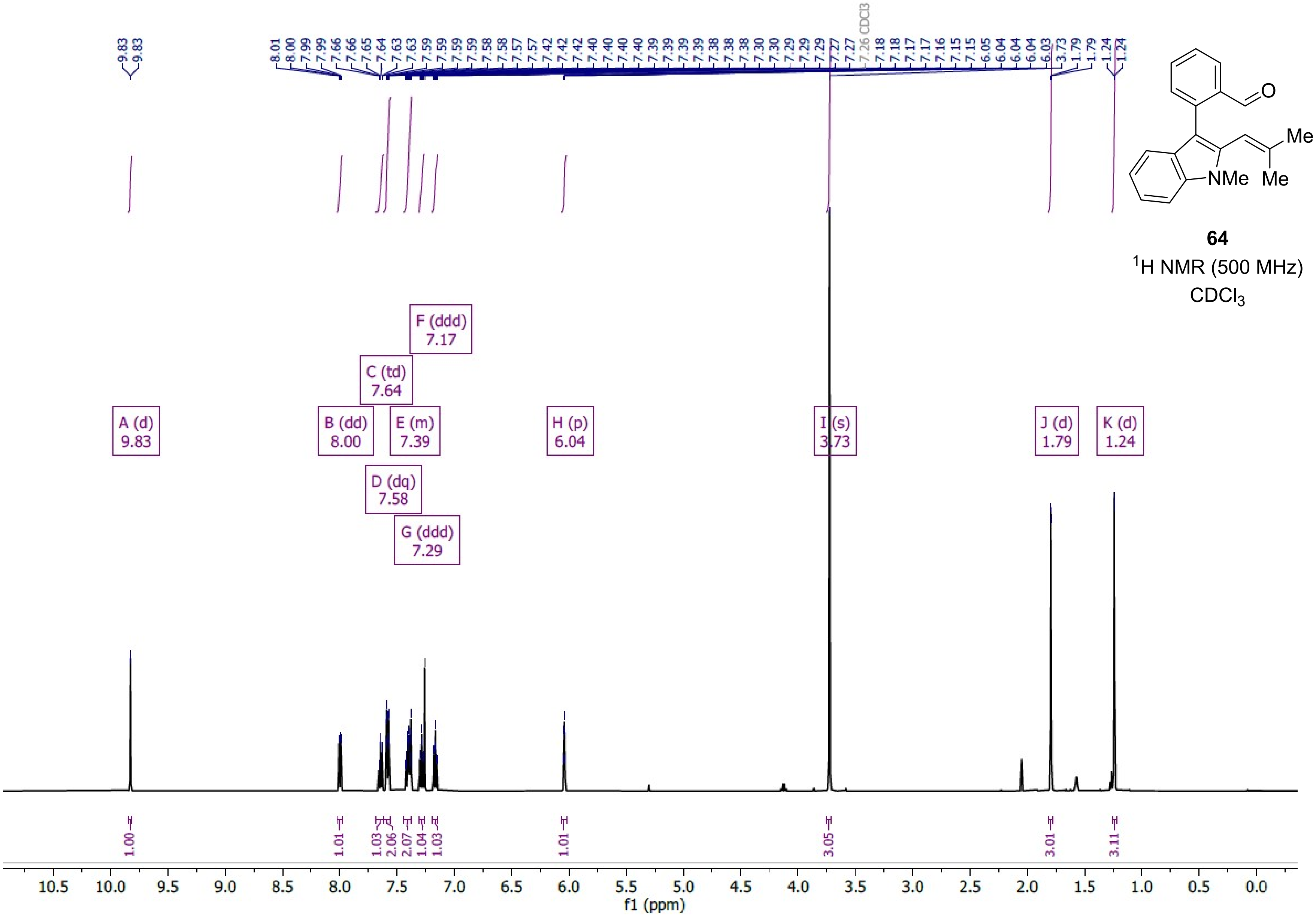

64

^{1}H NMR (500 MHz)

$CDCl_3$

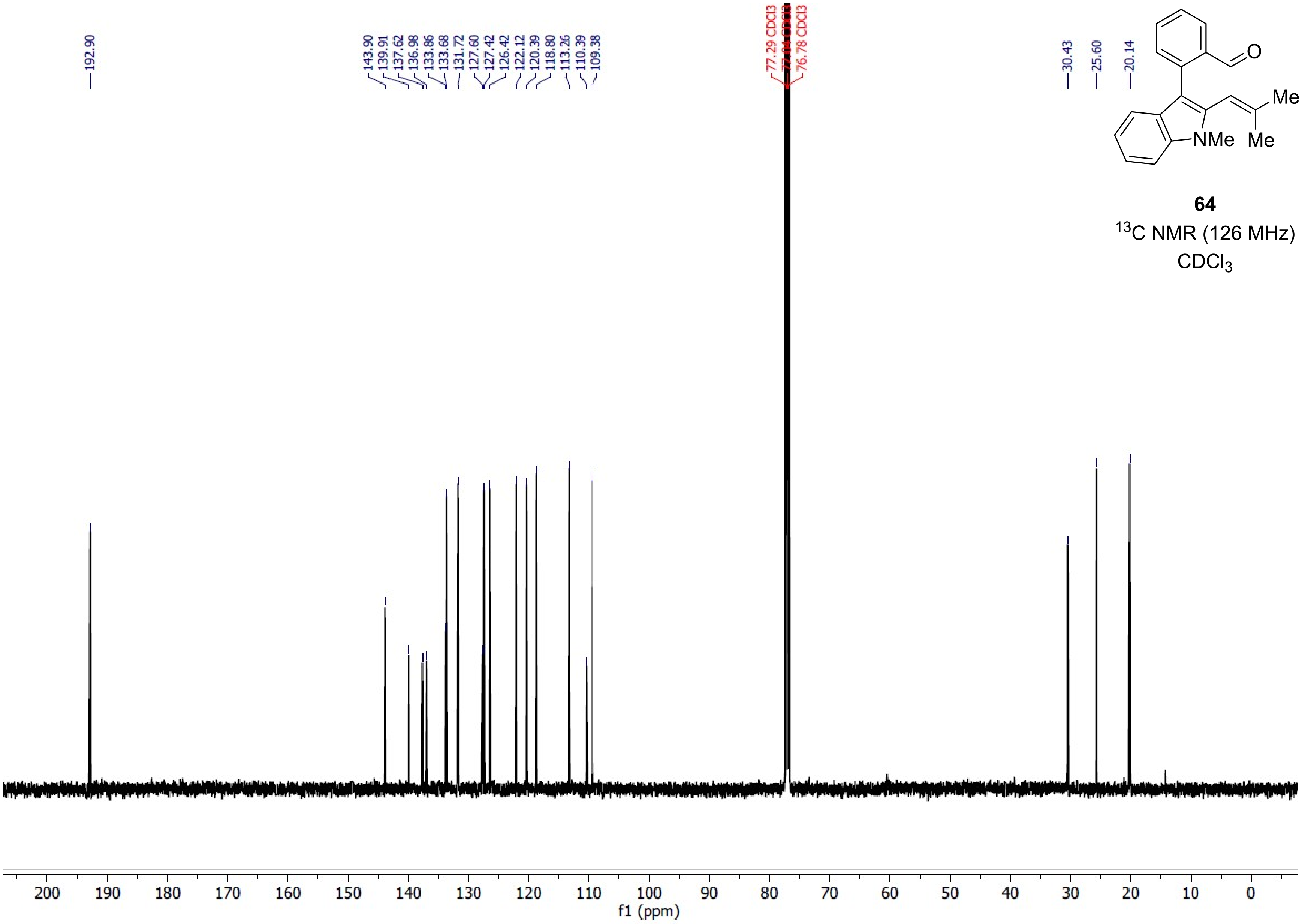

64

^{13}C NMR (126 MHz)

$CDCl_3$

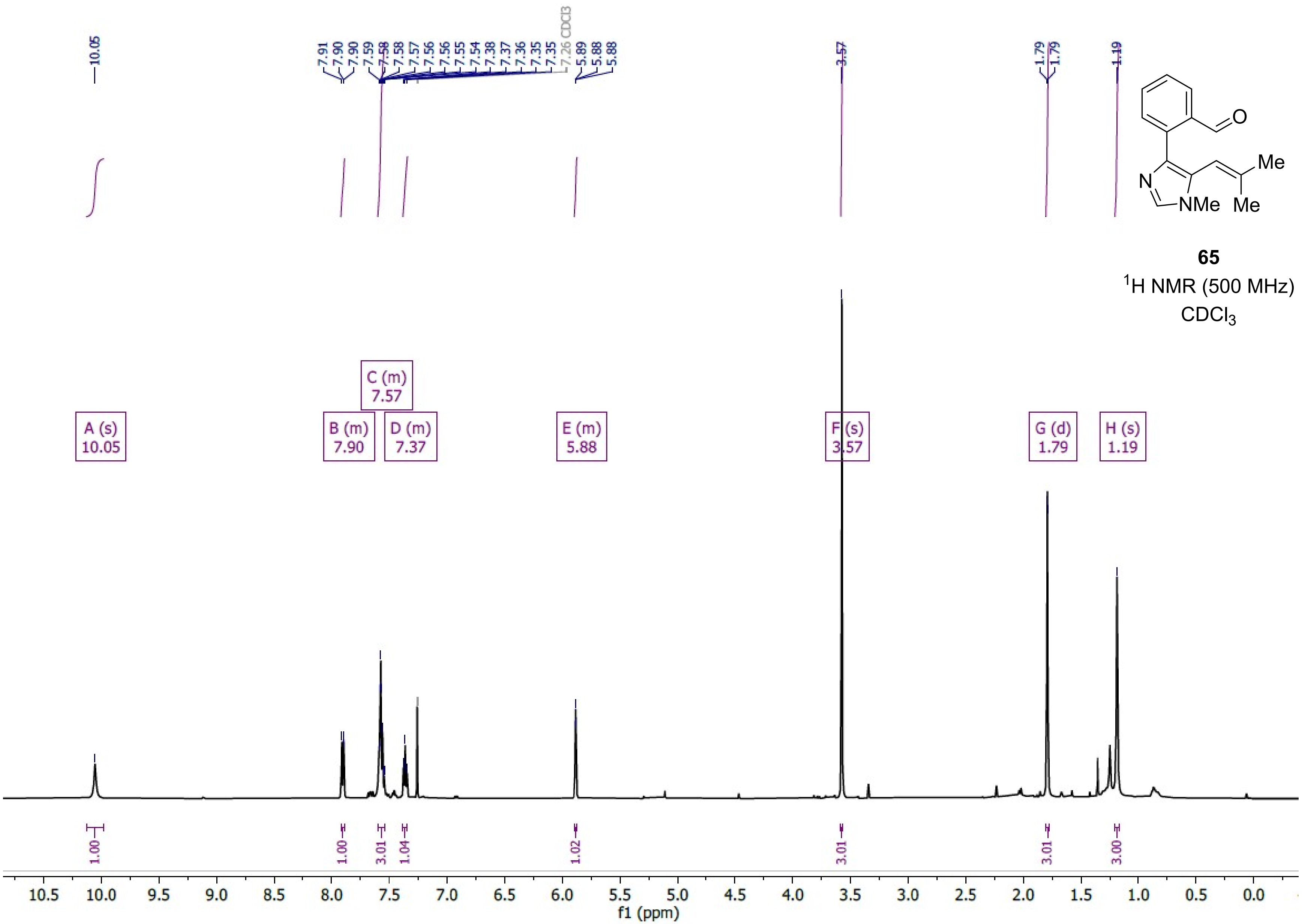

65

^{1}H NMR (500 MHz)

$CDCl_3$

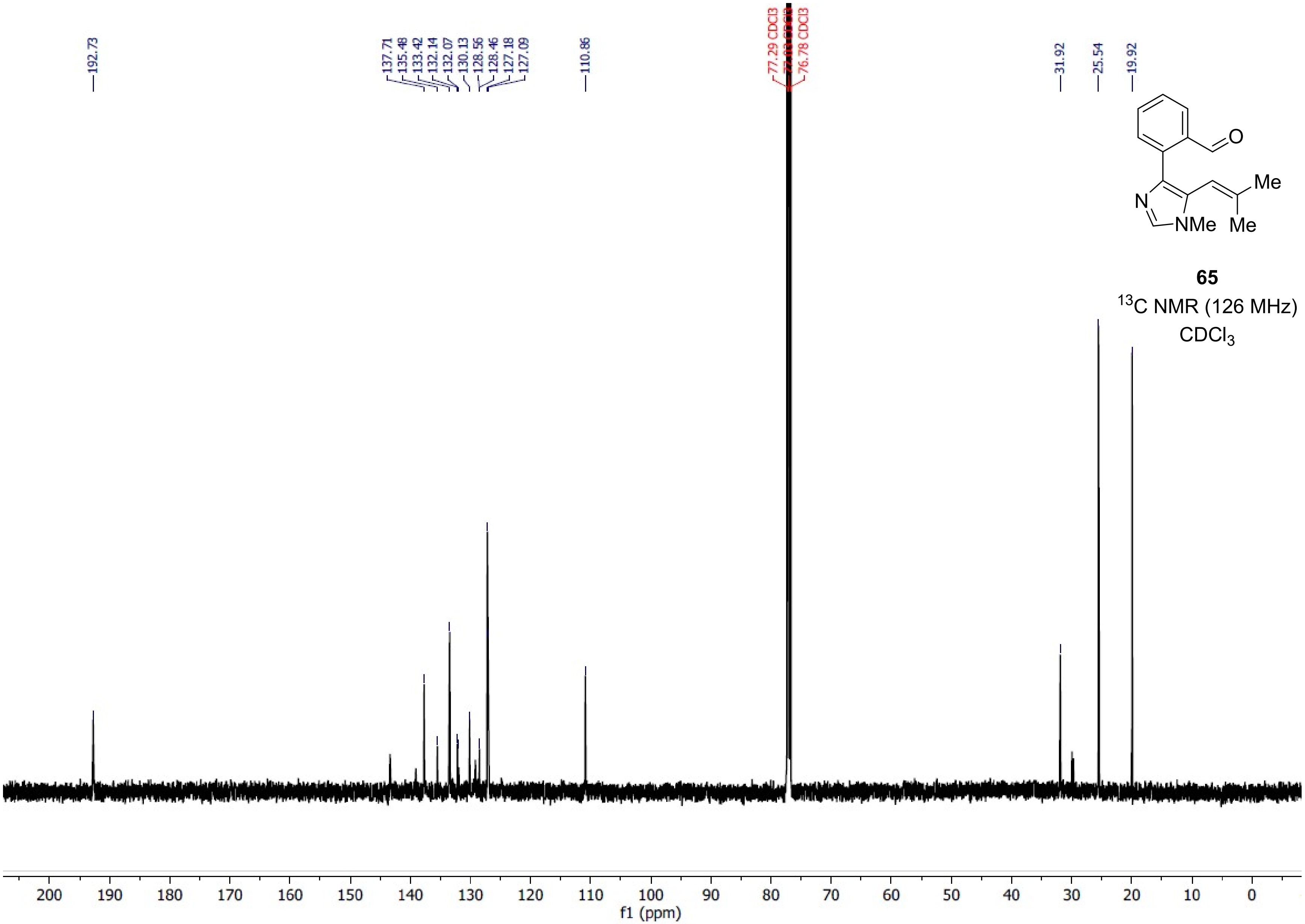

65

^{13}C NMR (126 MHz)

$CDCl_3$

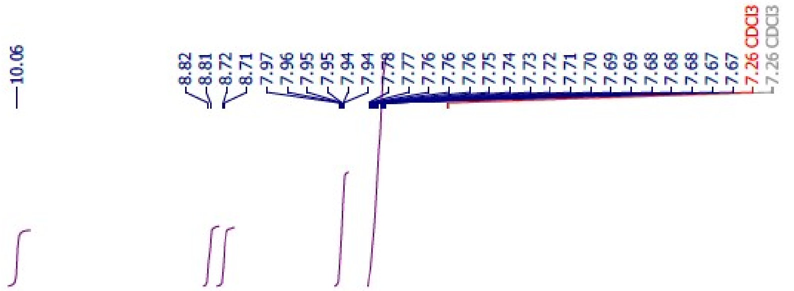
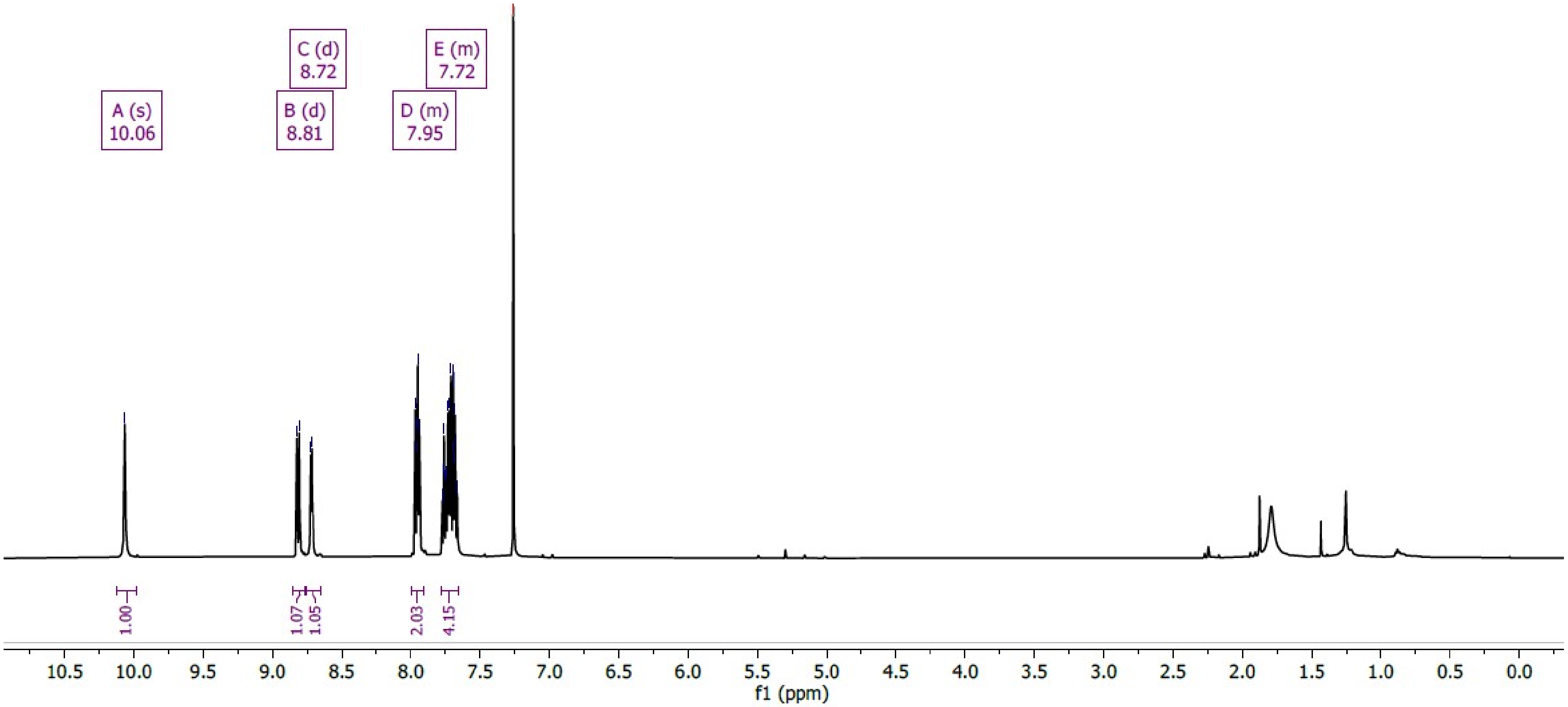

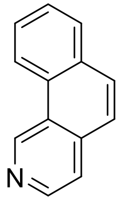

8

^{1}H NMR (500 MHz)

$CDCl_3$

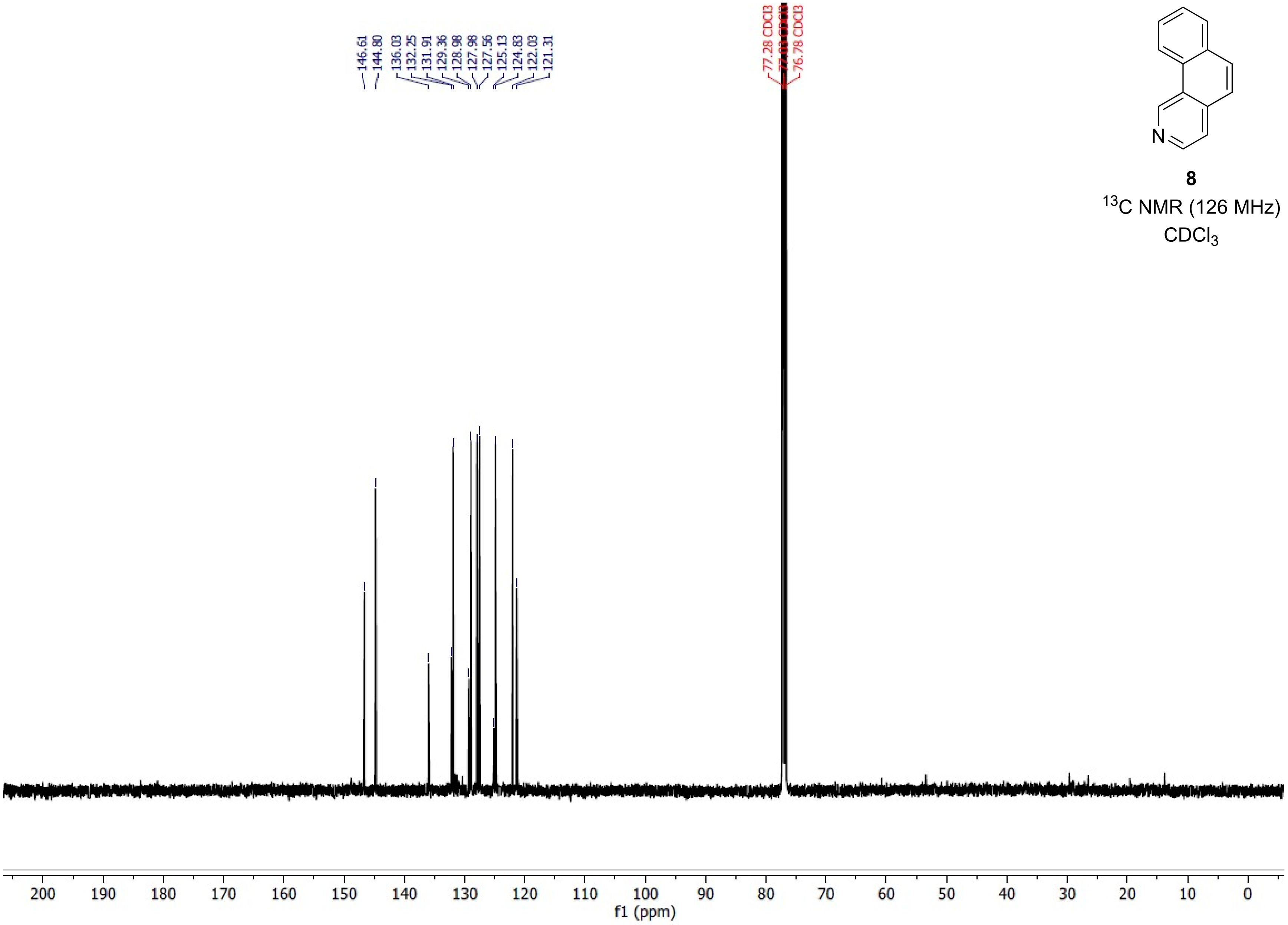

8

^{13}C NMR (126 MHz)

$CDCl_3$

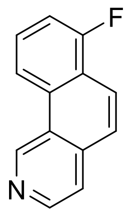

12

^1H NMR (500 MHz)

$CDCl_3$

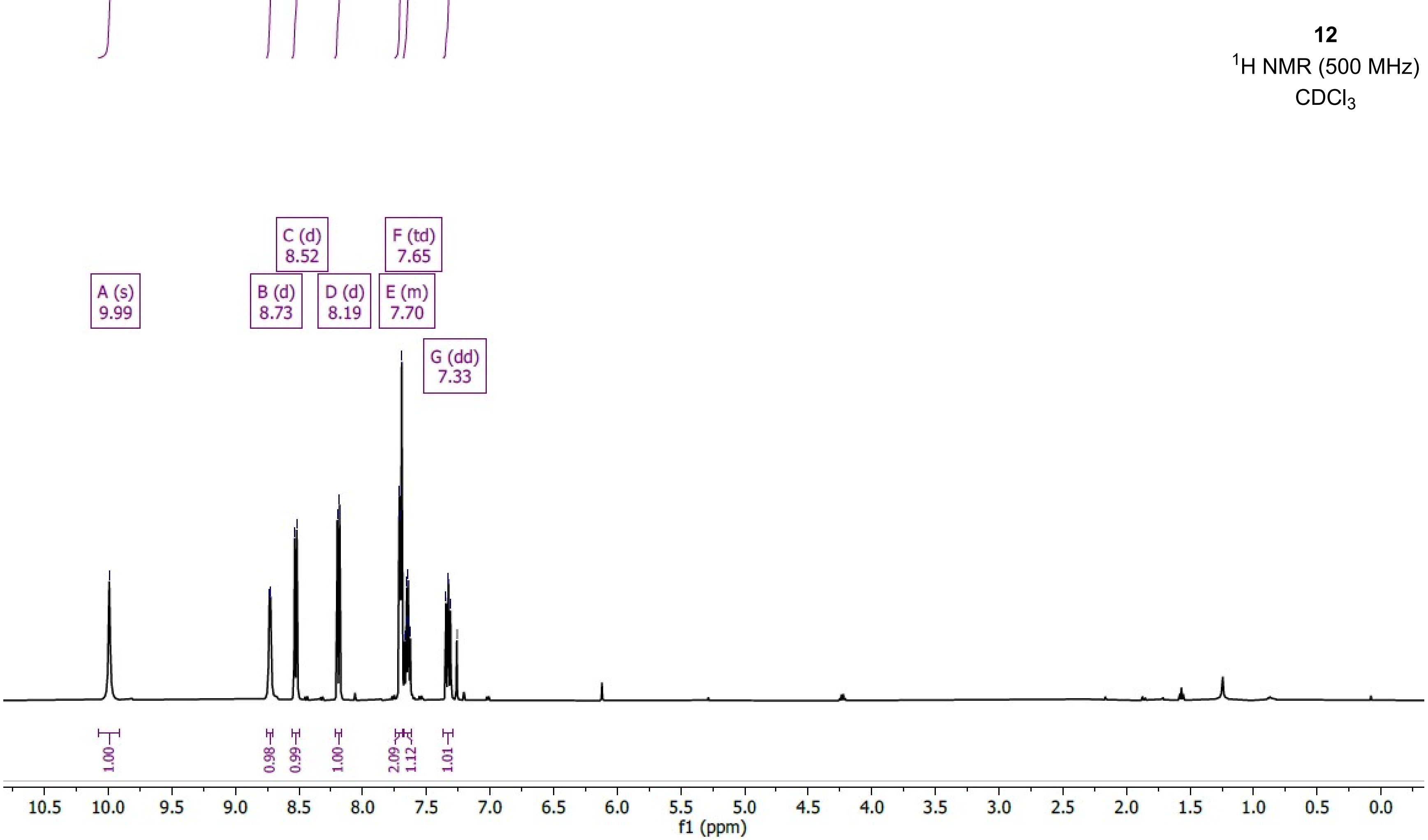

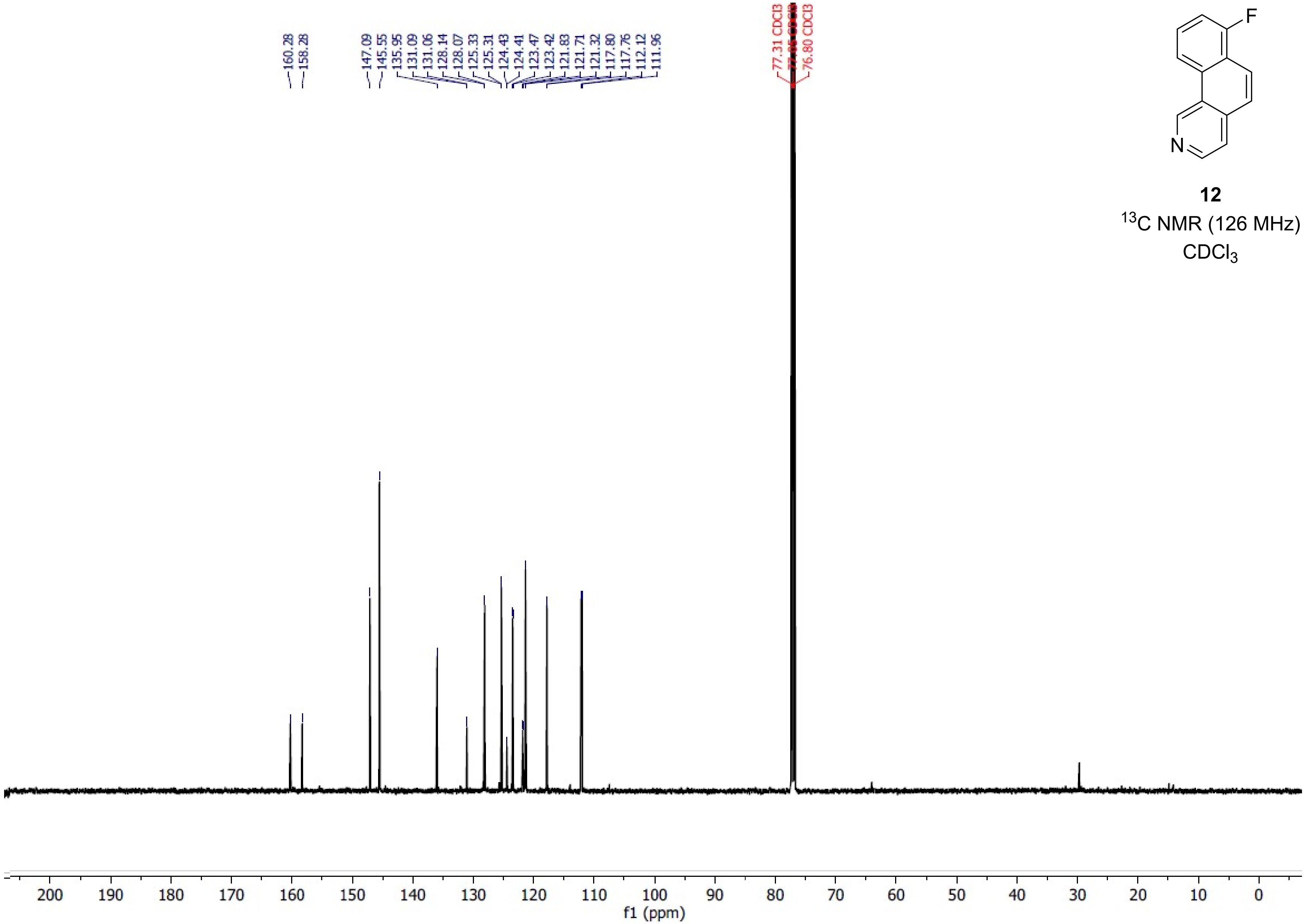

12

^{13}C NMR (126 MHz)

$CDCl_3$

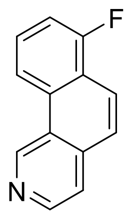

12

^{19}F NMR (470 MHz)

$CDCl_3$

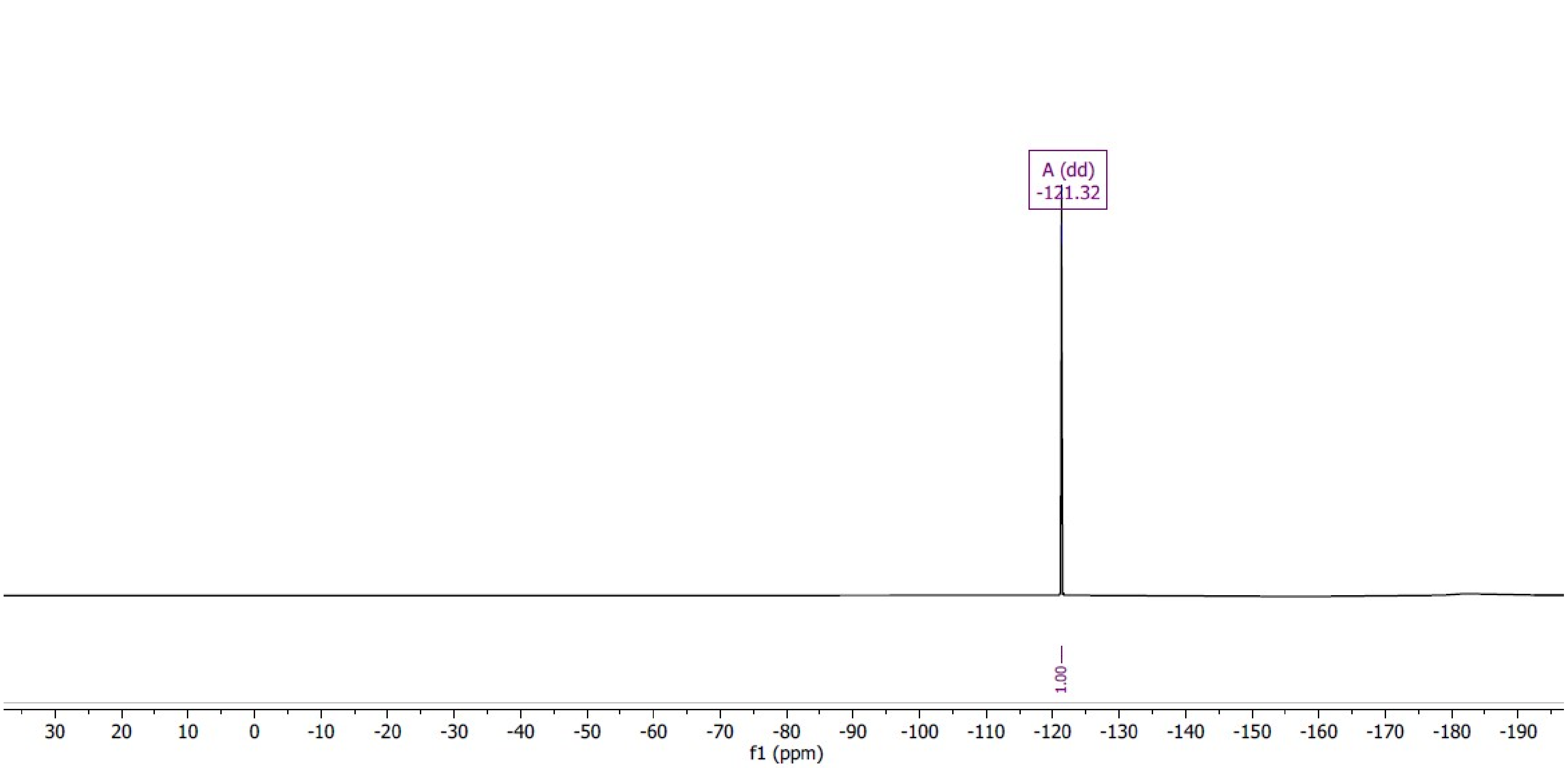

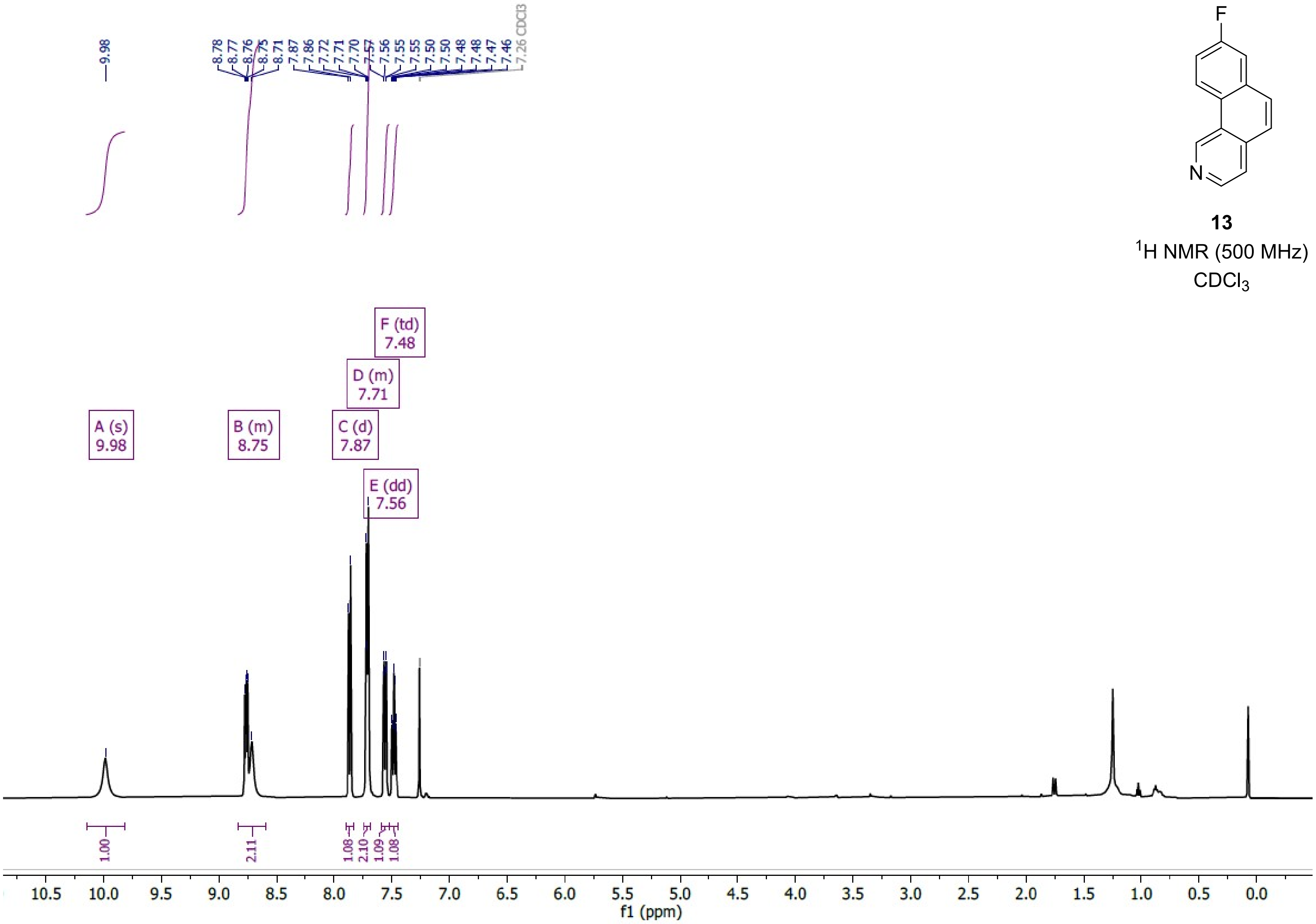

13

^{1}H NMR (500 MHz)

$CDCl_3$

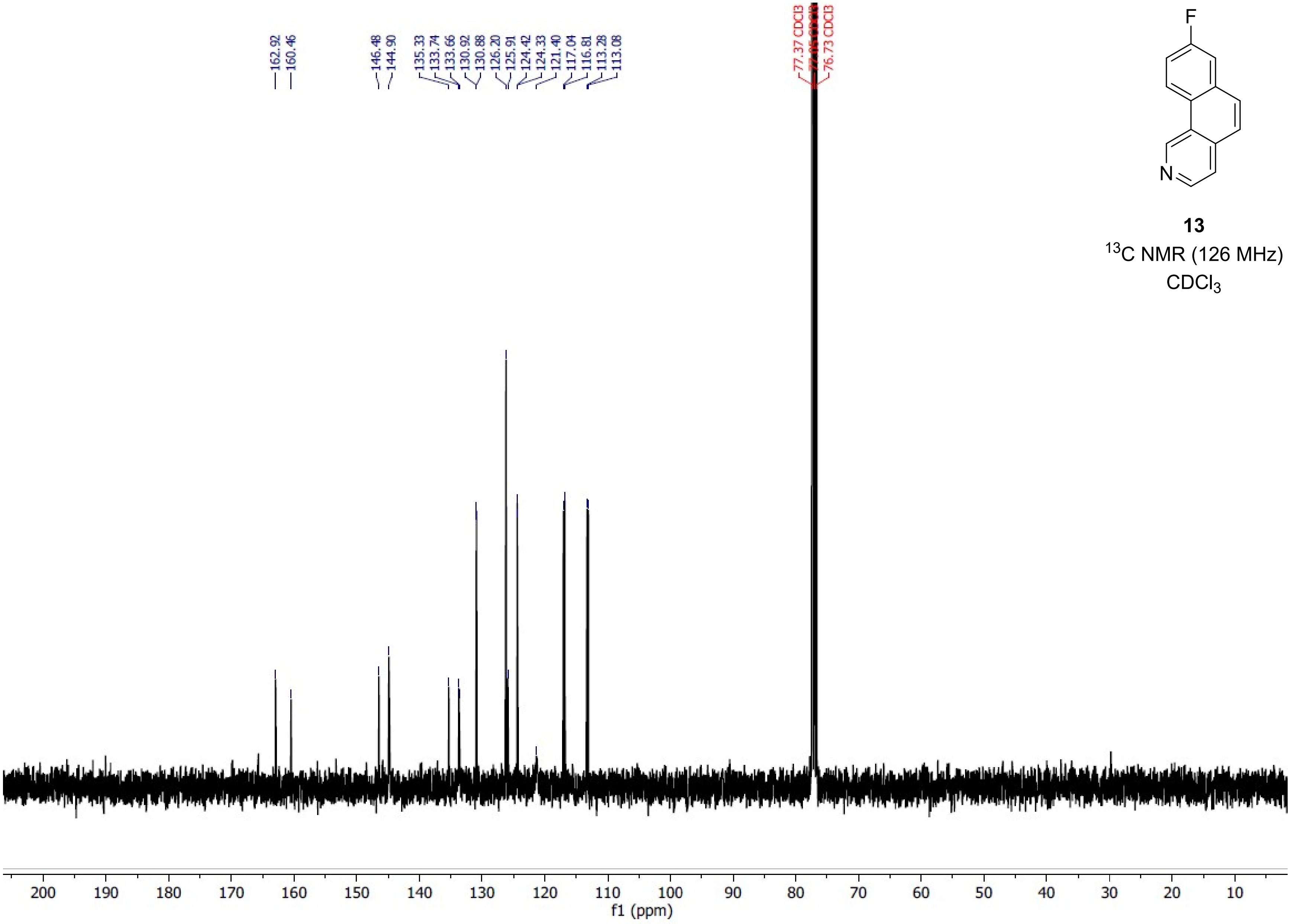

13
^{13}C NMR (126 MHz)
$CDCl_3$

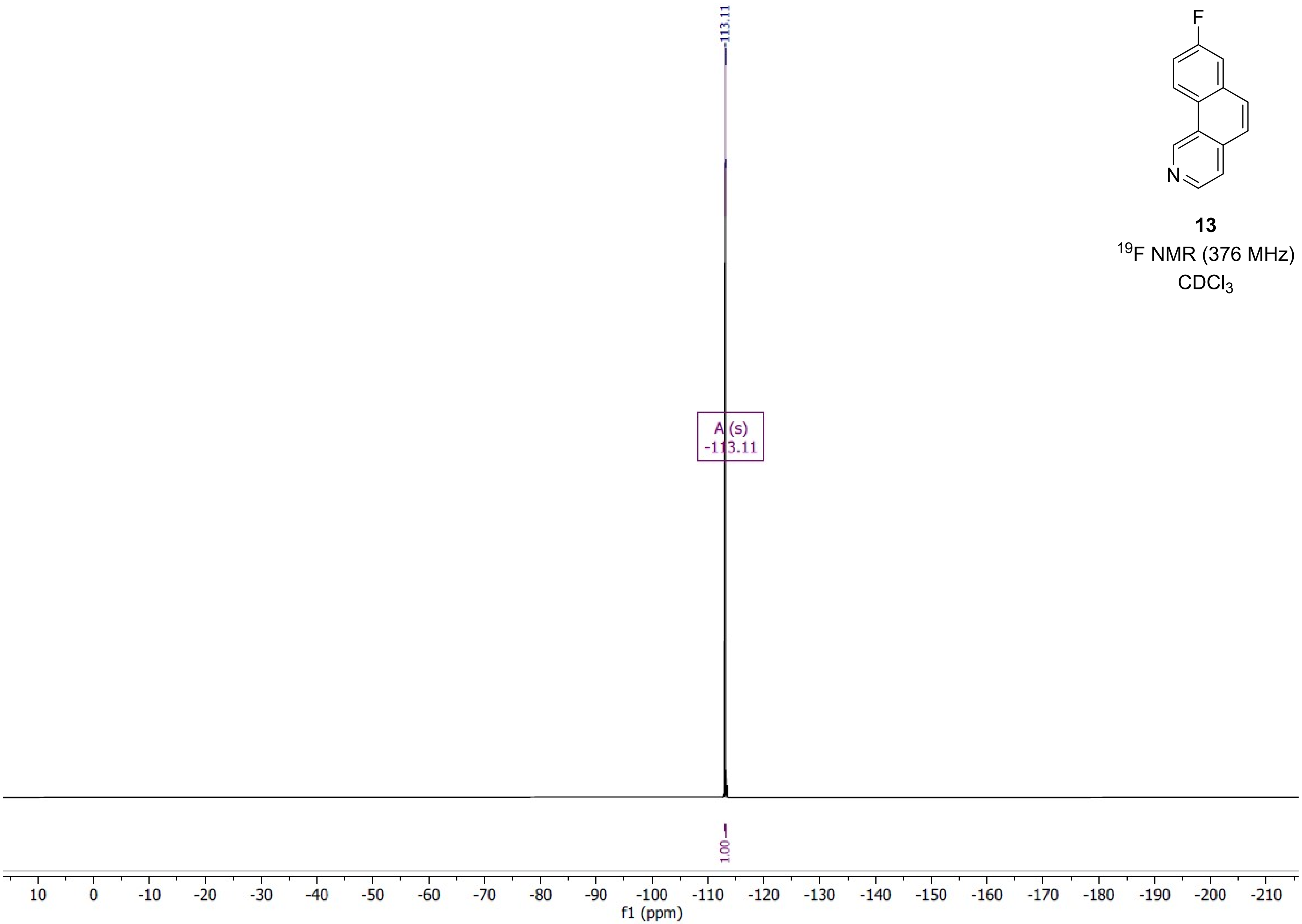

13
^{19}F NMR (376 MHz)
$CDCl_3$

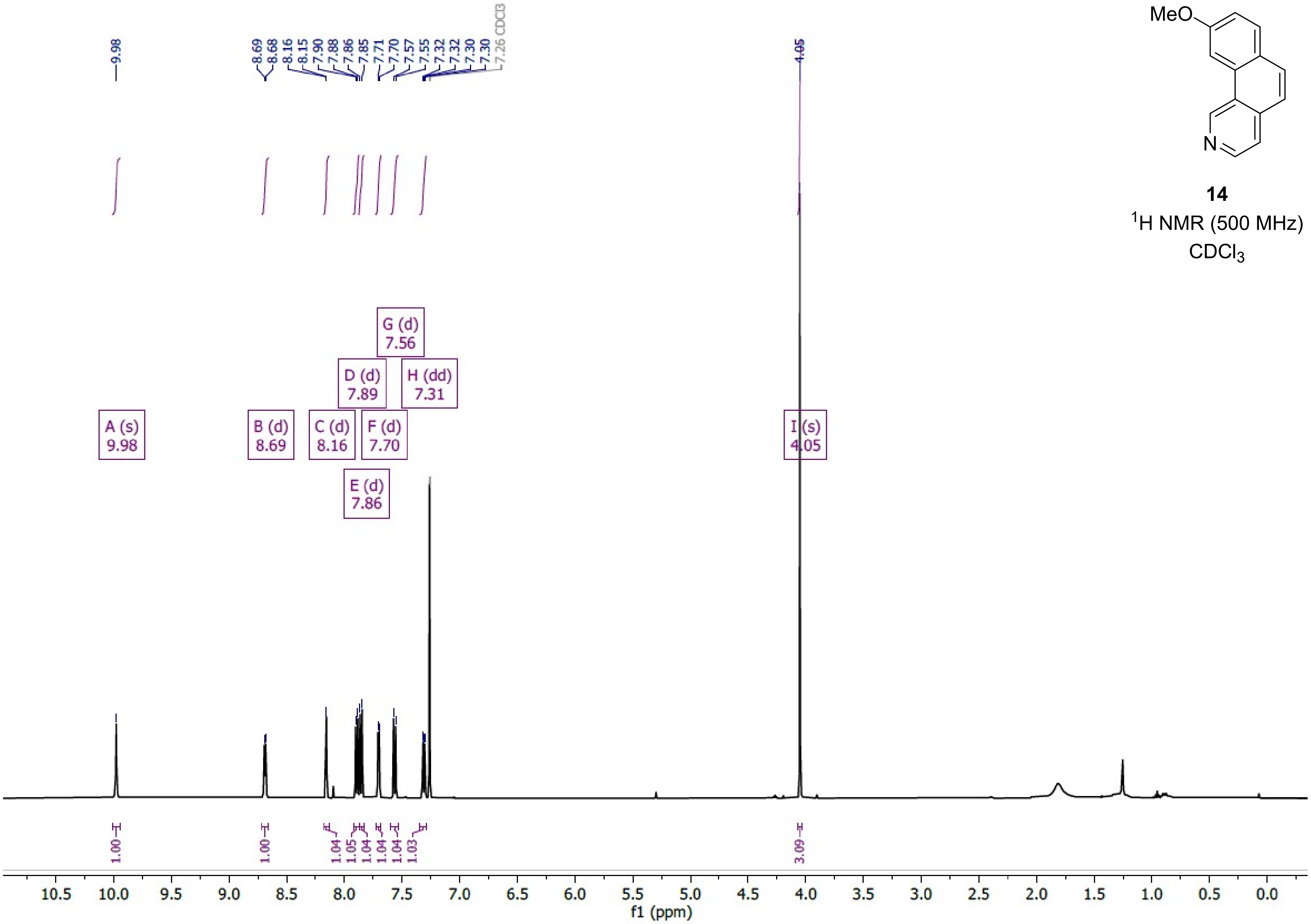

14

^{1}H NMR (500 MHz)

$CDCl_3$

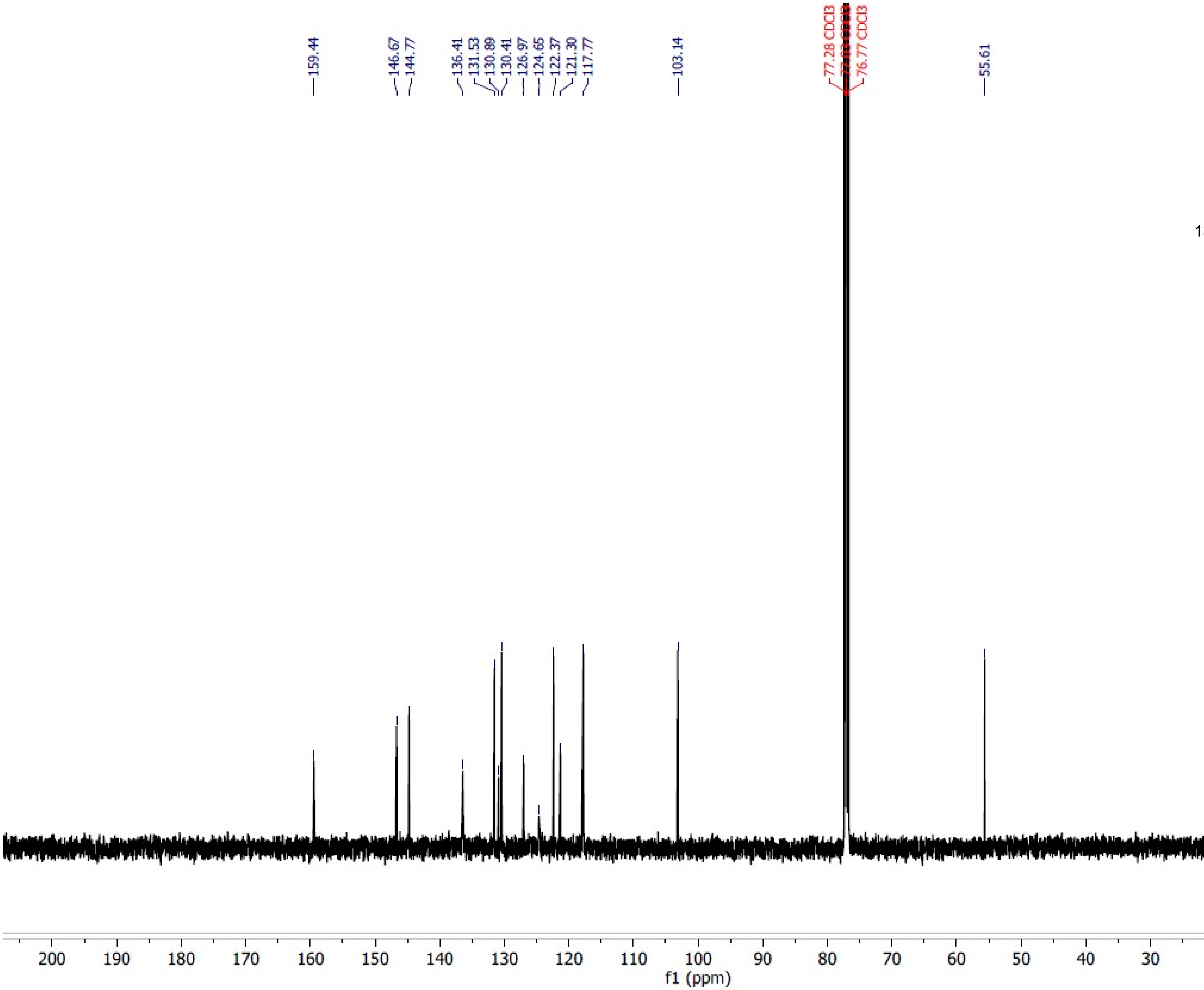

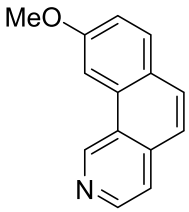

14

^{13}C NMR (126 MHz)

$CDCl_3$

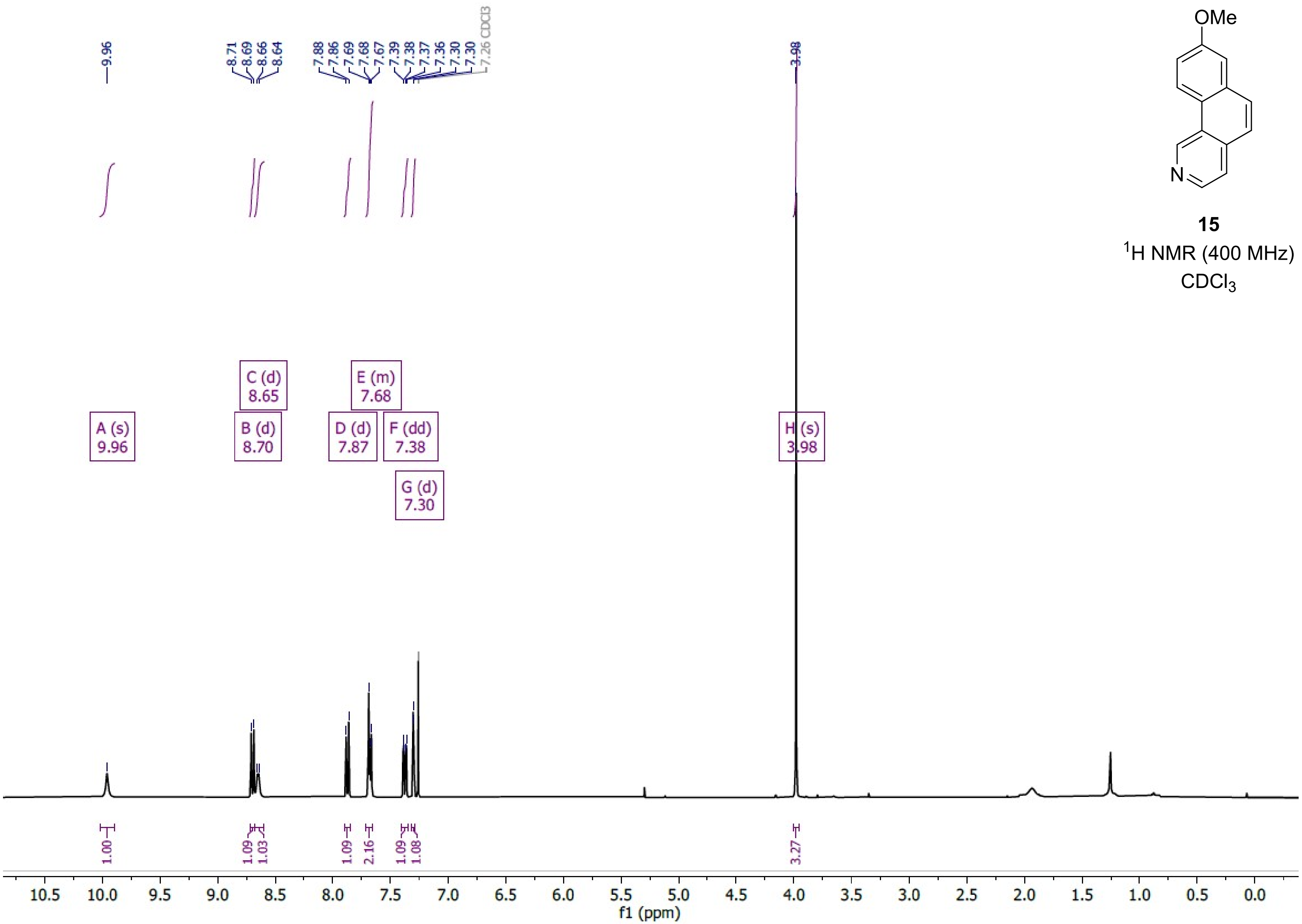

15

^{1}H NMR (400 MHz)

$CDCl_3$

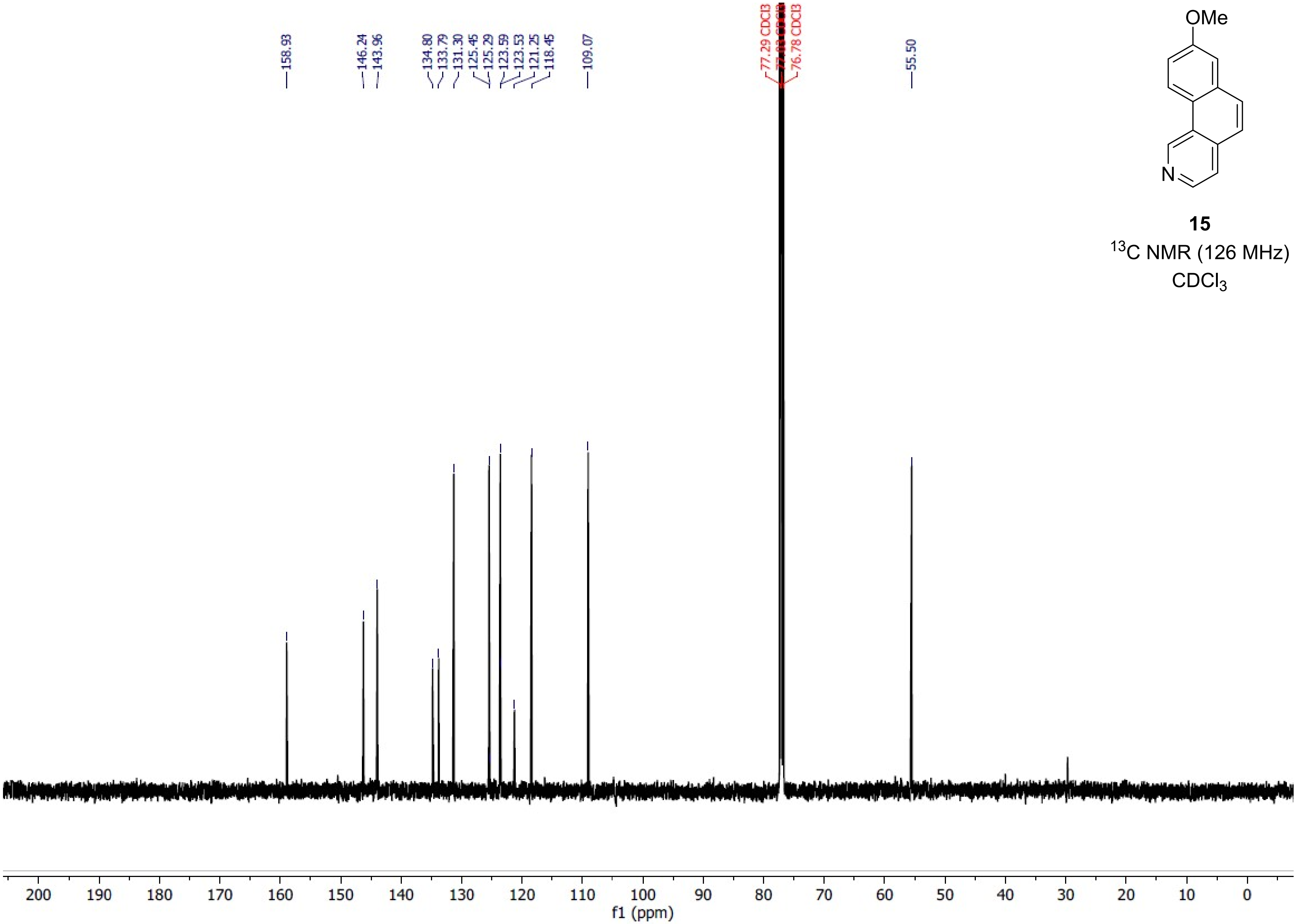

15

^{13}C NMR (126 MHz)

$CDCl_3$

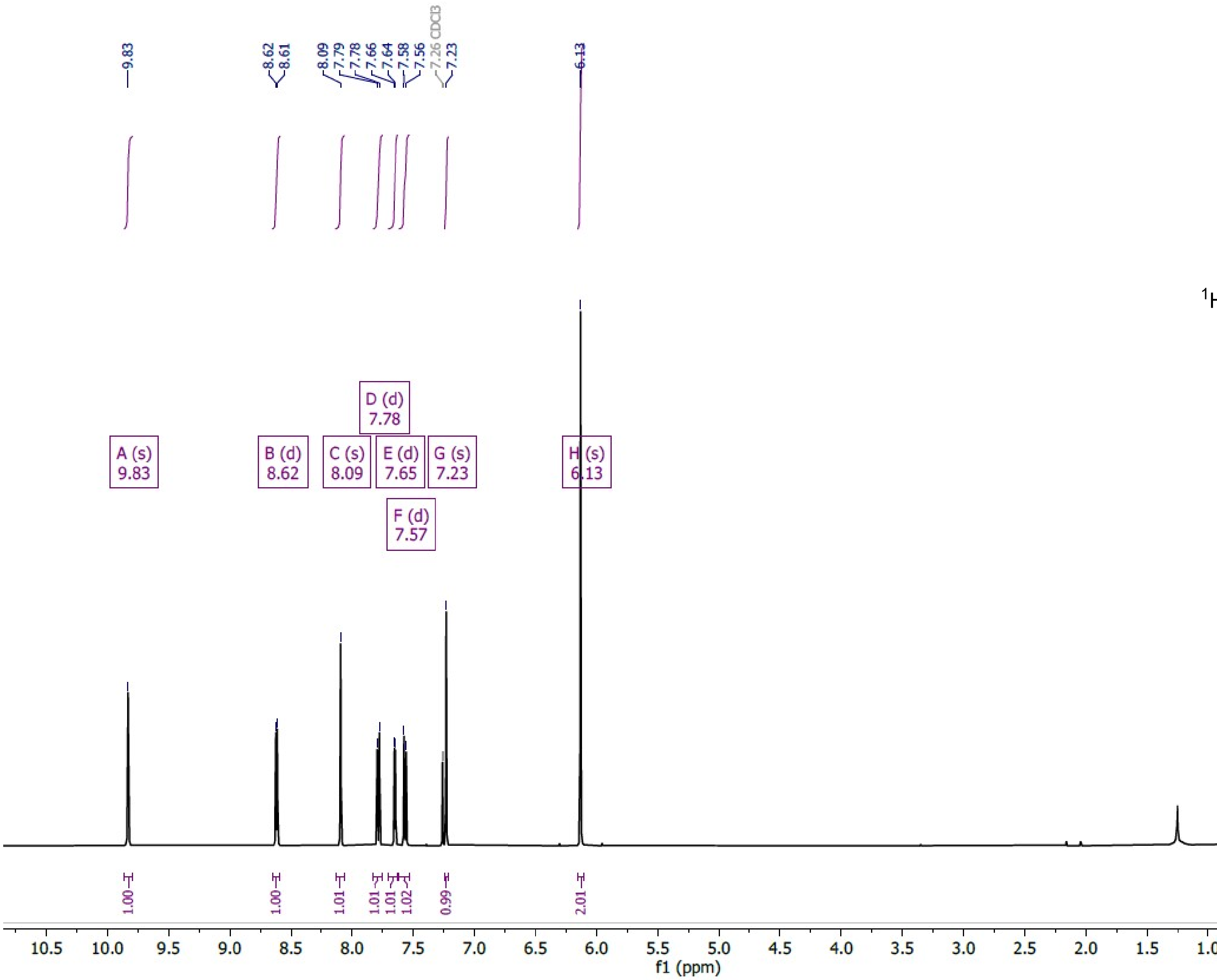

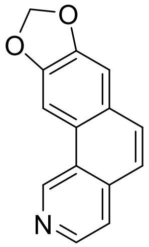

16

^{1}H NMR (500 MHz)

$CDCl_3$

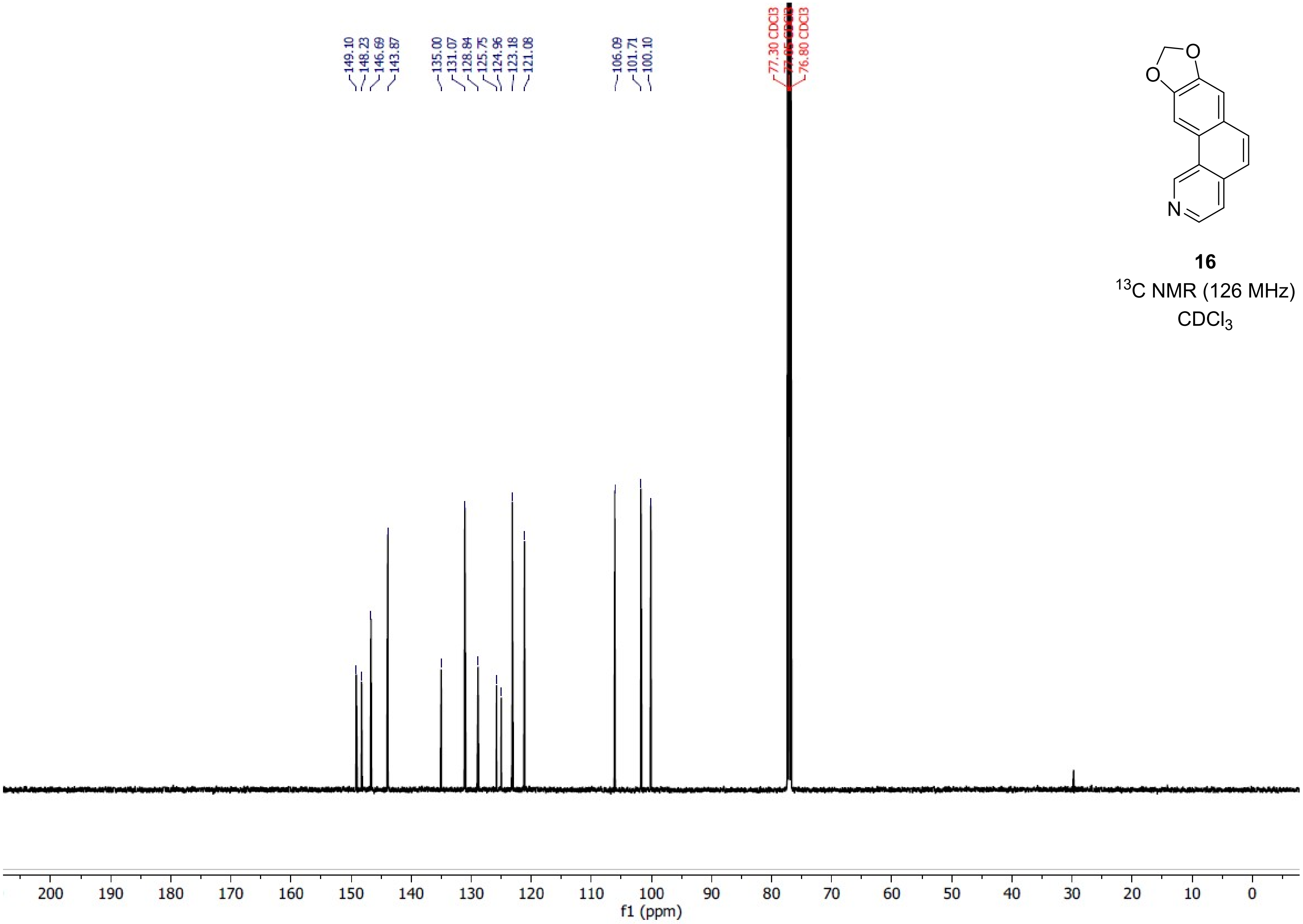

16

^{13}C NMR (126 MHz)

$CDCl_3$

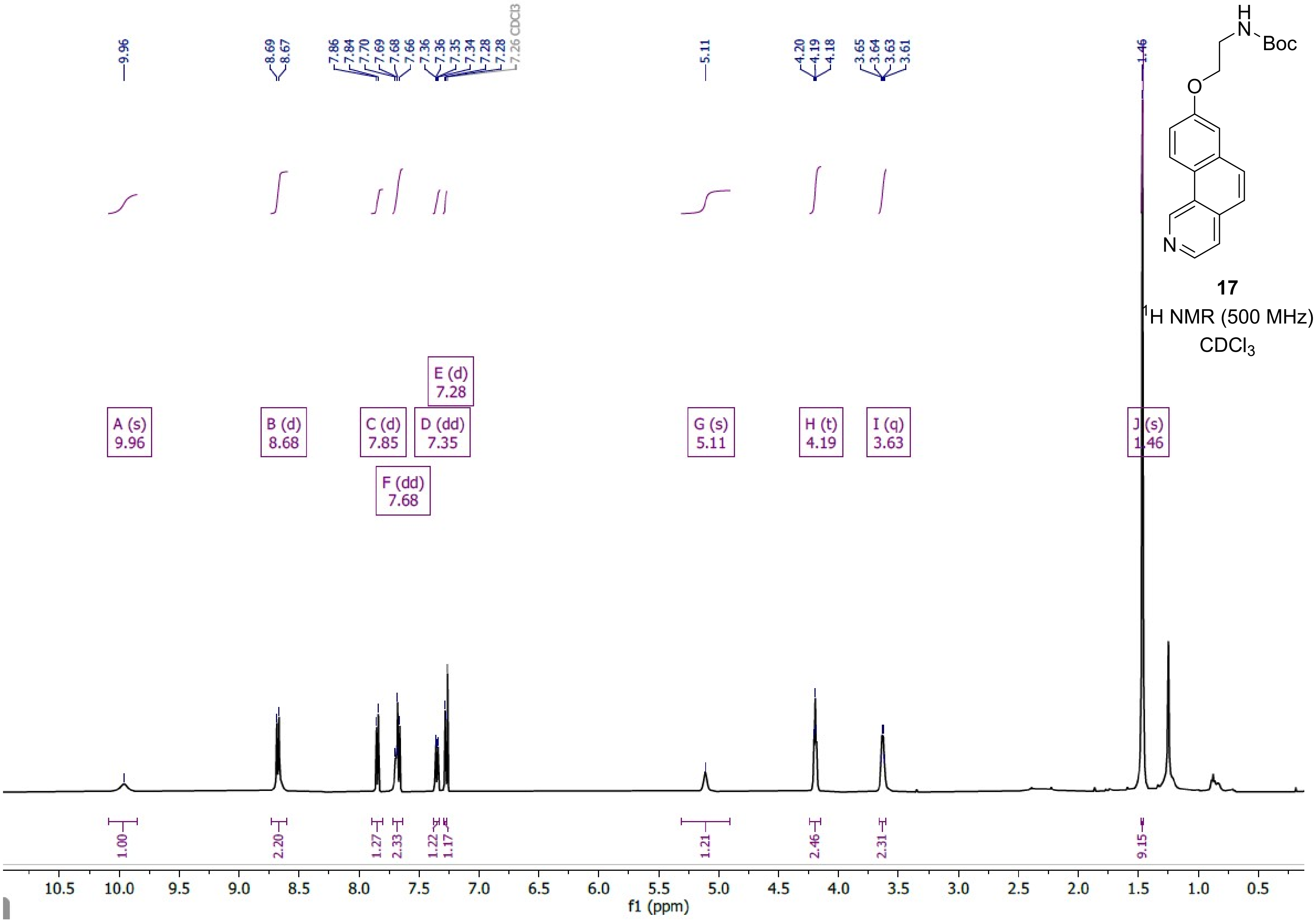

17
^{1}H NMR (500 MHz)
$CDCl_3$
A (s) 9.96
B (d) 8.68
C (d) 7.85
D (dd) 7.35
E (d) 7.28
F (dd) 7.68
G (s) 5.11
H (t) 4.19
I (q) 3.63
J (s) 1.46
f1 (ppm)

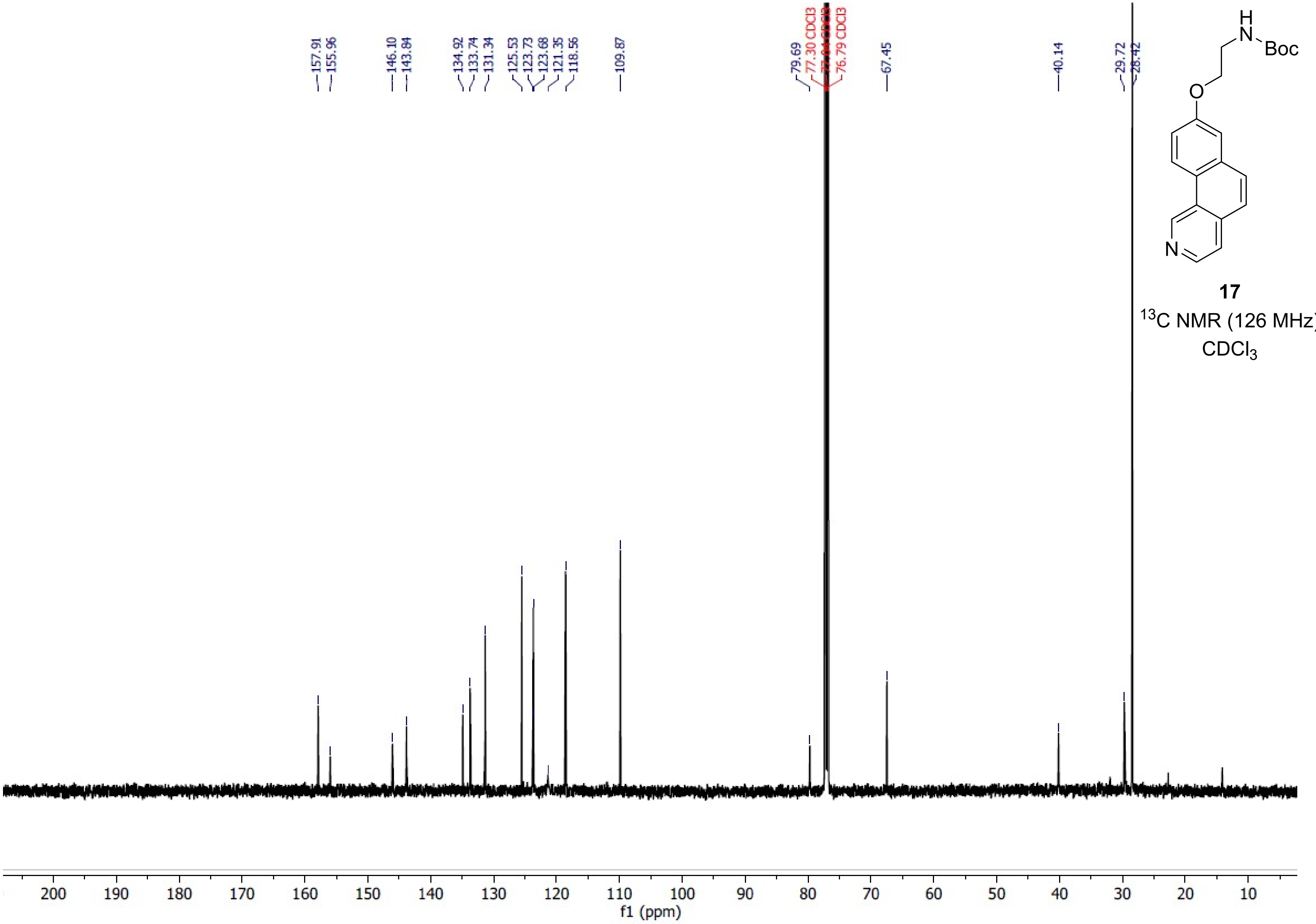

17

^{13}C NMR (126 MHz)

$CDCl_3$

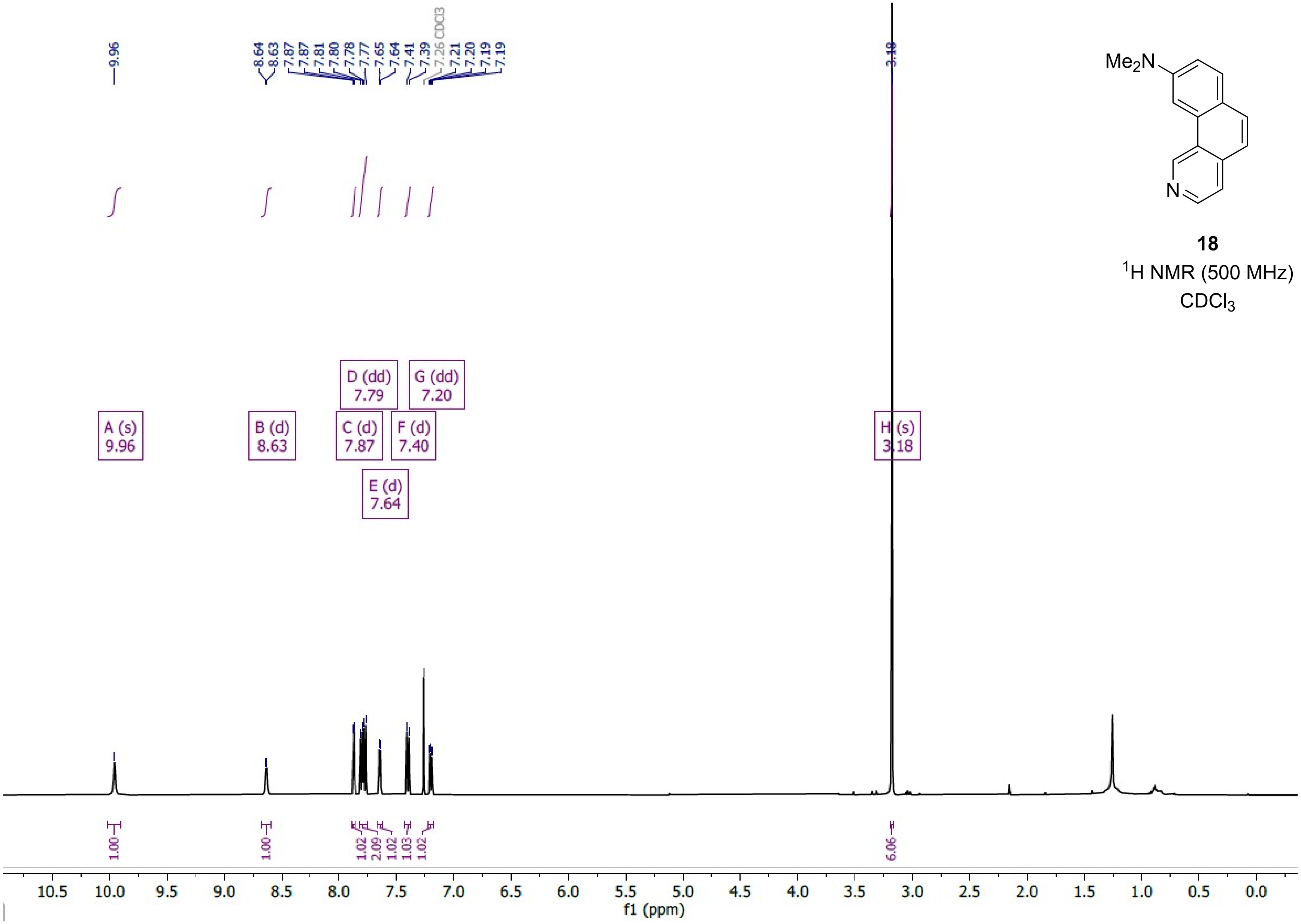

18

^{1}H NMR (500 MHz)

$CDCl_3$

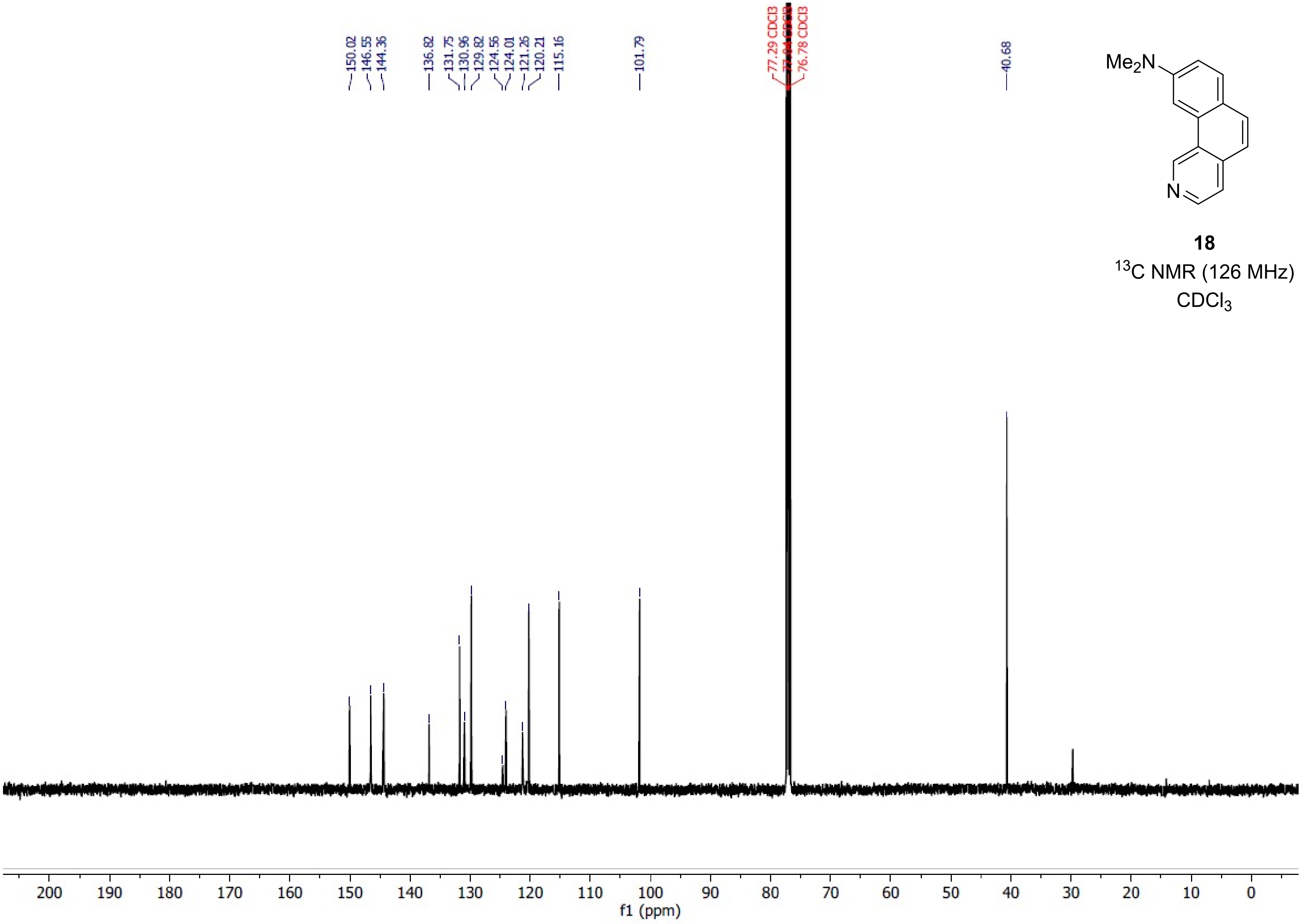

18

^{13}C NMR (126 MHz)

$CDCl_3$

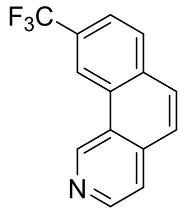

19
^1H NMR (500 MHz)
$CDCl_3$

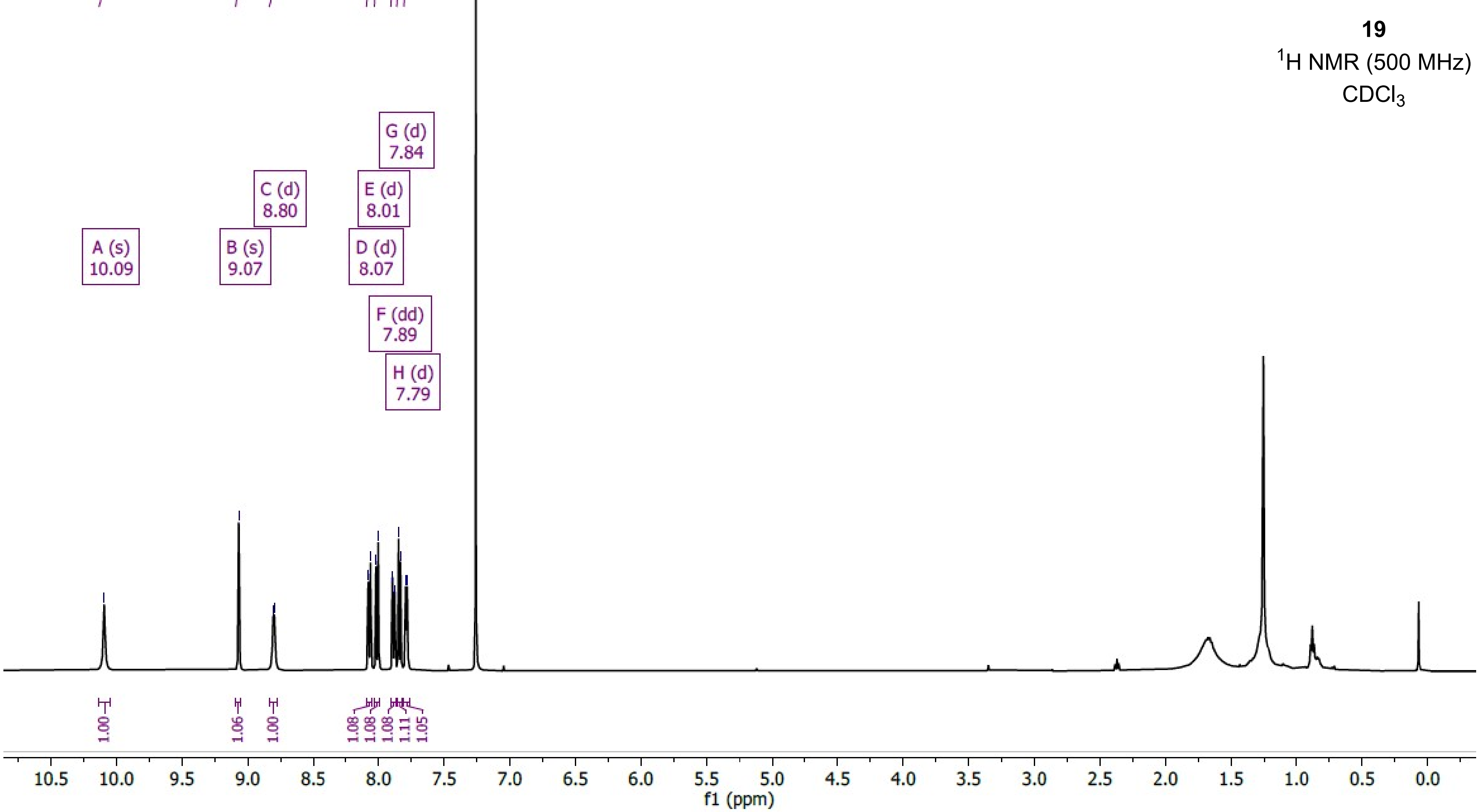

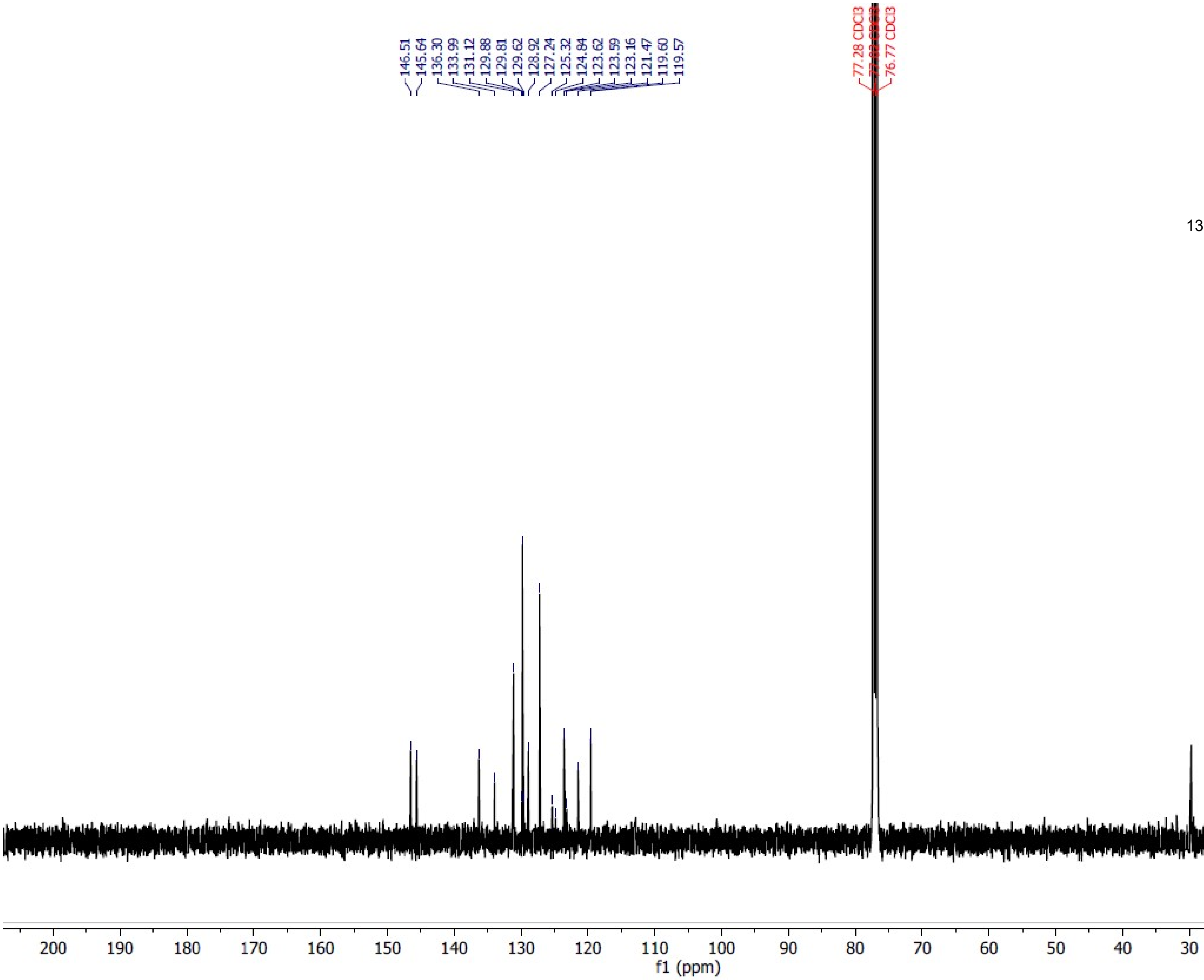

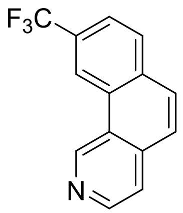

19

^{13}C NMR (126 MHz)

$CDCl_3$

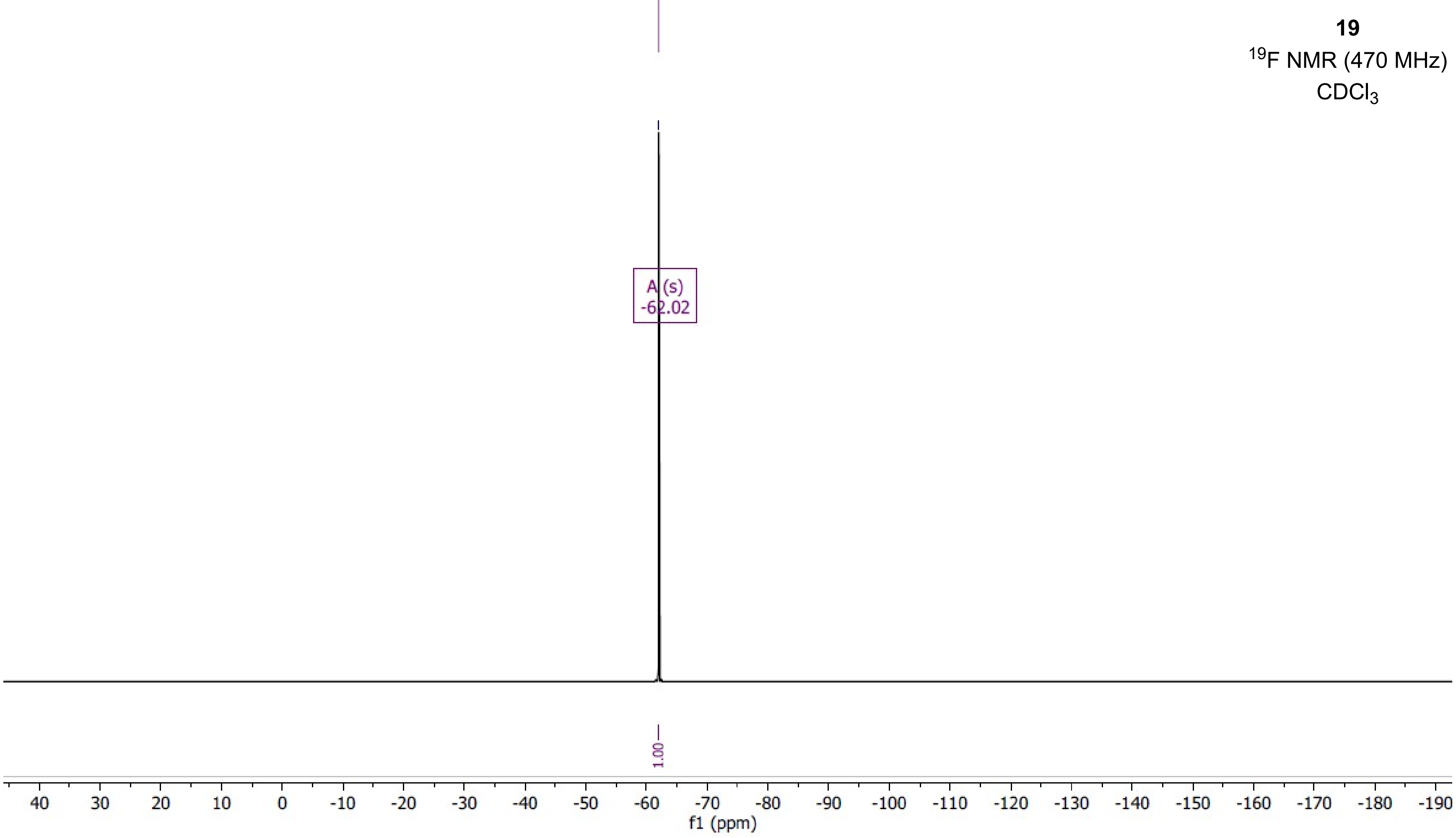

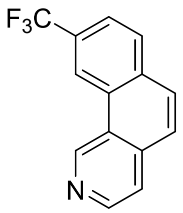

19

^{19}F NMR (470 MHz)

$CDCl_3$

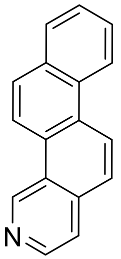

20
^1H NMR (500 MHz)
$CDCl_3$

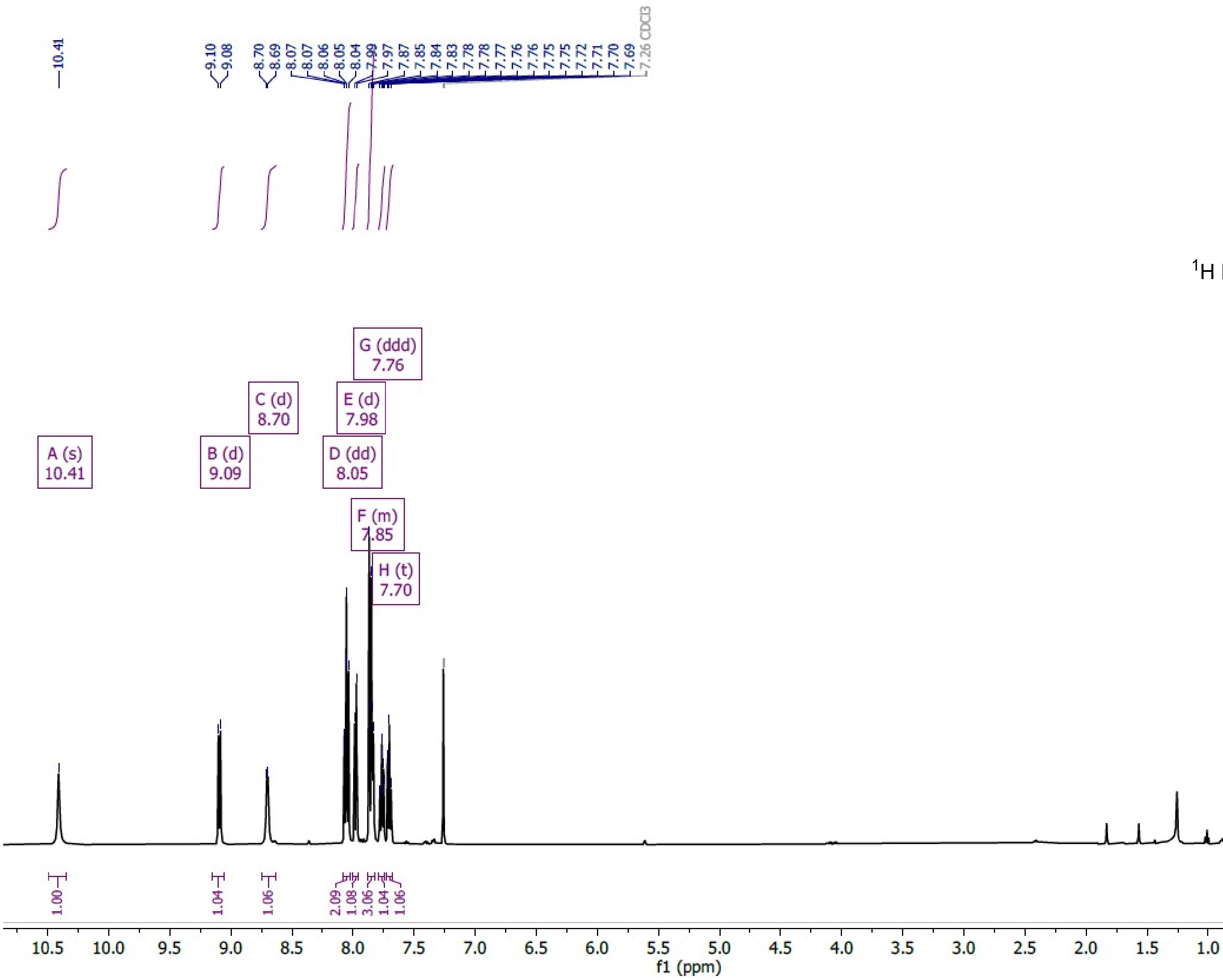

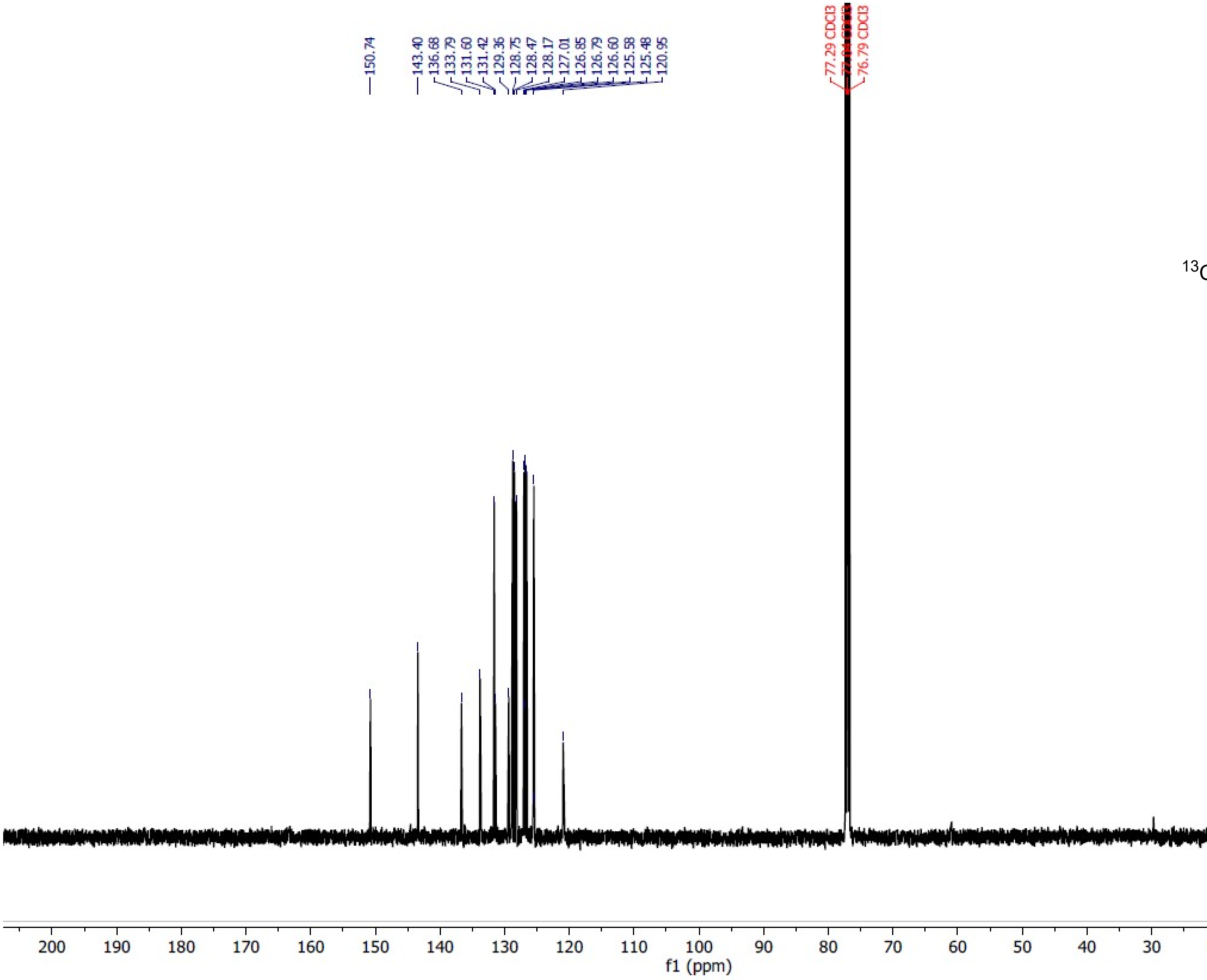

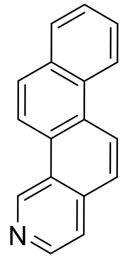

20

^{13}C NMR (126 MHz)

$CDCl_3$

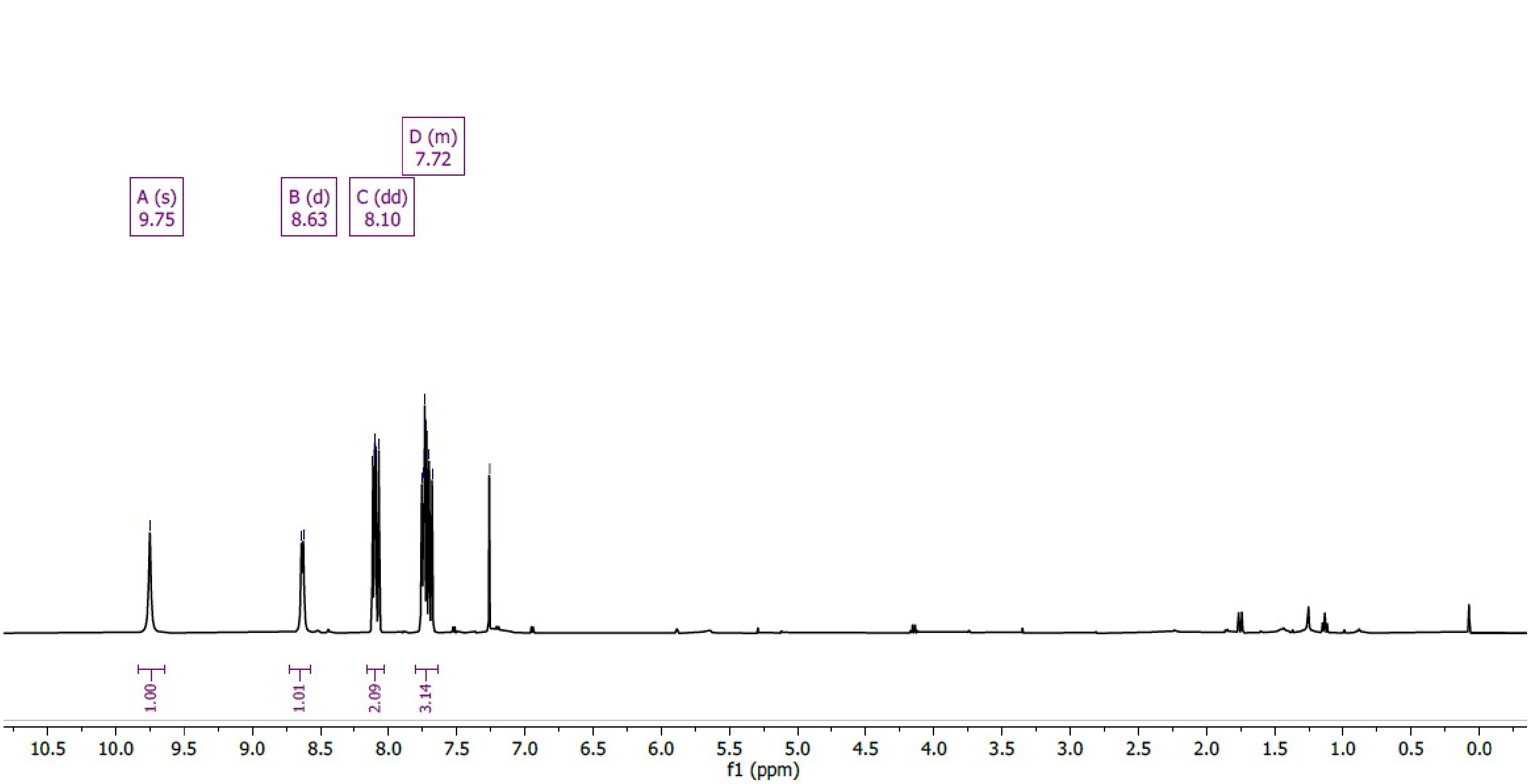

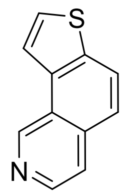

21

^{1}H NMR (400 MHz)

$CDCl_3$

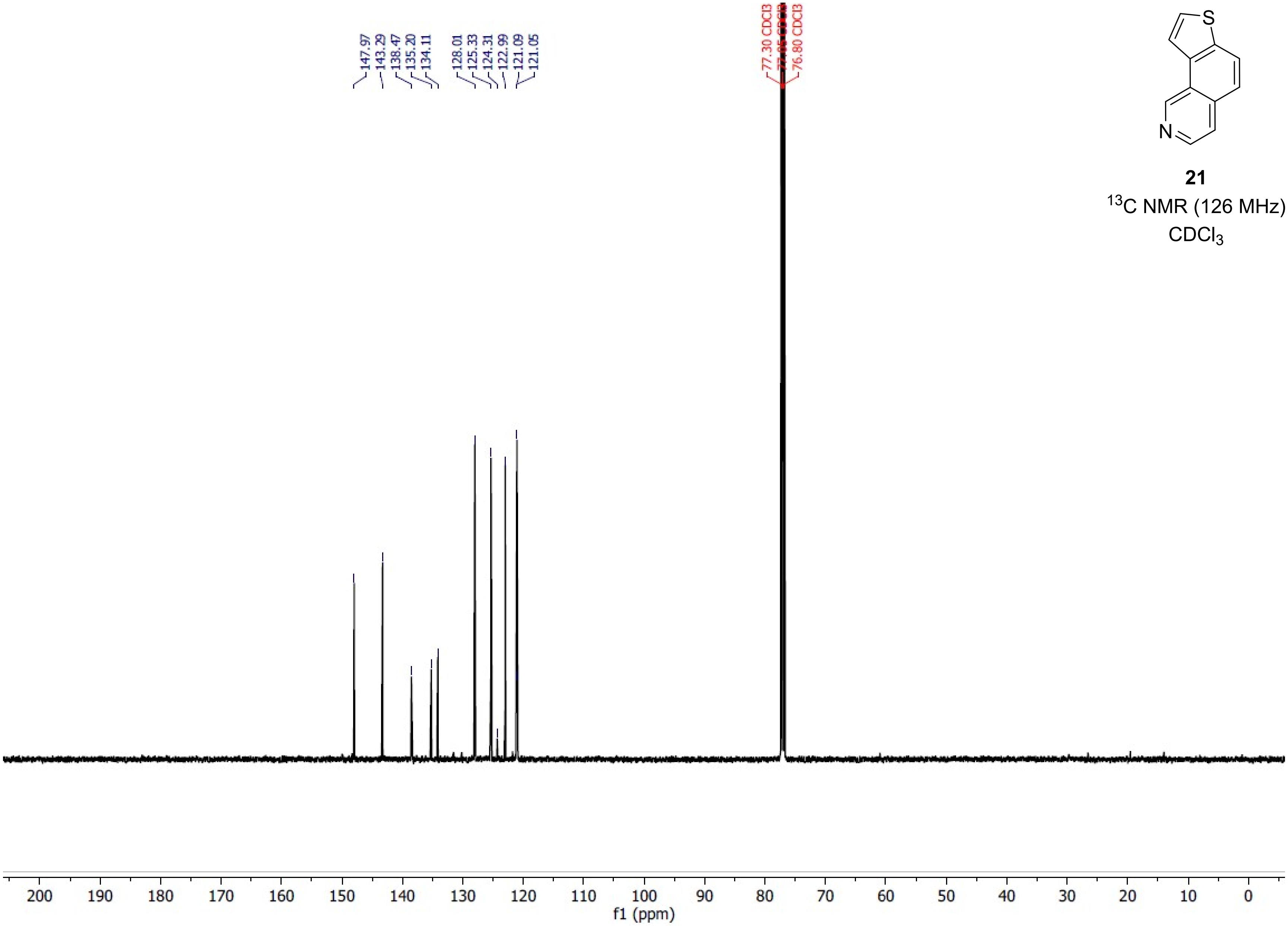

21

^{13}C NMR (126 MHz)

$CDCl_3$

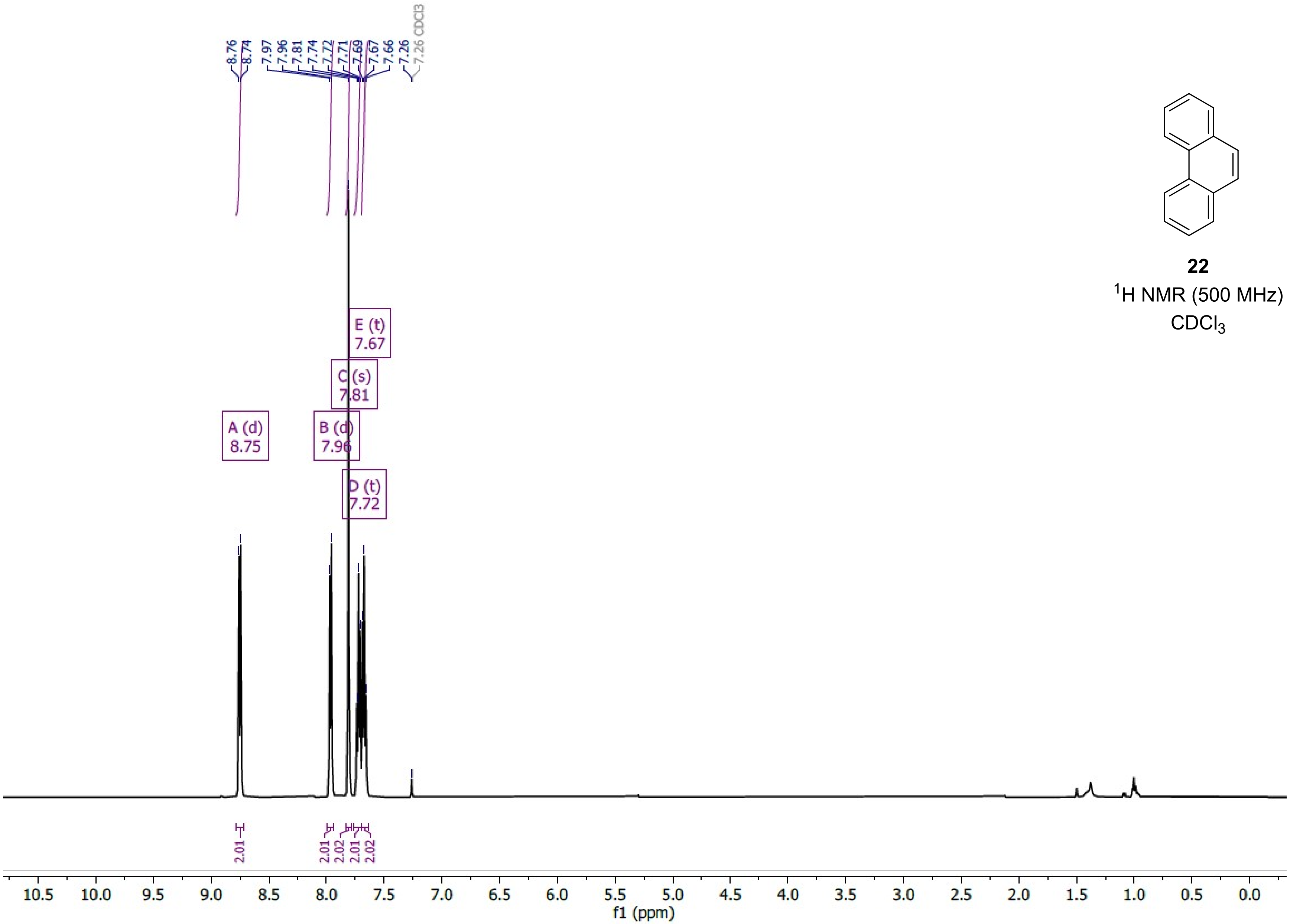

22

^{1}H NMR (500 MHz)

$CDCl_3$

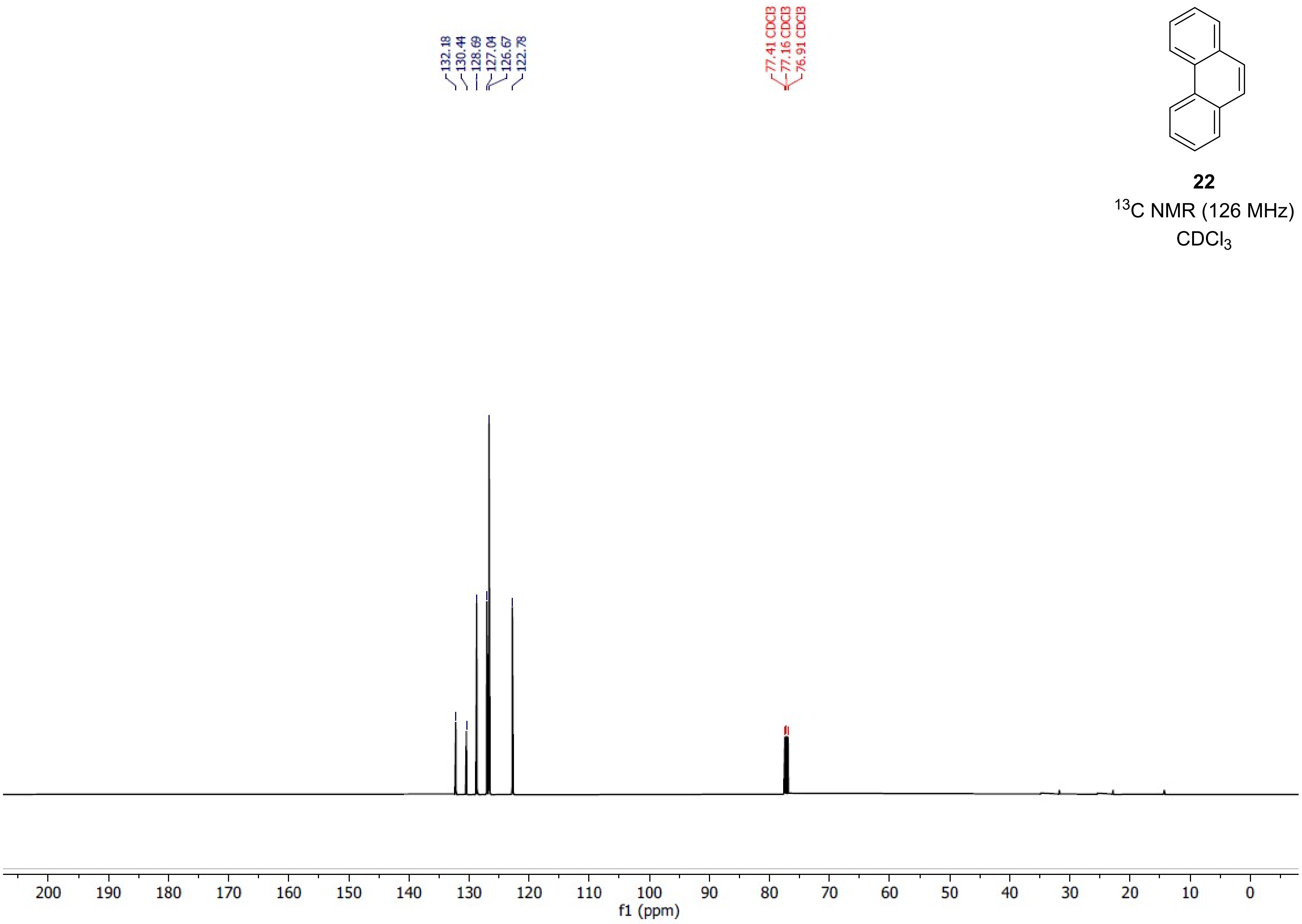

22
^{13}C NMR (126 MHz)
$CDCl_3$

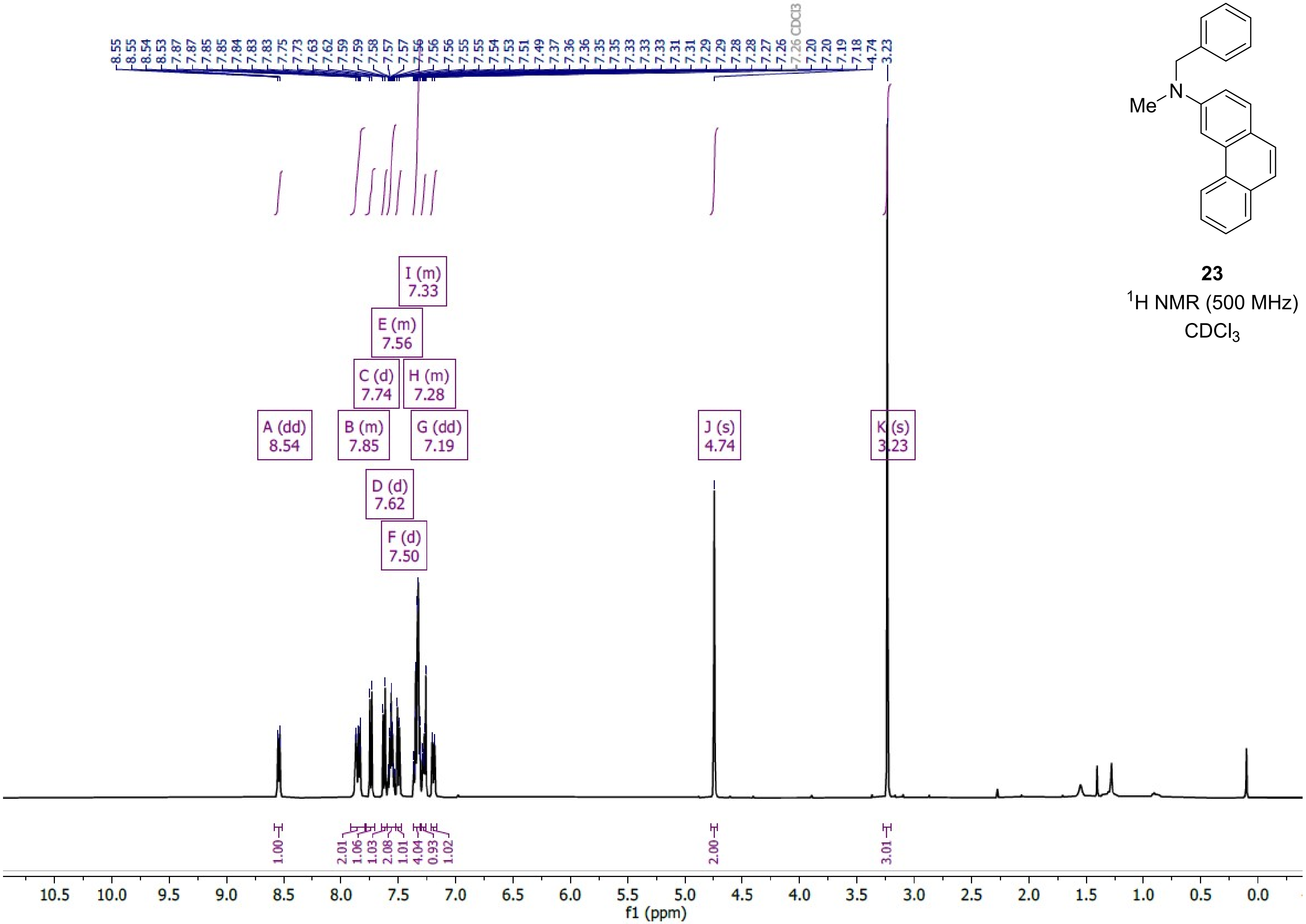

23

^{1}H NMR (500 MHz)

$CDCl_3$

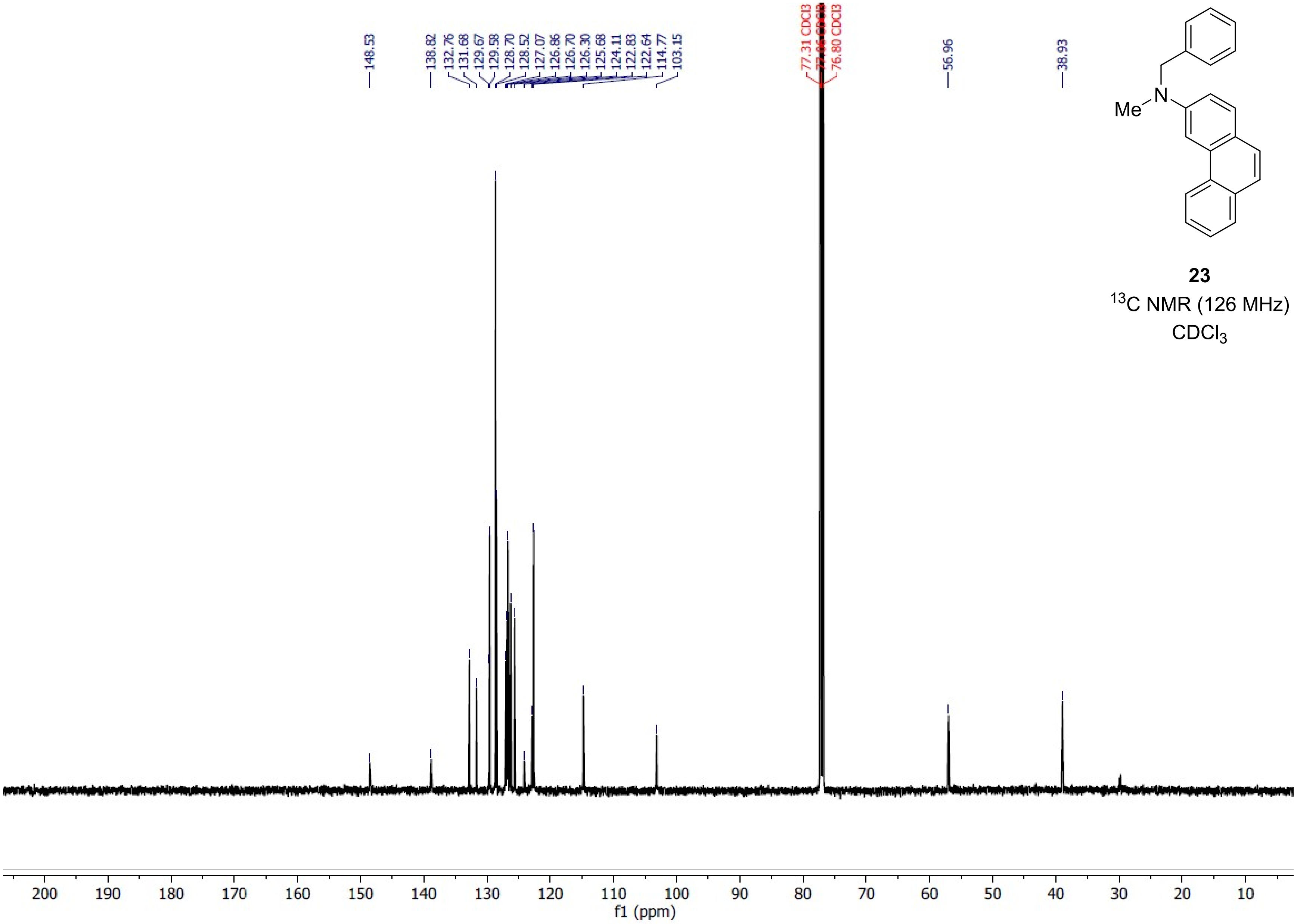

23

^{13}C NMR (126 MHz)

$CDCl_3$

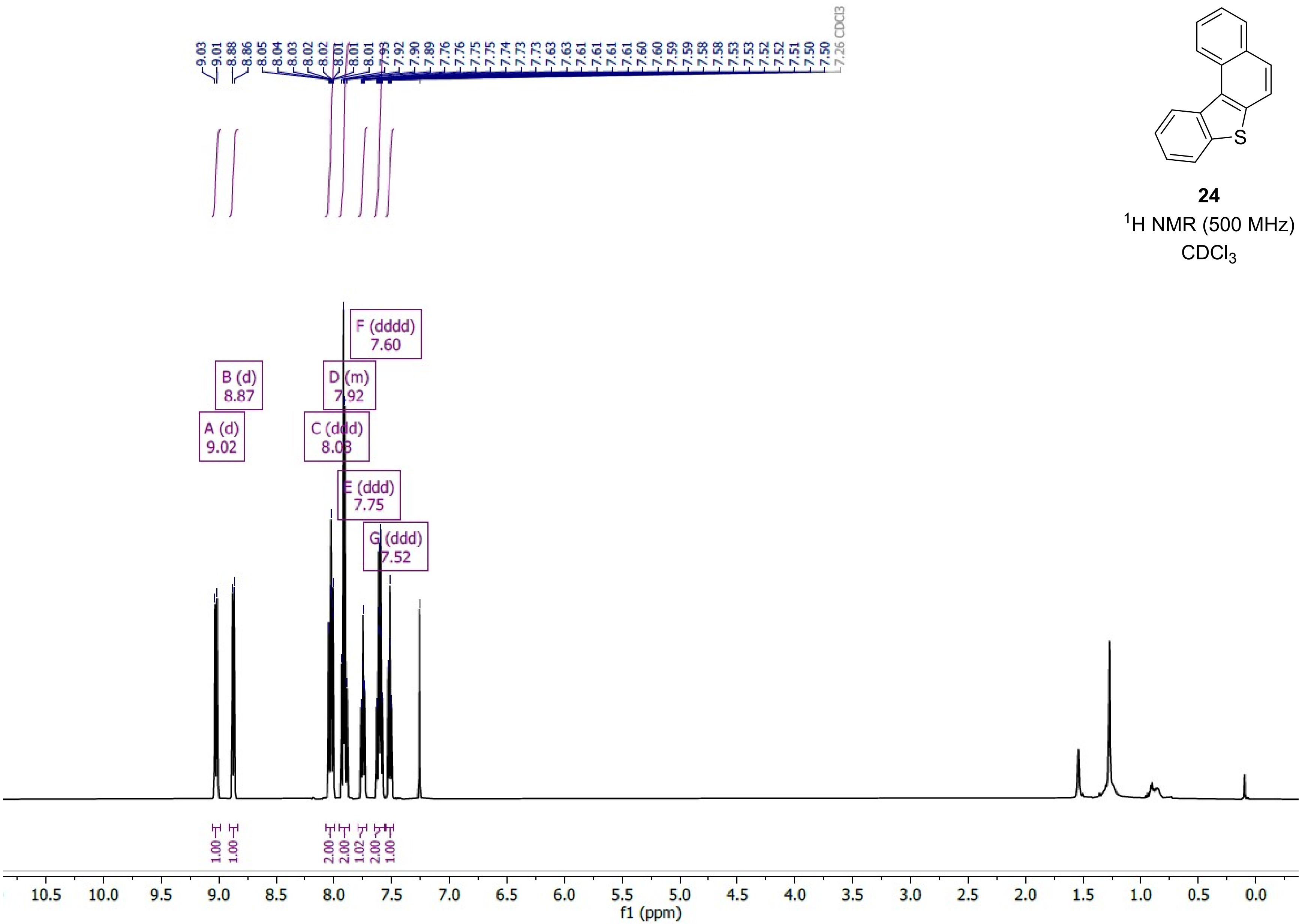

24

^{1}H NMR (500 MHz)

$CDCl_3$

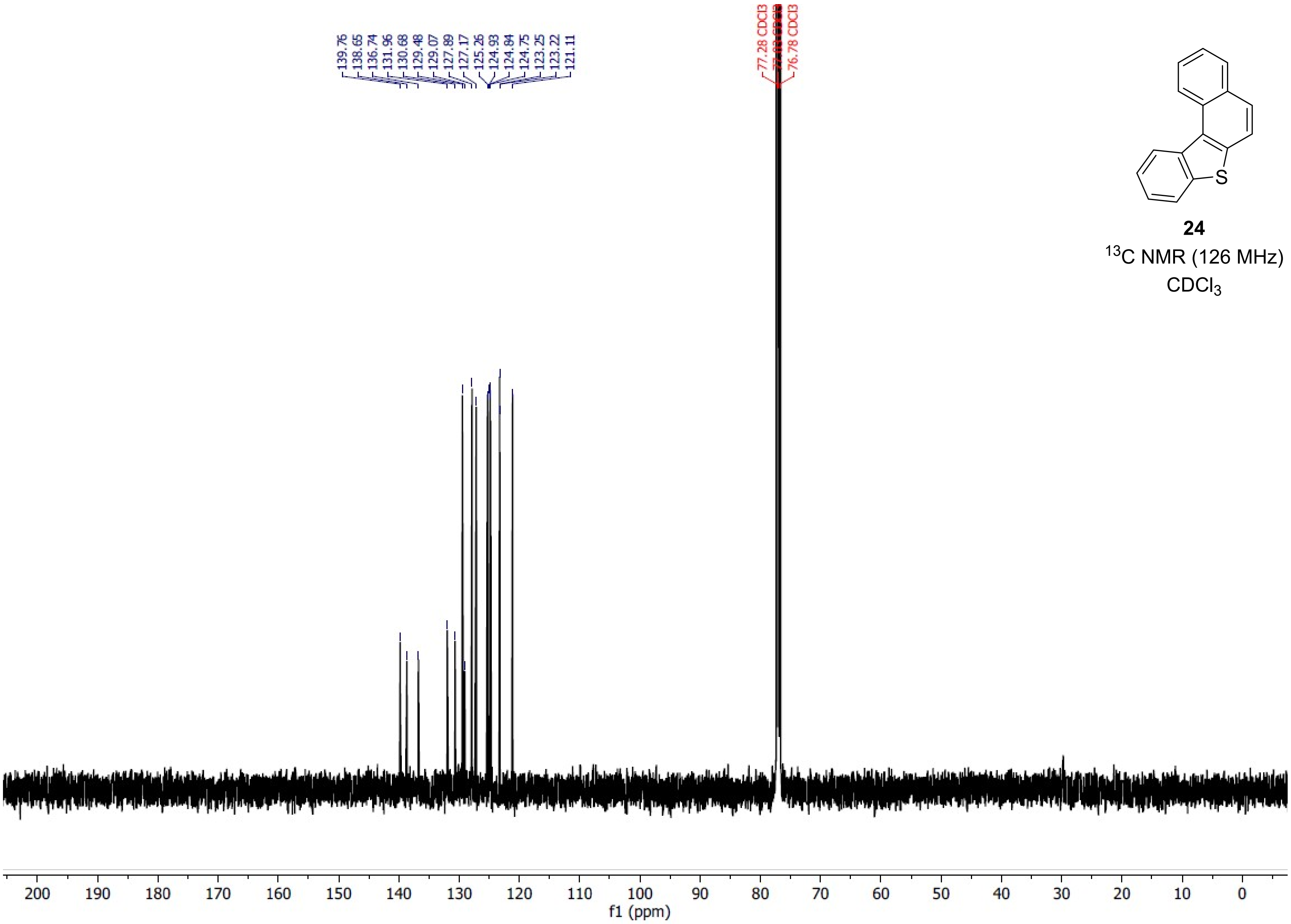

24

^{13}C NMR (126 MHz)

$CDCl_3$

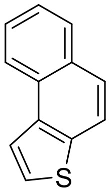

25

^{1}H NMR (500 MHz)

$CDCl_3$

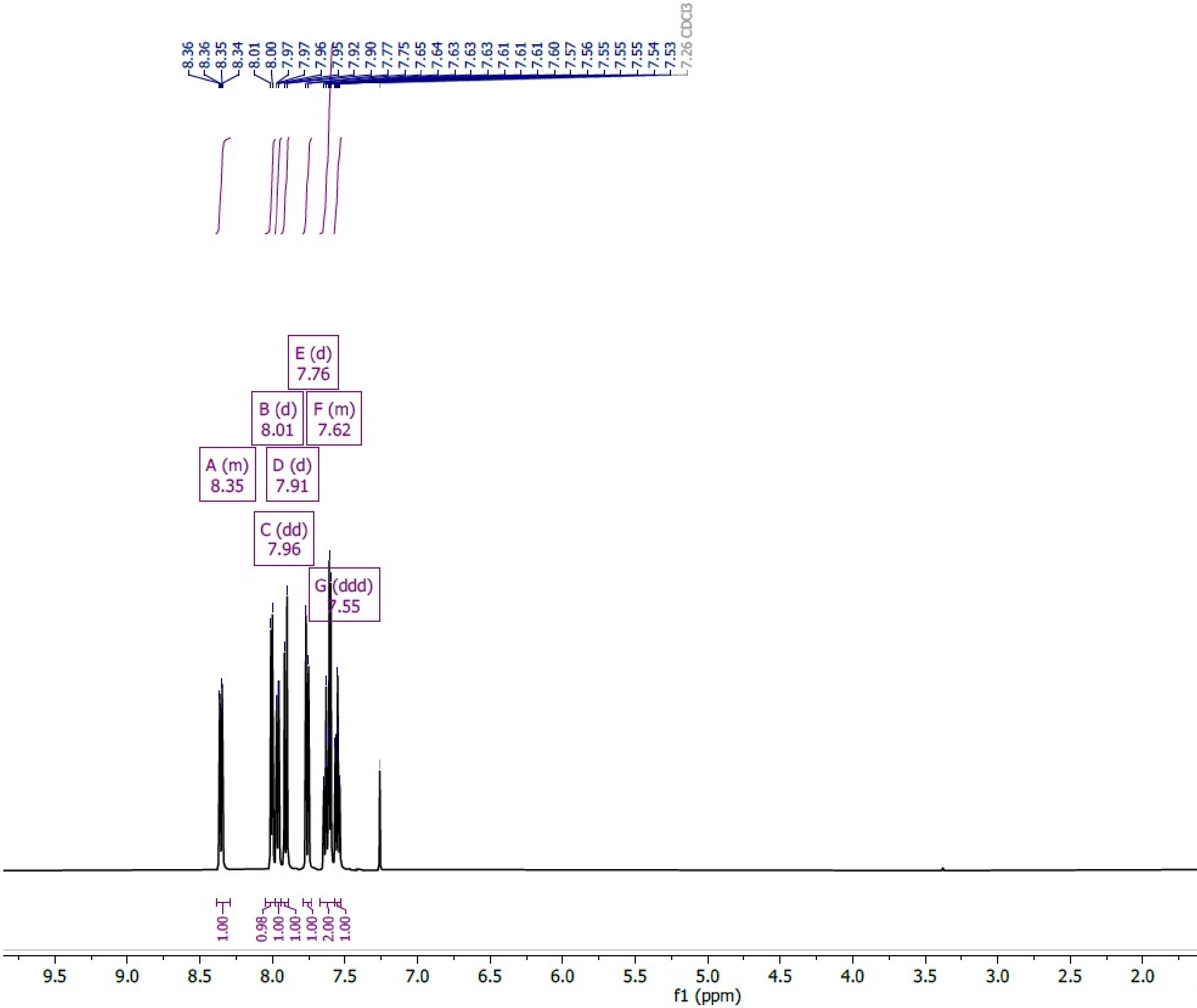

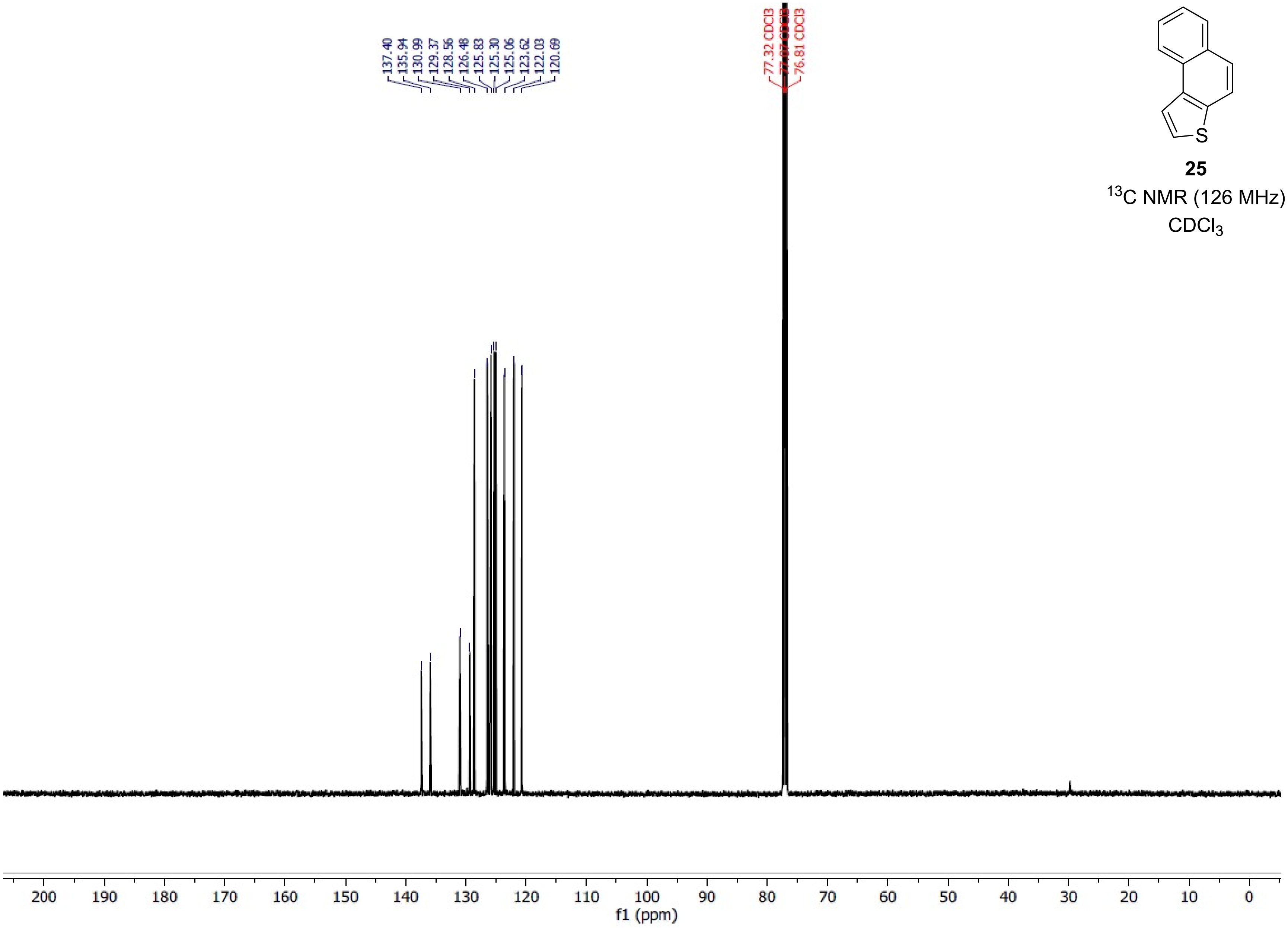
137.40
135.94
130.99
129.37
128.56
126.48
125.83
125.30
125.06
123.62
122.03
120.69
77.32 CDCl3
77.07 CDCl3
76.81 CDCl3
S
25
13C NMR (126 MHz)
CDCl3
200
190
180
170
160
150
140
130
120
110
100
90
80
70
60
50
40
30
20
10
0
f1 (ppm)

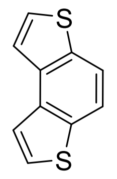

26

^{1}H NMR (500 MHz)

$CDCl_3$

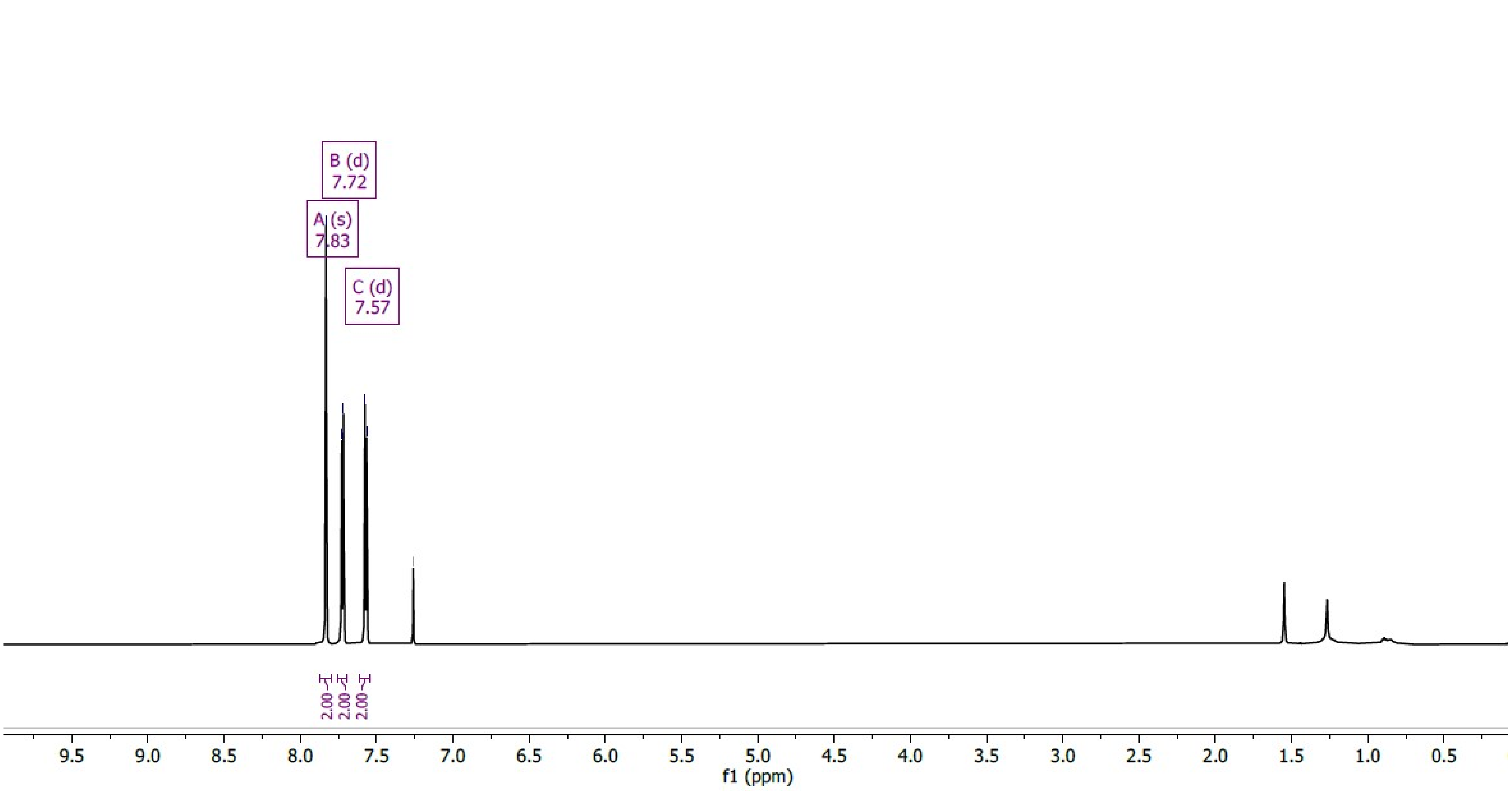

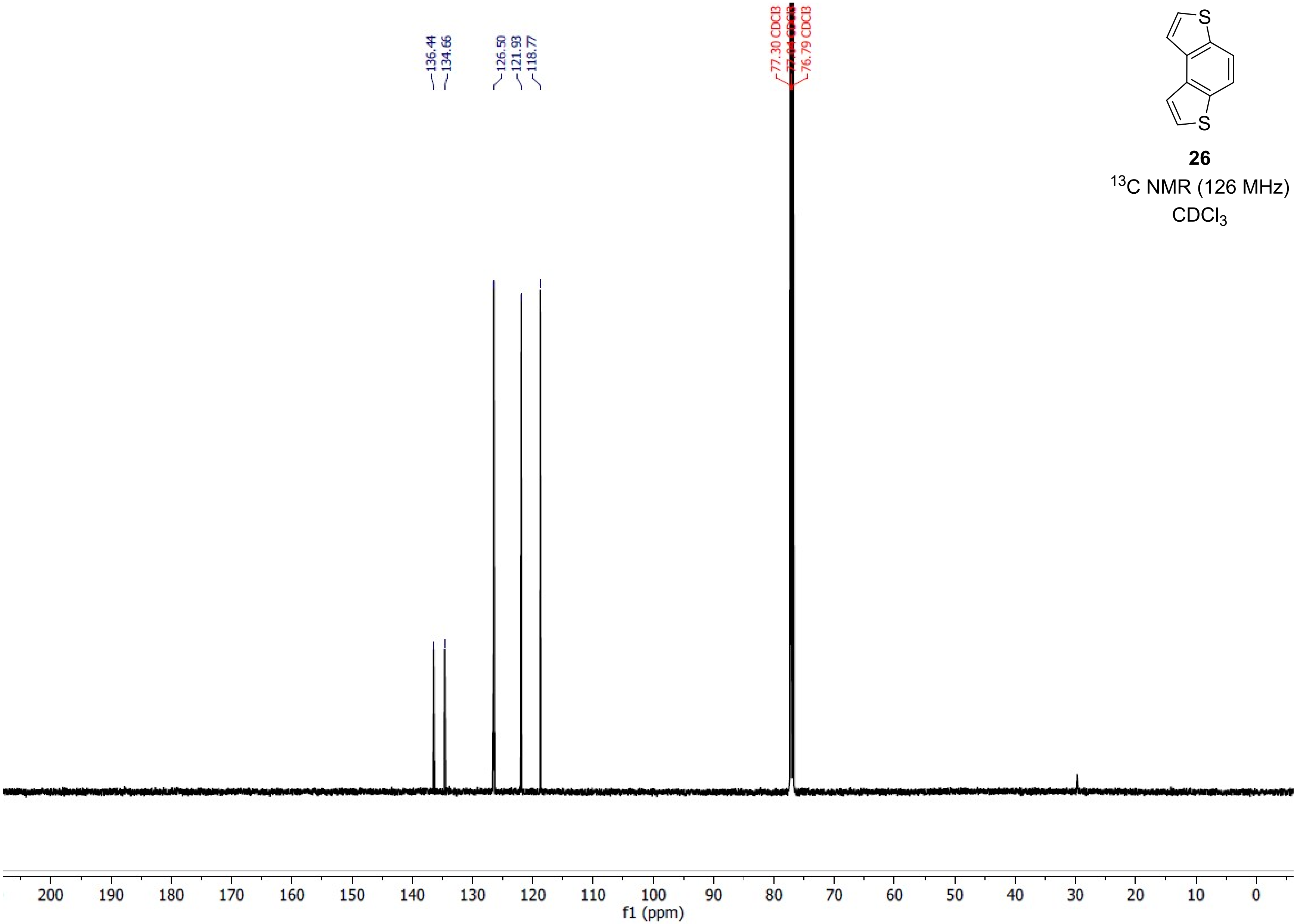

26

^{13}C NMR (126 MHz)

$CDCl_3$

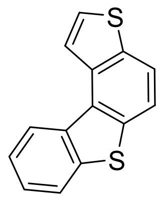

27

^{1}H NMR (500 MHz)

$CDCl_3$

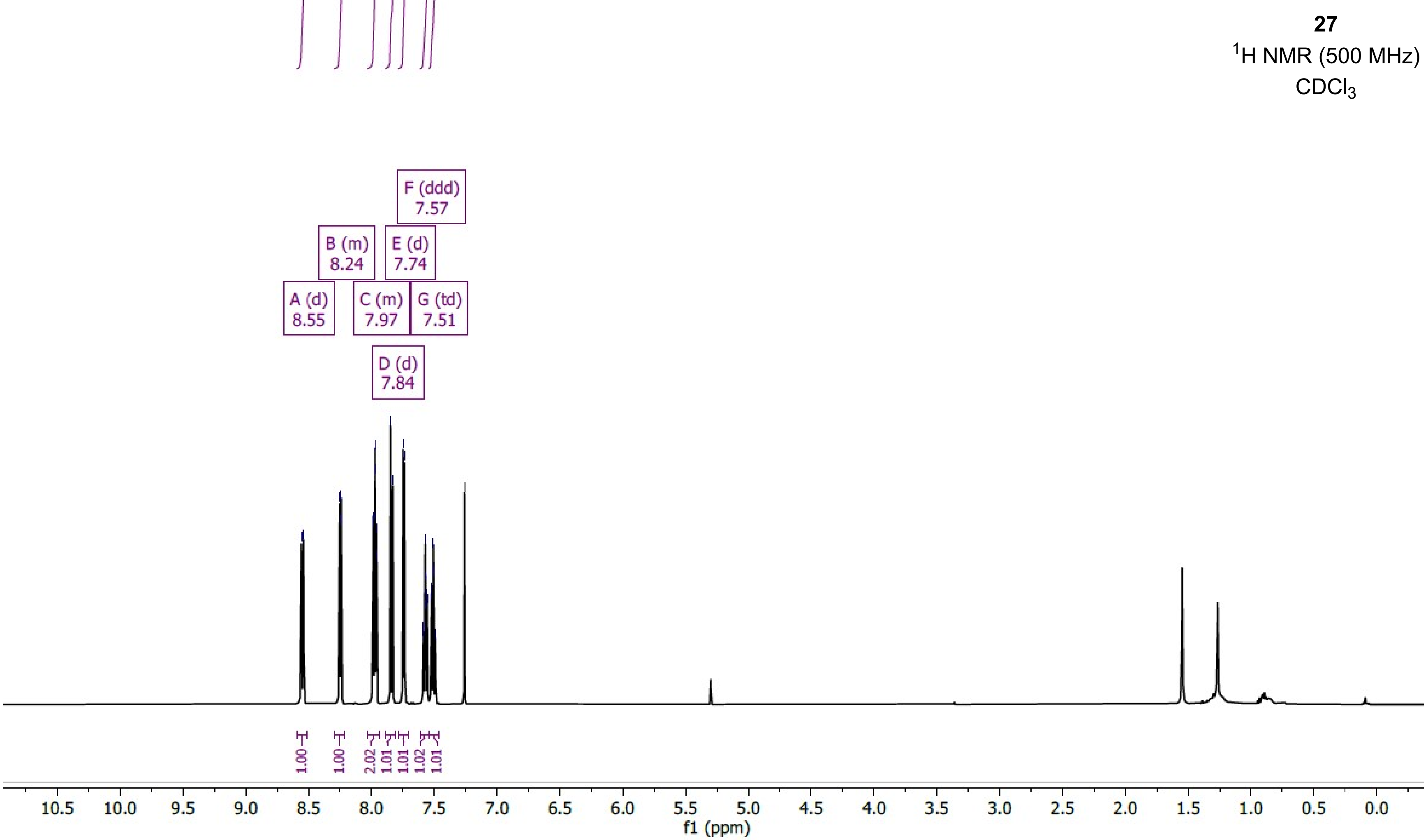

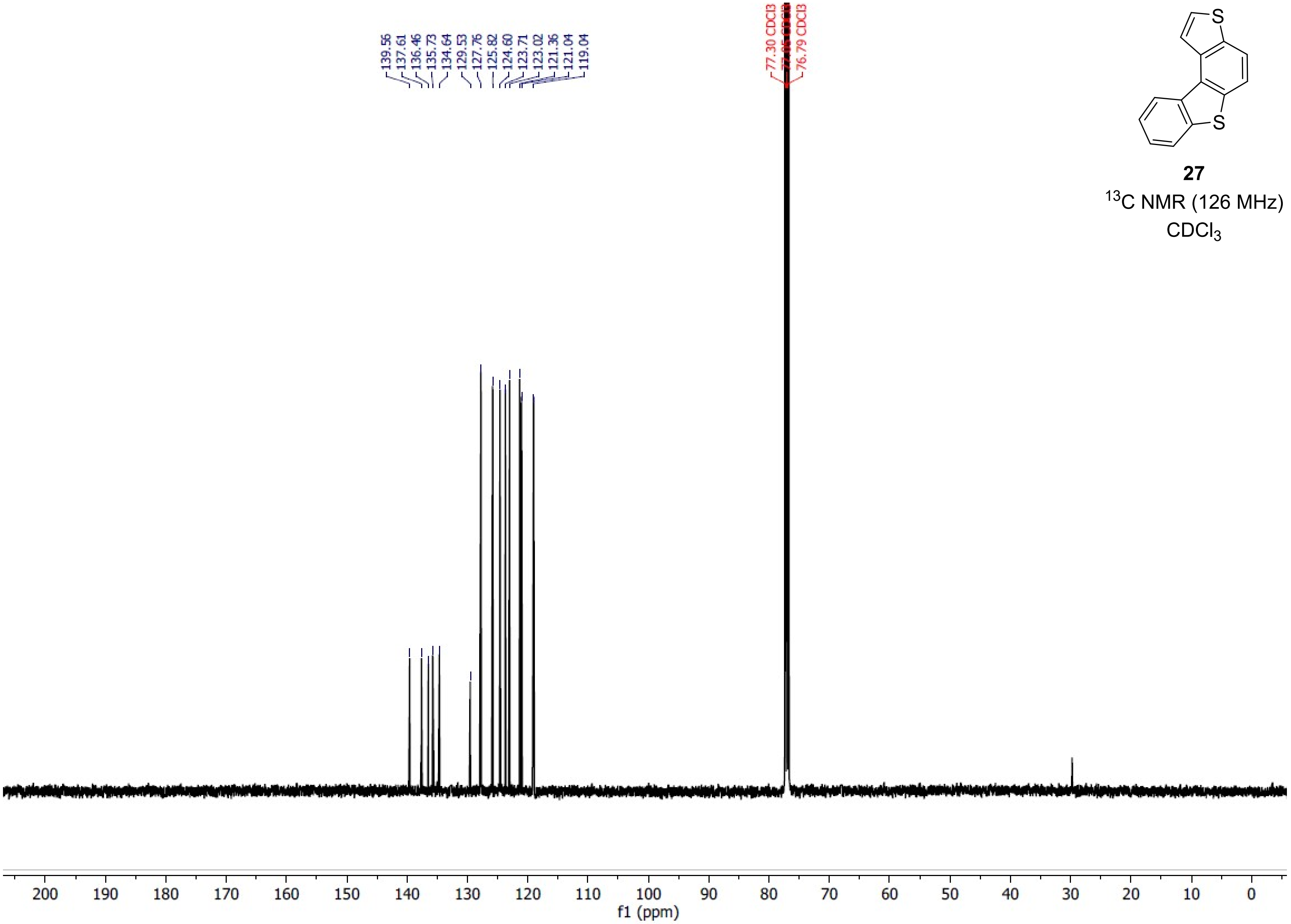

27

^{13}C NMR (126 MHz)

$CDCl_3$

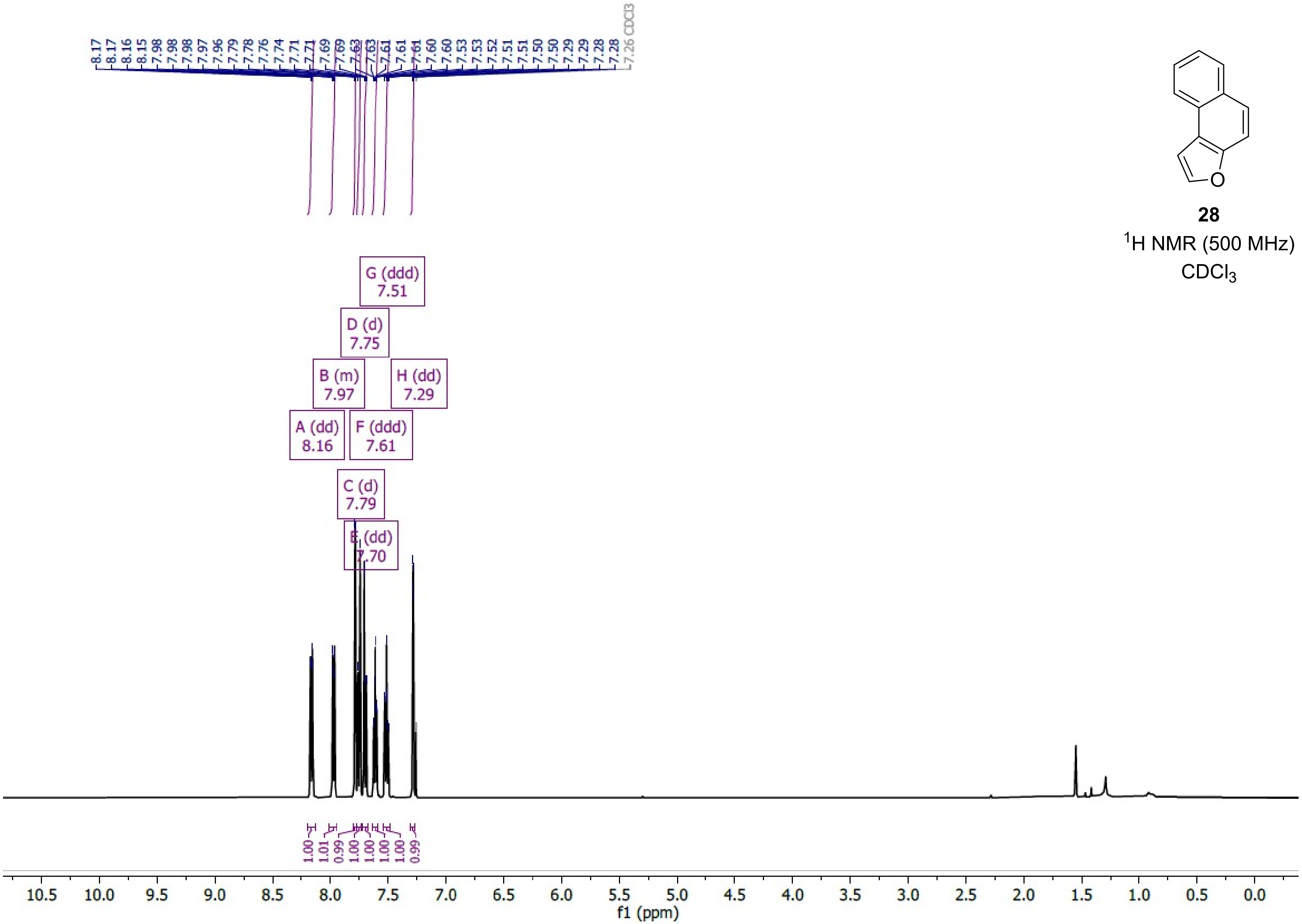

28

^{1}H NMR (500 MHz)

$CDCl_3$

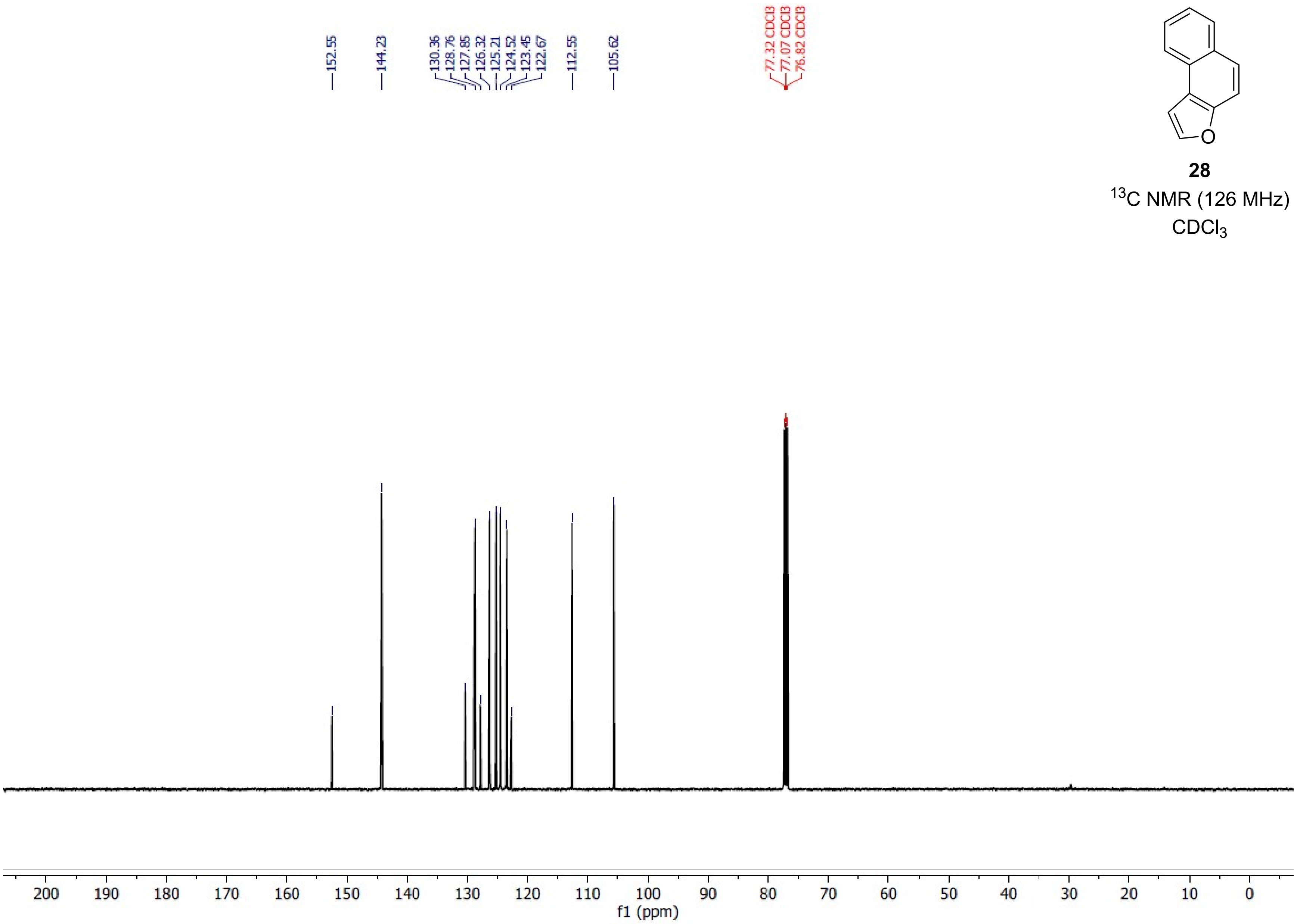

28

^{13}C NMR (126 MHz)

$CDCl_3$

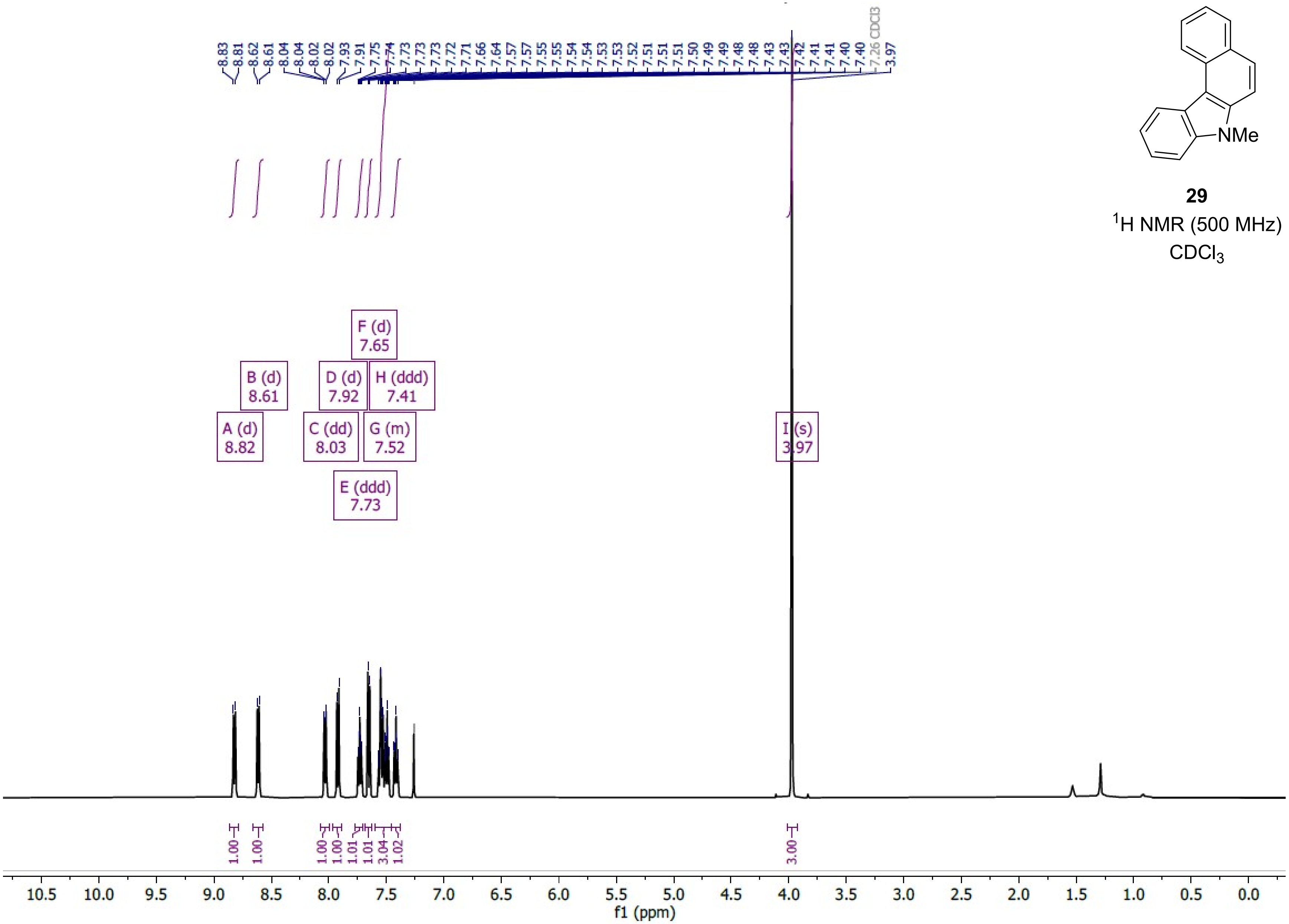

29

^{1}H NMR (500 MHz)

$CDCl_3$

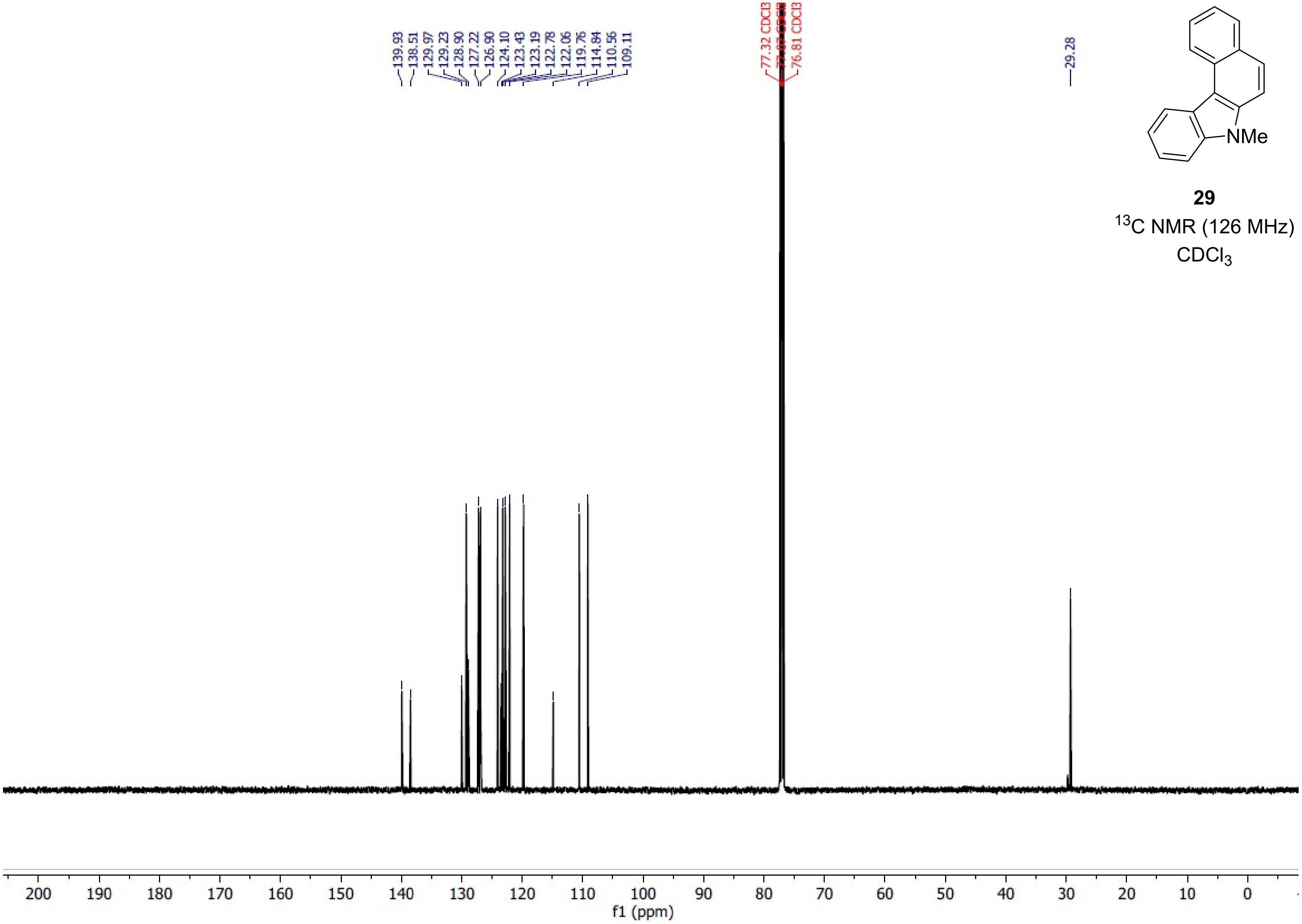

29

^{13}C NMR (126 MHz)

$CDCl_3$

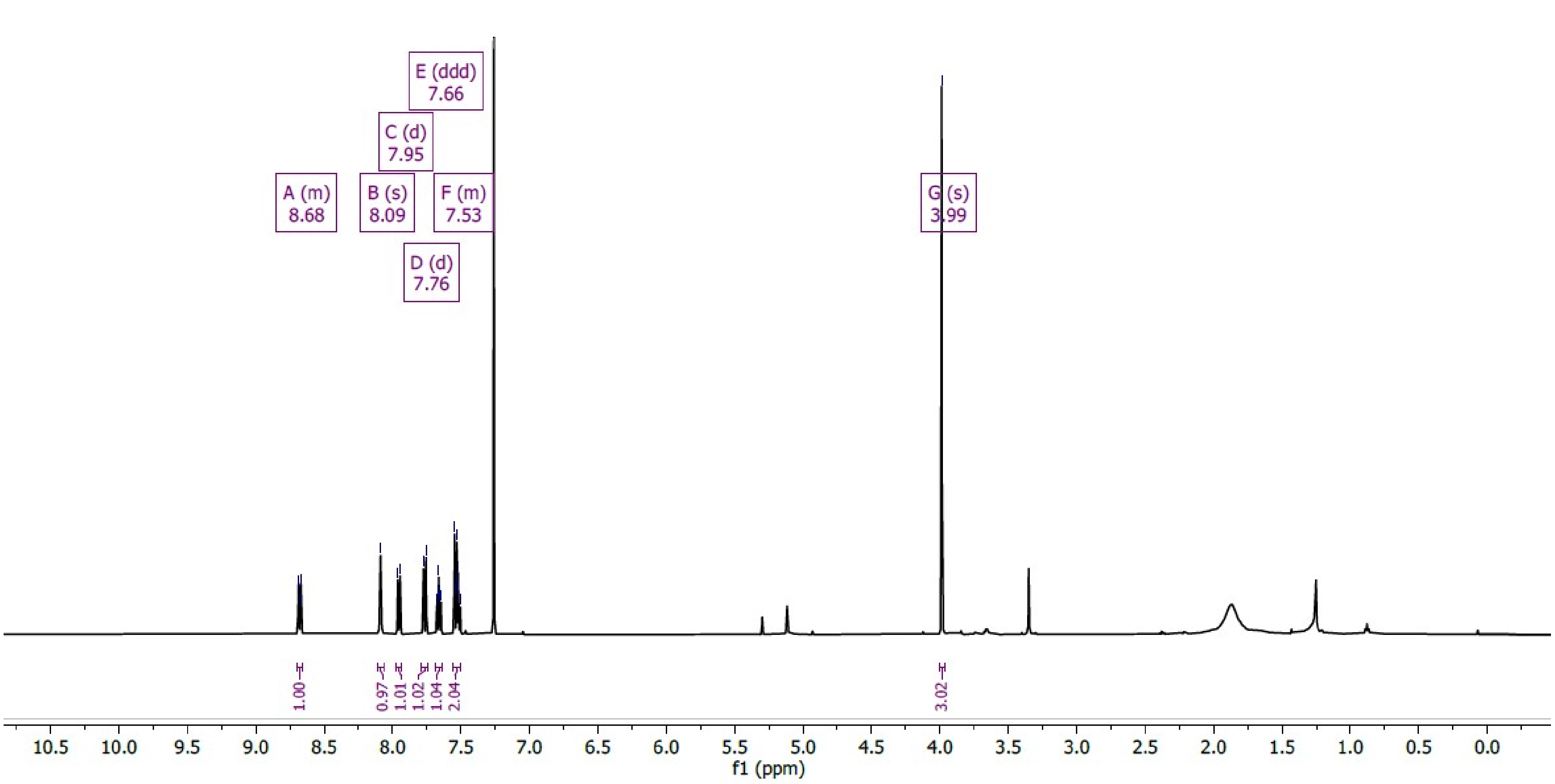

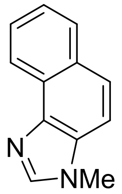

30

^{1}H NMR (500 MHz)

$CDCl_3$

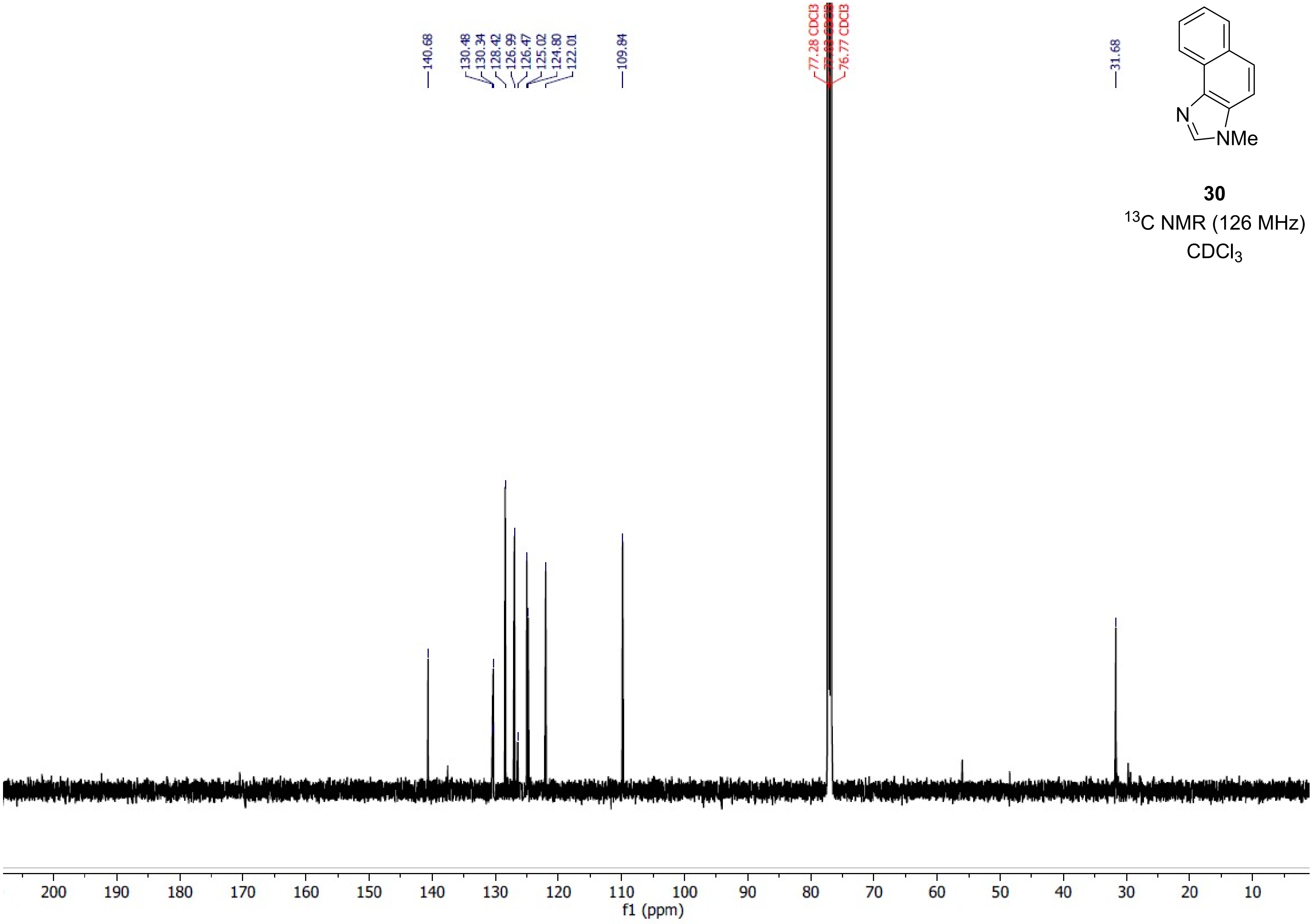

30

^{13}C NMR (126 MHz)

$CDCl_3$

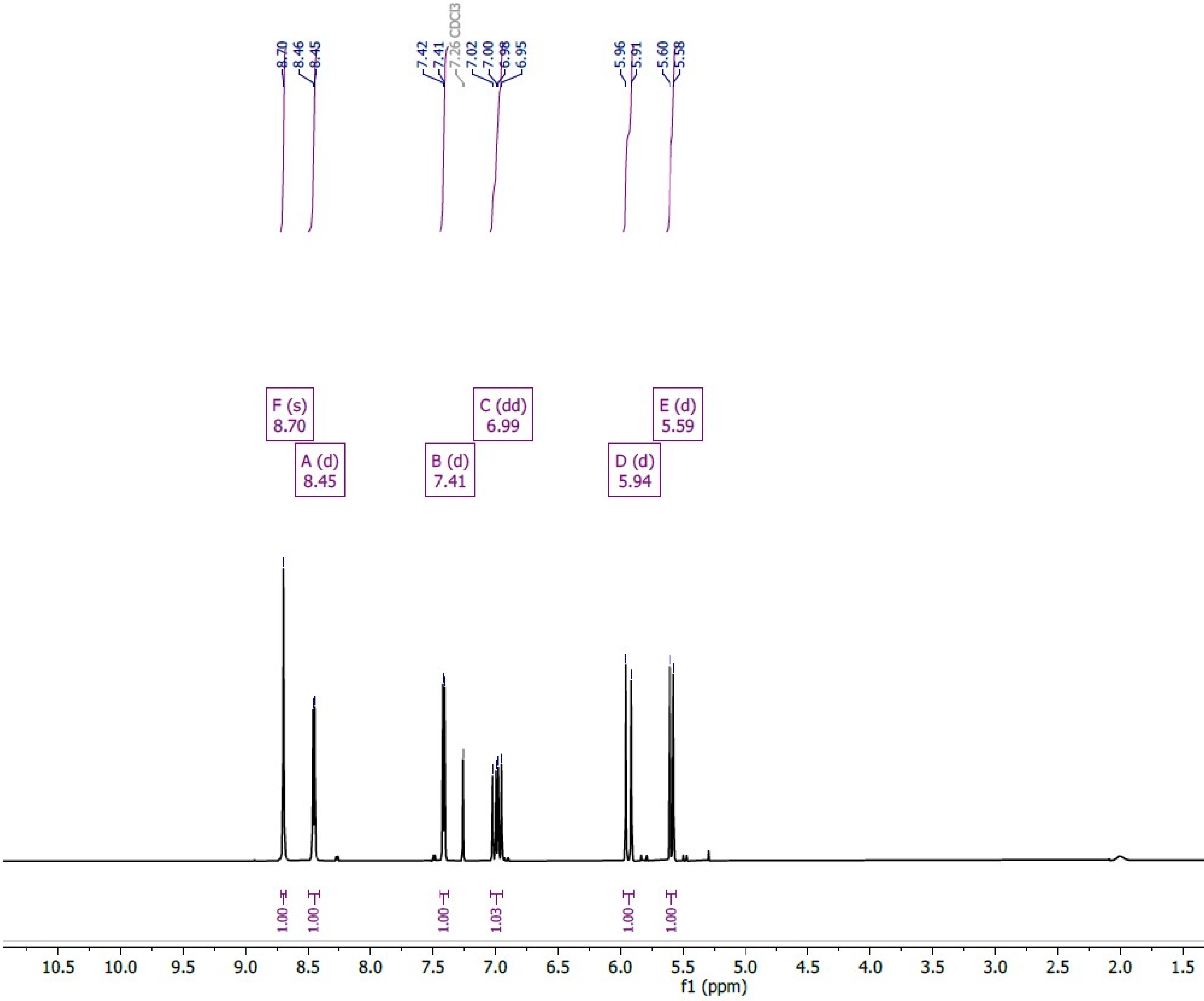

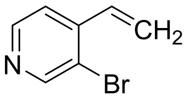

66

^{1}H NMR (400 MHz)

$CDCl_3$

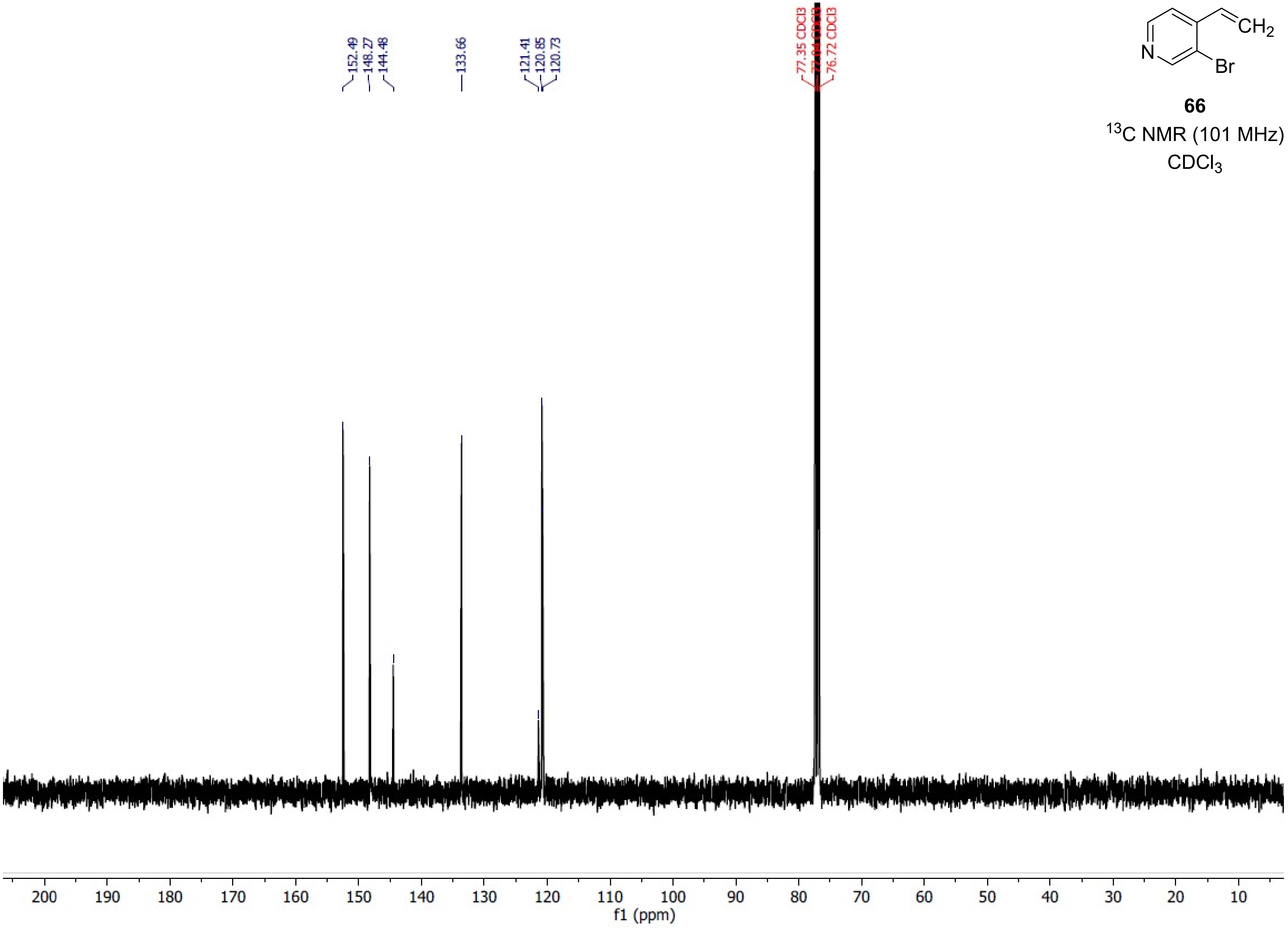

66

^{13}C NMR (101 MHz)

$CDCl_3$

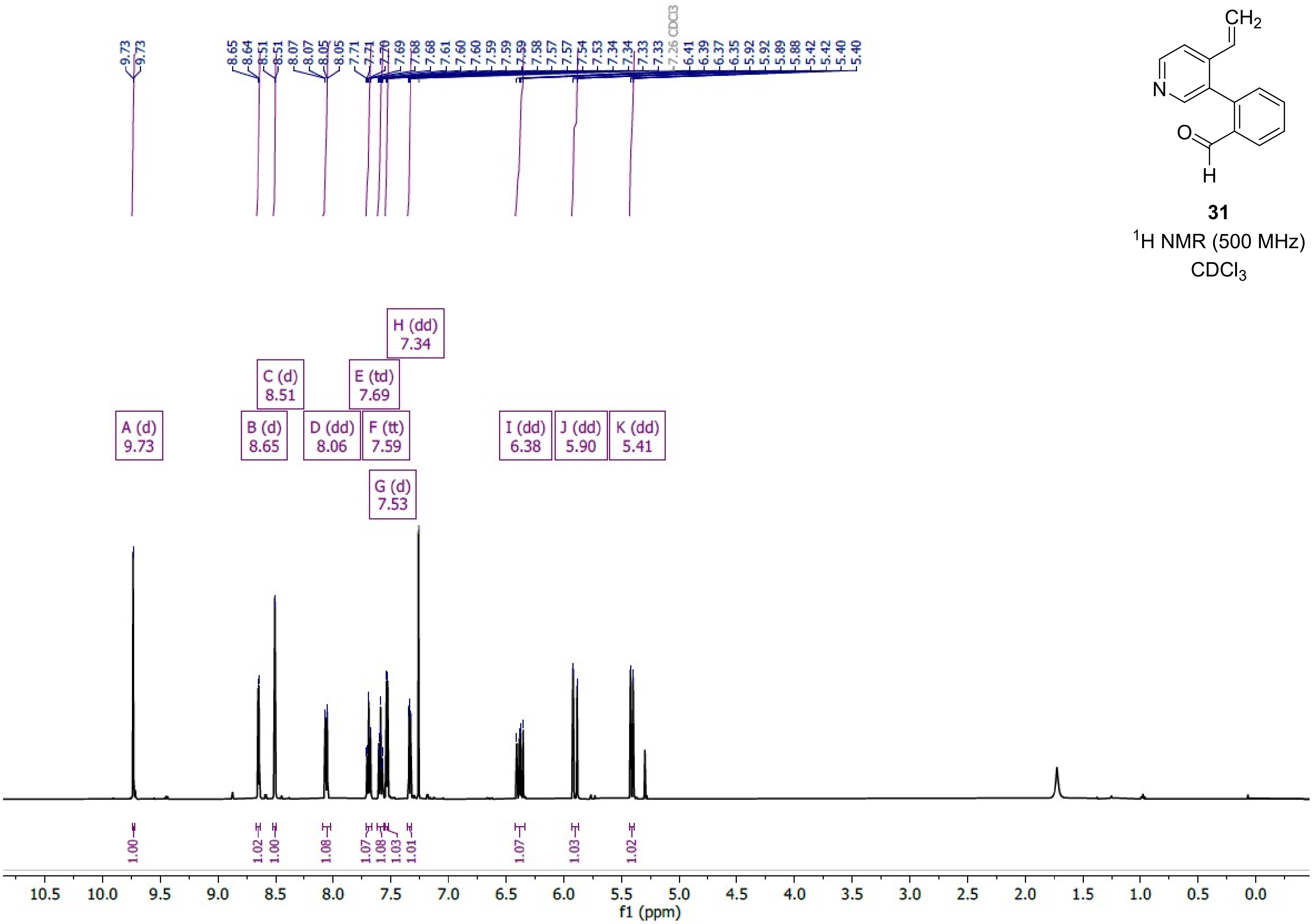

31

^{1}H NMR (500 MHz)

$CDCl_3$

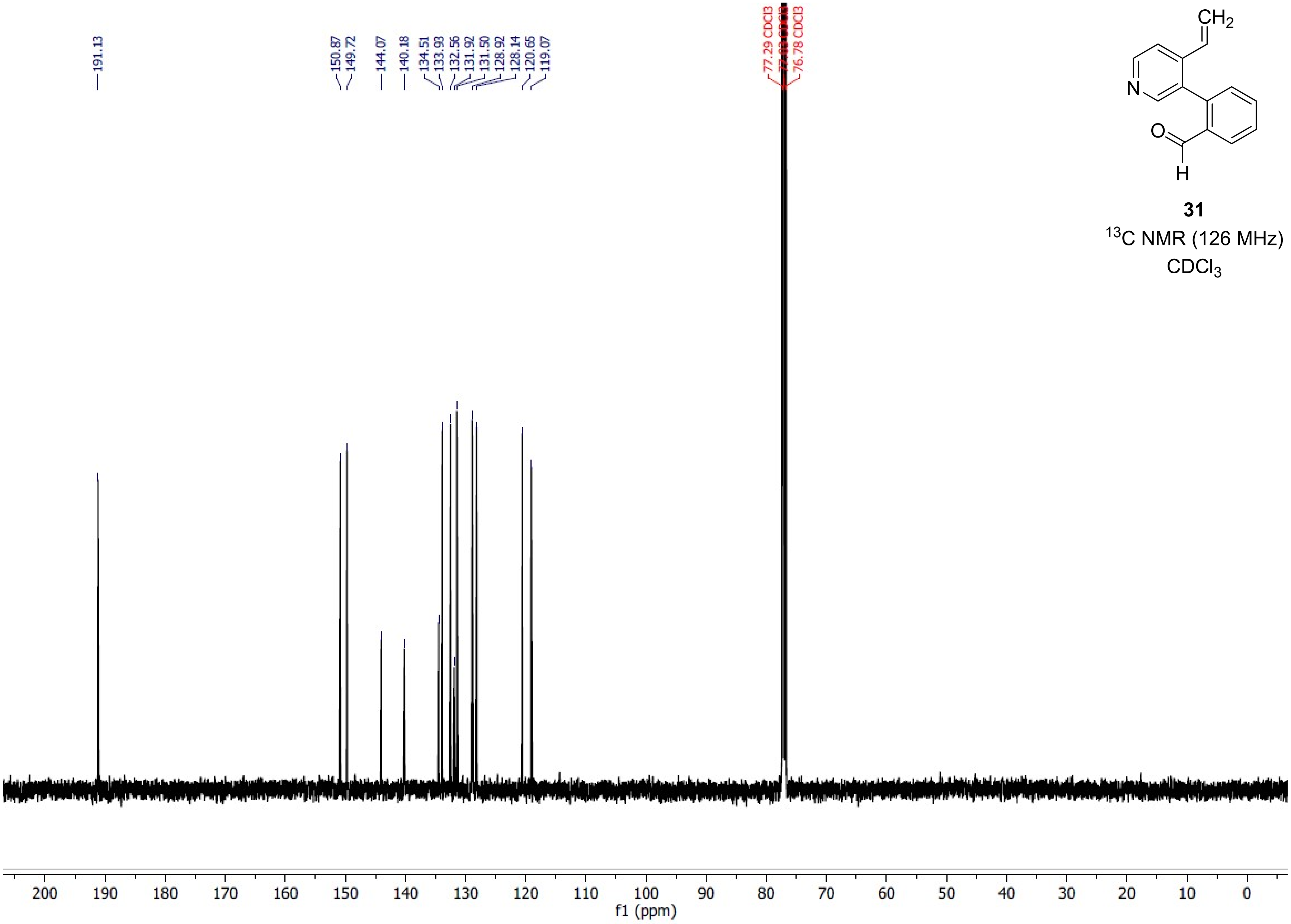

31

^{13}C NMR (126 MHz)

$CDCl_3$

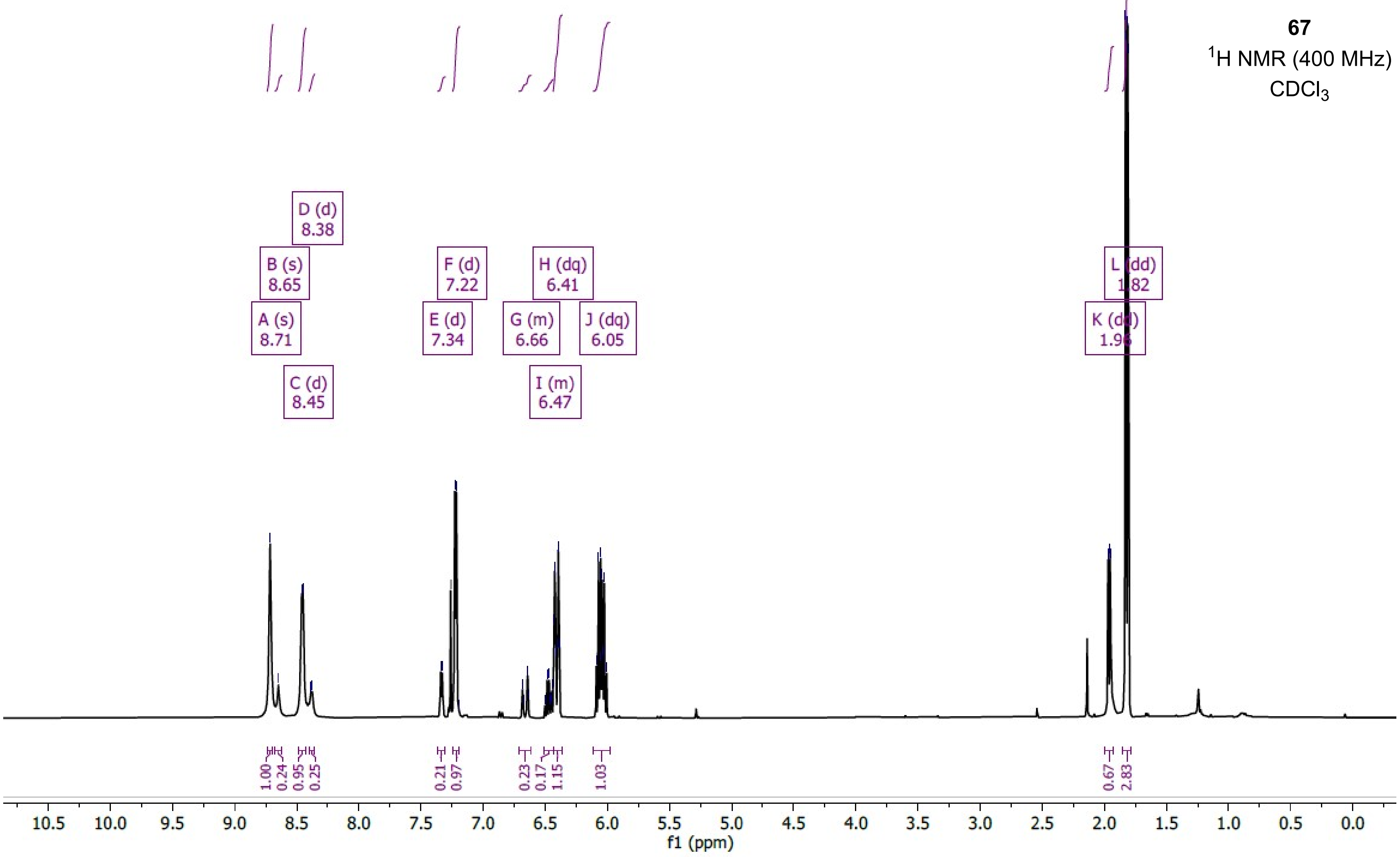

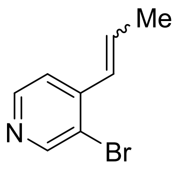

67

^{1}H NMR (400 MHz)

$CDCl_3$

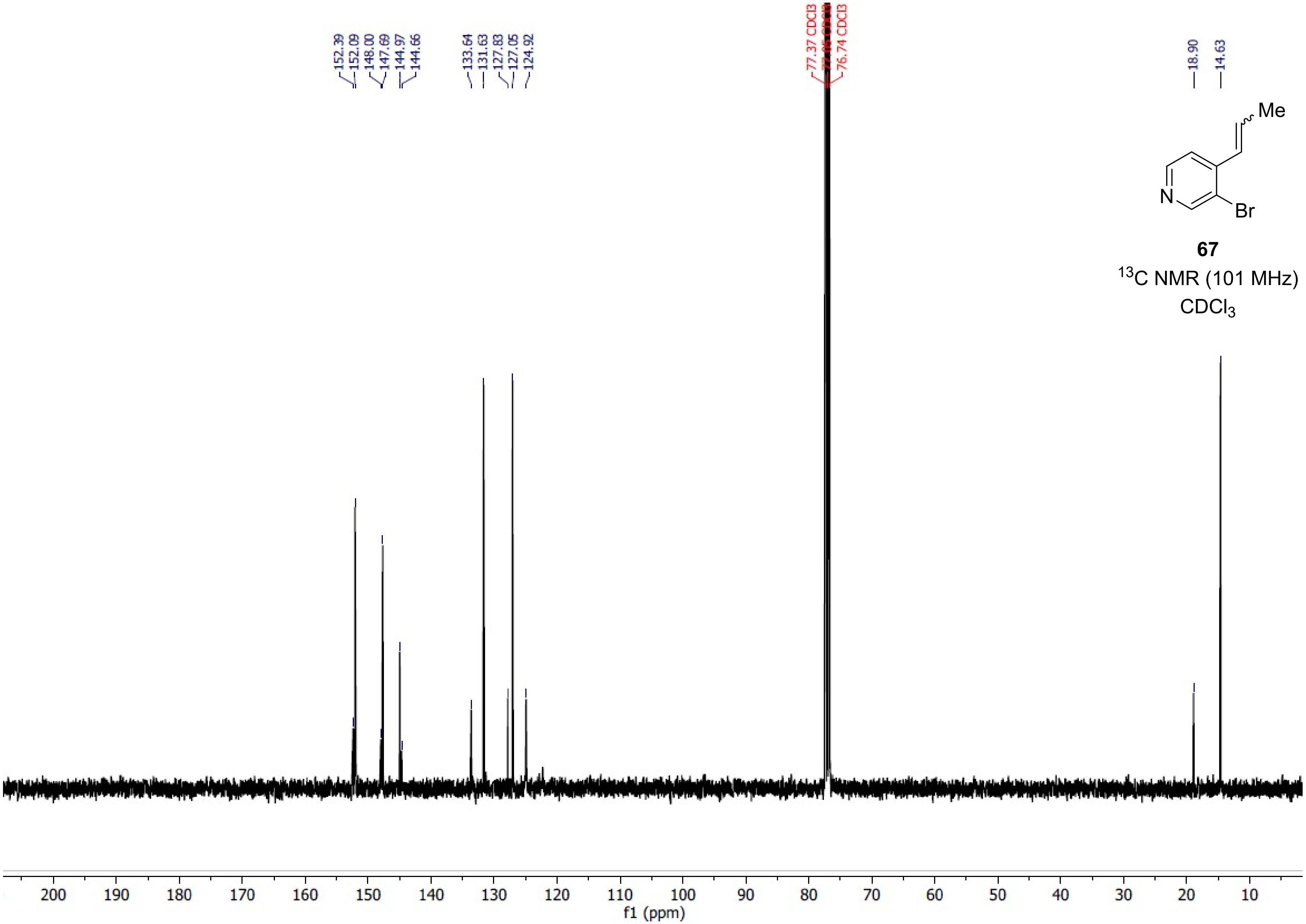

67

^{13}C NMR (101 MHz)

$CDCl_3$

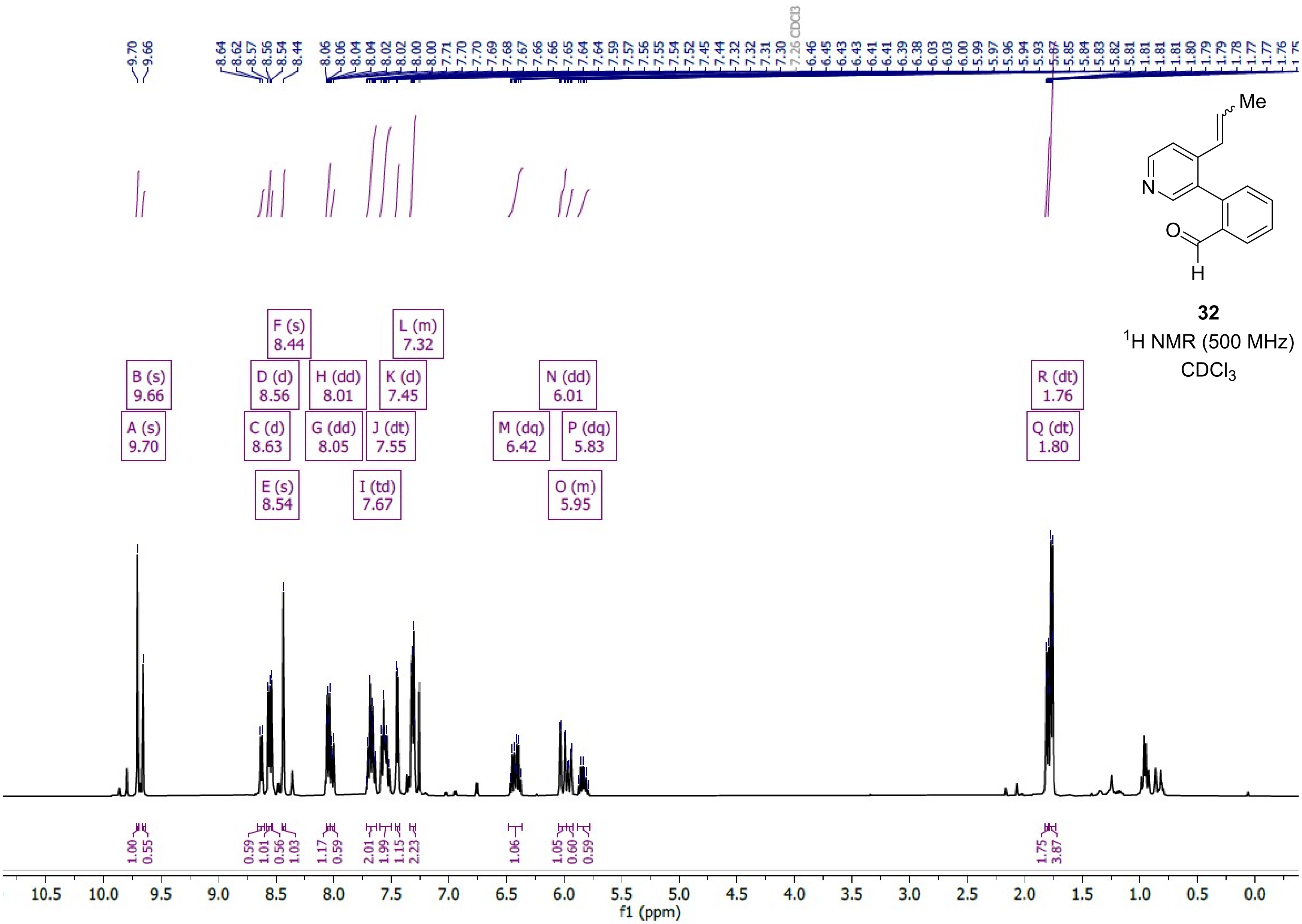

32

^{1}H NMR (500 MHz)

$CDCl_3$

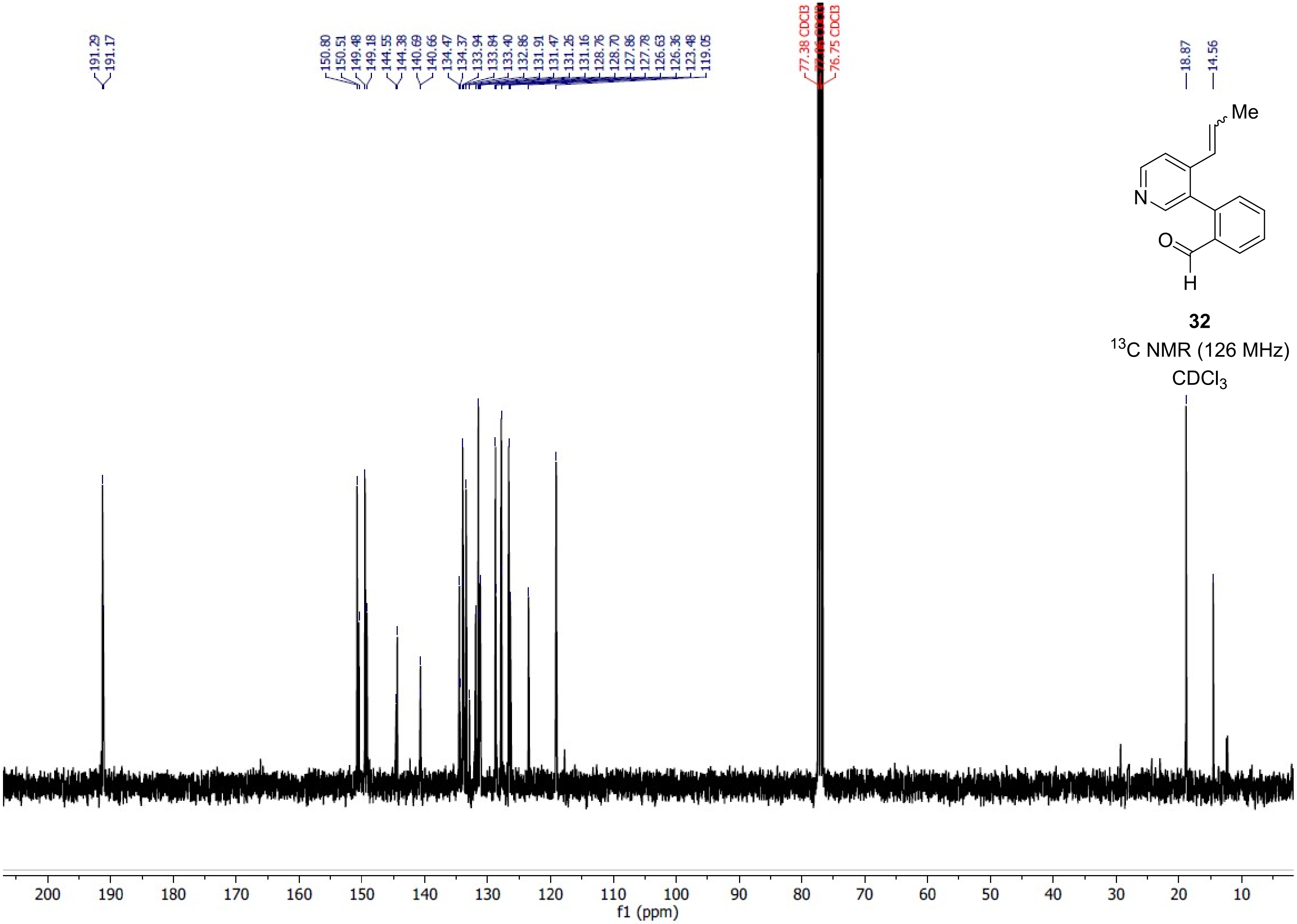

32

^{13}C NMR (126 MHz)

$CDCl_3$

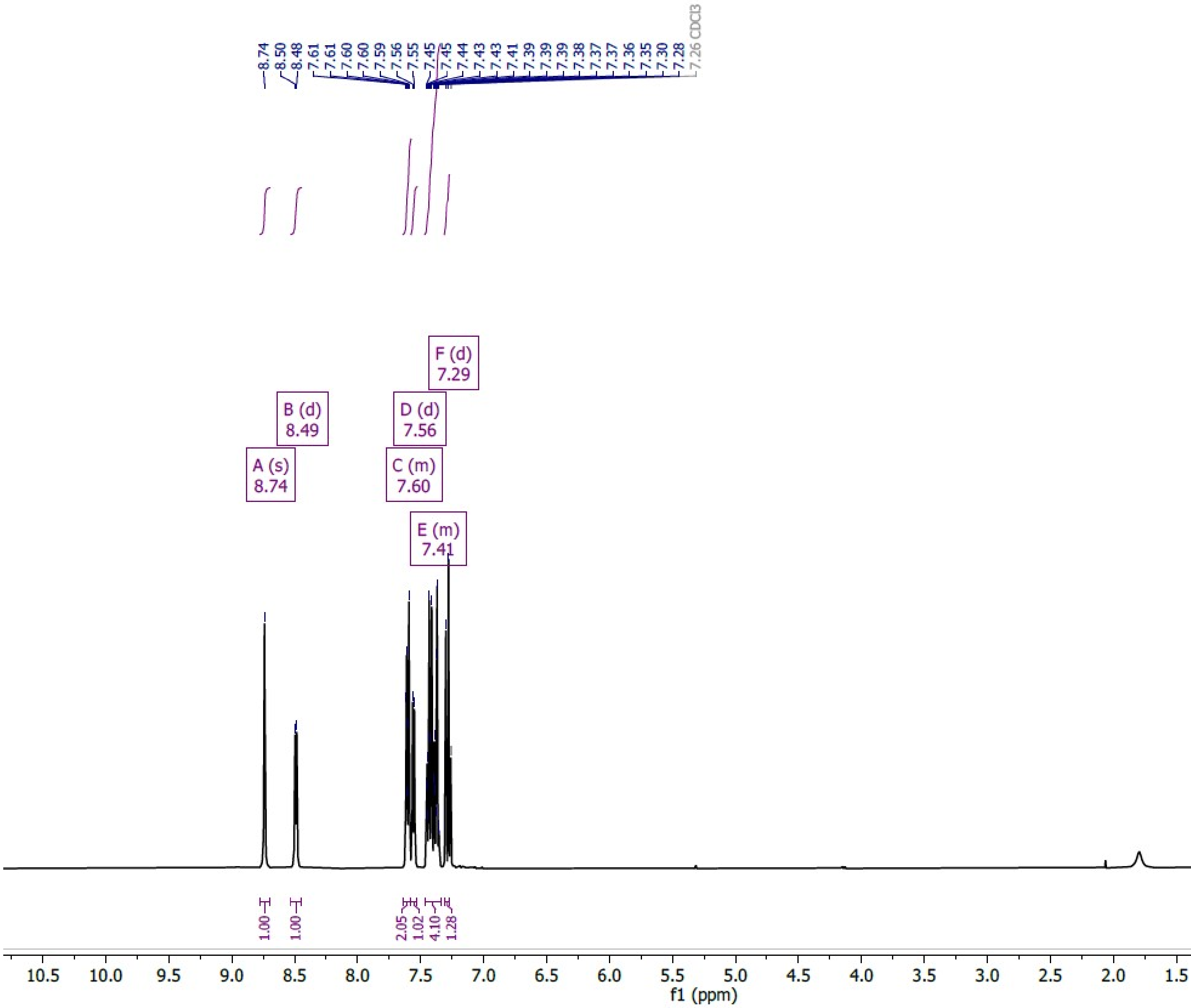

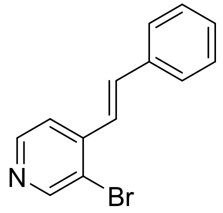

68

^{1}H NMR (400 MHz)

$CDCl_3$

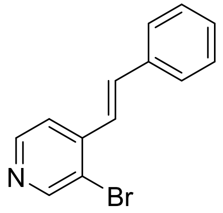

68

^{13}C NMR (101 MHz)

$CDCl_3$

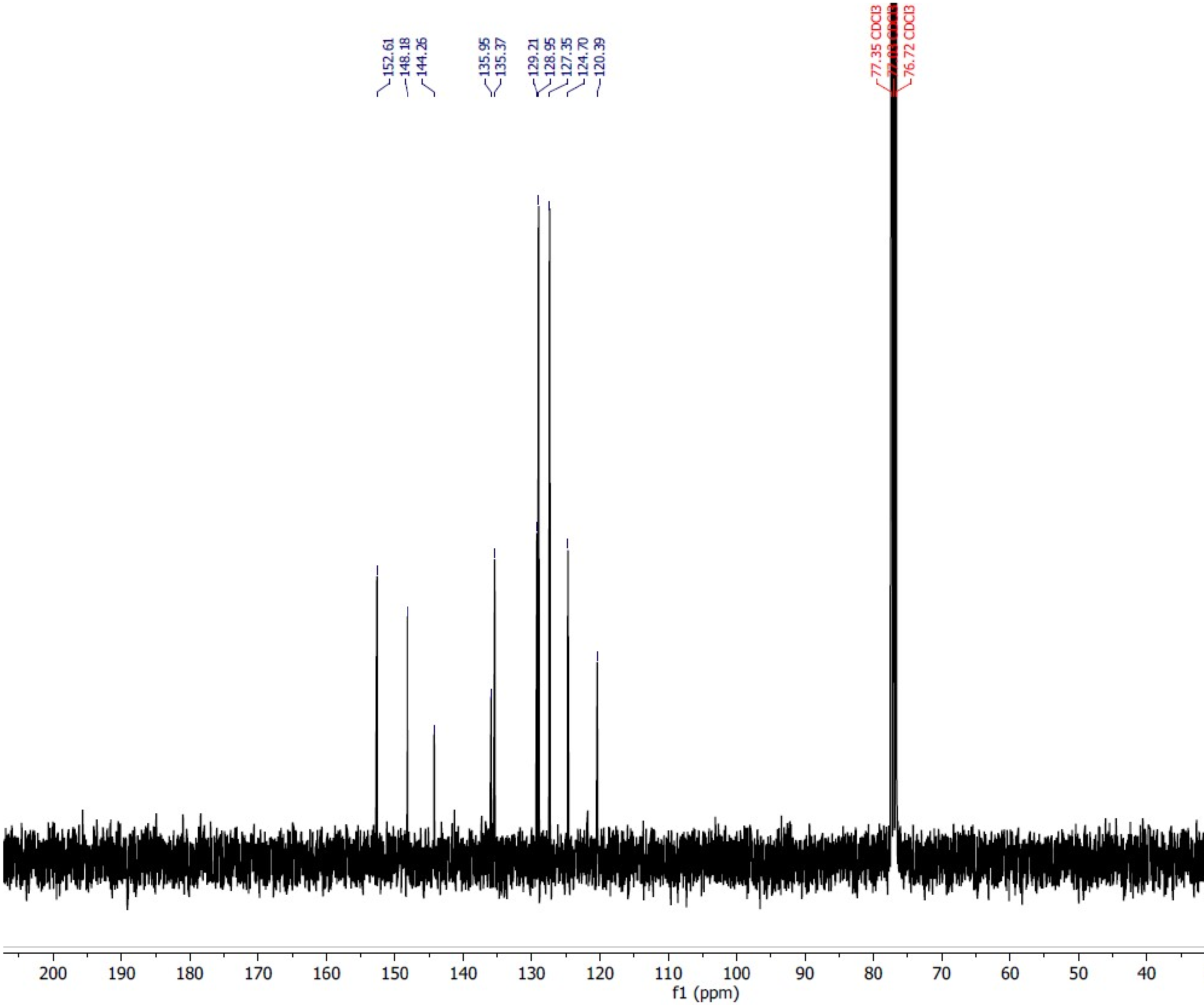

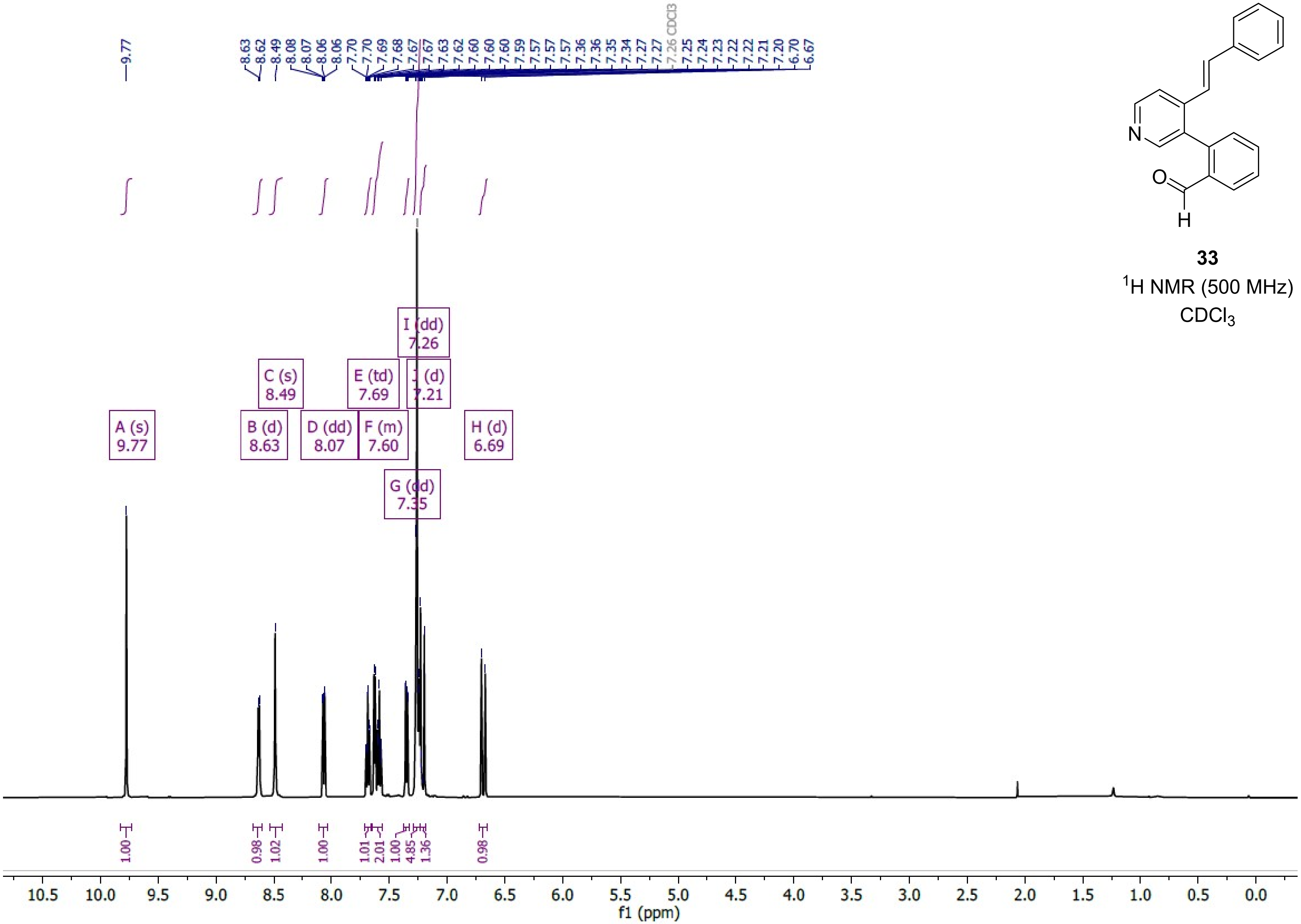

33

^{1}H NMR (500 MHz)

$CDCl_3$

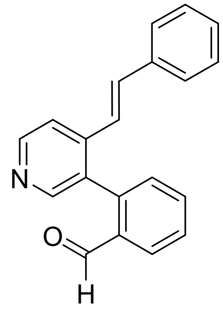

33

^{13}C NMR (126 MHz)

$CDCl_3$

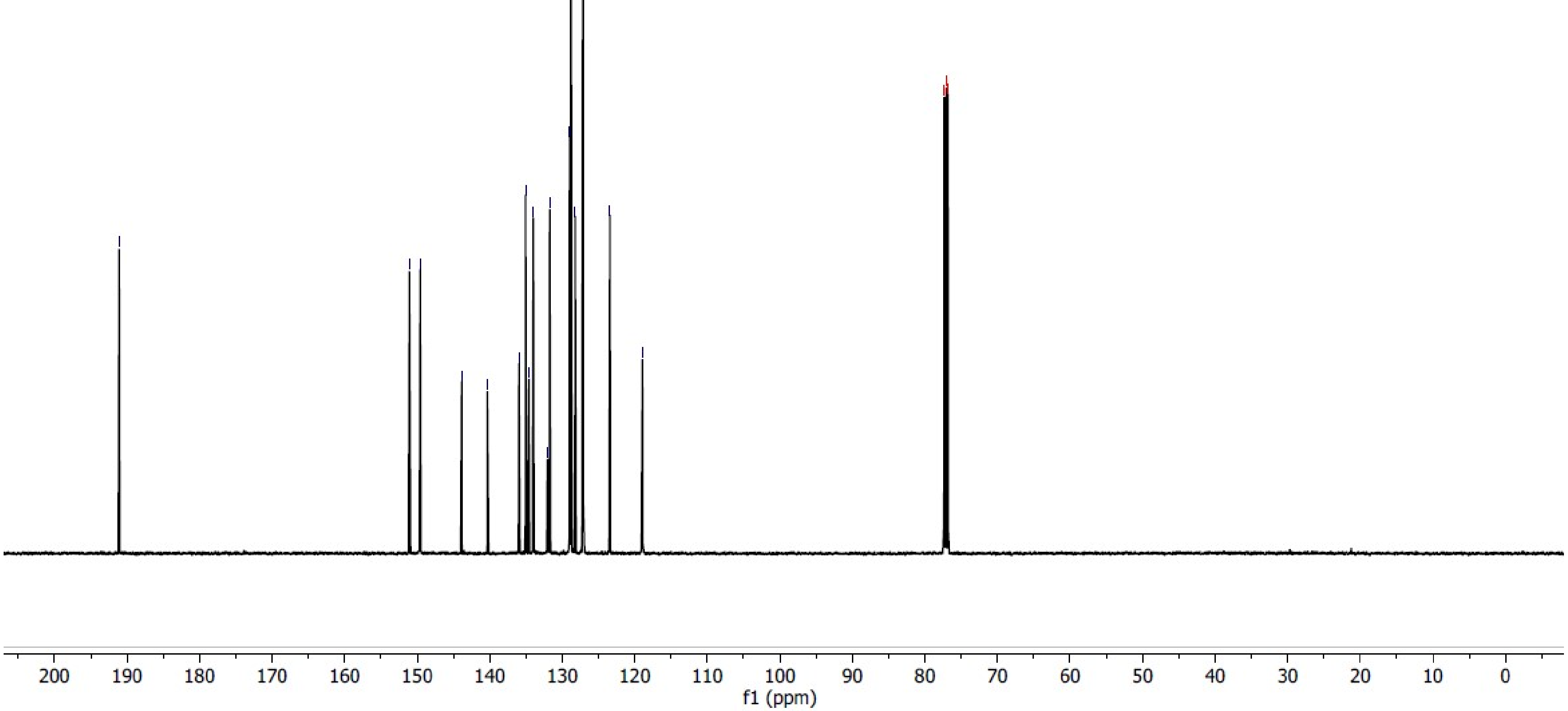

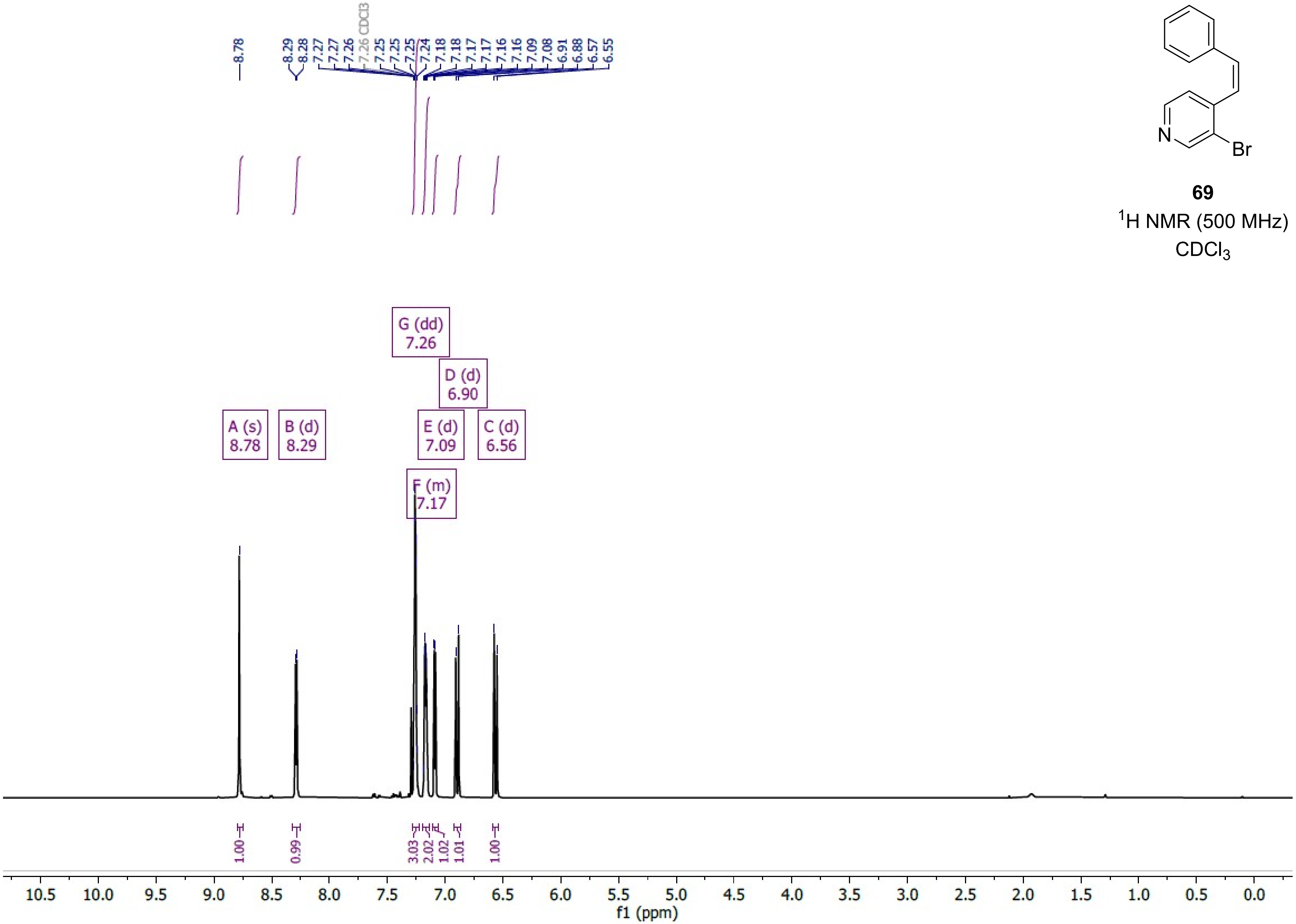

69

^{1}H NMR (500 MHz)

$CDCl_3$

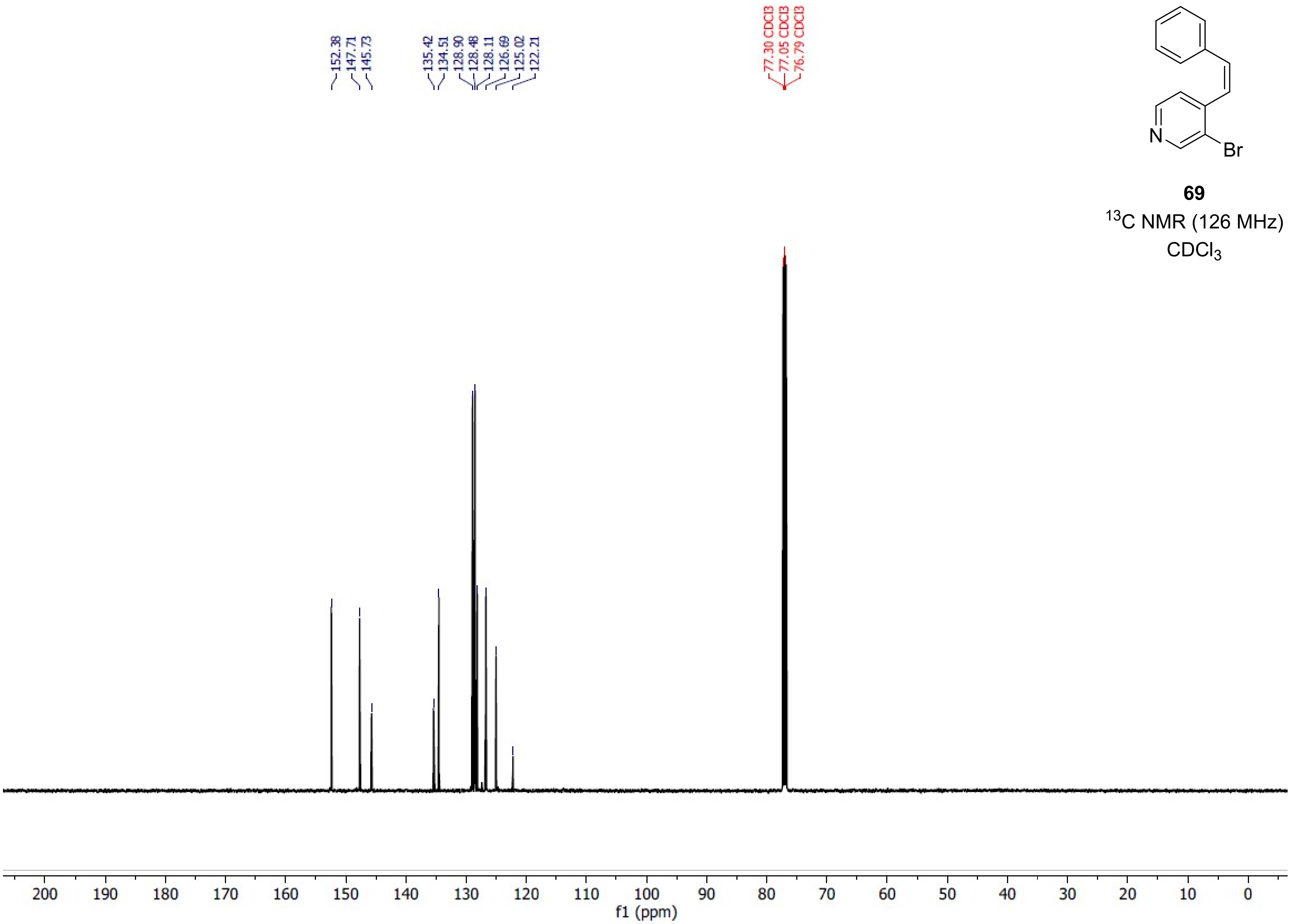

69

^{13}C NMR (126 MHz)

$CDCl_3$

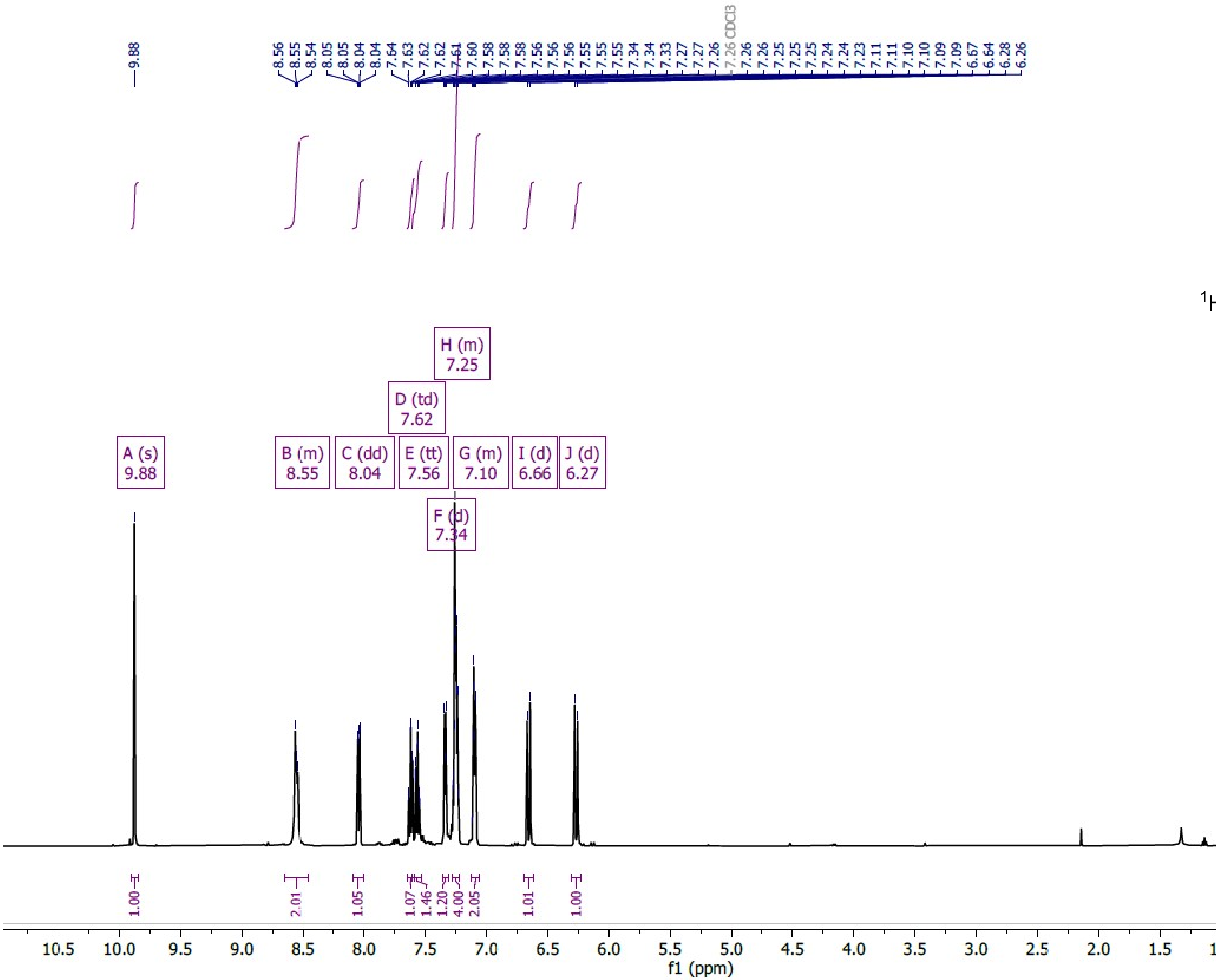

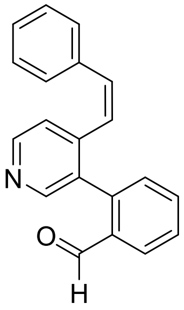

34

^{1}H NMR (500 MHz)

$CDCl_3$

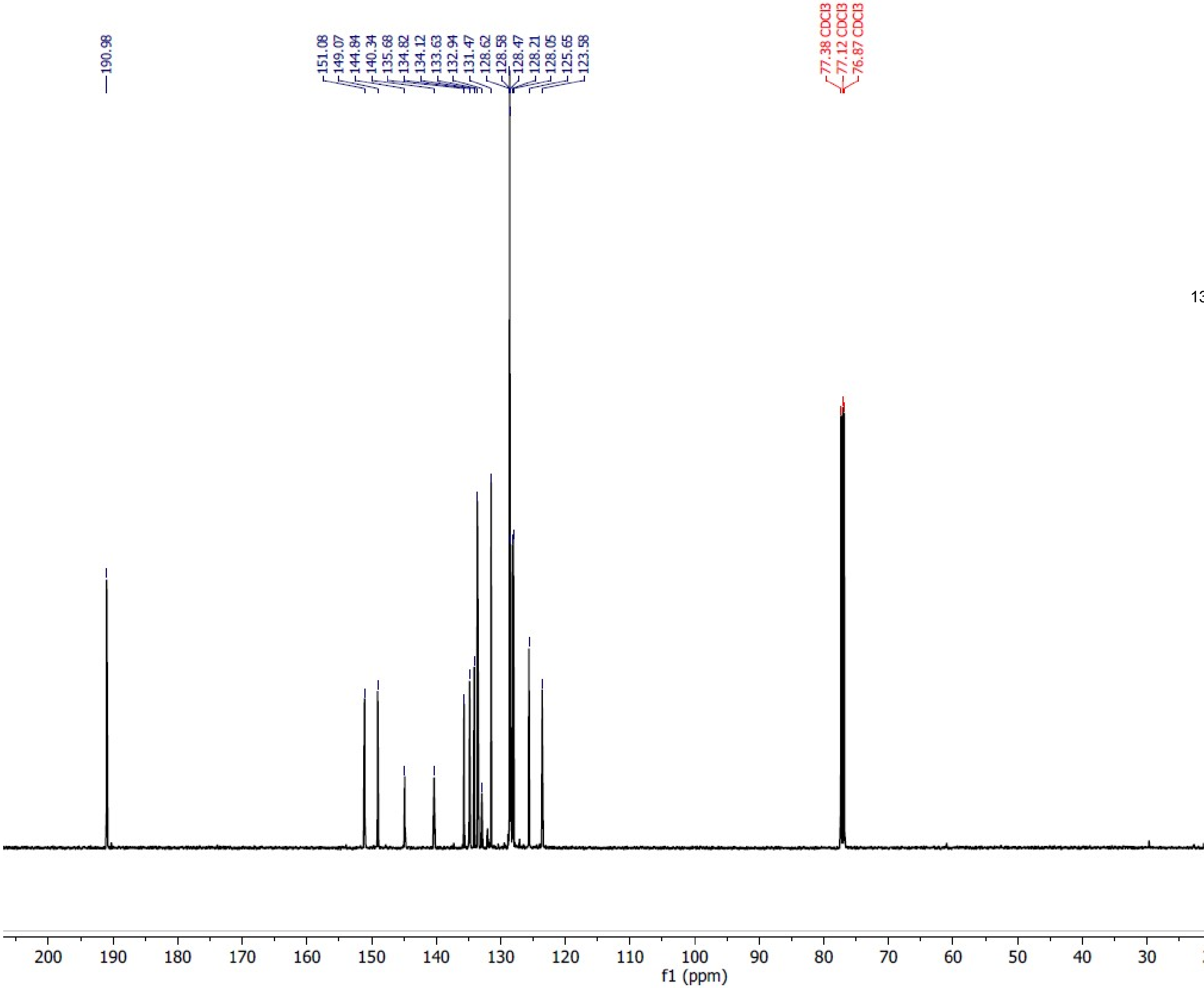

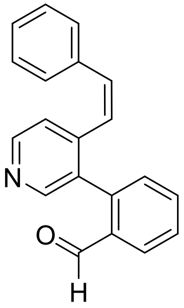

34

^{13}C NMR (126 MHz)

$CDCl_3$
